# Phase 1b Randomized Trial and Follow-Up Study in Uganda of the Blood-Stage Malaria Vaccine Candidate BK-SE36

**DOI:** 10.1371/journal.pone.0064073

**Published:** 2013-05-28

**Authors:** Nirianne Marie Q. Palacpac, Edward Ntege, Adoke Yeka, Betty Balikagala, Nahoko Suzuki, Hiroki Shirai, Masanori Yagi, Kazuya Ito, Wakaba Fukushima, Yoshio Hirota, Christopher Nsereko, Takuya Okada, Bernard N. Kanoi, Kohhei Tetsutani, Nobuko Arisue, Sawako Itagaki, Takahiro Tougan, Ken J. Ishii, Shigeharu Ueda, Thomas G. Egwang, Toshihiro Horii

**Affiliations:** 1 Department of Molecular Protozoology, Research Institute for Microbial Diseases, Osaka University, Suita, Osaka, Japan; 2 The Research Foundation for Microbial Diseases of Osaka University, Suita, Osaka, Japan; 3 Med Biotech Laboratories, Kampala, Uganda; 4 Makerere University School of Public Health, Kampala, Uganda; 5 The Research Foundation for Microbial Diseases of Osaka University, Kanonji, Kagawa, Japan; 6 Department of Public Health, Faculty of Medicine, Osaka City University, Osaka, Japan; 7 Sumida Hospital, Medical Co. Living Together Association (LTA) Clinical Pharmacology Center, Tokyo, Japan; 8 Lira Medical Centre, Lira, Uganda; 9 Laboratory of Adjuvant Innovation, National Institute of Biomedical Innovation, Ibaraki City, Osaka, Japan; 10 Laboratory of Vaccine Science, Immunology Frontier Research Center, World Premier Institute for Immunology, Osaka University, Suita, Osaka, Japan; Aeras, United States of America

## Abstract

**Background:**

Up to now a malaria vaccine remains elusive. The *Plasmodium falciparum* serine repeat antigen-5 formulated with aluminum hydroxyl gel (BK-SE36) is a blood-stage malaria vaccine candidate that has undergone phase 1a trial in malaria-naive Japanese adults. We have now assessed the safety and immunogenicity of BK-SE36 in a malaria endemic area in Northern Uganda.

**Methods:**

We performed a two-stage, randomized, single-blinded, placebo-controlled phase 1b trial (Current Controlled trials ISRCTN71619711). A computer-generated sequence randomized healthy subjects for 2 subcutaneous injections at 21-day intervals in Stage1 (21–40 year-olds) to 1-mL BK-SE36 (*BKSE1.0*) (*n* = 36) or saline (*n* = 20) and in Stage2 (6–20 year-olds) to *BKSE1.0* (*n* = 33), 0.5-mL BK-SE36 (*BKSE0.5*) (*n* = 33), or saline (*n* = 18). Subjects and laboratory personnel were blinded. Safety and antibody responses 21-days post-second vaccination (Day42) were assessed. Post-trial, to compare the risk of malaria episodes 130–365 days post-second vaccination, Stage2 subjects were age-matched to 50 control individuals.

**Results:**

Nearly all subjects who received BK-SE36 had induration (Stage1, *n* = 33, 92%; Stage2, *n* = 63, 96%) as a local adverse event. No serious adverse event related to BK-SE36 was reported. Pre-existing anti-SE36 antibody titers negatively correlated with vaccination-induced antibody response. At Day42, change in antibody titers was significant for seronegative adults (1.95-fold higher than baseline [95% CI, 1.56–2.43], *p* = 0.004) and 6–10 year-olds (5.71-fold [95% CI, 2.38–13.72], *p* = 0.002) vaccinated with *BKSE1.0.* Immunogenicity response to *BKSE0.5* was low and not significant (1.55-fold [95% CI, 1.24–1.94], *p* = 0.75). In the ancillary analysis, cumulative incidence of first malaria episodes with ≥5000 parasites/µL was 7 cases/33 subjects in *BKSE1.0* and 10 cases/33 subjects in *BKSE0.5 vs.* 29 cases/66 subjects in the control group. Risk ratio for *BKSE1.0* was 0.48 (95% CI, 0.24–0.98; *p* = 0.04).

**Conclusion:**

BK-SE36 is safe and immunogenic. The promising potential of BK-SE36, observed in the follow-up study, warrants a double-blind phase 1/2b trial in children under 5 years.

**Trial Registration:**

Controlled-Trials.com ISRCTN71619711 ISRCTN71619711

## Introduction

A malaria vaccine is crucial in the face of continued high malaria transmission, increasing drug and insecticide resistance, and inadequate coverage of current control interventions [1]–[4]. The recent phase 3 trial of the anti-sporozoite vaccine RTS,S/AS01 [5], [6] showed modest protection in children aged 6–16 weeks. There is a strong justification for blood-stage vaccines since protection by the anti-sporozoite vaccine is not complete and long lasting [6], asexual-stage parasites cause symptomatic malaria, blood-stage antigens are targets of acquired immunity, and controlling parasite density may reduce the generation of gametocytes [7]–[9]. The majority of blood-stage vaccine candidates, however, fell short of expectation in field trials, hampered by antigenic variation, extensive polymorphism, showed conformation-dependence or, in some instances, have safety concerns [reviewed in 4].

The *Plasmodium falciparum* serine repeat antigen-5 (SERA5) is an abundant blood-stage antigen secreted in large amounts into the lumen of the parasitophorous vacuole [10], [11]. SERA5 was demonstrated to play an essential role in the parasite life cycle [12] and was among the first physiological substrate identified for a serine protease implicated for parasite egress [13]. SERA5 was selected for clinical development on the basis of the following: (i) epidemiological studies showing high antibody titers that inversely correlate with malaria symptoms and severe disease; (ii) *in vitro* studies demonstrating induction of antibodies that are inhibitors of parasite growth, exert antibody-dependent complement-mediated lysis of schizonts, or antibody-dependent monocyte-mediated parasite growth inhibition; and (iii) animal studies demonstrating protection against *P. falciparum* challenge in non-human primates [14], [15]. Analysis of the *sera5* sequences from 445 *P. falciparum* world-wide samples revealed no strong evidence for positive selection acting on this gene [16]. A recombinant form of SERA5 N-terminal domain, SE36, was mass produced, purified under GMP conditions and formulated with aluminum hydroxide gel to yield BK-SE36. The safety and immunogenicity of BK-SE36 was demonstrated in a phase1a trial in malaria naïve Japanese adults [14]. As part of the BK-SE36 vaccine development plan, we report the safety and immunogenicity results of a two-stage randomized trial in Lira, Uganda. Additionally, because we observed numerous malaria episodes during Stage1, we were interested in comparing the risk of malaria episodes in Stage2 subjects 130–365 days post-second vaccination.

## Methods

The protocols for this trial and follow-up study, as well as supporting CONSORT checklist are available as supporting information; see Protocol S1, Protocol S2 and Checklist S1.

### Ethics Statement

The trial was conducted in compliance with the study protocol, the International Conference on Harmonization’s Good Clinical Practice standards, the Declaration of Helsinki and Uganda regulatory requirements (Uganda National Council for Science and Technology [UNCST] National Guidelines for Research Involving Humans as Research Participants, March 2007; National Drug Authority [NDA] Guidelines for the Conduct of Clinical Trials, December 2007). Approvals for the protocol, subject information and informed consent forms were obtained from the ethical institutional review committees (IRC) of Osaka University (Japan), Research Foundation for Microbial Diseases of Osaka University (BIKEN-IRC) (Japan) and Med Biotech Laboratories (MBL-IRC: IRB-00003990-MBL-BIOMEDICAL) (Uganda). Regulatory approval was obtained from UNCST (HS 635) and NDA (633/ESR/NDA/DID-11/09 and 135/ESR/NDA/DID-08/2010). UNCST provided introductory letters to the community. Permission to import (012/P/2010 and 258/P/2010) and administer the investigational product was granted by NDA. During the conduct of Stage1, the trial protocol was amended to reflect clarifications and changes in the inclusion and exclusion criteria for Stage2, deemed necessary based on Stage1 screening. Both Stage1 and Stage2 are registered under one identifier at Current Controlled trials ISRCTN71619711 (http://www.controlled-trials.com/isrctn/pf/71619711). The trial was monitored by Quintiles, a contract research organization (CRO), BIKEN and RIMD.

The follow-up study protocol and informed consent forms were reviewed and approved by RIMD-IRC, MBL-IRC (IRB-00003995-MBL-BIOMEDICAL), and UNCST (HS 866). RIMD monitored the follow-up study.

Study approvals are available as supporting information in Text S1.

### Study Setting and Design

The randomized, single-blinded phase 1b trial and follow-up study was conducted at Lira Medical Center (LMC), Lira district, Uganda between April 2010 to Feb. 2011; and Mar. to Nov. 2011, respectively. The site is located 347 km north of Kampala, in a region with perennial holoendemic malaria [3], [17], [18].

**Figure 1:**
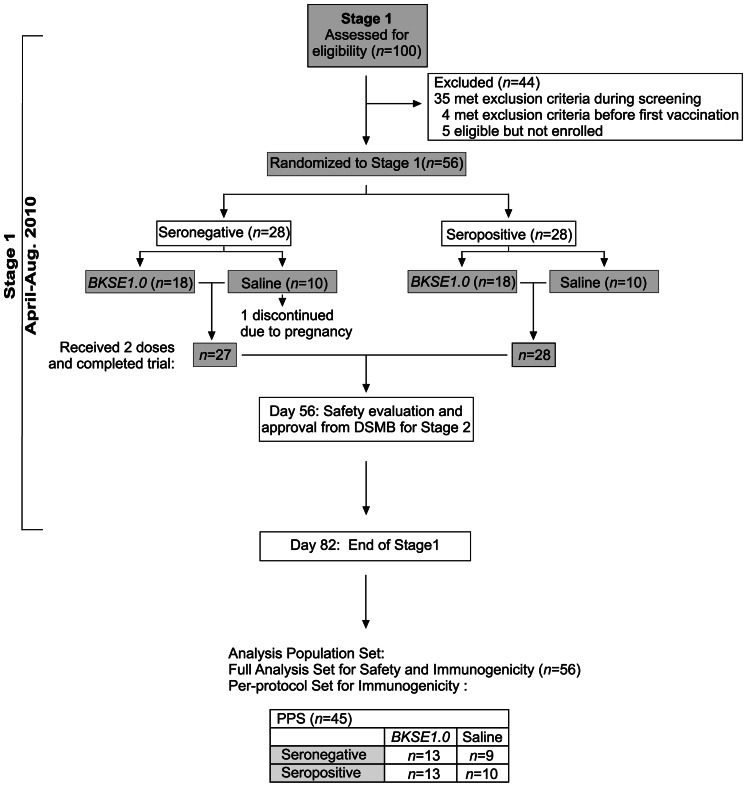
Enrollment and outcomes for Stage1. The number of subjects screened, those excluded (due to various medical conditions), those randomized to each treatment, events leading to changes in subject number and the final number of subjects contributing to analyses are indicated. All subjects were included in the full analysis set. Subjects with protocol deviations were excluded from the immunogenicity per-protocol set. Exclusive use of the whole third floor at LMC, facilitated transport and LMC being a primary health provider in Lira and neighboring districts favored high subject compliance rates to clinic visits.

**Figure 2:**
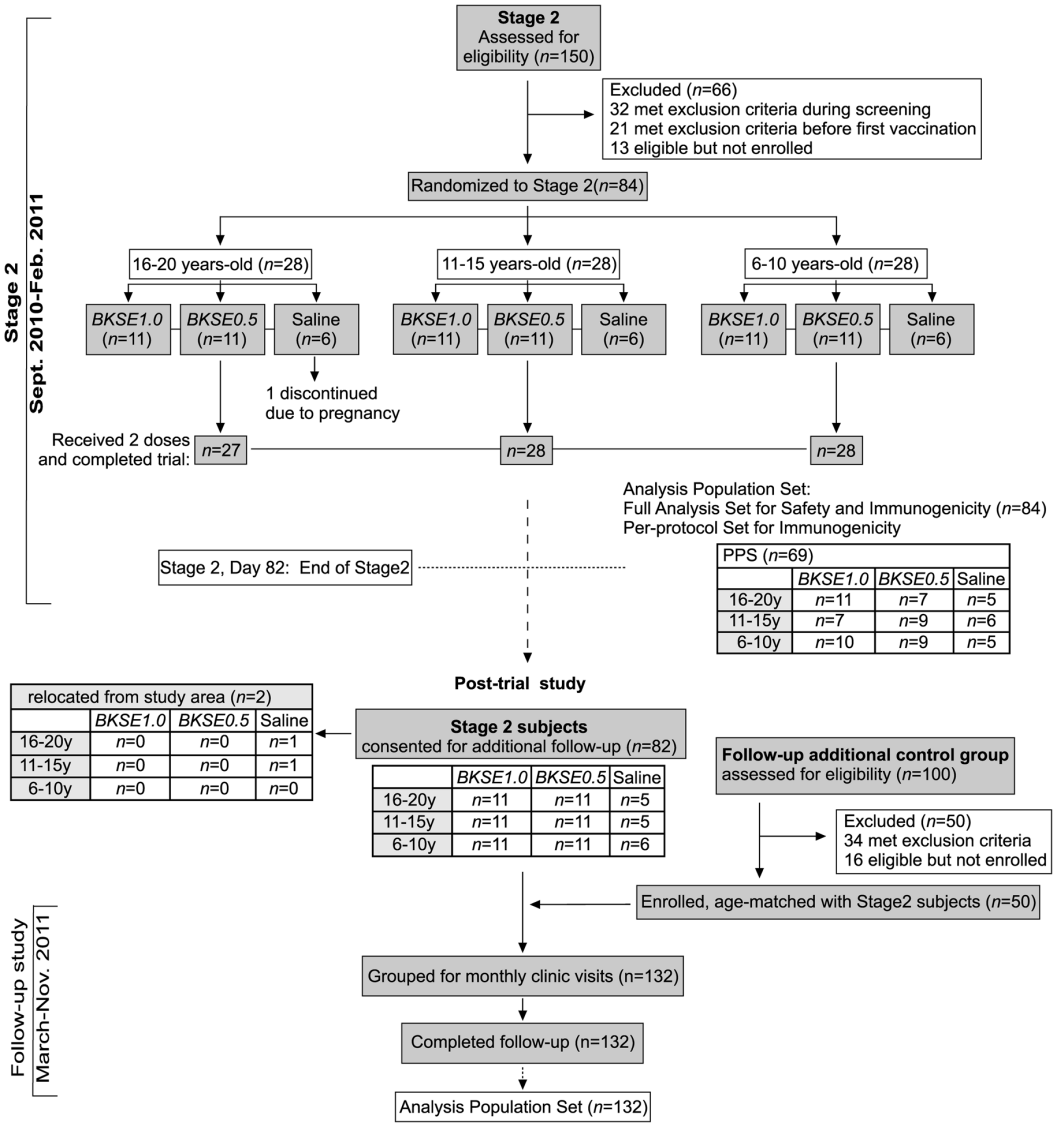
Enrollment and outcomes for Stage2. Trial schedule was similar to Stage1. Ethical clearance for a longitudinal study with additional age-match control group (no intervention) was approved after the trial. In the follow-up study, scheduled four weekly visits continued for up to 1-year post-second vaccination.

The trial was conducted in two stages. Stage1 was in healthy adults aged 21–40 years (n = 56), serologically-negative (seronegative) or positive (seropositive) to anti-SE36 antibody during screening (each cohort had an equal number of male and female subjects) (Fig. 1). Stage2, conducted in healthy children and young adults (n = 84), evaluated either 1.0- or 0.5-mL BK-SE36 (*BKSE1.0* or *BK0.5*, respectively) in 3 age cohorts (16–20-, 11–15-, and 6–10-year-olds) (Fig. 2). Cohort assignment in Stage2 did not take into consideration baseline anti-SE36 antibody concentration or seroconversion to SE36 since our earlier studies showed that most individuals below 20 years were seronegative [14]. For safety assessment and to guide the continuation to Stage2, more subjects received BK-SE36 than saline. A minimum group size of 10 subjects per treatment was chosen to balance the need to detect any possible untoward reactions against the need to limit the number of subjects involved for safety purposes. Enrollment for Stage2 started after approval of Stage1 safety data by an independent Data Safety and Monitoring Board. The decision to proceed to Stage2 was concluded after considering all adverse events (AEs) and serious adverse events (SAEs) observed until 21 days post-second vaccination (42 days of safety data).

Subcutaneous vaccination was in alternate arms 21 days apart between May-June 2010 for Stage1 and Oct-Nov 2010 for Stage2. In phase 1a, 2 vaccinations of *BKSE1.0* could achieve 100% seroconversion [14], thus, 2 vaccinations were adopted for this trial. Subjects were immunized in a staggered fashion.

### Intervention

BK-SE36 is a lyophilized preparation of a recombinant protein based on the N-terminal domain of *P. falciparum* (Honduras-1) serine repeat antigen-5 [14]. The protein, produced in and purified from *Escherichia coli*, was formulated with the adjuvant aluminum hydroxide gel (manufactured by BIKEN) at the GMP facilities of the Kanonji Institute, BIKEN. The white amorphous powder reconstituted shortly before subcutaneous vaccination with 1.3-mL of the supplied water for injection contained approximately 100 µg/mL SE36 protein and 1 mg/mL aluminum (the priming volume was established as 1-mL). *BKSE0.5* contained 50 µg SE36 protein and 0.5 mg aluminum hydroxide gel. The excipients included: dibasic sodium phosphate hydrate, sodium dihydrogen phosphate dihydrate and sodium chloride in addition to aluminum hydroxide gel. The control was 1.0- or 0.5-mL saline solution (Otsuka Pharmaceutical Co., Ltd., Japan).

Vaccines were transported and stored at the study site with devices to monitor fluctuations in temperature. Study numbers were assigned to vaccine vials prior to vaccination. The vaccine and dose assigned during the first vaccination was maintained for the second administration. Used vials were checked for correct allocation at the end of the trial.

### Participants

Prior to the screening visit, public announcements were made at a local radio station and discussion meetings were held in schools (with parents of potential volunteers) following the recommendation of the Community Advisory Board (made up of 5 representatives from the Lira community). At the trial site, initial information/informed consent discussions were done first in groups then individually with a clinical investigator. Written informed consents (ICF) were obtained either in English, Swahili or Luo from the subject/parent/guardian/legal representative according to national laws and regulations: ICF for subjects aged 18–40 years were signed by the subject themselves; ICFs for those between 8–17 years were signed by both the subject and parent/guardian (the child’s assent took precedence over the parent/guardian’s consent); ICFs for 6–7 year-olds were signed by the parent/guardian of the subject. Illiterate subjects/parents/guardians signed using a thumbprint, and an additional signature was obtained from a literate adult witness independent of the study team. All ICFs were signed prior to any trial-related procedure. A photograph was attached to the medical record and healthy volunteers were screened. The screening procedures included taking a full medical history and clinical examination (Text S1 for details on inclusion and exclusion criteria). Volunteers who had clinically significant illness at screening were excluded and treated by the trial clinicians or referred for appropriate treatment/management as per national referral systems. Exclusion criteria included presence (symptoms/signs) of disease that could interfere with interpretation of trial results or compromise the health of the subject; the inability to participate in trial activities; and/or if female, pregnant or breastfeeding. Screening was done within 30 days before the first vaccination. Those who were blood smear positive were treated. Only those who were blood smear negative were vaccinated.

### Randomization and Blinding

Eligible volunteers were given a unique study number and grouped into cohorts.

Randomization to intervention was computer-generated in blocks by an independent statistician (CRO) and emailed to the site. The trial pharmacist assigned sequential codes consecutively following the order in which the subjects signed the informed consent forms. The trial pharmacist did not participate in screening the subjects. Access to the randomization codes was limited to the principal investigator and study physicians (AY, EN). Vaccination records were filed separately from the medical records, kept in a separate locked cabinet and referred to only during vaccination days and when a serious adverse event occurred. Access was limited to study physicians, nurses and pharmacist.

Steps were taken in all trial procedures to prevent undue bias. Vaccine preparation was done in the pharmacy that was separate from the vaccination room. Vaccinations were performed in a closed room by two nurses out of view of anyone other than one physician and a blinded anesthesiologist independent from the trial team (on standby in case of any SAE). Syringe contents were masked using opaque tape to ensure that the subject was blinded. Clinic visits schedules followed the order in which subjects signed the ICF. A 24-hour phone line was maintained by three attending physicians in case of any adverse event. All subjects underwent all protocol assessments during scheduled and unscheduled visits. Measurements for weight and height, blood and urine sampling were done by nurses. Vital signs and physical examination were carried out by physicians. Urinalysis, hematology and blood chemistry tests were performed by laboratory personnel. Microscopists performed finger pricks and blood smears. Both laboratory personnel and microscopists were blinded to both vaccine assignments and clinical evaluations. The laboratory was physically separated from the trial floor. Reporting of subjective AEs (especially local AEs due to vaccination, *e.g.* pain) were based on a scoring table: 1 = mild/easily tolerated, 2 = moderate/interferes with daily activity, 3 = severe/medical intervention required, 4 = potentially life-threatening (Text S1).

### Trial Procedures and Assessments

The primary outcome was vaccine safety. Adverse events were monitored during active and passive visits; including solicited and unsolicited symptoms. All subjects were observed for at least an hour after vaccination. Appropriate medical treatment and equipment were readily available in case of an anaphylactic reaction. Access to health care was facilitated for scheduled visits on Day 0, 7, 14, 21, 22, 28, 35, and 42. The study clinic was also open daily to provide care to the subjects anytime until Day82.

Clinical assessments included monitoring vital signs (blood pressure, pulse rate, axillary temperature), physical examinations (including local site reactions), urinalysis (additionally urine β-hCG was tested in females of reproductive age), hematological and biochemical tests. Biochemical tests evaluated liver function (total protein, albumin, bilirubin, AST, ALT, AL-P, γ-GTP), cholesterol, pancreatic function (serum amylase, glucose), kidney/renal function (uric acid, urea nitrogen, creatinine) and serum electrolyte changes (Na and K). The nature of an AE, onset, outcome, severity and relationship were recorded.

Finger-prick blood was used for malaria smear and filter paper blots. The site had a laboratory technical supervisor and 2 certified microscopists trained at KEMRI affiliated Malaria Diagnostics Centre of Excellence, Kisumu, Kenya. Thick and thin blood smears (TTBS) were examined independently by two microscopists. A third microscopist reconciled any discrepant results. Parasites were counted against 200 white blood cells (WBC) (or per 500, if the count was <10 parasites/200 WBC) and parasite densities (PD) were calculated based on the standard WBC count of 8,000/µL. A thick blood smear was considered negative when the examination of 500 WBCs did not reveal asexual parasites or gametocytes. Thin blood smears were evaluated to determine parasite species. TTBS were read in real-time and subjects were treated with antimalarial medication (artemether-lumefantrine or dihydroartemisinin-piperaquine) if the smear was positive. All positive blood smears were coded as malaria, including asymptomatic cases, and data relating to malaria episodes were reviewed and verified.

The secondary outcome was anti-SE36 antibody titers. Similar to GLP-studies and phase 1a trial [14], immunoglobulin G (IgG) titers pre- and 21-days post-vaccination were used for the assessment of vaccine-induced immunity by enzyme-linked immunosorbent assay (ELISA). As per protocol, screening ELISA was carried out at MBL using a validated standard operating procedure [14] and results obtained were used on site to group subjects to seronegative or seropositive cohorts. ELISA was done using 100 and 200-fold diluted serum samples in 96-well flat-bottom microtiter plates adsorbed with recombinant SE36 (0.3 µg/well in carbonate coating buffer). Anti-SE36 standard serum (positive control) was from a pool of high titer sera from 10 individuals in Uganda; the negative control was a malaria naïve Japanese serum. When the OD value of a 100-fold diluted serum sample was less than the OD of the positive control at 800 dilution, the sample was categorized as seronegative. The ELISA grouping at MBL was confirmed by ELISA measurements at BIKEN Surveillance Center.

Prior to serum shipment, to ensure further unbiased assessment, a separate set of randomization numbers were assigned to serum samples in Kampala (randomization codes were computer generated by the CRO). This secondary randomization corresponded to the order in which antibody titers were determined at BIKEN and ruled out differences in assay conditions on different days, particularly for pre- and post-vaccination samples. Antibody titer measurements used 8 sequential 2-fold serial dilutions of test samples [14]. Samples that were outside the acceptable OD_490_ range were retested at an alternate dilution. The same standard high titer pool of serum was included on each ELISA plate in order to generate a standard curve. A 5000 unit value was assigned as the reciprocal of the dilution giving an OD_490_ = 1 in a standardized assay. Based on the antibody titer of the standard serum, the titer of each serum was calculated with an equilibrium line assay (Bioassay Assist software ver. 2.0.7). Anti-SE36 IgG geometric means (GM), 95% confidence intervals (CI) and mean fold change relative to previous visits (either to Day0 or Day21) were summarized for Stage1 and Stage2.

### Follow-up Study

Post-trial, malaria incidence up to 1-year post-second vaccination was compared between Stage2 subjects and 50 additional age-matched individuals (no intervention). There was a delay in obtaining ethical approval for the start of the follow-up period and thus recruitment of additional control group only took place during Jan-Feb 2011. For Stage2 subjects this meant the absence of active monthly visits during Dec 2010-Feb 2011. However, passive visits still continued and recorded data for malaria events could be extracted retrospectively from clinical records. During Dec 2010-Feb 2011 there were only 2 malaria episodes with ≥5000 parasites/µL from Stage2, both from the placebo group. Throughout the 30-day screening period for the additional control group, there were 9 recorded malaria episodes with ≥5000 parasites/µL. While we cannot strongly argue that it is unlikely that a significant number of malaria cases were missed, based on the health-seeking behavior of the population, it seems unlikely that only placebo/control subjects had passive visits. Notwithstanding, in the comparison of malaria episodes between vaccinees and controls, these and all malaria events prior to Mar 2011 were censored. Only malaria events between 130–365 days post-second vaccination were considered for the ancillary analysis.

Recruitment and screening for eligibility of the age matched cohorts was done in similar manner to Stage2. Eligible volunteers were enrolled in the order that they signed the informed consent form and, whenever possible, age and gender-matched to those who resided in the same locality with clinic visits falling on the same day. All subjects were trained to record their daily axillary temperature, and were given a digital thermometer and monthly diary card. Subjects had 4-weekly scheduled study visits, but were instructed to visit the clinic whenever they were ill. On a scheduled visit, subjects underwent all assessments. At every visit, a questionnaire was completed, medical history was taken; physical examination (including vital signs and axillary temperature), a TTBS and filter blot were completed for each subject. The questionnaire collected information on personal and demographic data, use of bednets and other malaria control activities practiced by the subject, as well as any signs and symptoms of malaria. Three physicians alternated for monthly visits. Data management was done by RIMD.

#### Case definition for malaria episodes

The risk period assessed for the follow-up study was between 130–365 days after the second vaccination and covered 2 observed malaria seasons (May-July and Sept-Oct, data not shown). Parasitemia was grouped into thresholds of >0, >500, >5000 and >10000 parasites/µL. A threshold density of ≥5000 parasites/µL was selected as an appropriate cut-off for a significant parasite count in our analysis. Likewise, previous studies in Uganda demonstrate that threshold parasitemias of ≥5000 parasites/µL show good prediction where malaria is judged to be the sole cause of illness or a substantial contributing factor [18]–[20]. Among malaria symptoms, only those events with fever were included for our ancillary efficacy analysis. A fever due to malaria was defined as axillary temperature of 37.5°C or higher associated with the presence of any *P. falciparum* parasitemia, with no other obvious causes of fever.

### Statistical Analysis

For Stage1 and Stage2, data management and data cleaning processes were done by the CRO. The CRO also reviewed medical records and laboratory results to ensure integrity and regulatory compliance. Data management remained blinded until after data collection had been completed. AEs were classified by preferred term and system organ class according to MedDRA version 12.0. Also before database lock, a statistical analysis plan was made by Quintiles with inputs from the medical adviser (TH) and BIKEN (NS and NMQP). Statistical analysis was provided by the CRO. The total analysis set included all subjects in the trial, with all available data included in the analysis. The safety analysis set consisted of all subjects with at least 1 vaccination. All adverse events were listed. No comparisons were made for subjects who received only one vaccination and those who received both since there were only 2 subjects who did not received a second vaccination.

The immunogenicity analysis was split into a full analysis set (FAS) and a per-protocol set (PPS). Immunogenicity FAS included all subjects whose ELISA results were available. Immunogenicity PPS include all subjects for whom assay results were available and had no major deviations according to the trial protocol (Text S1). Statistical outputs were produced using SAS® Version 9.2. Non-parametric tests (Wilcoxon signed rank test and Wilcoxon rank sum test) compared changes in the antibody titers within (PROC UNIVARIATE) and between treatment groups (PROC NPAR1WAY).

The data from the follow-up study was analysed at RIMD and the Department of Public Health, Osaka City University Faculty of Medicine. Cumulative incidence of first (or only) malaria infection with high parasitemia (≥5000 parasites/µL) and high parasitemia+fever were compared between BK-SE36 and the control group. Consecutive episodes that occurred within 28 days were regarded as a single episode. Survival curves, estimated using GraphPad Prism 5 software and PROC LIFETEST, were drawn following Pocock et al. [21]. Cox regression model (PROC PHREG) was used to evaluate vaccine efficacy against first (or only) malaria episode according to various threshold parasitemias (with or without fever). Multiple episodes were assessed by means of negative binomial regression. Protective efficacy was defined as 1 minus the hazard ratio. Efficacy estimates were also adjusted for age and gender.

All reported *p* values are two-sided. *P* values <0.05 were considered to be significant.

## Results

### Participant Flow

Of 100 adults (21–40 years) who gave consent, only 65 were eligible based on protocol (Fig. 1). The most common reasons for exclusion were medical illness and unavailability for the trial dates. Since pre-existing anti-SE36 antibody titers could affect both safety and immunogenicity of BK-SE36, eligible volunteers were grouped into seronegative (n = 33) or seropositive (n = 32) according to ELISA results. From 65 eligibles, 56 were randomized: 28 seronegative and 28 seropositive subjects (in each cohort, 18 subjects received *BKSE1.0* and 10 subjects received saline). Compliance and retention rates were high, with only 1 subject who did not receive a second dose (placebo) because of a positive urine β-hCG test, but she attended all clinic visits. The subject later reported that she opted to terminate her pregnancy. She was in good health prior to termination of the pregnancy and physical examination was normal at the next clinic visit. Two more subjects had a positive pregnancy test on Day42 (21 days post-second vaccination). One received two doses of BK-SE36 and later chose to have an abortion. She was also in good health before and after termination of her pregnancy. The other subject received two doses of saline. She was treated for malaria on Day43 and reported that she experienced heavy bleeding during the course of her malaria treatment but thought that it was due to delayed menstruation. Physical examination was normal and a repeat urine β-hCG test was negative at the next clinic visit. Investigators suspected this to be a complete abortion secondary to malaria infection.

For Stage2, 150 volunteers gave their consent (Fig. 2). A total of 84 healthy subjects aged 6–20 years were randomized: 28 per age cohort (16–20, 11–15, and 6–10 years old) assigned to either *BKSE1.0* (*n* = 11), *BKSE0.5* (*n* = 11), or saline (*n* = 6). Most subjects (*n* = 83, 99%) received 2 vaccinations except for a 17-year-old in the 1.0 mL placebo group, who was excluded from receiving the second dose because of suspected pregnancy. She completed most visits, physical examination was normal and a repeat urine β-hCG test on Day42 was negative.

In both stages and within each age cohort, treatment groups were generally similar with regards to baseline characteristics (Table 1).

**Table 1:** Baseline characteristics of study participants.

	(Phase 1b Trial)								Follow-up		
Study	Stage 1		Stage 2						(additional control group)		
Age cohort (years)	21–40		16–20		11–15		6–10		16–20	11–15	6–10
Vaccine	BK-SE36	Saline	BK-SE36	Saline	BK-SE36	Saline	BK-SE36	Saline	(None)	(None)	(None)
**Age (y)**	22.2 (1.16)	22.8 (2.49)	17.86 (0.99)	18.33 (1.03)	12.8 (1.38)	12.3 (0.52)	8.2 (1.44)	7.7 (1.86)	17.82 (1.13)	12.94 (1.34)	8.38 (1.5)
**[Min–Max]**	[21]–[24]	[21–32]	[16]–[20]	[17]–[20]	[11]–[15]	[12]–[13]	[6]–[10]	[6]–[10]	[16]–[20]	[11]–[15]	[6]–[10]
**Height (cm)**	168.6 (7.25)	166.4 (6.06)	168.5 (7.43)	165.4 (5.75)	154.7 (7.42)	149.1 (6.45)	132.7 (9.76)	130.6 (11.6)	165.1 (7.32)	156.0 (10.86)	130.8 (9.14)
**[Min–Max]**	[153.7–184.3]	[157.0–183.0]	[157.0–182.2]	[160.0–172.5]	[140.0–169.7]	[140.0–160.0]	[110.5–153.0]	[116.7–147.4]	[150.7–175.0]	[136.5–178.3]	[115.6–148.5]
**Weight (kg)**	61.8 (7.06)	59.98 (7.49)	58.32 (3.96)	55.85 (5.17)	44.06 (8.46)	39.97 (5.40)	28.4 (5.60)	28.25 (6.18)	54.79 (6.25)	43.34 (8.59)	27.96 (6.47)
**[Min–Max]**	[48.2–74.8]	[48.5–75.5]	[50.0–67.0]	[49.0–61.0]	[34.0–63.0]	[33.5–47.5]	[17.0–42.0]	[20.5–38.0]	[42.0–63.0]	[29.5–56.9]	[19.0–41.0]
**BMI**	21.7 (2.11)	21.68 (2.58)	20.65 (1.94)	20.43 (1.91)	18.31 (2.45)	18.02 (2.28)	15.95 (1.4)	16.45 (1.95)	20.20 (1.78)	17.64 (1.88)	16.18 (2.33)
**[Min–Max]**	[17.8–25.7]	[18.1–28.0]	[18.1–25.0]	[18.3–23.8]	[15.4–24.2]	[15.5–20.7]	[13.9–19.8]	[14.3–18.9]	[18.1–23.7]	[14.5–21.8]	[14.2–22.5]
**Parasite positive**	2/36	1/20	2/22	1/6	7/22	3/6	4/22	2/6	4/17	5/17	6/16
**(n/N)**											
**Anti-SE36 titer (GM)**	114.65	101.34	149.7	163.90	79.66	90.57	25.62	51.98	121.21	76.28	14.27
**(95% CI)**	(65.98; 199.24)	(49.71; 206.63)	(75.4; 296.9)	(41.05; 654.5)	(49.62; 127.9)	(18.02; 455.2)	(14.49; 45.31)	(23.26; 116.2)	(55.39; 265.3)	(31.71; 183.5)	(7.71; 26.43)

BMI, body mass index; Table shows the mean characteristics (SD) of the subjects during screening. For *P. falciparum* infection values, n = no. of subjects/N = total subjects enrolled and randomized. Those who were blood smear positive were treated. Only those who were blood smear negative were vaccinated. Anti-SE36 titers are reflected as geometric mean titers (GM: 95% CI, confidence interval).

In the subsequent follow-up study (post-trial), 82 of 84 Stage2 subjects consented to participate (66 BK-SE36 and 16 saline vaccinees, respectively). Two subjects in the placebo group relocated from the study area. To increase the statistical power of the study, the number of controls was increased. A total of 50 age-matched controls were recruited from among 100 volunteers who gave informed consent (Fig. 2). Controls were healthy subjects (with no intervention) who passed the screening test based on the Stage2 inclusion and exclusion criteria, and whenever possible age and gender-matched to those who resided in the same locality and had clinic visits scheduled on the same day. The characteristics of the additional control group at screening were similar to that of the Stage2 subjects (Table 1).

### Vaccine Safety

Local AEs were reported for nearly all of the subjects who received BK-SE36. In both Stage1 (Table 2) and Stage2 (Table 3), the majority of local AEs were induration (Stage1: *n* = 33, 92%; Stage2: *n* = 63, 96%), tenderness (Stage1: *n* = 23, 64%; Stage2: *n* = 34, 51%) and pain (Stage1: *n* = 14, 39%; Stage2: *n* = 17, 26%). The symptom severity was mostly mild to moderate, although, a seropositive male in *BKSE1.0* cohort experienced severe pain/tenderness after the first vaccination (Table S1). In Stage1, the mean and median area of skin affected and duration of induration/nodule formation were similar between cohorts after the first and second administrations (data not shown). There was no association between pre-existing anti-SE36 antibody titers and the number/severity/duration of local AEs (Tables 2, S1, S2). In Stage2, there was no dose-related trend in the number/severity of local AEs (Tables 3, S1, S2), although the mean and median area of skin affected by induration/nodule formation was higher in those that received *BKSE1.0* compared to *BKSE0.5*. Other local AEs were edema/swelling, erythema/redness, hyperpigmentation and hyperemia. Reactogenicity profile did not cause any study withdrawals and the two subjects who had their vaccination discontinued (due to positive urine β-hCG test) were in the placebo group.

**Table 2:** Adverse events (AEs) and serious adverse events (SAEs) in seronegative and seropositive adults.

	*BKSE1.0*		Saline (1.0 mL)	
	Sero−	Sero+	Sero−	Sero+
	[*n* = 18] (%)	[*n* = 18] (%)	[*n* = 10] (%)	[*n* = 10] (%)
***Local AEs***				
Induration	16 (88.9)	17 (94.4)	0	0
Pain	6 (33.3)	8 (44.4)	1 (10)	0
Tenderness	13 (72.2)	10 (55.5)	0	0
Swelling	3 (16.7)	0	0	0
Erythema	0	1 (5.6)	0	0
Redness	1 (5.6)	0	0	0
Hyperpigmentation	0	2 (11.1)	0	0
***Systemic AEs***				
Fever (≥37.5°C)	3 (16.7)	2 (11.1)	2 (20)	1 (10)
Fatigue	1 (5.6)	1 (5.6)	0	0
Blood pressure decrease	0	1 (5.6)	0	0
Blood pressure increase	1 (5.6)	4 (22.2)	5 (50)	1 (10)
Dizziness	1 (5.6)	0	0	0
Headache	1 (5.6)	1 (5.6)	0	0
***Serious AEs***				
Acute gastritis†	1 (5.6)	0	0	0

For local AEs, number and percentages refer to the number of subjects with at least one upper arm experiencing a specified symptom; however, subjects can experience the same symptom in both arms.

† One subject experienced acute gastritis and was hospitalized. On follow-up, the subject later admitted that he took 2 tablets of metronidazole on the day of vaccination and had a remote history of abdominal pain associated with vomiting and diarrhea. The subject recovered without sequelae.

**Table 3:** Adverse events (AEs) and serious adverse events (SAEs) in 6 to 20 years-old.

	BK-SE36		Saline (mL)	
	*BKSE1.0*	*BKSE0.5*	1.0	0.5
	[*n* = 33] (%)	[*n* = 33] (%)	[*n* = 9] (%)	[*n* = 9] (%)
***Local AEs***				
Induration	32 (97.0)	31 (93.9)	0	0
Pain	11 (33.3)	6 (18.2)	0	0
Tenderness	17 (51.5)	17 (51.5)	1 (11.1)	0
Swelling	0	0	0	0
Erythema	4 (12.1)	1 (3.0)	0	0
Redness	0	0	0	0
Hyperpigmentation	1 (3.0)	0	0	0
Hyperemia	1 (3.0)	0	0	0
***Systemic AEs***				
Fever (≥37.5°C)	1 (3.0)	1 (3.0)	0	0
Headache	0	0	1 (11.1)	0
High aspartate aminotransferase (AST)	0	1 (3.0)	0	0
High alanine aminotransferase (ALT)	0	1 (3.0)	0	0
***Serious AEs***	0	0	0	0

For local AEs, number and percentages refer to the number of subjects with at least one upper arm experiencing a specified symptom; however, subjects can experience the same symptom in both arms.

Abnormally high AST and ALT were observed in a 6 year-old subject right after treatment for lower respiratory tract infection.

There was one serious AE (SAE), unlikely related to vaccination, after the second *BKSE1.0* dose in a 21-year old seronegative male (Table 2). The subject was hospitalized due to bacterial gastritis. The severity of the event changed to moderate the next day and resolved without sequelae. There were no other SAEs (Table S3).

Most AEs were common/similar across treatments groups and considered not related to BK-SE36 vaccine (Table S3). Upper respiratory tract infection was the most frequently recorded besides malaria (Table S4). We observed large variations (but no trends) in hematology, blood chemistry, and urinalysis measurements (data not shown). Most out-of-range values were not clinically significant. Clinically significant out-of-range values in 3 subjects were observed right after malaria, pyelonephritis, or urinary tract infection treatments (Table S5). No trends were observed in vital signs and physical examinations over time and between treatment groups or cohorts (data not shown).

### Immunogenicity

Anti-SE36 IgG levels measured at baseline (before administration, Day0) and 21 days post-second vaccination (Day42) are shown in Tables 4 and 5. In Stage1, many adults did not show any obvious increases in their antibody titers 21 days post-second vaccination. Using the full analysis set (FAS), on Day0 mean anti-SE36 antibody levels were low among seronegative subjects, both for those who received *BKSE1.0* and saline (GM for seronegative adults: *BKSE1.0* = 28.13 [95% CI, 19.67–40.22]; placebo = 29.93 [95% CI, 16.19–55.34]). There was, however, a significant change in antibody titers 21 days post-second vaccination (Day42) among subjects who received *BKSE1.0* (GM*_BKSE1.0_* = 45.12 [95% CI, 34.22–59.49], *p* = 0.004). There was also a significant difference in mean changes in antibody titers between *BKSE1.0* and placebo (GM*_saline_* for Day42 = 28.20 [95% CI, 13.41–59.31], *p* = 0.02). Similar results were obtained for the PPS analysis: significant mean changes from Day0 to Day42 in *BKSE1.0* subjects (*p* = 0.04); and between *BKSE1.0* and placebo (*p* = 0.014). No significant changes were observed among seropositive subjects (Table 4).

**Table 4:** Anti-SE36 antibody titers pre-vaccination (baseline) and 21 days post-second vaccination in Stage1.

				ELISA titer		Change from
				Geometric mean [95% CI]		baseline
Seroconversion	Gender	Vaccine	Statistic	Baseline	21 days after 2^nd^ vaccination	*p* value
Negative	Male	*BKSE1.0*	FAS *n*	9	9	FAS:
			GM	35.90 [22.73; 56.70]	59.05 [44.84; 77.77]	*Within BKSE1.0* = 0.004
			PPS *n*	7	7	
			GM	34.26 [18.33; 64.03]	56.02 [40.35; 77.77]	*Between BKSE1.0 vs.*
		Saline	FAS *n*	5	5	Saline = 0.02
			GM	24.35 [13.67; 43.35]	29.79 [9.00; 98.62]	
			PPS *n*	4	4	PPS:
			GM	21.07 [11.41; 38.91]	19.66 [12.28; 31.47]	*Within BKSE1.0* = 0.04
	Female	*BKSE1.0*	FAS *n*	9	9	
			GM	22.04 [12.08; 40.19]	34.48 [21.66; 54.87]	*Between BKSE1.0 vs.*
			PPS *n*	6	6	Saline = 0.014
			GM	29.41 [13.13; 65.85]	44.45 [26.84; 73.61]	
		Saline	FAS/PPS *n*	5	4	
			GM	36.80 [8.75; 154.74]	26.32 [4.46; 155.30]	
Positive	Male	*BKSE1.0*	FAS *n*	9	9	FAS:
			GM	465.40 [207.70;1042.87]	403.87 [205.73; 792.81]	*Within BKSE1.0* = 0.39
			PPS *n*	6	6	
			GM	511.44 [160.85;1626.15]	418.59 [170.79;1025.91]	*Between BKSE1.0 vs.*
		Saline	FAS/PPS *n*	5	5	Saline = 0.47
			GM	237.52 [142.75; 395.19]	333.08 [145.9; 760.40]	
	Female	*BKSE1.0*	FAS *n*	9	9	PPS:
			GM	469.32 [260.81; 844.52]	496.68 [254.68; 968.65]	*Within BKSE1.0* = 0.11
			PPS *n*	7	7	
			GM	521.47 [257.93;1054.29]	460.62 [206.87;1025.62]	*Between BKSE1.0 vs.*
		Saline	FAS/PPS n	5	5	Saline = 0.19
			GM	495.74 [114.01; 2155.55]	340.44 [67.66; 1713.09]	

Statistic is presented either as immunogenicity full analysis set (FAS) or per-protocol set (PPS); n = number of subjects; GM = geometric mean; CI = confidence interval of geometric mean; For additional information on PPS, refer to Text S1. *p* values are based on analysis within treatment group (Signed Rank), and between treatment groups (Wilcoxon Rank Sums).

**Table 5:** Anti-SE36 antibody titers pre-vaccination (baseline) and 21 days post-second vaccination in Stage2.

			ELISA titer		Change from baseline		
			Geometric mean [95% CI]		(within and between treatment groups)		
Age (y)	Vaccine	Statistic	Baseline	21 days after 2^nd^ vaccination	*p* value		
6 to 10	*BKSE1.0*	FAS *n*	11	11	FAS:		
		GM	25.28 [8.99; 71.10]	124.73 [46.78; 332.56]	***BKSE1.0*** = 0.002		
		PPS *n*	10	10	*vs*. Saline = 0.01		
		GM	25.04 [7.85; 79.92]	130.46 [43.64; 389.96]	***BKSE0.5*** = 0.24		
	*BKSE0.5*	FAS *n*	11	11	*vs*. Saline = 0.14		
		GM	25.97 [12.83; 52.56]	48.03 [27.37; 84.29]			
		PPS *n*	9	9	PPS:		
		GM	28.72 [12.66; 65.18]	55.93 [29.30; 106.76]	***BKSE1.0*** = 0.004		
	Saline	FAS *n*	6	6	*vs*. Saline = 0.03		
		GM	51.98 [23.26; 116.20]	38.77 [17.64; 85.24]	***BKSE0.5*** = 0.30		
		PPS *n*	5	5	*vs*. Saline = 0.17		
		GM	57.06 [20.68; 157.40]	43.79 [16.83; 113.90]		*Regardless of age*:	
11 to 15	*BKSE1.0*	FAS *n*	11	11	FAS:	FAS:	
		GM	81.93 [35.34; 189.95]	100.56 [42.02; 240.65]	***BKSE1.0*** = 0.83	**BKSE1.0** = 0.03	*Overall:*
		PPS *n*	7	7	*vs*. Saline = 0.02	*vs*. Saline = 0.007	FAS:
		GM	74.72 [22.07; 252.93]	111.45 [30.64; 405.32]	***BKSE0.5*** = 0.003	**BKSE0.5** = 0.75	**BK-SE36** = 0.17
	*BKSE0.5*	FAS *n*	11	11	*vs*. Saline = 0.81	*vs*. Saline = 0.21	*vs*. Saline = 0.005
		GM	77.46 [42.08; 142.57]	63.48 [36.55; 110.24]	PPS:		
		PPS *n*	9	9	***BKSE1.0*** = 0.81	PPS:	PPS:
		GM	62.82 [32.62; 120.98]	51.71 [29.42; 90.89]	*vs*. Saline = 0.03	**BKSE1.0** = 0.02	**BK-SE36** = 0.05
	Saline	FAS/PPS *n*	6	6	***BKSE0.5*** = 0.01	*vs*. Saline = 0.007	*vs*. Saline = 0.003
		GM	90.57 [18.02; 455.2]	59.51 [13.04; 271.7]	*vs*. Saline = 0.93	**BKSE0.5** = 0.89	
16 to 20	*BKSE1.0*	FAS/PPS *n*	11	11	FAS:	*vs*. Saline = 0.15	
		GM	167.85 [60.13; 468.52]	204.93 [79.03; 531.40]	***BKSE1.0*** = 0.52		
	*BKSE0.5*	FAS *n*	11	11	*vs*. Saline = 0.69		
		GM	133.46 [44.70; 398.49]	156.22 [56.62; 430.99]	***BKSE0.5*** = 0.76		
		PPS *n*	7	7	*vs*. Saline = 0.70		
		GM	66.61 [19.42; 228.41]	88.00 [31.86; 243.07]	PPS:		
	Saline	FAS *n*	6	6	***BKSE1.0*** = 0.52		
		GM	163.90 [41.05; 654.5]	111.90 [36.26; 345.4]	*vs*. Saline = 0.69		
		PPS *n*	5	5	***BKSE0.5*** = 0.58		
		GM	123.70 [25.98; 588.8]	104.90 [24.03; 458.2]	*vs*. Saline = 0.57		

FAS, full analysis set; PPS, per-protocol set; n = number of subjects; GM = geometric mean; CI = confidence interval of geometric mean; For additional information on PPS, refer to Text S1. *p* values are based on analysis within treatment group (Signed Rank), and between treatment groups (Wilcoxon Rank Sums).

In Stage2, greater than 2-fold increases in antibody titers from baseline (Day0) were observed in the youngest cohort (Table 5). Before vaccination, subjects in the youngest cohort had generally low antibody titers (GM_BK*6–10*_ *= *25.62 [95% CI, 14.49–45.31]; GM_saline*6–10*_ = 51.98 [95% CI, 23.26–116.2]). The change in antibody titers from baseline to Day42 was significant for *BKSE1.0* (GM*_BK1.06–10_* for Day 42* = *124.73 [95% CI, 46.78–332.56], *p* = 0.002) but not for *BKSE0.5* subjects (GM*_BK0.56–10_* = 48.03 [95% CI, 27.37–84.29], *p* = 0.24) (Table 5). Likewise, only the change in antibody titers for *BKSE1.0* was significant from the placebo subjects (GM*_saline6–10_* for Day 42 = 38.77 [95% CI, 17.64–85.24], *p* = 0.01). In the placebo group there was <1.5-fold change in anti-SE36 IgG (95% CI, 1.00–1.48). Similar trend was found in the PPS analysis (Table 5).

In the 11 to 15 year-old cohort, mean Day0 anti-SE36 titers were higher compared to levels in the 6 to 10 year-olds (GM_BK*11–15*_ *= *79.66 [95% CI, 49.62–127.9]; GM_saline*11–15*_ = 90.57 [95% CI, 18.02–455.2]). Antibody titers in *BKSE0.5* subjects remained low at Day42 (GM *_BK0.511–15_ = *63.48 [95% CI, 36.55–110.2]). We observed a modest but not significant increase in anti-SE36 titers in *BKSE1.0* subjects (GM *_BK1.011–16_ = *100.56 [95% CI, 42.02–240.65]). However, similar to the 6 to 10 year-old cohort, there was a significant difference in the mean change in antibody titers from Day0 to Day42 among subjects who received *BKSE1.0* compared to those who received saline (GM*_saline11–15_* for Day42 = 59.51 [95% CI, 13.04–271.7], FAS: *p* = 0.02; PPS: *p* = 0.03). A fold change <1 (95% CI, 0.69–1.35) was observed in the placebo group.

Compared to all age cohorts in Stage2, mean Day0 anti-SE36 titers were highest in the oldest cohort, 16 to 20 year-old (GM_BK*16–20*_ *= *149.7 [95% CI, 75.42–297.0], GM_saline*16–20*_ = 163.9 [95% CI, 41.05–654.5]). In this age group there was no significant change in antibody titers (Table 5), although a fold change >1 (95% CI, 1.19–1.79) was observed consistently in those vaccinated with BK-SE36.

Regardless of age, subjects who received *BKSE1.0* had a better immune response than those vaccinated with *BKSE0.5* (FAS: GM_BK1.0_ Day 42 = 136.98 [95% CI, 83.88–223.71], *BKSE1.0* mean change from Day0 to Day42: *p* = 0.03; GM_BK0.5_ Day 42 = 78.10 [95% CI, 51.30–118.88], *BKSE0.5* mean change from Day0 to Day42: *p* = 0.75; PPS: GM_BK1.0_ Day42 = 149.77 [95% CI, 86.21–260.19]; GM_BK0.5_ Day42 = 61.73 [95% CI, 42.98–88.65]). Generally there was an increase in anti-SE36 antibody titers among subjects who received 2 doses of BK-SE36 compared to those who received a placebo (FAS, BK-SE36 *vs*. saline for Day42: GM_BKSE-36_ = 103.43 [95% CI, 74.97–142.69] *vs*. GM_saline_ = 60.44 [95% CI, 33.25–109.89], *p* = 0.005; PPS, BK-SE36 *vs*. saline: GM_BKSE-36_ = 98.59 [95% CI, 69.51–139.84] *vs.* GM_saline_ = 64.55 [95% CI, 34.65–120.26], *p* = 0.003).

At 130 days post-second vaccination (the start of the follow-up study), only the full-dose group (*BKSE1.0*) had high anti-SE36 antibody titers compared to the other groups (Geometric mean for all *BKSE1.0* subjects* = *87.48 [95% CI, 53.82–142.2]; *BKSE0.5* = 52.20 [95% CI, 32.86–82.92]; placebo = 55.92 [95% CI, 27.78–112.6]; additional control group = 56.40 [95% CI, 35.89–88.62]) (data not shown).

### Follow-up Study

**Figure 3:**
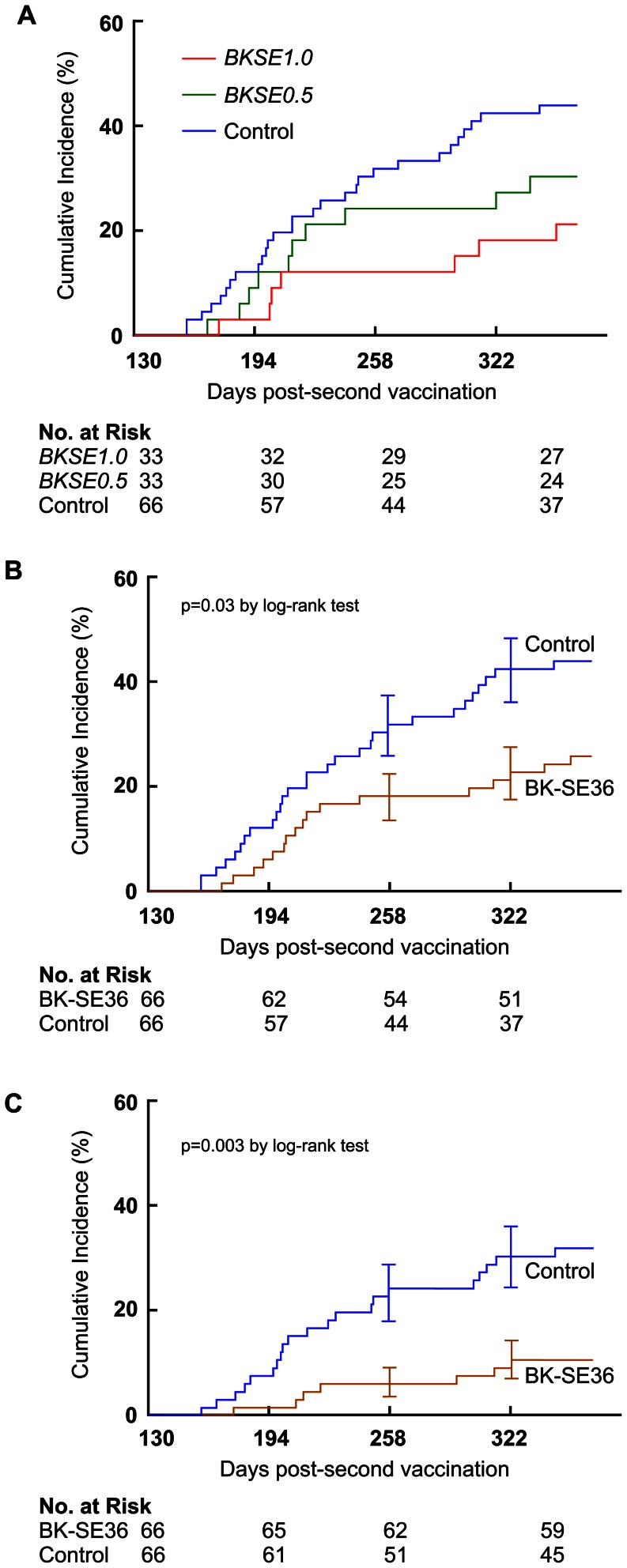
Kaplan-Meier curves of falciparum malaria episodes in 6 to 20-year old, 130–365 days post-second vaccination. Incidence of first (or only) high parasitemia (≥5000 parasites/µL) episodes. The control group consisted of both placebo (vaccinated with saline) and subjects with no intervention. (A) According to vaccine group. Log rank test detected significant difference between control *vs. BKSE1.0* (Chi square 4.92, *p* = 0.03) but not *vs. BKSE0.5* (Chi square 1.59, *p* = 0.21). (B) Pooled analysis of all subjects vaccinated with BK-SE36 (*BKSE1.0*, *BKSE0.5*) compared to control with at least one episode of high parasitemia (Chi square 5.27, *p* = 0.02) or (C) high parasitemia+fever (axillary temperature ≥37.5°C) (Chi square 9.10, *p* = 0.003).

The trial was not designed to measure efficacy but we examined the possibility of a protective effect by BK-SE36. Ancillary analysis shows that between 130–365 days post-second vaccination, fewer subjects in *BKSE1.0* group (7 of 33 [21%]), and in *BKSE0.5* group (10 of 33 [30%]) had a first (or only) episode of malaria infection with parasitemia levels ≥5000 parasites/µL compared to the control subjects (29 of 66 [44%]). Risk ratio for high parasitemia in *BKSE1.0* group was 0.48 (95% CI, 0.24–0.98; *p* = 0.04) and 0.69 (95% CI, 0.38–1.24; *p* = 0.21) in *BKSE0.5* subjects. Kaplan-Meier curves also showed a significant delay for first (or only) high parasitemia (≥5000 parasites/µL) episodes among *BKSE1.0* compared to control subjects (*p* = 0.03 [log-rank test]) (Figure 3A). When BK-SE36 group was pooled, cumulative incidence for the first (or only) malaria infection with high parasitemia was 0.26 (17 of 66), as compared with 0.44 (29 of 66) in the control group (Figure 3B); and 0.11 (7 of 66) *vs*. 0.32 (21 of 66) when considering high parasitemia+fever (Figure 3C). Person-time and hazard ratios are shown in Table S6 for first (or only) malaria episodes. Hazard ratio to first episodes of ≥5000 parasites/µL in BK-SE36 was 0.50 (95% CI, 0.28–0.92, *p* = 0.02) and for first episodes of high parasitemia+fever, 0.28 (95% CI, 0.12–0.66, *p*<0.01) (Table S6). When adjusted for age and gender, hazard ratio was similar to crude estimates: 0.50 [95% CI, 0.27–0.91], *p* = 0.02 for high parasitemia and 0.26 [95% CI, 0.10–0.61], *p*<0.01 for high parasitemia+fever (Table S6). Person-time and hazard ratios for all/multiple malaria episodes are shown in Table S7. Against all/multiple malaria episodes the hazard ratio for high parasitemia was 0.57 (95% CI, 0.33–0.99, *p* = 0.05) [HR_age and gender adj_ = 0.58 (95% CI, 0.34–0.97, *p* = 0.04)] and for high parasitemia+fever, 0.34 (95% CI, 0.15–0.76, *p* = 0.01) [HR_age and gender adj_ = 0.34 (95% CI, 0.15–0.74, *p* = 0.01)] (Table S7). Estimates of efficacy for malaria episodes against other parasite density thresholds (>0, >500, and >10000 parasites/µL) showed significant protective efficacies only for high parasitemia episodes and malaria episodes+fever, suggesting that BK-SE36 may likely have a disease-ameliorating effect rather than preventing infection per se (Tables S6 and S7).

## Discussion

A blood-stage vaccine either alone or as a component of a multi-stage vaccine would be useful to protect against severe or epidemic malaria. This is the first comprehensive safety and immunogenicity study of BK-SE36, a malaria vaccine candidate based on *P. falciparum* SERA5, in a malaria endemic population. Our findings show that two doses of BK-SE36 were safe and had acceptable tolerability in Ugandan adults and older children. Local adverse events were comparable to those observed in Japanese volunteers during phase 1a [14] and with other vaccines of the same adjuvant system [22], [23]. Additional data for safety, tolerability and reactogenicity of BK-SE36 will be collected in larger phase1/2b trials in younger children.

Pre-existing anti-SE36 antibody titers negatively correlated with vaccination-induced antibody response, with greater than 2-fold increases in antibody titers found in the youngest cohort; and higher antibody response in those that received *BKSE1.0* compared to those who received *BKSE0.5.* The seroconversion rate in malaria-exposed Ugandan volunteers differed from the 100% seroconversion observed in malaria naïve Japanese volunteers [14]. In vaccine trials of merozoite surface protein3-long synthetic peptide (MSP3-LSP), high baseline antibody levels in semi-immune adults (due to natural infection/high malaria transmission intensity) were presumed to have overshadowed the inductive capacity of the vaccine [22], [24] but the mechanism for this suppressed immune response remains unclear. We also noted that some seronegative adult Ugandans did not respond to vaccination, confirming the low seroconversion to SERA5 (or SE36) observed in seroepidemiological studies in holoendemic areas [14], [15], [25]. Alternatively, coinfections, prevalent in the study region also merit future evaluation/assessment and how this affects vaccine-immune responses [reviewed in 26,27]. In a recent study of preschool-age children vaccinated with GMZ2 (a malaria vaccine candidate based on MSP3 and glutamate rich protein [GLURP]), antibody responses to GMZ2 was 3.4-fold higher in *Trichuris trichiura*-negative subjects compared to *Trichuris trichiura*-positive subjects [28]. In this trial we did not collect stool samples to analyze helminth infections, although subject medical records of concomitant medications do show some coinfections. Likewise, there are a number of confounding factors that also need to be taken into account (e.g. host genetic variation, host immune status) for the absence of antibody responses in some seronegative malaria-exposed subjects [29]. There was, however, no indication of general immune suppression correlated to race or genetic background since antibody responses were induced in malaria-naïve Japanese adults, the majority of seronegative Ugandan adults and young cohorts. The notably higher frequency of subjects with >2-fold antibody responses in the 6–10 year-old cohort (Table S8) suggests that BK-SE36 might be more immunogenic in younger age group in endemic areas.

In the ancillary analysis, using combined age cohorts, BK-SE36 vaccinees tended to have substantial differences in the time-to-first high parasitemia and all/multiple high parasitemia episodes. This was observed for 2 peak seasons and in a study population reporting 84% usage of bednets and low (2%) coverage of indoor insecticide spraying. During the 130–365 days post-second vaccination, there was a lower-than-expected incidence of high parasitemia episodes in the study population probably because of the close follow-up and improved health care of subjects. Nevertheless, at the end of the follow-up, the incidence of malaria infection with parasitemia levels ≥5000 parasites/µL was significantly lower in BK-SE36 than in the control (Tables S6 and S7).

Our study precludes a robust estimate of vaccine efficacy. Being a phase 1 trial, the study was not designed to detect efficacy. Stage2 had an uneven allocation of subjects with the additional control group enrolled at a later time. The small subject size does not provide sufficient statistical power for assessment of age-dependent protection, thus, comparisons were made using combined age cohorts. Although it can be argued that the levels of antibody response varied with age, the antibody response required for protection against blood-stage parasites remains unknown and overall in Stage2, vaccination did increase antibody titers against the SE36 protein (Table 5). Several aspects of the study made it possible to make an unbiased comparison of malaria episodes between vaccinees and the control group. First, for the entire duration of the follow-up, there were <1% missed visits where antimalarial medication was used prior to on-site evaluation and blood smears, indicating a high degree of study awareness and subject participation. Second, we restricted our ancillary analysis to two quantitative endpoints, parasite density and axillary temperature, which were measured with a high degree of objectivity and reliability. Third, the age and demographic matching of the additional control group left little space for investigators bias. Drug treatment for all episodes of parasitemia encountered could have contributed to bias [30]. Due to ethical considerations in a phase 1 trial, all individuals with asymptomatic infections received antimalarial treatment regardless of parasite count. The degree to which drug treatment affected the interplay of low-density asymptomatic parasitemia, immunity and vaccine-protective response remains to be assessed in future trials.

In conclusion, despite the study limitations discussed above, our preliminary results show promise for BK-SE36 as a malaria vaccine candidate and strongly support the design and conduct of a phase 1/2b double-blind study in children under 5 years.

## Supporting Information

Table S1 **Severity of local site reactions and systemic AEs judged “unlikely” related to BK-SE36.**
(DOC)

Table S2 **Mean area (mm^2^) and duration (days) of local site reactions.**
(DOC)

Table S3 **Overview of adverse events.**
(DOC)

Table S4 **Adverse events with incidence ≥10% from first vaccination to Day82.**
(DOC)

Table S5 **Abnormal clinically significant laboratory values.**
(DOC)

Table S6 **Hazard ratio for first (and only) episodes of malaria in 6 to 20 year-olds.**
(DOC)

Table S7 **Hazard ratio for all or multiple malaria episodes in 6 to 20 year-olds.**
(DOC)

Table S8 **Proportion of subjects with ≥2-fold increase in antibody titers according to age cohorts.**
(DOC)

Text S1 **Supplementary Technical Information.** (**1:** Inclusion and exclusion criteria used for the trial and follow-up study. **2:** Severity grading table for adverse events. **3:** Subject compliance and the per-protocol analysis set for immunogenicity. **4:** Study approvals)
(PDF)

Checklist S1 **CONSORT 2010 checklist of information to include when reporting a randomized trial.**
(DOC)

Protocol S1 **Protocol for the trial.**
(PDF)

Protocol S2 **Protocol for the follow-up study.**
(PDF)
