# Supplementary material for: Phase 1b Randomized Trial and Follow-Up Study in Uganda of the Blood-Stage Malaria Vaccine Candidate BK-SE36
Source: PLoS One. 2013 May 28;8(5):e64073. doi: 10.1371/journal.pone.0064073 (PMC3665850; doi:10.1371/journal.pone.0064073)
Supplement: Table S1 — Severity of local site reactions and systemic AEs judged “unlikely” related to BK-SE36. (DOC) [file pone.0064073.s001.doc]

**Table S1.** Severity of local site reactions and systemic AEs judged “unlikely” related to BK-SE36.

|  | **BK-SE36** | | | **Saline** | | |
| --- | --- | --- | --- | --- | --- | --- |
|  | **Mild** | **Moderate** | **Severe** | **Mild** | **Moderate** | **Severe** |
| ***Stage1*** |  | **(*n*=36)** |  |  | **(*n*=20)** |  |
| Induration | 9 | 24 | 0 | 0 | 0 | 0 |
| Pain | 8 | 5 | 1 | 0 | 1 | 0 |
| Tenderness | 13 | 9 | 1 | 0 | 0 | 0 |
| Swelling | 2 | 1 | 0 | 0 | 0 | 0 |
| Erythema | 1 | 0 | 0 | 0 | 0 | 0 |
| Redness | 1 | 0 | 0 | 0 | 0 | 0 |
| Hyperpigmentation | 2 | 0 | 0 | 0 | 0 | 0 |
| *Systemic AEs* |  |  |  |  |  |  |
| Fever | 5 | 0 | 0 | 3 | 0 | 0 |
| Fatigue | 0 | 2 | 0 | 0 | 0 | 0 |
| Blood pressure decrease | 1 | 0 | 0 | 0 | 0 | 0 |
| Blood pressure increase | 5 | 0 | 0 | 5 | 1 | 0 |
| Dizziness | 1 | 0 | 0 | 0 | 0 | 0 |
| Headache | 1 | 1 | 0 | 0 | 0 | 0 |
| Acute gastritis | 0 | 0 | 1 | 0 | 0 | 0 |
|  |  |  |  |  |  |  |
| ***Stage2*** |  | **(*n*=66)** |  |  | **(*n*=18)** |  |
| Induration | 47 | 16 | 0 | 0 | 0 | 0 |
| Pain | 13 | 4 | 0 | 0 | 0 | 0 |
| Tenderness | 15 | 19 | 0 | 1 | 0 | 0 |
| Swelling | 0 | 0 | 0 | 0 | 0 | 0 |
| Erythema | 5 | 0 | 0 | 0 | 0 | 0 |
| Redness | 0 | 0 | 0 | 0 | 0 | 0 |
| Hyperpigmentation | 1 | 0 | 0 | 0 | 0 | 0 |
| Hyperemia | 0 | 1 | 0 | 0 | 0 | 0 |
| *Systemic AEs* |  |  |  |  |  |  |
| Fever | 2 | 0 | 0 | 0 | 0 | 0 |
| Headache | 0 | 0 | 0 | 0 | 1 | 0 |
| High aspartate  aminotransferase (AST) | 1 | 0 | 0 | 0 | 0 | 0 |
| High alanine  aminotransferase (ALT ) | 1 | 0 | 0 | 0 | 0 | 0 |
