# Supplementary material for: Phase 1b Randomized Trial and Follow-Up Study in Uganda of the Blood-Stage Malaria Vaccine Candidate BK-SE36
Source: PLoS One. 2013 May 28;8(5):e64073. doi: 10.1371/journal.pone.0064073 (PMC3665850; doi:10.1371/journal.pone.0064073)
Supplement: Table S2 — Mean area (mm2) and duration (days) of local site reactions. (DOC) [file pone.0064073.s002.doc]

**Table S2.** Mean area (mm^2^) and duration (days) of local site reactions.

|  | **Stage1** | | **Stage2** | |
| --- | --- | --- | --- | --- |
|  | **Sero–** | **Sero+** | ***BKSE1.0*** | ***BKSE0.5*** |
| **Induration** *n* | **16** | **17** | **32** | **31** |
| Area, mean mm^2^ | 240.1 | 306.2 | 107.8 | 74 |
| (SD) | (119.9) | (186.8) | (76.8) | (68.44) |
| Min/Max | 82/513 | 31/625 | 9/300 | 4/300 |
| Duration, mean days | 22.1 | 25.9 | 26.7 | 23.4 |
| (SD) | (18.23) | (20.51) | (18.37) | (17.69) |
| Min/Max | 7/61 | 7/72 | 7/85 | 7/62 |
| **Pain/Tenderness**  *n* | **13** | **10** | **18** | **19** |
| Duration, mean days | 6.6 | 7.3 | 7.0 | 6.2 |
| (SD) | (1.48) | (0.74) | (0.71) | (1.74) |
| Min/Max | 2/8 | 6/9 | 5/8 | 1/7 |
| **Edema/Swelling** *n* | **3** | **0** | **0** | **0** |
| Duration, mean days | 7·3 | n/a | n/a | n/a |
| (SD) | (0.58) |  |  |  |
| Min/Max | 7/8 |  |  |  |
| **Erythema/Redness** *n* | **1** | **1** | **4** | **1** |
| Duration, mean days | 7 | 6 | 7 | 7 |
| (SD) | n/a | n/a | (0.00) | n/a |
| Min/Max |  |  | 7/7 |  |
| **Other (Hyperpigmentation; Hyperemia)**  *n* | **0** | **2** | **2** | **0** |
| Duration, mean days | n/a | 7·8 | 7·5 | n/a |
| (SD) |  | (0.35) | (0.71) |  |
| Min/Max |  | 8/8 | 7/8 |  |

*n*= no. of events

n/a, not applicable
