# Supplementary material for: Phase 1b Randomized Trial and Follow-Up Study in Uganda of the Blood-Stage Malaria Vaccine Candidate BK-SE36
Source: PLoS One. 2013 May 28;8(5):e64073. doi: 10.1371/journal.pone.0064073 (PMC3665850; doi:10.1371/journal.pone.0064073)
Supplement: Table S3 — Overview of adverse events. (DOC) [file pone.0064073.s003.doc]

**Table S3.** Overview of adverse events.

| ***Description*** | **BK-SE36** | | **Saline** | |
| --- | --- | --- | --- | --- |
| **A. Stage1** | ***n*** | **(%)** | ***n*** | **(%)** |
| ***Number of subjects with:*** |  |  |  |  |
| -At least one AE (from Day 0, first vaccination to Day82, last visit) | 31 | 86.1 | 17 | 85.0 |
| -At least one serious AE | 1 | 2.8 | 0 | 0 |
| -An AE leading to premature discontinuation/ trial withdrawal/ death | 0 | 0 | 0 | 0 |
| ***Severity:*** |  |  |  |  |
| -Mild | 25 | 69.4 | 14 | 70 |
| -Moderate | 21 | 58.3 | 9 | 45 |
| -Severe | 3 | 8.3 | 0 | 0 |
| -Life-threatening | 0 | 0 | 0 | 0 |
| ***Outcome:*** |  |  |  |  |
| -Resolved | 29 | 80.6 | 16 | 80 |
| -Resolved with sequelae | 0 | 0 | 0 | 0 |
| -Death | 0 | 0 | 0 | 0 |
| -Ongoing | 11^a^ | 30.6 | 3 | 15 |
| ***Relationship to investigational product (vaccine):*** |  |  |  |  |
| -Not related | 31 | 86.1 | 17 | 85 |
| -Related | 0 | 0 | 0 | 0 |
| **B. Stage2** |  |  |  |  |
| ***Number of subjects with:*** |  |  |  |  |
| -At least one AE (from Day 0, first vaccination to Day82, last visit) | 44 | 66.7 | 12 | 66.7 |
| -At least one serious AE | 0 | 0 | 0 | 0 |
| -An AE leading to premature discontinuation/ trial withdrawal/ death | 0 | 0 | 0 | 0 |
| ***Severity:*** |  |  |  |  |
| -Mild | 26 | 39.4 | 1 | 5.6 |
| -Moderate | 29 | 43.9 | 11 | 61.1 |
| -Severe | 2 | 3.0 | 0 | 0 |
| -Life-threatening | 0 | 0 | 0 | 0 |
| ***Outcome:*** |  |  |  |  |
| -Resolved | 43 | 65.2 | 12 | 66.7 |
| -Resolved with sequelae | 1^b^ | 1.5 | 0 | 0 |
| -Death | 0 | 0 | 0 | 0 |
| -Ongoing | 2^a^ | 3.0 | 0 | 0 |
| ***Relationship to investigational product (vaccine):*** |  |  |  |  |
| -Not related | 44 | 66.7 | 12 | 66.7 |
| -Related | 0 | 0 | 0 | 0 |

*n*= no. of subjects, % =based on the number of subjects in the safety analyses set. Includes AEs which started after the first vaccination of the investigational product (BK-SE36 or saline) or that already existed before the first vaccination but worsened after vaccination. AEs were considered ‘Related’ if their relationship was indicated on the case report form as ‘Possibly’, ‘Probably’, ‘Definitely’ or ‘Missing’ to the investigational product (vaccine). AEs were ‘Not related’ if indicated as ‘Unlikely’ or ‘Not related’.

^a^Assessed during the last visit date (Day82). Some ongoing AEs were due to indurations that were still palpable at the last visit. There were no reports of sterile abscesses.

^b^Subject was a male adult (17 y-old) in the cohort receiving *BKSE0.5* (50µg dose). AE was fungal infection at anterior chest wall due to *Tinea versicolor*.
