# Supplementary material for: Phase 1b Randomized Trial and Follow-Up Study in Uganda of the Blood-Stage Malaria Vaccine Candidate BK-SE36
Source: PLoS One. 2013 May 28;8(5):e64073. doi: 10.1371/journal.pone.0064073 (PMC3665850; doi:10.1371/journal.pone.0064073)
Supplement: Table S4 — Adverse events with incidence ≥10% from first vaccination to Day82. (DOC) [file pone.0064073.s004.doc]

**Table S4.** Adverse events with incidence ≥10% from first vaccination to Day82.

| **Preferred Term** | **BK-SE36** | | **Placebo** | |
| --- | --- | --- | --- | --- |
|  | ***n*** | **(%)** | ***n*** | **(%)** |
| **Stage1** | **36** |  | **20** |  |
| *Malaria* | 14 | 38.9 | 9 | 45.0 |
| *Upper Respiratory Tract Infection* | 9 | 25.0 | 4 | 20.0 |
| *Body Tinea* | 5 | 13.9 | 1 | 5.0 |
| *Rhinitis* | 4 | 11.1 | 1 | 5.0 |
| *Vulvovaginal candidiasis* | 4 | 11.1 | 1 | 5.0 |
| *Gastroenteritis* | 0 | 0 | 2 | 10.0 |
| **Stage2** | **66** |  | **18** |  |
| *Malaria* | 22 | 33.3 | 5 | 27.8 |
| *Upper Respiratory Tract Infection* | 19 | 28.8 | 3 | 16.7 |
| *Rhinitis* | 8 | 12.1 | 2 | 11.1 |
| *Toothache* | 0 | 0 | 2 | 11.1 |

During the trial, all episodes of positive blood smears captured from screening to Day82 were coded as malaria, including asymptomatic cases.
