# Supplementary material for: Phase 1b Randomized Trial and Follow-Up Study in Uganda of the Blood-Stage Malaria Vaccine Candidate BK-SE36
Source: PLoS One. 2013 May 28;8(5):e64073. doi: 10.1371/journal.pone.0064073 (PMC3665850; doi:10.1371/journal.pone.0064073)
Supplement: Table S5 — Abnormal clinically significant laboratory values. (DOC) [file pone.0064073.s005.doc]

**Table S5.** Abnormal clinically significant laboratory values.

| **Stage** | **Cohort/Treatment** | **Gender/Age** | **Visit Day** | **Variable (Unit)** | **Baseline** | **Abnormal**  **Value** | **Severity** | **Relationship to BK-SE36** |
| --- | --- | --- | --- | --- | --- | --- | --- | --- |
| 1 | Sero-negative/ *BKSE1.0* | Male/22 | 21 days after 2^nd^ vaccination^a^ (Day42) | Alanine aminotransferase ( ALT) (U/L) | 29.4 | 74.9 | Mild | Not Related |
|  |  |  |  | Aspartate aminotransferase (AST) (U/L) | 37.10 | 50.2 | Mild | Not Related |
|  |  |  |  | Total Bilirubin (mg/dL) | 0.80 | 2.4 | Moderate | Not Related |
|  |  |  |  | γ-Glutamyltranspeptidase(GTP) (U/L) | 78.10 | 102 | Mild | Not Related |
|  |  |  |  |  |  |  |  |  |
| 1 | Sero-positive/ *BKSE1.0* | Female/24 | Unscheduled^b^ (Day32) | Glucose (mg/dL) | 102.82 | 150.18 | Mild | Not Related |
|  |  |  |  |  |  |  |  |  |
| 2 | 16 to 20 y-old/ *BKSE0.5* | Male/19 | Unscheduled^c^ (Day21) | Potassium (K) (mmol/L) | 4.54 | 6.36 | Moderate | Not Related |
|  |  |  |  | Platelet Count (10^9^/L) | 116.0 | 91 | Moderate | Not Related |
|  |  |  |  | Urinalysis (blood) |  | Pos^e^ | Mild | Not Related |
|  |  |  |  | Urinalysis (urine color) |  | Amber | Mild | Not Related |
|  |  |  |  | Urinalysis (urine turbidity) |  | Hazy | Mild | Not Related |
|  |  |  | 21 days after 1^st^ vaccination^d^ (Day42) | Potassium (K) (mmol/L) | 4.54 | 5.9 | Moderate | Not Related |

Abnormal values were observed: ^a^ right after malaria treatment, ^b^ when subject was being monitored for pyelonephritis in the left kidney, ^c^ right after malaria and urinary tract infection treatment, and ^d^ when subject was diagnosed with hyperkalemia.

^e^Pos, positive
