# Supplementary material for: Phase 1b Randomized Trial and Follow-Up Study in Uganda of the Blood-Stage Malaria Vaccine Candidate BK-SE36
Source: PLoS One. 2013 May 28;8(5):e64073. doi: 10.1371/journal.pone.0064073 (PMC3665850; doi:10.1371/journal.pone.0064073)
Supplement: Table S6 — Hazard ratio for first (and only) episodes of malaria in 6 to 20 year-olds. (DOC) [file pone.0064073.s006.doc]

**Table S6.** Hazard ratio for first (or only) malaria episodes in 6 to 20 year-olds.

|  |  | | |  | | |  | **Adjusted** |  | | **Adjusted** | |
| --- | --- | --- | --- | --- | --- | --- | --- | --- | --- | --- | --- | --- |
|  | **BK-SE36 Vaccinees** | | | **Control** | | | **Hazard** | **Hazard** | **Protective** | | **Protective** | |
|  | **(*BKSE1.0*, *BKSE0.5*)** | | | **(Saline + no intervention)** | | | **Ratio** | **Ratio^a^** | **Efficacy** | | **Efficacy^a^** | |
|  | **No. of** | **Person-** | **Event** | **No. of** | **Person-** | **Event** |  |  | **%** |  | **%** |  |
|  | **Events** | **Yr** | **Rate** | **Events** | **Yr** | **Rate** | **(CI)** | **(CI)** | **(CI)** | ***p*** | **(CI)** | ***p*** |
| **First (or only) episode, 130-365 days post second-vaccination**  **Parasite density (parasites/µL), any axillary temperature:** | | | | | | | | | | | | |
| **>0** | 45 | 24.8 | 1.81 | 49 | 21.3 | 2.30 | 0.80  (0.53-1.20) | 0.81  (0.54-1.22) | 20  (-20-47) | 0.27 | 19  (-22-46) | 0.32 |
| **>500** | 34 | 29.3 | 1.16 | 36 | 26.3 | 1.37 | 0.86  (0.54-1.37) | 0.89  (0.56-1.42) | 14  (-37-46) | 0.52 | 11  (-42-44) | 0.62 |
| **>5,000** | 17 | 35.4 | 0.48 | 29 | 30.2 | 0.96 | 0.50  (0.28-0.92) | 0.50  (0.27-0.91) | 50  (8-72) | 0.02 | 50  (9-73) | 0.02 |
| **>10,000** | 14 | 36.3 | 0.39 | 24 | 31.8 | 0.76 | 0.51  (0.26-0.99) | 0.52  (0.27-1.01) | 49  (1-74) | 0.05 | 48  (-1-73) | 0.05 |
| **First (or only) episode, 130-365 days post second-vaccination**  **Parasite density (parasites/µL), axillary temperature ≥ 37.5°C:** | | | | | | | | | | | | |
| **>0** | 11 | 40.4 | 0.27 | 25 | 40.4 | 0.62 | 0.38  (0.18-0.76) | 0.34  (0.17-0.70) | 62  (24-82) | 0.01 | 66  (30-83) | <0.01 |
| **>500** | 10 | 37.7 | 0.27 | 25 | 31.9 | 0.78 | 0.34  (0.16-0.71) | 0.31  (0.15-0.65) | 66  (29-84) | <0.01 | 69  (35-85) | <0.01 |
| **>5,000** | 7 | 39.1 | 0.18 | 21 | 32.8 | 0.64 | 0.28  (0.12-0.66) | 0.26  (0.11-0.61) | 72  (34-88) | <0.01 | 74  (39-89) | <0.01 |
| **>10,000** | 6 | 39.5 | 0.15 | 18 | 33.7 | 0.53 | 0.28  (0.11-0.71) | 0.27  (0.11-0.67) | 72  (29-89) | 0.01 | 73  (33-89) | 0.01 |

All recorded episodes were included. In the person-year analysis, parasite density was assumed to decline to zero 3 days after drug treatment. For subjects with no malaria event, the event date was substituted with the censored date, *i.e.* Day365. Event rate or incidence rate = (no. of events)/(person-time at risk). Hazard ratio = (hazard rate of vaccine)/(hazard rate of control). Adjusted hazard ratios were assessed using Cox regression model adjusted for age and gender. Protective efficacy was assessed as 1 minus the hazard ratio. CI, 95% confidence interval. *p* values are two-sided.
