# Supplementary material for: Phase 1b Randomized Trial and Follow-Up Study in Uganda of the Blood-Stage Malaria Vaccine Candidate BK-SE36
Source: PLoS One. 2013 May 28;8(5):e64073. doi: 10.1371/journal.pone.0064073 (PMC3665850; doi:10.1371/journal.pone.0064073)
Supplement: Table S7 — Hazard ratio for all or multiple malaria episodes in 6 to 20 year-olds. (DOC) [file pone.0064073.s007.doc]

**Table S7.** Hazard ratio for all or multiple malaria episodes in 6 to 20 year-olds.

|  |  | | |  | | |  | **Adjusted** |  | | **Adjusted** | |
| --- | --- | --- | --- | --- | --- | --- | --- | --- | --- | --- | --- | --- |
|  | **BK-SE36 Vaccinees** | | | **Control** | | | **Hazard** | **Hazard** | **Protective** | | **Protective** | |
|  | **(*BKSE1.0*, *BKSE0.5*)** | | | **(Saline + no intervention)** | | | **Ratio** | **Ratio** | **Efficacy** | | **Efficacy** | |
|  | **No. of** | **Person-** | **Event** | **No. of** | **Person-** | **Event** |  |  | **%** |  | **%** |  |
|  | **Events** | **Yr** | **Rate** | **Events** | **Yr** | **Rate** | **(CI)** | **(CI)** | **(CI)** | ***p*** | **(CI)** | ***p*** |
| **All episodes, 130-365 days post second-vaccination**  **Parasite density (parasites/µL), any axillary temperature:** | | | | | | | | | | | | |
| **>0** | 75 | 40.8 | 1.84 | 103 | 40.0 | 2.58 | 0.71  (0.52-0.98) | 0.72  (0.53-0.97) | 29  (2-48) | 0.03 | 28  (3-47) | 0.03 |
| **>500** | 46 | 40.8 | 1.13 | 65 | 40.0 | 1.63 | 0.69  (0.46-1.05) | 0.70  (0.48-1.02) | 31  (-5-54) | 0.08 | 30  (-2-52) | 0.07 |
| **>5,000** | 22 | 40.8 | 0.54 | 38 | 40.0 | 0.95 | 0.57  (0.33-0.99) | 0.58  (0.34-0.97) | 43  (1-67) | 0.05 | 42  (3-66) | 0.04 |
| **>10,000** | 17 | 40.8 | 0.42 | 32 | 40.0 | 0.80 | 0.52  (0.28-0.98) | 0.53  (0.29-0.95) | 48  (2-72) | 0.04 | 47  (5-71) | 0.03 |
| **All episodes, 130-365 days post second-vaccination**  **Parasite density (parasites/µL), axillary temperature ≥ 37.5°C:** | | | | | | | | | | | | |
| **>0** | 13 | 40.8 | 0.32 | 32 | 40.0 | 0.80 | 0.40  (0.20-0.78) | 0.40  (0.21-0.77) | 60  (22-80) | 0.01 | 60  (23-79) | 0.01 |
| **>500** | 12 | 40.8 | 0.29 | 32 | 40.0 | 0.80 | 0.37  (0.18-0.73) | 0.37  (0.19-0.72) | 63  (27-82) | <0.01 | 63  (28-81) | <0.01 |
| **>5,000** | 9 | 40.8 | 0.22 | 26 | 40.0 | 0.65 | 0.34  (0.15-0.76) | 0.34  (0.15-0.74) | 66  (24-85) | 0.01 | 66  (26-85) | 0.01 |
| **>10,000** | 7 | 40.8 | 0.17 | 22 | 40.0 | 0.55 | 0.31  (0.13-0.76) | 0.31  (0.13-0.74) | 69  (24-87) | 0.01 | 69  (26-87) | 0.01 |

All recorded episodes were included. Comparison of multiple malaria episodes was done by means of negative binomial regression. In the person-year analysis, parasite density was assumed to decline to zero 3 days after drug treatment. Event rate or incidence rate = no. of events/person-time at risk. Hazard ratio = hazard rate of vaccine/hazard rate of control. Adjusted hazard ratios = adjusted for age and gender. Protective efficacy was assessed as 1 minus the hazard ratio. CI, 95% confidence interval. *p* values are two-sided.
