# Supplementary material for: Phase 1b Randomized Trial and Follow-Up Study in Uganda of the Blood-Stage Malaria Vaccine Candidate BK-SE36
Source: PLoS One. 2013 May 28;8(5):e64073. doi: 10.1371/journal.pone.0064073 (PMC3665850; doi:10.1371/journal.pone.0064073)
Supplement: Table S8 — Proportion of subjects with ≥2-fold increase in antibody titers according to age cohorts. (DOC) [file pone.0064073.s008.doc]

**Table S8.** Proportion of subjects with ≥2-fold increase in antibody titers according to age cohorts.

|  | ***BKSE1.0*** | | ***BKSE0.5*** | | **Saline** | |
| --- | --- | --- | --- | --- | --- | --- |
| **Age (y)** | ***n*** | **Percentage**  **(%)** | ***n*** | **Percentage (%)** | ***n*** | **Percentage (%)** |
| **6 to 10** | 8/11 | 73 | 5/11 | 45 | 0/6 | 0 |
| **11 to 15** | 3/11 | 27 | 0/11 | 0 | 0/6 | 0 |
| **16 to 20** | 3/11 | 27 | 3/11 | 27 | 0/6 | 0 |

*n*= subjects with ≥2- fold increase in antibody titers/total subjects vaccinated
