# Supplementary material for: Phase 1b Randomized Trial and Follow-Up Study in Uganda of the Blood-Stage Malaria Vaccine Candidate BK-SE36
Source: PLoS One. 2013 May 28;8(5):e64073. doi: 10.1371/journal.pone.0064073 (PMC3665850; doi:10.1371/journal.pone.0064073)
Supplement: Text S1 — Supplementary Technical Information. (1: Inclusion and exclusion criteria used for the trial and follow-up study. 2: Severity grading table for adverse events. 3: Subject compliance and the per-protocol analysis set for immunogenicity. 4: Study approvals) (PDF) [file pone.0064073.s009.pdf]

Text S1. Supplementary Technical Information.

Additional information is described: (1) inclusion and exclusion criteria used for the trial and follow-up study; (2) severity grading table for AEs; (3) subject compliance and the per-protocol analysis set for immunogenicity; and (4) study approvals.

1. Inclusion and Exclusion Criteria

A. Inclusion Criteria

Subjects were eligible for inclusion into Stage 1 if they met the following criteria:

- Healthy Ugandan male, female subjects aged 21 to 40 years (age on informed consent).
- No severe malnutrition (defined as an adult whose weight-for-height is below -3 standard deviation [SD] or less than 70% of the median of the National Center for Health Statistics [NCHS]/World Health Organization [WHO] normalized reference values).
- Agreed to comply with matters to be observed during participation in the trial, undergo consultation/examination, and to abide with follow-up procedures as described in the protocol, and report any symptoms.
- Meets the screening assessments:

  Vital signs (temperature, pulse and blood pressure) and physical examination within the normal range.

  Hematology counts were within 25% deviation from the upper and lower limits of the normal range. The differential white blood count (WBC) was not questioned when the total WBC was within the normal range.

  Blood chemistry:

  - aspartate aminotransferase (AST), alanine aminotransferase (ALT), and creatinine levels within the normal range
  - total bilirubin level was within 50% deviation from the upper limit
  - serum electrolytes were within the normal range
  - other blood chemistry parameters were within 25% deviation from the upper and lower limits of the normal range

  Urinalysis was within the normal range.

Subjects were eligible for inclusion into Stage 2 and the follow-up study if they met the following criteria:

- Ugandan male, female subjects aged 6 to 20 years (age on informed consent).
- No severe malnutrition (body mass index was between the 5th percentile to less than the 85th percentile for subjects aged 6 to 19 years and between 18.5 to 25.0 kg/m^2 for subjects aged 20 years).
- Agreed to participate in the trial: for children between 8 to 17 years, the child's assent took precedence over the consent from the parent(s)/guardian(s).
- Agreed to comply with matters to be observed during participation in the trial, undergo consultation/examination, abide by follow-up procedures as described in the protocol, and report any symptoms.
- Meets the screening assessments:

  Vital signs and physical examination within the normal range

Hematology within 25% deviation from the upper and lower limits of the normal range. The differential WBC was not questioned when the WBC count was within the normal range.

Blood chemistry:

- AST, ALT, and creatinine within the normal range
- total bilirubin within 50% deviation from the upper limit
- serum electrolytes within the normal range
- other blood chemistry parameters are within 25% deviation from the upper and lower limits of the normal range

Urinalysis was within the normal range.

B. Exclusion Criteria

Stage1: Subjects were excluded if they met any of the following criteria:

- Fever of 37.5°C or higher on the day of vaccination.
- Clear history of food/drug-related anaphylaxis.
- Female subjects who were pregnant or had a positive urine beta-human chorionic gonadotropin (β-hCG) on the day of, or prior to, vaccination.
- Female subjects currently lactating or breast-feeding.
- Acute or chronic cardiovascular, pulmonary, hepatic, renal, or neurological condition, which in the opinion of the investigator, could have increased the risk of the subject by participating in the trial.
- History of fever within 2 days after preventive injection with other types of vaccine, or those in whom symptoms had suggested systemic allergy.
- History of convulsion.
- Confirmed or suspected immunosuppressive or immunodeficient condition. (HIV testing was not performed. Severe, suspected infectious diseases were ruled out by the investigator during physical examination/consultation, and from results of blood hematology/chemistry tests).
- History or tentative diagnosis of drug allergy.
- History or current drug/alcohol dependency.
- Intake of medication within 1 week before vaccination (except for artemether/lumefantrine and dihydroartemisinin-piperaquine).
- Vaccination of live vaccine within 4 weeks before vaccination, or of inactivated vaccine/toxoid within 1 week of vaccination.
- Participation in another trial within 4 months before vaccination.
- Donation of 200 mL blood within 1 month before vaccination, or more than 400 mL of blood within 3 months before vaccination.
- Others who were not considered to be eligible by the investigator or those, whose medical condition would, in the opinion of the investigator, have made the subject unsuitable for the trial.

Stage2 and the follow-up study: Subjects were excluded if they met any of the following criteria:

- Fever of 37.5°C or higher on the day of vaccination.
- Clear history of food/drug-related anaphylaxis.
- Female subjects who were pregnant or had a positive urine β-hCG on the day of, or prior to, vaccination.
- Female subjects currently lactating or breast-feeding.
- Acute or chronic cardiovascular, pulmonary, hepatic, renal, or neurological condition, which in the opinion of the investigator, could have increased the risk of the subject by participating in the trial.
- History of fever within 2 days after preventive injection with other types of vaccine, or those in whom symptoms had suggested systemic allergy.
- History of convulsion other than febrile convulsions in malaria within 6 months to 1 year.
- Confirmed or suspected immunosuppressive or immunodeficient condition. (HIV testing was not performed. Severe, suspected infectious diseases were ruled out by the investigator during physical examination/consultation, blood hematology/chemistry tests).

  Additionally, subjects verbally informed the investigator if they had been tested positive for HIV. (Information on the child's HIV status was obtained from their parents or guardians.)
- History or tentative diagnosis of drug allergy; especially to common drugs like penicillin and sulphonamides.
- History of chronic alcohol consumption and/or illicit drug use.
- Intake of medication within 1 week before vaccination (except for artemether/lumefantrine and dihydroartemisinin-piperaquine).
- Vaccination of live vaccine within 4 weeks before vaccination, or inactivated vaccine/toxoid within 1 week before vaccination.
- Participation in another trial within 4 months before vaccination.
- Donation of 200 mL blood within 1 month before vaccination, or more than 400 mL of blood within 3 months before vaccination.
- Blood transfusion within 3 months.
- Those, whose medical condition would, in the opinion of the investigator, have made the subject unsuitable for the trial.

2. Severity grading table for AEs in adults and children.

CLINICAL				
**PARAMETER**	**GRADE 1 MILD**	**GRADE 2 MODERATE**	**GRADE 3 SEVERE**	**GRADE 4 POTENTIALLY LIFE-THREATENING**
**ESTIMATING SEVERITY GRADE**				
Clinical adverse event NOT identified elsewhere in this AE grading table	Symptoms causing no or minimal interference with usual social & functional activities. Mild discomfort. No need for medical intervention.	Symptoms causing greater than minimal interference with usual social & functional activities. Moderate discomfort. May require no or minimal medical intervention.	Symptoms causing inability to perform usual social & functional activities. Severe discomfort. Requires medical intervention.	Symptoms causing inability to perform basic self-care functions OR Medical or operative intervention indicated to prevent permanent impairment, persistent disability, or death
**ADMINISTRATION SITE REACTIONS**				
Administration site **pain** (pain without touching) Or **Tenderness** (pain when area is touched)	Pain/tenderness causing no or minimal limitation of use of arm	Pain/tenderness limiting use of arm OR Pain/tenderness causing greater than minimal interference with usual social & functional activities (May require single dose of analgesic)	Pain/tenderness causing inability to perform usual social & functional activities. Requires medical intervention (e.g. repeated doses of analgesic)	Pain/tenderness causing inability to perform basic self-care function OR Hospitalization (other than emergency room visit) indicated for management of pain/tenderness
**Erythema (redness) or skin discoloration**	Light red blush up to 25% of the circumference of the upper arm	Marked redness involving up to 50% of the circumference of the arm	Brick red involving >50% of the circumference of the upper arm	NA
**Edema (swelling)**	Light edema involving up to 25% of the circumference of the upper arm	Marked edema involving up to 50% of the circumference of the arm	Significant edema >50% of the circumference of the upper arm	NA
**Induration/nodule formation**	Hardening under the skin <1.5 cm in diameter	Hardening under the skin 1.5-3.0 cm in diameter	Hardening under the skin >3.0 cm in diameter	NA
**Skin damage (vesicle, ulcer)**	Vesicles or superficial disruption of epithelium < 1 cm	Vesicles or superficial disruption of epithelium 1–2 cm	Full thickness disruption of the epithelium (ulceration) > 2 cm	Necrosis (involving dermis and deeper tissue)
**Formation of crust or scab**	Crust, scab or scar ≤ 2cm	Crust, scab or scar 2–4cm	Crust, scab or scar > 4cm	NA
**Lympadenopathy**	Local, > 1 node/ >2cm	Regional with associated graded pain	Generalized and/or with associated graded pain	Generalized with constitutional symptoms (i.e. fever, headache, etc) requiring hospitalization
**SYSTEMIC REACTOGENICITY** SIGNS/SYMPTOMS				
**Headache**	Minimal headache causing no interference with usual social & functional activities	Moderate headache causing some interference with daily activities. May require single dose of analgesic.	Significant headache causing marked limitation of daily activities (e.g. unable to work). Requires medical intervention (e.g. repeated doses of analgesic)	Symptoms causing inability to perform basic self-care functions OR Hospitalization indicated (other than emergency room visit) OR Headache with significant impairment of alertness or other neurologic function
**Chills**	Symptoms causing no or minimal interference with usual social & functional activities	Symptoms causing greater than minimal interference with usual social & functional activities	Symptoms causing inability to perform usual social & functional activities	NA

PARAMETER	GRADE 1<br>MILD	GRADE 2<br>MODERATE	GRADE 3<br>SEVERE	GRADE 4<br>POTENTIALLY LIFE-THREATENING
**Fatigue Malaise**	Symptoms causing no or minimal interference with usual social & functional activities	Symptoms causing greater than minimal interference with usual social & functional activities	Symptoms causing inability to perform usual social & functional activities	Incapacitating fatigue/ malaise symptoms causing inability to perform basic self-care functions
**Myalgia (non-administration site)**	Muscle pain causing no or minimal interference with usual social & functional activities	Muscle pain causing greater than minimal interference with usual social & functional activities	Muscle pain causing inability to perform usual social & functional activities	Disabling muscle pain causing inability to perform basic self-care functions
**Arthralgia**	Joint pain causing no or minimal interference with usual social & functional activities	Joint pain causing greater than minimal interference with usual social & functional activities	Joint pain causing inability to perform usual social & functional activities	Disabling joint pain causing inability to perform basic self-care functions
**Fever (nonaxillary)**	37·7–38·6°C	38·7– 39·3°C	39·4– 40·5°C	> 40·5°C
**Nausea**	Transient (< 24 hours) or intermittent nausea with no or minimal interference with oral intake	Persistent nausea resulting in decreased oral intake for 24 – 48 hours	Persistent nausea resulting in minimal oral intake for > 48 hours OR Aggressive rehydration indicated (e.g., IV fluids)	Life-threatening consequences (e.g., hypotensive shock)
**Vomiting**	Transient or intermittent vomiting with no or minimal interference with oral intake	Frequent episodes of vomiting with no or mild dehydration	Persistent vomiting resulting in orthostatic hypotension OR Aggressive rehydration indicated (e.g., IV fluids)	Life-threatening consequences (e.g., hypotensive shock)
**Pruritis associated with administration** See also:Pruritis (itching - no skin lesions)	Itching localized to administration site AND Relieved spontaneously or with < 48 hours treatment	Itching beyond the administration site but not generalized OR Itching localized to administration site requiring ≥ 48 hours treatment	Generalized itching causing inability to perform usual social & functional activities	NA
**Pruritis** (itching – no skin lesions)	Itching causing no or minimal interference with usual social & functional activities	Itching causing greater than minimal interference with usual social & functional activities	Itching causing inability to perform usual social & functional activities	NA
**Rash**	Localized maculopapular (non-urticarial) rash at the injection site area causing no interference with daily activities	Diffuse maculopapular (non-urticarial) rash at the injected arm (beyond injection site) causing some limitation of daily activities	Diffuse macular, maculopapular, or morbilliform rash with vesicles or limited number of bullae OR Superficial ulcerations of mucous membrane limited to one site	Extensive or generalized bullous lesions OR Stevens-Johnson syndrome OR Ulceration of mucous membrane involving 2 or more distinct mucosal sites OR Toxic epidermal necrolysis (TEN)
**Allergic reaction**	Localized urticaria at injection site causing no interference with daily activities	Diffuse urticaria at the injected arm (beyond injection site) and causing some limitation of daily activities	Systemic urticaria or angioedema causing marked limitation of daily activities. Requires medical intervention	Acute anaphylaxis OR Life-threatening bronchospasm OR laryngeal edema
**OTHER CLINICAL OBSERVATIONS**				
**Cardiac Arrhythmia**	Asymptomatic with transient dysrhythmia causing no interference with daily activities.<br>No treatment required.	Notable symptoms causing some interference with daily activities.<br>Non-urgent treatment required	Symptomatic and incompletely controlled by medical or invasive treatment.	NA

PARAMETER	GRADE 1<br>MILD	GRADE 2<br>MODERATE	GRADE 3<br>SEVERE	GRADE 4<br>POTENTIALLY LIFE-THREATENING
**Hypotension**	NA	Symptomatic, corrected with oral fluid replacement	Symptomatic, IV fluids indicated	Shock requiring use of vasopressors or mechanical assistance to maintain blood pressure
**Hypertension** – **Adult > 17 years** (with repeat testing at same visit)	> 140–159 mmHg systolic<br>OR<br>> 90–99 mmHg diastolic	> 160–179 mmHg systolic<br>OR<br>> 100–109 mmHg diastolic	> 180 mmHg systolic<br>OR<br>> 110 mmHg diastolic	Life-threatening consequences (e.g., malignant hypertension) OR Hospitalization indicated (other than emergency room visit)
**Hypertension** – **Pediatric ≤ 17 years** (with repeat testing at same visit)	NA	91st–94th percentile adjusted for age, height, and gender (systolic and/or diastolic)	≥ 95th percentile adjusted for age, height, and gender (systolic and/or diastolic)	Life-threatening consequences (e.g., malignant hypertension) OR Hospitalization indicated (other than emergency room visit)
**Hemorrhage, blood loss**	Asymptomatic and requiring no therapy	Mildly symptomatic	Gross blood loss **AND/OR** 1–2 units transfused	NA
**Constipation**	Minimally symptomatic. No medical intervention required.	Significant abdominal pain with impaction requiring prescription	Requiring disimpaction<br>**AND/OR**<br>Hospital treatment	NA
**Diarrhea**	Transient or intermittent episodes of unformed stools OR Increase of ≤ 3 stools over baseline per 24-hour period	Persistent episodes of unformed to watery stools OR Increase of 4–6 stools over baseline per 24-hour period	Bloody diarrhea OR Increase of ≥ 7 stools per 24-hour period OR IV fluid replacement indicated	Life-threatening consequences (e.g., hypotensive shock)
**Oral Discomfort/Dysphagia**	Mild discomfort, no difficulty swallowing	Difficulty swallowing but able to eat and drink	Unable to swallow solids; Loss of appetite associated with significant weight loss	Life-threatening consequences OR Aggressive intervention indicated [e.g., tube feeding or total parenteral nutrition (TPN)
**Alteration in personality-behavior or in mood** (e.g., agitation, anxiety, depression, mania, psychosis)	Alteration causing no or minimal interference with usual social & functional activities	Alteration causing greater than minimal interference with usual social & functional activities	Alteration causing inability to perform usual social & functional activities	Behavior potentially harmful to self or others (e.g., suicidal and homicidal ideation or attempt, acute psychosis) OR Causing inability to perform basic self-care functions
**Paresthesia (burning, tingling, etc.)**	Minimal discomfort resulting in minimal or no interference with daily activities	Notable symptoms resulting in greater than minimal changes in daily activities	Marked and persistent discomfort resulting in significant incapacity<br>**AND/OR**<br>Narcotic analgesia required for symptomatic improvement	NA
**Infection** (any other than HIV infection)	Localized, no systemic antimicrobial treatment indicated AND Symptoms causing no or minimal interference with usual social & functional activities	Systemic antimicrobial treatment indicated OR Symptoms causing greater than minimal interference with usual social & functional activities	Systemic antimicrobial treatment indicated AND Symptoms causing inability to perform usual social & functional activities OR Operative intervention (other than simple incision and drainage) indicated	Life-threatening consequences (e.g., septic shock)
**Neuromuscular weakness** (including myopathy & neuropathy)	Asymptomatic with decreased strength on exam OR Minimal muscle weakness causing no or minimal interference with usual activities	Muscle weakness causing greater than minimal interference with usual social & functional activities	Muscle weakness causing inability to perform usual social & functional activities	Disabling muscle weakness causing inability to perform basic self-care functions OR Respiratory muscle weakness impairing ventilation

PARAMETER		GRADE 1<br>MILD	GRADE 2<br>MODERATE	GRADE 3<br>SEVERE	GRADE 4<br>POTENTIALLY LIFE-THREATENING
**Dyspnea**	**Adult ≥ 14 years**	Dyspnea on exertion with no or minimal interference with usual social & functional activities	Dyspnea on exertion causing greater than minimal interference with usual social & functional activities	Dyspnea at rest causing inability to perform usual social & functional activities	Respiratory failure with ventilatory support indicated
	**Pediatric < 14 years**	Wheezing OR minimal increase in respiratory rate for age	Nasal flaring OR Intercostal retractions OR Pulse oximetry 90–95%	Dyspnea at rest causing inability to perform usual social & functional activities OR Pulse oximetry< 90%	Respiratory failure with ventilatory support indicated
**Cough**		Transient cough resulting in minimal or no interference with daily activities	Recurrent or persistent cough resulting in greater than minimal interference with daily activities	Uncontrolled cough causing significant interference with daily activities	NA
**Arthritis**		Mild pain with no joint swelling.<br>No interference with daily activities.	Moderate pain with inflammation, erythema, or joint swelling.<br>Some interference with daily activities.	Severe pain with inflammation, erythema, or joint swelling causing significant incapacity.	NA
**Skin** (general)		Localized, asymptomatic.	Diffuse with notable symptoms and/or some interference with daily activities.	Generalized, marked symptoms and/or significantly interference with daily activities.	NA
**HEMATOLOGY:** values in the first column are reference ranges used for Stage1 and Stage2					
Hemoglobin (Hgb)					
	Male<br>*(14 –17·4 g/dl)*<br>*Female*<br>*(12–16 g/dl)*<br>*Children*<br>*(9·5–14·8 g/dl)*	10·0–10·9 g/dl<br>OR<br>Any decrease<br>2·5– 3·4 g/dl	9·0– 9·9 g/dl<br>OR<br>Any decrease<br>3·5–4·4 g/dl	7·0– 8·9 g/dl<br>OR<br>Any decrease<br>≥ 4·5 g/dl	< 7·0 g/dL
Platelets, decreased<br>Stage1: (150–400 x 10^9/l)<br>Stage2:(130–450 x 10^9/l)		100·000 x 10^9–<br>124·999 x 10^9/l	50·000 x 10^9–<br>99·000 x 10^9/l	25·000 x 10^9–<br>49·999 x 10^9/l	< 25·000 x 10^9/l
Platelets, elevated		NA	550-600 x 10^9/l	> 600 x 10^9/l	NA
WBC, decreased<br>Adult (5–10 x 10^9/l)<br>Children (4–13·5 x 10^9/l)		2·0 x 10^9 –<br>2·5 x 10^9/l	1·5 x 10^9 –<br>1·9 x 10^9/l	1·0 x 10^9 –<br>1·4 x 10^9/l	< 1·0 x 10^9/l
**BLOOD CHEMISTRY**					
Albumin, serum, low<br>(3·8– 5·1 g/dl)		3·0 g/dl –< LLN	2.0–2.9 g/dl	< 2·0 g/dl	NA
Alkaline Phosphatase<br>Stage1: Male (80–306 U/l), Female (64–306 U/l)<br>Stage2: Male (up to 306 U/l), Female (up to 64–306 U/l);<br>6–15y (up to 644·0), 15–17y (up to 483·0)		1·25–2·5 x ULN	2·6–5·0 x ULN	5·1–10·0 x ULN	> 10·0 x ULN
ALT (SGPT)<br>Male (up to 42 U/l)<br>Female (up to 32 U/l)		1·25 – 2·5 x ULN	2·6–5·0 x ULN	5·1–10·0 x ULN	> 10·0 x ULN
AST (SGOT)<br>Male (up to 37 U/l)<br>Female (up to 31 U/l)		1·25– 2·5 x ULN	2·6–5·0 x ULN	5·1–10·0 x ULN	> 10·0 x ULN

PARAMETER		GRADE 1 MILD	GRADE 2 MODERATE	GRADE 3 SEVERE	GRADE 4 POTENTIALLY LIFE-THREATENING
Bilirubin (Total)<br>Stage1: (up to 1·1 mg/dl),<br>Stage2: (up to 1·2 mg/dl)		1·1–1·5 x ULN	1·6 – 2·5 x ULN	2·6–5·0 x ULN	> 5·0 x ULN
Cholesterol (fasting) *LMC values: up to 30 y: (up to 180mg/dl); above 30 y: (up to 200 mg/dl)*					
	Adult ≥ 18 years	>180–239 mg/dl	240–300 mg/dl	> 300 mg/dl	NA
	Pediatric < 18 years	170–199 mg/dl	200–300 mg/dl	> 300 mg/dl	NA
Creatinine<br>Male (0·6 - 1·1 mg/dl)<br>Female (0·5 - 0·9 mg/dl)		1·1–1·3 x ULN	1·4–1·8 x ULN	1·9–3·4 x ULN	≥ 3·5 x ULN
Glucose, serum, high LMC values: random blood sugar (70–115 mg/dl); fasting blood sugar (70–100 mg/dl)					
	Nonfasting	116–160 mg/dl	161–250 mg/dl	251–500 mg/dl	> 500 mg/dl
	Fasting	110–125 mg/dl	126–250 mg/dl	251–500 mg/dl	> 500 mg/dl
Glucose, serum, low					
	Adult and Pediatric<br>≥ 1 month	55– 64 mg/dl	40–54 mg/dl	30–39 mg/dl	< 30 mg/dl
Serum amylase<br>(up to 90 U/l)		1·1–1·5 x ULN	1·6–2·0 x ULN	2·1–5·0 x ULN	> 5·0 x ULN
Potassium, serum, high<br>(3·6 –5·5 mmol/l)		5·6–6·0 mmol/l	6·1–6·5 mmol/l	6·6–7·0 mmol/l	> 7·0 mmol/l
Potassium, serum, low<br>(3·6 –5·5 mmol/l)		3·2–3·4 mmol/l	3·0–3·1 mmol/l	≤ 2·9 mmol/l	NA
Sodium, serum, high<br>(135–155 mmol/l)		146–150 mmol/l	151–154 mmol/l	155–159 mmol/l	≥ 160 mmol/l
Sodium, serum, low<br>(135–155 mmol/l)		130–135 mmol/l	125–129 mmol/l	121–124 mmol/l	≤ 120 mmol/l
Uric acid<br>*Male* (3·4–7·0 mg/dl)<br>*Female* (2·4–5·7 mg/dl)		7·5–10·0 mg/dl	10·1–12·0 mg/dl	12·1–15·0 mg/dl	> 15·0 mg/dl
Urea nitrogen (BUN)*<br>Stage1: (10–50 mg/dl),<br>Stage2: Adult (10–50 mg/dl),<br>Children (7–22 mg/dl),		1·25– 2·5x ULN	2·6–5x ULN	5·1–10x ULN	>10x ULN
γ-GTP*<br>Stage1:*Male* (11– 61 U/l),<br>*Female* (9–39 U/l);<br>Stage2: Male (up to 61 U/l),<br>*Female* (up to 39 U/l)		1·25– 2·5x ULN	2·6–5x ULN	5·1–10x ULN	>10x ULN
Total protein, hypoproteinemia**<br>(6·6–8·7 g/dl)		5·5–6·0 g/dl	5·0–5·4 g/dl	<5·0 g/dl	NA

3. Subject compliance and the per-protocol analysis set for immunogenicity

The following deviations were excluded per-protocol analysis for immunogenicity: (a) use of medication within 1 week before vaccination, and (b) blood sampling was not performed within 21±2 days after the first or second vaccination of BK-SE36 or saline. These were considered in the trial protocol as deviations that may affect immunogenicity or the treatment of the subject. Commonly reported medications include antibiotics, antihistamines, non-steroidal anti-inflammatory drug, and contraceptives. Subject compliance to the protocol and the resulting dataset are shown below:

Stage1:

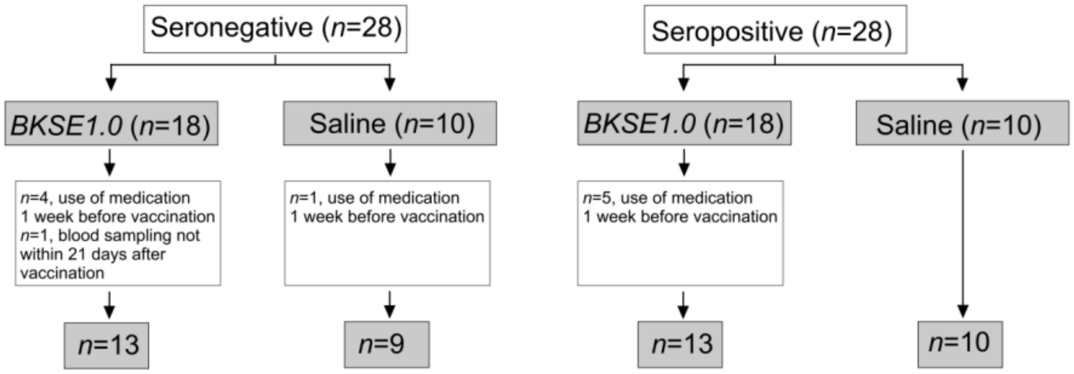

Stage2:

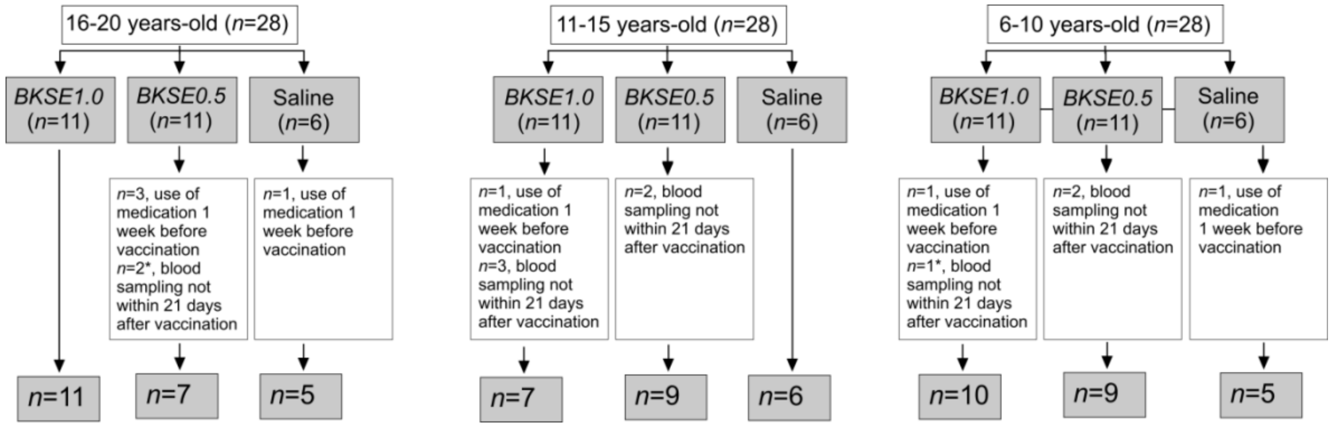

*One subject was excluded for both concomitant medication and blood sampling date.

4. Study approvals

A. UNCST Approvals for Phase1b
B. NDA Approvals and Import Permissions for Phase1b
C. UNCST Approval for the Follow-up Study

A. UNCST Approvals for Phase1b trial

Uganda National Council For Science and Technology

(Established by Act of Parliament of the Republic of Uganda)

UNCST

NDA
date: 08/09/09
reg# 5379
dept: S/K
class. code:
all:
c.c.:1.
2.
3.

Your Ref:..........................

Our Ref: HS 635

Date: 08/09/09

Dr. Thomas Gordon Egwang
Principal Investigator
Med Biotech Laboratories
P O Box 9364
Kampala

Dear Dr. Egwang,

Re: Research Project Approval and Clearance

This is to inform you that on **July 29, 2009** the Uganda National Council for Science and Technology (UNCST) approved the research proposal titled, **"Single Blind, Randomised, Controlled, Phase 1b Trial of the Safety and Immunogenicity of Lyophilized Recombinant Precipitated Tropical Malaria Vaccine (BK-SE 36) in Uganda"** BK-SE36/002/P03; Version 2.0, 03/Aug/2009; ISRCTN No; ISRCTN71619711. The approval is valid until **July 29, 2010**. If it is necessary to continue with the research project beyond the expiry date, a request for continuation should be made in writing, and a progress report should be provided, to the Executive Secretary, UNCST. Please note that UNCST has no objection to **Dr. Adoke Yeka, Dr. Jane Achan, Dr. Edward H. Ntege and Dr. Simon Bagumaho** participating in this study

Any problems of a serious nature related to the execution of your research project should be brought to the attention of the UNCST, and any changes to the research protocol should not be implemented without UNCST's approval except when necessary to eliminate apparent immediate hazards to the research participant(s).

This letter also serves as proof of UNCST approval and as a reminder for you to submit to UNCST timely progress reports and a final report on completion of the research project.

Please, note that you are required to obtain a certificate for the importation and/or use of the study drug(s) from the National Drug Authority.

Yours sincerely,

Jane Nabbuto
for: Executive Secretary
UGANDA NATIONAL COUNCIL FOR SCIENCE AND TECHNOLOGY

cc Executive Director, National Drug Authority

Uganda National Council For Science and Technology

(Established by Act of Parliament of the Republic of Uganda)

UNCST

Your Ref:..........................

Our Ref:................HS 635 **Date:**..11/03/2010........

Dr. Thomas Gordon Egwang
Principal Investigator
Med Biotech Laboratories
P.O Box 9364
Kampala

Dear Dr. Egwang,

Re: Clarification Memo

Single Blind, Randomized, Controlled, Phase 1b Trial of the Safety and Immunogenicity of Lyophilized Recombinant Precipitated Tropical Malaria Vaccine (BK-SE 36) In Uganda, BK-SE36/002/P03: Version 2.0, Dated 03 August 2009; ISRCTN No. ISRCTN71619711.

In our letter dated 08 September 2009 we referred to the date of approval of the above protocol as 29 July 2009 which is the date that the protocol was actually submitted to Uganda National Council for Science and technology for registration.

We wish to clarify that the correct date of approval was 31 August 2009 and not 29 July 2009. Please note the corrected date; and note that the approval is valid until August 31, 2010.

We apologize for that error.

Yours sincerely,

Leah NaweguIo
for: Executive Secretary
UGANDA NATIONAL COUNCIL FOR SCIENCE AND TECHNOLOGY

cc Executive Director, National Drug Authority

LOCATION/CORRESPONDENCE

Plot 3/5/7, Nasser Road
P.O. Box 6884
KAMPALA, UGANDA.

COMMUNICATION

TEL: (256) 414-250499, (256) 414 705500
FAX: (256) 414-234579
EMAIL: uncst@starcom.co.ug
WEBSITE: http://www.uncst.go.ug

Uganda National Council For Science and Technology

(Established by Act of Parliament of the Republic of Uganda)

UNCST

Your ref: .... HS 635

Date : 13/08/2010

Our ref: ....

Dr. Thomas Egwang
Med Biotech Laboratories
P.O Box 9364
Kampala

Dear Dr. Egwang,

RE: RESEARCH PROJECT RENEWAL

This is to inform you that on **August 12, 2010** the Uganda National Council for Science and Technology (UNCST) approved the research proposal entitled, **"Single Blind, Randomized, Controlled, Phase 1b Trial of the Safety And Immunogenicity Of Lyophilized Recombinant Precipitated Tropical Malaria Vaccine (BK-SE36), in Uganda, BK-SE36/002/P05: Version 4.0, Dated June 24, 2010; ISRCTN No. ISRCTN71619711"**. The approval is valid until **August 31, 2011.** If it is necessary to continue with the research project beyond the expiry date, a request for continuation should be made in writing, and a progress report should be provided, to the Executive Secretary, UNCST.

Any problems of a serious nature related to the execution of your research project should be brought to the attention of the UNCST, and any changes to the research protocol should not be implemented without UNCST's approval except when necessary to eliminate apparent immediate hazards to the research participant(s).

This letter also serves as proof of UNCST approval and as a reminder for you to submit to UNCST timely progress reports and a final report on completion of the research project.

Please, note that you are required to obtain a certificate for the importation and/or use of the study drug(s) from the National Drug Authority.

Yours sincerely,

Leah Nawegulo
for: Executive Secretary
UGANDA NATIONAL COUNCIL FOR SCIENCE AND TECHNOLOGY

cc Executive Director, National Drug Authority, Kampala

LOCATION / CORRESPONDENCE
Plot 3/5/7, Nasser Road
P.O. Box 6884
Kampala, Uganda

COMMUNICATION
TEL: (256) 414-250 499, (256) 414 705 500
FAX: (256)414-234 579
EMAIL: uncst@starcom.co.ug
WEBSITE: http://www.uncst.go.ug

B. NDA Approvals and Import Permissions for Phase1b trial

NATIONAL DRUG AUTHORITY

Plot 46-48 Lumumba Avenue
P.O. Box 23096
Fax: 041 4 255758
Tel: 0414 255665 / 347391 / 347392
Kampala, Uganda (E.A)
Email: nda@nda.or.ug
Website:www.nda.or.ug

Your Ref: ....

Our Ref: 633/ESR/NDA/DID-11/09

Date: 18. 11. 2009

The Principle Investigator
BK-SE36/002 Study
Med Biotech Laboratories, Plot 3438 Muyenga, Tank Hill
By-pass, P.O. Box 9364, Kampala, Uganda
Tel +254-712-504010
Email: doublestrand2000@yahoo.com

Dear Professor Thomas Egwang,

AUTHORIZATION FOR THE IMPORTATION OF UNREGISTERED PRODUCT IN TERMS OF SECTION 40 OF THE NATIONAL DRUG POLICY AND AUTHORITY ACT (CAP.206).

PRODUCTS: . Malaria vaccine (BK-SE36)

Your application dated 27 Aug 2009 refers

1. RESOLUTION AND APPROVAL

It has been resolved by the National Drug Authority that; the clinical trial application according to the following Protocol be approved:-

A Single blind, randomized, controlled, phase 1b trial of the safety and immunogenicity of lyophilized recombinant precipitated tropical malaria vaccine (BK-SE36) in Uganda
Protocol No: BK-SE36/002/P03 Version No:2.0 Dated August 3 2009

2. AUTHORISATION

Authorisation is hereby granted for the importation and administration of a sufficient quantity, for the duration of the trial, of the investigational product (200 vials of the white lyophilised powder with diluent).

3. THIS AUTHORISATION IS SUBJECT TO THE FOLLOWING PROVISIONS

(a) The NDA shall be informed immediately of any toxic effects or death, which may occur during the Clinical Trial and of any data received which, might cast doubt on the validity of the continuation of the Clinical Trial.

(b) The NDA shall be notified of any decision to discontinue the Clinical Trial. The reason for such cancellation shall be stated.

(c) The Clinical Trial shall be conducted in accordance with the NDA approved Protocol Any Amendment(s) to the Protocol shall first be submitted to the NDA for approval. All Clinical Trials be conducted in accordance with ICH-GCP Guidelines

(d) The medicine shall be administered by or under the direction of the authorised PI. In the case where the PI permits another Medical Practitioner to administer a medicine, which is exempted from the registration for the purpose of the Trial, the PI shall remain responsible for any eventuality arising from such usage.

(e) In the event of the authorised PI ceasing to participate in the Clinical Trial, the NDA shall be informed and the reason for such ceasation shall be given.

This approval is valid up to 19/11/2010 and if you have to continue with the study, a request for continuation should be made in writing to the Executive Secretary, National Drug Authority

Apollo Muhairwe
EXECUTIVE SECRETARY/REGISTRAR

Copy to: Head Inspectorate
Drug Information Department
Drug Assessment & Registration

NATIONAL DRUG AUTHORITY

Plot 46-48 Lumumba Avenue
P.O. Box 23096
Fax: 041 4 255758
Tel: 0414 255665 / 347391 / 347392
Kampala, Uganda (E.A)
Email: ndaug@nda.or.ug
Website:www.nda.or.ug

Your Ref: .... Date: 16/08/2010

Our Ref: 135/ESR/NDA/DID-08/2010

Principal Investigator
BIKEN malaria vaccine trial in Uganda
P.O Box 9364 Kampala, Uganda

Dear Dr. Thomas Egwang

Re: Re: Approval of the amended protocol entitled: *Single Blind, Randomized, Controlled, Phase Ib Trial of the Safety and Immunogenicity of Lyophilized Recombinant Precipitated Tropical Malaria Vaccine (BK- SE36), in Uganda* BK-SE36/002/P05; version 4.0; June 24, 2010.

Your application dated July 7, 2010 refers.
This is to inform you that NDA has reviewed your application and we have noted that several changes have been made to the previous protocol *version* BK-SE36/002/P04; version 3.0; January 25, 2010.

We recognize that there are some substantive amendments particularly changes in the randomization of subjects. In the amended version we recognize that participants may be randomized after an additional visit 1.

We also recognize the go opinion of the Data and Safety Monitoring Board (DSMB) in their meeting dated 23rd July 2010 for continuation to stage 2 of this trial.

Approval is hereby granted for you to continue with the study as detailed in the protocol version 4.0: "A single blind randomized, controlled, phase Ib trial of the safety and immunogenicity of lyophilized recombinant precipitated tropical malaria vaccine (BK-SE36) in Uganda".

This approval is valid up to 16/08/2011 and if you have to continue with the study, a request for continuation shall be made in writing to the Executive Secretary, National Drug Authority.

Wishing you continued cooperation in this matter.

Apollo Muhairwe
EXECUTIVE SECRETARY/REGISTRAR

82050

DRUG

NATIONAL AUTHORITY

The National Drug Policy and Authority
(Issue of Licences) Regulations 1995

Import Licence

(issued under s44 and s45 of the Act)

This is to certify that:

the Applicant named **Med Biotech Laboratories**

of Address **P.O. Box 9364, Kampala**

having TIN

is hereby granted a permit to *import into Uganda, pursuant to the provisions of section 44 and 45 of the Act, the following classified drugs of classes A,B,C and pharmaceutical raw materials subject to the provisions of sections 44 and 45 of the Act and the conditions specified in this licence.

NATIONAL DRUG AUTHORITY
12 JAN 2010
PLOT 46 - 48 LUMUMBA AVENUE
P. O. BOX 23096 KAMPALA

Conditions

Medical supplies as on Proforma invoice No BK-SE36-091216-1 dated 16/12/2009.

The above items are from Kanonji Institute- Japan

Proper records must be maintained.

This licence is valid only for importation through authorised Customs entry points. Each consignment to be imported must be verified in advance by the National Drug Authority.

Permit. No **012/P/2010** Date **12/01/2010**

NATIONAL DRUG AUTHORITY
12 JAN 2010
PLOT 46-48 LUMUMBA AVENUE
P. O. BOX 23096 KAMPALA

Fee Paid Ushs **100,000/-** This permit expires on the **12/03/2010**

GENUINE GENUINE

PP

SECRETARY/REGISTRAR, NATIONAL DRUG AUTHOR

89887

DRUG

NATIONAL AUTHORITY

The National Drug Policy and Authority (Issue of Licences) Regulations 1995

Import Licence

(issued under s44 and s45 of the Act)

This is to certify that:

the Applicant named **Med Biotech Laboratories**

of Address **P.O. Box 9364, Kampala Uganda**

having TIN
is hereby granted a permit to *import into Uganda, pursuant to the provisions of section 44 and 45 of the Act, the following classified drugs of classes A,B,C and pharmaceutical raw materials subject to the provisions of sections 44 and 45 of the Act and the conditions specified in this licence.

Conditions

Medical supplies as on Proforma invoice No. BK-SE36-100730-1 dated 30/07/2010
The above items are from Kanonji Institute - Japan
Proper records must be maintained.

This licence is valid only for importation through authorised Customs entry points. Each consignment to be imported must be verified in advance by the National Drug Authority.

Permit. No **258/P/2010** Date **23/08/2010**

Fee Paid Ushs **100,000/-** This permit expires on the **23/10/2010**

NATIONAL DRUG AUTHORITY
23 AUG 2010
PLOT 46 - 48 LUMUMBA AVENUE
P. O. BOX 23096, KAMPALA

EXECUTIVE SECRETARY/REGISTRAR,
NATIONAL DRUG AUTHORITY

GENUINE NDA GENUINE

C. UNCST Approval for the Follow-up Study

Uganda National Council For Science and Technology

(Established by Act of Parliament of the Republic of Uganda)

UNCST

Your ref: ....

Date : ....

Our ref: ....
HS 866

06/01/2011

Dr. Thomas Egwang
Director
Med Biotec Laboratories
Kampala

Dear Dr. Egwang,

RE: RESEARCH PROJECT, "LONGITUDINAL STUDY OF THE IMMUNOGENICITY OF BK-SE36 CANDIDATE MALARIA VACCINE IN CHILDREN AND YOUNG ADULTS THAT PARTICIPATED IN THE BK-SE36 MALARIA VACCINE TRIAL IN UGANDA"

This is to inform you that on **November 23, 2010** the Uganda National Council for Science and Technology (UNCST) reviewed and approved the above observational follow up study. Approval will expire on **November 23, 2011.** The approval granted covers the following documents;

1. Study protocol
2. Informed consent form
3. Data collection questionnaire
4. Shipping procedure for serum samples from Uganda and Japan
5. Serum storage and accountability

If it is necessary to continue with the research beyond the expiry date, a request for continuation should be made in writing to the Executive Secretary, UNCST.

Any problems of a serious nature related to the execution of your research project should be brought to the attention of the UNCST, and any changes to the research protocol should not be implemented without UNCST's approval except when necessary to eliminate apparent immediate hazards to the research participant(s).

This letter also serves as proof of UNCST approval and as a reminder for you to submit to UNCST timely progress reports and a final report on completion of the research project.

Yours sincerely,

Jane Nabbuto
for: Executive Secretary
UGANDA NATIONAL COUNCIL FOR SCIENCE AND TECHNOLOGY

LOCATION / CORRESPONDENCE
Plot 3/5/7, Nasser Road
P.O. Box 6884
Kampala, Uganda

COMMUNICATION
TEL: (256) 414-250 499, (256) 414 705 500
FAX: (256)414-234 579
EMAIL: uncst@starcom.co.ug
WEBSITE: http://www.uncst.go.ug
