# Supplementary material for: Phase 1b Randomized Trial and Follow-Up Study in Uganda of the Blood-Stage Malaria Vaccine Candidate BK-SE36
Source: PLoS One. 2013 May 28;8(5):e64073. doi: 10.1371/journal.pone.0064073 (PMC3665850; doi:10.1371/journal.pone.0064073)
Supplement: Protocol S1 — Protocol for the trial. (PDF) [file pone.0064073.s011.pdf]

Protocol amendments: (June 24, 2010) [BK-SE36/002/P05, Version 4.0]

Changes incorporated:

Page	Field	Before	After	Explanations
4	MEDICAL ADVISERS, RESEARCH COORDINATOR and JAPAN RESEARCH LABORATORY (2)	Ken Ishii, M.D., PhD.<br>Associate Professor<br>Tel +81(6)-6879-8280<br>*Fax* +81(6)-6879-8281<br>*email*: kenishii@biken.osaka-u.ac.jp	Ken Ishii, M.D., PhD.<br>Principal Investigator,<br>Laboratory of Vaccine Science<br>WPI, IFReC, Osaka University<br>Tel +81(6)-6879-8303<br>*Fax* +81(72)-641-8043<br>*email*: kenishii@biken.osaka-u.ac.jp	For updating
12, 39	Rationale/<br>Trial design	An overall view of trial stages from regulatory approval to post trial follow-up is presented in the Appendix, Section 18.1, p. 89.	An overall view of trial stages from regulatory approval to post trial follow-up is presented in the Appendix, Section 18.2, p. 91.	For updating
14, 43	[Inclusion criteria 2]:<br>Stage 2, 6 to 20 y old		Healthy subjects are specified, and the inclusion criteria of malnutrition index and laboratory test values were included to reduce individual variation.	For clarification
14, 43-44	[Inclusion criteria 2]:<br>Stage 2, 6 to 20 y old	<2.1> Healthy volunteers, irrespective of gender, aged 6 to 20 years (age on informed consent)<br><2.2> Those who do not suffer from severe malnutrition (defined as a child or adult whose weight-for-height is below -3 standard deviation or less than 70% of the median of the NCHS/WHO normalized reference values);	<2.1> Volunteers, irrespective of gender, aged 6 to 20 years (age on informed consent)<br><2.2> Those who do not suffer from severe malnutrition, whose BMI is between 5th percentile to less than the 85th percentile for 6-19 y old; and between 18.5-25.0 for 20 y old;	<2.1> To be more clearer for interpretation, the word "healthy" is removed as it is otherwise detailed in other criteria. This item pertains to age inclusion alone<br><br><2.2> For this criteria, the clinical investigators deems it much more applicable to use BMI as health index rather than weight-for-height
15, 44	Exclusion criteria for both Stage 1 and 2	<7> Persons with a history of convulsion;	<7> Persons with a history of convulsion other than febrile convulsions in malaria in the past 6 months to 1 year	Investigators raised the concern about convulsions due to malaria which are very common in Ugandan children. With due consideration to Japanese Vaccination Guideline, the criteria was modified to rule out recent convulsions due to malaria.
15-16, 45	Exclusion criteria for both Stage 1 and 2	<8>... Additional oral confirmation: Subject informed the investigator that he/she has been tested positive for HIV/AIDS.	<8>.... Additional oral confirmation: Subject informed the investigator that he/she has been tested positive for HIV/AIDS. (Information on the child's HIV status could be obtained from their parents or guardians.)	For children, additional information on child's HIV status will be solicited from parents or guardians.
16, 45	Exclusion criteria for both Stage 1 and 2	<9> Persons with a history or tentative diagnosis of drug allergy	<9> Persons with a history or tentative diagnosis of drug allergy; especially to common drugs like penicillin, sulphonamides, etc.	Clinical investigators basis was the Stage 1 experience--since the trial is not conducting tests for drug allergy; and some previous volunteers from the community have known allergy to aspirin, chloroquine, etc.
16, 45	Exclusion criteria for both Stage 1 and 2	<10> Persons with a history of or present drug/ alcohol dependency;	<10> Persons with (history of) chronic alcohol consumption and/or illicit drug use;	Same rationale as above, since the trial is not conducting any tests for

<table>
<tr><th>Page</th><th>Field</th><th>Before</th><th>After</th><th>Explanations</th></tr>
<tr><td></td><td></td><td></td><td></td><td>drug/alcohol dependency.</td></tr>
<tr><td>16, 45</td><td>Exclusion criteria for both Stage 1 and 2</td><td><13> Persons who participated in another trial within 4 months before administration of this test vaccine</td><td><13> Persons who participated in another trial within 4 months before administration of this test vaccine; or simultaneous participation in any other clinical trial;</td><td>Lira community is likely to welcome other clinical trials soon; this sentence is inserted to be able to rule out other drug/ vaccine influences which may affect safety of volunteers.</td></tr>
<tr><td>16, 45</td><td>Exclusion criteria for both Stage 1 and 2</td><td></td><td><15> Persons who have recently undergone blood transfusion in the last 3 months.</td><td>Criteria was inserted based from site applicability. This additional criteria takes into consideration possible immunomodulation effects due to recent blood transfusions.</td></tr>
<tr><td>33</td><td>Phase 1a description on adverse events</td><td>The predominant adverse event has been local swelling, although, erythema and induration at the administration site has been noted.</td><td>The predominant adverse event was erythema and induration at the administration site (Table 4). To a lesser degree fever and fatigue were observed as systemic symptoms.</td><td>Updated the information according to statistical report and safety database</td></tr>
<tr><td>33</td><td>Table 4</td><td>Table 4. Summary of adverse events.
<table>
<tr><th></th><th>Group 1 (50µg)</th><th>Group 2 (100µg)</th></tr>
<tr><td>n</td><td>n=15</td><td>n=15</td></tr>
<tr><td>Swelling</td><td>13</td><td>13</td></tr>
<tr><td>Swelling and induration</td><td>2</td><td>0</td></tr>
<tr><td>Other possible severe effects</td><td>0</td><td>0</td></tr>
</table></td><td>Table 4. Summary of adverse events. There were no significant differences among the proportion of volunteers with an injection site reaction of any severity between dose groups.
<table>
<tr><th></th><th>Group1 (50mg)</th><th>Group2 (100mg)</th></tr>
<tr><td>Number of subjects (n)</td><td>15</td><td>15</td></tr>
<tr><td>Fatigue</td><td>2</td><td>2</td></tr>
<tr><td>Fever</td><td>2</td><td>4</td></tr>
<tr><td>Administration site reactions:</td><td></td><td></td></tr>
<tr><td>Erythema</td><td>14</td><td>10</td></tr>
<tr><td>Induration</td><td>15</td><td>10</td></tr>
<tr><td>Itchiness</td><td>0</td><td>4</td></tr>
<tr><td>Pain</td><td>0</td><td>1</td></tr>
<tr><td>Swelling</td><td>2</td><td>0</td></tr>
</table></td><td>For updating</td></tr>
<tr><td>35</td><td>1.6 Clinical Experience with Other Alum-based Adjuvant and Possible Adverse Reactions</td><td></td><td>For neurological adverse events of immunization (Expert Rev Vaccines, 2007: 6: 863-869; http://www.medscape.com/viewarticle/565691_3), the administration of DTP vaccine to infants has been associated with screaming, persistent crying, convulsions, encephalopathy and hypotonic, hyporesponsive episodes, but these signs have generally been linked to the pertussis component and not to the adjuvant. During the last 15 years, macrophagic myofasciitis (clinical presentations include myalgias, arthralgias, fatigue and multiple sclerosis; inflammatory myopathies); has been reported with aluminum adjuvanted vaccines, although the association of histologic lesions and the clinical symptoms is still considered an unproven hypothesis.</td><td>For updating</td></tr>
<tr><td>42</td><td>3.2.3 Blinding</td><td>For Stage 2, subjects will not be told that the different doses (½ and full dose) will vary with respect to volume,</td><td>removed</td><td>The information is provided in the ICD but care will be exercised to maintain</td></tr>
</table>

Page	Field	Before	After	Explanations
		in the event that very astute subjects will observe or discern how far syringe plungers extend from the barrel.		subject blinding.
43	4.1 Inclusion Criteria	The standard and acceptable ranges of laboratory test values in healthy children and adults are set following the baseline ranges at LMC (First column in Table 6).	The standard and acceptable ranges of laboratory test values in healthy children and adults are set following the baseline ranges at LMC (for Stage 1) and those obtained from the clinical investigators (for Stage 2).	Based from Stage 1 experience, it was deemed necessary to adopt Ugandan reference values so that it is more applicable to the trial site and volunteer population.
45	[Justification for the exclusion criteria]		Blood transfusion carries the risk of immunomodulation.	Justification for exclusion criteria <15>
48	6.1 Recruitment, Consent, Enrollment, Randomization and Administration		An alternative for Stage 2 if the desired age cohorts cannot be obtained, is a cohort of 33 volunteers for each dose of BK-SE36 (half and full-dose) and 9 volunteers for each dose of placebo.	The alternative treatment regime takes into consideration the statistical validity of the trial to detect any possible adverse event.
50	6.1 Recruitment, Consent, Enrollment, Randomization and Administration		Volunteers, who were evaluated by investigators as otherwise healthy but falls out of range from urinalysis, hematology or biochemical parameters can be re-evaluated again. If a particular exclusion criteria is met during screening, the investigator evaluates if this is clinically non-significant and/or the condition can be resolved prior to administration. If considered as healthy by doctors medical assessment, but falls out of screening range, the volunteer can be included after seeking a subject waiver.	Because current reference range for healthy volunteers may not entirely be representative of the study population normal ranges, clinical investigators assessment is important to avoid high screening failures of an otherwise healthy potential subject.
51	6.1 Recruitment, Consent, Enrollment, Randomization and Administration	A CRF will also be filled in for all subjects randomized	A CRF will be filled in for all subjects that were administered at Visit 2.	Correction (takes into consideration subject replacement at Visit 2)
51	6.1 Recruitment, Consent, Enrollment, Randomization and Administration		(Alternatively, additional visit 1 maybe conducted before randomization).	(For logistics purposes.) This takes into consideration that subject waivers maybe raised by the site and approval by the Sponsor and MBL-IRC is necessary; but subjects can come to the Centre for further evaluation/screening/ malaria treatment a week prior to administration day.
51	6.1 Recruitment, Consent, Enrollment, Randomization and Administration		For stage 2, this window period may be extended for an additional day (thus ± 2 days) .	Window period for stage 2 is extended for a day, taking into consideration logistics and subject compliance for study visits.
51	6.1 Recruitment, Consent, Enrollment, Randomization and Administration	In all subjects, the first and second administrations will be preferably injected on the left and right arms, respectively.	In all subjects, the first and second administrations will preferably be injected on opposite arms.	For site of administration, whichever arm to be administered first is not so important, although for observing AEs it is much easier and preferable that opposite arms would be

Page	Field	Before	After	Explanations
				injected.
52	6.1 Recruitment, Consent, Enrollment, Randomization and Administration	All data, except (a) the investigators evaluation for AE severity grade and causality, and (b) concomitant medication/treatment, will be recorded on source documents and transcribed into the CRF by trained investigators.	All data, except the investigators evaluation for AE severity grade and causality, will be recorded on source documents and transcribed into the CRF by trained investigators.	Removed: "and (b) concomitant medication/ treatment". Concomitant medications are captured also in medical records, SOP09.
52	6.2 Malaria Episodes and Management of Symptomatic Malaria	For any readings ≥37.5°C, a blood smear will be taken to confirm the diagnosis and all subjects with asexual parasitemia will be treated.	For any readings ≥37.5°C, or for any presumptive malaria diagnosis, a blood smear will be taken to confirm the diagnosis and all subjects with asexual parasitemia will be treated.	Irrespective of fever, investigator can gauge if subject needs to be tested for malaria smear
60	8.1 Safety	Weekly axillary temperatures will be obtained for all subjects to monitor malaria infection. If axillary temperature is ≥37.5°C, thick and thin blood smears will be made.	Weekly monitoring of malaria infection will be done either by axillary temperature readings or when clinical investigators deemed fit. If axillary temperature is ≥37.5°C, or presumptive malaria diagnosis is made, thick and thin blood smears will be made.	Irrespective of fever, investigator can gauge if subject needs to be tested for malaria smear.
60	8.1 Safety	A thick blood smear will be considered negative when the examination of 100 high power fields does not reveal asexual parasites or gametocytes.	A thick blood smear will be considered negative when the examination of 500 WBCs does not reveal asexual parasites or gametocytes.	Correction
72-73	Table 6, header and first column	[Values indicated in the first column [parameter] are reference ranges of these tests as obtained from LMC	Values indicated in the first column [parameter] are reference ranges of these tests as applicable for Stage 1. Reference ranges applicable for Stage 2 are appended in *Supplemental Reference 1*.	Correction<br>(Children reference ranges on the first column of the table for:<br>Hemoglobin,<br>WBC and<br>AL-P were removed)
77	11.3 Data Management Procedures	Case report forms (CRFs) will be used to record data of subjects enrolled in the trial.	Case report forms (CRFs) will be used to record data of subjects administered with investigational product.	Correction
78	11.3.1 Original documents: direct access and record keeping	Regarding items directly described in the case report that requires judgment by an investigator (AE severity grading and causality; concomitant medications), the case report is handled as an original document.	Regarding items directly described in the case report that requires judgment by an investigator (AE severity grading and causality; or when applicable, concomitant medications), the case report is handled as an original document.	Concomitant medications are captured also in medical records.
85	13.5.5 Payment	Compensation will cover for time spent, meal(s) and travel costs. Details will be available in the SOP [SOP for Clinical Trials at Lira Medical Centre].	Compensation will cover for time spent, meal(s) and travel costs at each trial visit. Details will be available in the ICD [*Informed consent documents*].	Correction
89	Supplemental Reference 1.		The standard and acceptable ranges of laboratory test values for Stage 2.	Laboratory reference ranges (Shaded grey areas, means no change from last protocol version)

SINGLE BLIND, RANDOMIZED, CONTROLLED, PHASE 1b TRIAL OF THE SAFETY AND IMMUNOGENICITY OF LYOPHILIZED RECOMBINANT PRECIPITATED TROPICAL MALARIA VACCINE (BK-SE36), IN UGANDA

ISRCTN No.: **ISRCTN71619711**

Running Title: ***BK-SE36 Phase 1b***

BIKEN Trial Number: BK-SE36/002

BIKEN Protocol Number: BK-SE36/002/P05

Version 4.0 (June 24, 2010)

The Research Foundation
for Microbial Diseases of Osaka University
3-1 Yamadaoka, Suita, Osaka 565-0871, Japan
http://www.biken.or.jp/
Contact information: c/o Department of Molecular Protozoology
Research Institute for Microbial Diseases (RIMD), Osaka University
3-1 Yamadaoka, Suita, Osaka 565-0871, Japan
Tel +81(6)-6879-8280
Fax +81(6)-6879-8281

INVESTIGATORS AND INSTITUTIONAL AFFILIATIONS

PRINCIPAL INVESTIGATOR	Thomas Egwang, PhD<br>Director General/Scientific Director<br>Med Biotech Laboratories,<br>Plot 3438 Muyenga, Tank Hill By-pass,<br>PO Box 9364, Kampala, Uganda<br>Tel +254-712-504 010; +256-775-494 752<br>*email*: doublestrand2000@yahoo.com
Co-PRINCIPAL INVESTIGATOR and CLINICAL TRIAL COORDINATOR	Adoke Yeka, MB. Ch.B., M.P.H.<br>Uganda Malaria Surveillance Project,<br>Makerere Medical School,<br>PO Box 7475, Kampala, Uganda<br>Tel +256 414 530 692; +256 772 473 533<br>*Fax* +256 414 540 524<br>*email*: yadoke@yahoo.com; yadoke@muucsf.org
CLINICAL INVESTIGATORS	Jane Achan, MB. Ch.B., M.Med Paed.<br>Uganda Malaria Surveillance Project,<br>Makerere Medical School<br>P.O. Box 7475<br>Kampala, Uganda<br>Tel + 256 772 410 183<br>*Fax* +256 414 540 524<br>*email:* achanj@yahoo.co.uk
	Edward Hosea Ntege, MB. Ch.B<br>Med Biotech Laboratories,<br>P.O. Box 9364, Kampala,Uganda<br>Tel + 256 772- 431 442<br>*email*: edwardsdoc@yahoo.com
	Christopher Nsereko, M.D.<br>c/o Lira Medical Centre,<br>P.O. Box 1075, Plot #15, Moroto Road,<br>Lira, Uganda<br>Tel +256-775-15 54 22<br>*email:* necochris@yahoo.co.uk
CLINICAL TRIAL SITE and CLINICAL LABORATORY	Lira Medical Centre<br>P.O. Box 1075, Plot #15, Moroto Road, Lira, Uganda<br>Tel +256-392-948 833; +256-772-419 397<br>(c/o Dr. Ogwang Ochoo Ben, Director, LMC)<br>*http://www.lira-medical-centre.org/*
Laboratory Investigators	Paul Byagami<br>Lira Medical Centre<br>P.O. Box 1075, Plot #15, Moroto Road, Lira, Uganda<br>Tel +256-392-948 833; +256-772-419 397

SITE MANAGEMENT ORGANIZATION and UGANDA RESEARCH LABORATORY	Med Biotech Laboratories,<br>Plot 3438 Muyenga, Tank Hill By-pass,<br>PO Box 9364, Kampala, Uganda<br>Tel +254-712-504 010
Laboratory Investigator	Bernard N. Kanoi<br>Tel +256 782 334 853<br>*email:* bnkanoi@gmail.com

CONTRACT RESEARCH ORGANIZATION (CRO)

Associate Director<br>Clinical Operations<br>Quintiles South Africa	Charlean Kleynhans<br>QUINTILES Centurion (QCEN) South Africa<br>1011 Pretorius Ave South Lyttelton Manor<br>0157, South Africa<br>Tel +27 12 671 2335<br>*Fax* + 27 12 671 2388/2238<br>*email :* charlean.kleynhans@quintiles.com
Project Manager	Mientjie Barnard<br>Tel +233 26 300 2160<br>*Fax* + 27 12 671 2388/2238<br>email: mientjie.barnard@quintiles.com
Executive Director<br>Clinical Operations<br>Quintiles South Africa	Sorika van Niekerk<br>Tel +27 12 671 2333<br>*Fax* + 27 12 671 2283<br>*email :* sorika.vanniekerk@quintiles.com
Senior Clinical Trial Assistant	Anita Greeff<br>Tel +27 12 672 6509<br>*Fax* + 27 12 671 2388/2238<br>*email :* anita.greeff@quintiles.com

SPONSOR and JAPAN RESEARCH LABORATORY (1)

	Yasushi Higashi<br>Chairman, The Board of Directors<br>The Research Foundation<br>for Microbial Diseases of Osaka University<br>3-1 Yamadaoka, Suita, Osaka 565-0871, Japan<br>Tel +81(6)-6877-4804<br>*Fax* +81(6)-6876-1984<br>http://www.biken.or.jp/
Responsible Person for Clinical Trial Supervision	Shigeharu Ueda, M.D., D.M.Sc.<br>Professor Emeritus of Osaka University<br>Director, The Research Foundation for Microbial Diseases of Osaka University<br>3-1 Yamadaoka, Suita, Osaka 565-0871, Japan<br>Tel +81(6)-6877-3305<br>*Fax* +81(6)-6876-1984

Responsible Person for Clinical Trial Enforcement

Hiroki Shirai
Kanonji Institute, The Research Foundation for Microbial Diseases of Osaka University
2-9-41 Yahata-cho, Kanonji, Kagawa 768-0061
Japan
Tel +81(875)-25-4171
Fax +81(875)-25-4817
email: hshirai@mail.biken.or.jp

GCP Audit Section

Hiroyuki Fujita
Kanonji Institute, The Research Foundation for Microbial Diseases of Osaka University
2-9-41 Yahata-cho, Kanonji, Kagawa 768-0061
Japan
Tel +81(875)-25-4857
Fax +81(875)-25-4838
email: ajfujita@mail.biken.or.jp

Surveillance Section
Surveillance Center

Yoshinobu Okuno
Kanonji Institute, The Research Foundation for Microbial Diseases of Osaka University
2-9-41 Yahata-cho, Kanonji, Kagawa 768-0061
Japan
Tel +81(875)-25-4171
Fax +81(875)-25-4817
email: yokuno@mail.biken.or.jp

MEDICAL ADVISERS, RESEARCH COORDINATOR and JAPAN RESEARCH LABORATORY (2)

Toshihiro Horii, PhD
Professor
Department of Molecular Protozoology
Research Institute for Microbial Diseases (RIMD), Osaka University
3-1 Yamadaoka, Suita, Osaka 565-0871, Japan
Tel +81(6)-6879-8280
Fax +81(6)-6879-8281
email: horii@biken.osaka-u.ac.jp

Ken Ishii, M.D., PhD.
Principal Investigator,
Laboratory of Vaccine Science
WPI, IFReC, Osaka University
Tel +81(6)-6879-8303
Fax +81(72)-641-8043
email: kenishii@biken.osaka-u.ac.jp

Nirianne Palacpac, PhD
Assistant Professor
Tel +81(6)-6879-8280
Fax +81(6)-6879-8281
email: nirian@biken.osaka-u.ac.jp

STATEMENT OF INVESTIGATORS:

I, the undersigned, have reviewed and understood this protocol, including Appendices (SOPs and Forms), and will ensure that the clinical trial as described will adhere to the principles of the ICH/GCP as well as all applicable regulatory requirements.
Thomas Egwang, PhD
Principal Investigator

____
Signature

____
Date

I, the undersigned, have reviewed and understood this protocol, including Appendices (SOPs and Forms), and will ensure that the clinical trial as described will adhere to the principles of the ICH/GCP as well as all applicable regulatory requirements.
Adoke Yeka, MB. Ch.B., M.P.H.
Co-Principal Investigator

____
Signature

____
Date

I, the undersigned, have reviewed and understood this protocol, including Appendices (SOPs and Forms), and will ensure that the clinical trial as described will adhere to the principles of the ICH/GCP as well as all applicable regulatory requirements.
Jane Achan, MB. Ch.B., M.Med Paed.
Co- Investigator

____
Signature

____
Date

I, the undersigned, have reviewed and understood this protocol, including Appendices (SOPs and Forms), and agrees to conduct the clinical trial as described, and will adhere to the principles of the ICH/GCP as well as all applicable regulatory requirements.
Edward Hosea Ntege, MB. Ch.B
Co-Investigator

____
Signature

____
Date

I, the undersigned, have reviewed and understood this protocol, including Appendices (SOPs and Forms), and agrees to conduct the clinical trial as described, and will adhere to the principles of the ICH/GCP as well as all applicable regulatory requirements.
Christopher Nsereko, M.D.
Co-Investigator

____
Signature

____
Date

STATEMENT OF COMPLIANCE

This trial will be carried out in accordance with the ICH HARMONISED TRIPARTITE GUIDELINE FOR GOOD CLINICAL PRACTICE E6(R1) (ICH-GCP) standards, regulatory authorities requirements, this protocol and the standard operation procedures (SOPs) appended.

The protocol and SOPs are based on the ethical principles stated in the:

Declaration of Helsinki: "Recommendations Guiding Medical Doctors in Biomedical Research Involving Human Subjects," adopted at the General Assembly of the World Medical Association held in Helsinki in 1964 (revised 2000 at Edinburgh, Scotland);

ICH Guidelines for Clinical Trials E6 (R1) CPMP/ICH/135/95 (updated 2002);

"*Departmental Regulations for Standards of Implementation of Clinical Trial with Drugs and Medicines (GCP),*" (Japan Ministry of Health and Welfare Ordinance No. 28, March 27, 1997);

"Departmental Regulations for Revised Standards of Implementation of Clinical Trial with Drugs and Medicines (GCP)," (Japan Ministry of Health, Labour and Welfare Ordinance No. 106, June 12, 2003);

"*National Guidelines for Research Involving Humans as Research Participants,*" Ugandan National Council for Science and Technology (UNCST) (Kampala, Uganda; March 2007);

and "*Guidelines for the Conduct of Clinical Trials,*" National Drug Authority (NDA) (Kampala, Uganda; December 2007).

The eligibility to conduct this trial, with regard to ethical and scientific validities, will be examined beforehand by the Scientific Committee/Institutional Review Committee of the Research Institute for Microbial Diseases, Osaka University (RIMD) and Med Biotech Laboratory (MBL-IRC); and applied for approval to the regulatory authorities of UNCST and the NDA.

To be continuously reviewed by the MBL-IRC and regulatory authorities, UNCST and NDA, the principal investigator will submit documents on the current state of the trial to the institutions when requested.

TABLE OF CONTENTS

LIST OF ABBREVIATIONS……………………………………………………......…11

PROTOCOL SYNOPSIS..………..………………………………………… 12

REFERRED REPORTING TIMELINES IN THE PROTOCOL FOR THE PRINCIPAL INVESTIGATOR AND CO-PRINCIPAL INVESTIGATOR…...18

1.0 **INTRODUCTION AND RATIONALE**

1.1 The Burden of Malaria…………………………………………………20
1.2 Rationale for Selection of the Vaccine Candidate………………………22
1.3 The Serine Repeat Antigen (SERA)……………………………………22
1.3.1 Evidence of SERA as a vaccine candidate…………………..23
1.3.2 From SE47' to SE36 protein ………………………………. 24
1.4 Investigational Product Description
1.4.1 Generic name………..………….………- - -....….....-…...…26
1.4.2 Description……..……...…..……………......-……..…-..-......…26
1.4.3 Active ingredient and Excipients ...…………………........……..26
1.4.4 Preclinical studies with BK-SE36 …..………….…..........………26
1.4.5 Toxicology studies with BK-SE36…………….……......- …...28
1.5 Clinical Experience with BK-SE36 ………………..………………31
1.6 Clinical Experience with Other Alum-based Adjuvant and Possible Adverse Reactions……………………………………………………..34
1.7 Clinical Experience with Recombinant Protein Vaccines ….…. ………35
1.8 Clinical Development Plan …………………………………………..35
1.9 Justification for Administration Route, Dose and Frequency
1.9.1 Route of administration ……………………........……………..36
1.9.2 Dose and frequency of administration ……………….............…36
1.10 Rationale for Physiological Saline as Placebo ………………………36
1.11 Rationale for Trial Design: Two stages. Stepwise. Single Blind.….36

2.0 **OBJECTIVES**

2.1 **Stage 1**, 21 to 40 y old
2.1.1 Primary objective……………………………………………...37
2.1.2 Secondary objective…………………………………………...37
2.2 **Stage 2**, 6 to 20 y old
2.2.1 Primary objective……………………………………………...38
2.2.2 Secondary objective…………………………………………...38

3.0 **TRIAL DESIGN** …..…………………………………………………….38

3.1 Primary and Secondary Endpoints
3.1.1 Primary endpoint……………………………………………...39
3.1.2 Secondary endpoint…………………………………………...39

3.2 Trial Design. Phase 1b, Stage 1 and 2. Single blind, randomized ….….39
3.2.1 Trial size, gender and age…………....…………………….….…….40
3.2.2 Randomization …………………………. . ……......……...….…..42
3.2.3 Blinding………..……..42

4.0 **TRIAL POPULATION**

4.1 Inclusion Criteria…………………………………………………….43
4.2 Exclusion Criteria ……………………………………………………44
4.3 Treatments Potentially Interfering with Vaccine-induced Immunity……45
4.4 Contradictions to Administration ………………………………………46
4.5 Indications for Deferral of Administration ……………………………..46

5.0 **TRIAL INTERVENTION: INVESTIGATIONAL PRODUCTS** ……….47

6.0 **CONDUCT OF THE TRIAL**

6.1 Recruitment, Consent, Enrollment, Randomization and Administration..48
6.2 Malaria Episodes and Management of Symptomatic Malaria…………52
6.3 Concomitant Medication/Treatment ………………………………....53
6.4 Health Care Provision ……………………………….………………53
6.5 Trial Staff ……………..…………………………………….............53

7.0 **TRIAL SCHEDULE/TIME SEQUENCE**

7.1 Detailed Description of Trial Visits……………………………………..54
7.2 Outline of Trial Procedures ……………………………………………58

8.0 **TRIAL PARAMETERS AND LABORATORY TESTS**

8.1 Safety ……………………………………………………………….60
8.2 Immunogenicity ……………………………………………………..61

9.0 **ADVERSE EVENTS**……...........…………..……………………………...61

9.1 Definition of Adverse Events …….……………………………………61
9.2 Definition of Serious Adverse Events ………. …………………………62
9.3 Identification of Adverse Events………………………………………..62
9.4 Following-up of Adverse Events and Assessment of Outcome ………...63
9.5 Reporting of Serious Adverse Events ……………...………………….. 63
9.6 Treatment and Actions Taken Upon Occurrence of AEs and SAEs…..64
9.7 Discontinuation Criteria Due to Adverse Events ……………………..64
9.8 Stopping Criteria ……………………………………………………..64
9.9 Assessment of AEs and SAEs ……………………………………….....65

10.0 **SUBJECT COMPLIANCE AND WITHDRAWAL**

10.1 Responsibility of the Principal Investigator and Co-Principal Investigator with Regards to Subject Management …………………75

10.2 Subject Withdrawal/Drop-out
10.2.1 Withdrawal by subject ……………………………………..76
10.2.2 Ineligibility to be a subject ……………...........……………….76
10.2.3 Subject replacement ………………..………......…..……..76
10.2.4 Subject drop-out ………..........................….….......…………...76

11.0 **DATA COLLECTION, MANAGEMENT AND ANALYSIS**

11.1 Collection
11.1.1 Primary endpoints ……………………………………….76
11.1.2 Secondary endpoints ……………………………………..77
11.2 Trial Groups/Datasets to be Evaluated
11.2.1 Total cohort …………………………………………………77
11.2.2 Safety cohort ……………………………………………...77
11.2.3 Immunogenicity cohort …………………………………...77
11.3 Data Management Procedures
11.3.1 Original documents: direct access and record keeping…........77
11.3.2 Case report form…..……………..………………………….78
11.4 Statistical Methods and Consideration…………………………………79
11.4.1 Other assessments ……..……………………………………79
11.4.2 Safety ………………………………………………………80
11.4.3 Efficacy ……………………………………………………80
11.4.4 Handling of cases …………………………………….......80
11.5 Retention of Records ………………………………………………81

12.0 **QUALITY CONTROL AND QUALITY ASSURANCE** ………………81

13.0 **ETHICAL CONSIDERATIONS**

13.1 Statement of Compliance and Ethical Reviews………………………..82
13.2 Institutional Review Committee ………………………….…..……82
13.3 Other Committees
13.3.1 Community Advisory Board…………………………………83
13.3.2 Data and Safety Monitoring Board…………………….……..83
13.4 Informed Consent……………………………………………………84
13.5 Risk and Potential Benefits
13.5.1 Administration ………………………………………………84
13.5.2 Precautions ………..………………………………………...84
13.5.3 Benefits ..……………………………………………………..85
13.5.4 Medical treatment for subjects.………………………………85
13.5.5 Payment ……….……………………………………………85
13.5.6 Compensation for health problems of subjects ……………...85

14.0 **CONFIDENTIALITY**……………………………………….…………….……85

15.0 **FINANCING AND INSURANCE** ..……………………………………..86

16.0 **PUBLICATION POLICY**……………………………………………………..86

17.0 **REFERENCES**………………………………………………………………86

18.0 **APPENDICES**

18.1 Flow chart of all trial stages from regulatory approval to post trial follow-up…………………………………………………………………91

18.2 Informed Consent Forms (ICF)

18.2.1 ICF for Adults: 21 to 40 y old……………………………......…… 94

18.2.2 ICF for Adults: 18 to 20 y old ………….………………….........101

18.2.3 ICF for Parent(s)/Guardian(s): 6 to 7 y old……………..........…108

18.2.4 ICF for Children and Parent(s)/Guardian(s): 8 to 17 y old…......115

18.3 Additional Documents for All Informed Consent

18.3.1 Trial Schedule and Activities 122

18.3.2 Form for Impartial Witness for Volunteers Unable to Read or Write……......…………..……………………..125

18.3.3 Consent Form for Identification Photograph …...….....……….126

18.3.4 Informed Consent for Future Use of Blood Samples..............127

LIST OF ABBREVIATIONS

ADCI	Antibody-dependent cell-mediated inhibition assay
AE	Adverse Event
AIDS	Acquired Immune Deficiency Syndrome
A/G	Albumin/Globulin ratio
AL	Artemether/lumefantrine
AL-P	Alkaline phosphatase
ALT	Alanine aminotransferase
AST	Aspartate aminotransferase
BIKEN	The Research Foundation for Microbial Diseases of Osaka University (Japan)
BK-SE36	Lyophilized recombinant precipitated tropical malaria vaccine
CRF	Case Report Form
CRO	Contract Research Organization
CRP	C-reactive protein
DP	Dihydroartemisinin-piperaquine
ELISA	Enzyme linked immunosorbent assay
GCP	Good Clinical Practice
GIA	Growth inhibition assay
GMP	Good Manufacturing Practice
γ-GTP	Gamma glutamyl transpeptidase
β-hCG	Beta-human chorionic gonadotropin
HIV	Human immunodeficiency virus
IC	Informed Consent
ICF	Informed Consent Form
ICH	International Conference on Harmonization [of Technical Requirements for Registration of Pharmaceuticals for Human Use]
ID	Identification photograph
IRC	Institutional Review Committee
LAP	Leukocyte alkaline phosphatase
LDH	Lactate dehydrogenase
LMC	Lira Medical Centre (Uganda)
MBL	Med Biotech Laboratories (Uganda)
MHLW	Ministry of Health, Labor and Welfare (Japan)
NCHS	National Center for Health Statistics (US)
NDA	National Drug Authority (Uganda)
PI	Principal Investigator
PT	Preferred Term
RIMD	Research Institute for Microbial Diseases (Japan)
SAE	Serious Adverse Event
SE36	SE47’ without serine repeats
SE47’	N-terminal 47-kDa domain of SERA
SERA	Serine Repeat Antigen 5
SOC	System Organ Class
SOP	Standard Operating Procedure
UNCST	Ugandan National Council for Science and Technology
WHO	World Health Organization

PROTOCOL SYNOPSIS

Title

Single blind, randomized, controlled, Phase 1b trial of the safety and immunogenicity of lyophilized recombinant precipitated tropical malaria vaccine (BK-SE36), in Uganda

Running Title

BK-SE36 Phase 1b

Rationale

Combating malaria is a complex proposition. No vaccine exists today against malaria. Research identified SE36 protein based from the N-terminal domain of Serine Repeat Antigen (SERA5) of *Plasmodium falciparum*, as a vaccine candidate with strong potential. Immunoepidemiological data underscores the uniqueness of SERA vs. other candidates, showing a semi-perfect correlation of the naturally induced antibody response to SE36 protein with increased protective immunity in adults and children. GMP-grade SE36 was formulated adsorbed to aluminum hydroxide gel as BK-SE36. The preclinical toxicity, safety and reactogenicity studies demonstrate that BK-SE36 was immunogenic and well tolerated. In a small clinical trial in malaria-naïve adults in Japan, BK-SE36 was proven safe and immunogenic. This will be the first trial in an endemic area and will evaluate the safety and immunogenicity of BK-SE36 in malaria-exposed individuals aged 6-40 y old.

The trial will be conducted in two stepwise stages:
Stage 1 population will be healthy adults aged 21 to 40 y old serologically negative (sero-negative) and positive (sero-positive) against the SE36 protein.
Stage 2 population will be healthy 6 to 20 y olds. Stage 2 will also assess the safety and immunogenicity of 50 μg and 100 μg dose levels of BK-SE36. This stage will only proceed after a safety assessment of Stage 1, (go or no-go). *An overall view of trial stages from regulatory approval to post trial follow-up is presented in the Appendix, Section 18.1, p.91.*

Objectives

<u>Stage 1, 21 to 40 y old</u>
Primary
To assess the safety and reactogenicity of BK-SE36 subcutaneously administered 2 times (at a 21 ± 1 day interval) to healthy male and female adults, sero-negative and sero-positive against the SE36 protein.

Secondary
To examine changes in the immune response as a result of 2 times administration of BK-SE36 in healthy male and female adults sero-negative and sero-positive against the SE36 protein.

<u>Stage 2, 6 to 20 y old</u>
Primary
To assess the safety and reactogenicity of 50 μg and 100 μg BK-SE36 subcutaneously administered 2 times (at a 21 ± 1 day interval) to healthy malaria-exposed 6 to 20 year olds.

Secondary
To examine changes in the immune response as a result of 2 times administration of 50 μg and 100 μg BK-SE36 in healthy malaria-

exposed 6 to 20 year olds.

Investigational Products

(1) Test vaccine: BK-SE36
Supplied as white, lyophilized powder; when reconstituted with 1.3 ml of the supplied diluent, the solution contains 100 µg of *P. falciparum* SE36 protein.

Dose: Administration of 1.0 ml will deliver 100 µg SE36.
Administration of 0.50 ml will deliver 50 µg SE36.

(2) Placebo: physiological saline
Achromatic transparent solution containing 0.9% sodium chloride.

Trial Design

- Phase 1b, two-stage, stepwise:

Stage 1: 2 cohorts, 1 dose (100 µg SE36)
Sero-negative, male: BK-SE36, placebo
female: BK-SE36, placebo
Sero-positive, male: BK-SE36, placebo
female: BK-SE36, placebo

Stage 2: 3 cohorts, 2 doses:
6 to 10 y old: 50 µg SE36, 100 µg SE36;
0.5 ml placebo, 1.0 ml placebo;
11 to 15 y old: 50 µg SE36, 100 µg SE36;
0.5 ml placebo, 1.0 ml placebo;
16 to 20 y old: 50 µg SE36, 100 µg SE36;
0.5 ml placebo, 1.0 ml placebo

- One trial center: Lira Medical Centre (LMC)
- Single blind, randomized, placebo (physiological saline) controlled
- Administration schedule: Trial days 1 and 22
- Route: Subcutaneous administration (upper arm)
- Active surveillance (continuous/scheduled clinic visits) for adverse events: approximately 6 weeks per subject (excluding screening)
- Post-trial follow-up (passive surveillance or voluntary clinic visits): 40 days after visit 10

Number of Subjects

(1) Stage 1, 21 to 40 y old: 56 subjects;

	BK-SE36		Placebo	
	Male	Female	Male	Female
Sero-negative	9	9	5	5
Sero-positive	9	9	5	5

(2) Stage 2, 6 to 20 y old : 84 subjects;

	BK-SE36		Placebo	
	50µg	100µg	0.5 ml	1.0 ml
6 to 10y	11	11	3	3
11 to 15y	11	11	3	3
16 to 20y	11	11	3	3

**Inclusion/Exclusion Criteria**	

Inclusion/Exclusion Criteria

[Inclusion 1]: Stage 1, 21 to 40 y old

Healthy subjects are specified, and the inclusion criteria of malnutrition index and laboratory test values were included to reduce individual variation.

<1.1> Healthy adult. Ugandan males and females aged 21 to 40 (age on informed consent);

<1.2> Those who do not suffer from severe malnutrition (defined as an adult whose weight-for-height is below -3 standard deviation or less than 70% of the median of the NCHS/WHO normalized reference values);

<1.3> Those who are able to agree, comply with matters to be observed during participation in the trial, undergo consultation/examination, as described in this protocol, and report symptoms;

<1.4> Those who are considered to be eligible to participate in this trial based on screening

<1.4.1> Vital signs and physical examination are within baseline range

<1.4.2> Hematology: Within 25% deviations from the upper and lower limits of the baseline range. The differential white blood count is not questioned when the white blood cell count is within the baseline range.

<1.4.3> Blood chemistry:

- AST, ALT, and creatinine: Within the baseline range
- Total bilirubin: Within 50% deviation from the upper limit.
- Serum electrolytes: Within the baseline range
- Other blood chemistry items: Within 25% deviation from the upper and lower limits of the baseline range.

<1.4.4> Urinalysis: Within the normal range

[Inclusion criteria 2]: Stage 2, 6 to 20 y old

Healthy subjects are specified, and the inclusion criteria of malnutrition index and laboratory test values were included to reduce individual variation.

In this stage, there will be 3 different informed consent (IC) requirements:
IC for children aged 6 to 7 y will be solicited from parent(s)/guardian(s)
IC for ages 8 to 17 y will be solicited from both child and parent(s)/guardian(s)
IC for ages 18 to 20 y will be solicited from the volunteers themselves

<2.1> Volunteers, irrespective of gender, aged 6 to 20 years (age on informed consent)

<2.2> Those who do not suffer from severe malnutrition, whose BMI is between 5th percentile to less than the 85th percentile for 6-19 y old; and between 18.5-25.0 for 20 y old;

<2.3> Those who can give affirmative agreement to participate in the trial. For children between 8 to 17 y, the child’s assent takes

precedence over the parent(s)/guardian(s) consent.

<2.4> Those who are able to agree, comply with matters to be observed during participation in the trial, undergo consultation/examination, as described in this protocol, and report symptoms;

<2.5> Those who are considered to be eligible to participate in this trial based on screening:

<2.5.1> Vital signs and physical examination are within baseline range

<2.5.2> Hematology: Within 25% deviations from the upper and lower limits of the baseline range. The differential white blood count is not questioned when the white blood cell count is within the baseline range.

<2.5.3> Blood chemistry:

- AST, ALT, and creatinine: Within the baseline range
- Total bilirubin: Within 50% deviation from the upper limit.
- Serum electrolytes: Within the baseline range
- Other blood chemistry items: Within 25% deviation from the upper and lower limits of the baseline range.

<2.5.4> Urinalysis: Within the normal range

Exclusion criteria for both Stage 1 and 2

Any subject meeting any of the exclusion criteria at baseline will be excluded from trial participation.

<1> Persons with fever (37.5°C or higher) on administration of the test vaccine;

<2> Persons with a clear history of food/drug-related anaphylaxis;

<3> Females (adolescents/adults) who are pregnant or have a positive urine β-hCG on the day of, or prior to, administration;

<4> Females currently lactating or breast-feeding;

<5> Persons with acute or chronic cardiovascular, pulmonary, hepatic, renal, or neurological condition, which in the opinion of the investigator may increase the risk of the subject from participating in the trial;

<6> Persons with a history of fever within 2 days after preventive administration with other types of vaccine, or those in whom symptoms have suggested systemic allergy;

<7> Persons with a history of convulsion other than febrile convulsions in malaria in the past 6 months to 1 year

<8> Persons with any confirmed or suspected immunosuppressive or immunodeficient condition, including human immunodeficiency virus (HIV) infection. (No infectious disease testing will be conducted. HIV testing will not be done. Severe, suspected infectious diseases will be ruled out by investigators during physical examination/consultation, blood hematology/chemistry tests; although not conclusive of the causative agent);

Additional oral confirmation: Subject informed the investigator

that he/she has been tested positive for HIV/AIDS. (Information on the child's HIV status could be obtained from their parents or guardians.)

<9> Persons with a history or tentative diagnosis of drug allergy; especially to common drugs like penicillin, sulphonamides, etc.

<10> Persons with (history of) chronic alcohol consumption and/or illicit drug use;

<11> Persons who took any medication within 1 week before administration of this test vaccine (except for artemether/lumefantrine and dihydroartemisinin-piperaquine);

<12> Persons to whom any live vaccine was administered within 4 weeks before administration of this test vaccine, or inactivated vaccine/toxoid was administered within 1 week;

<13> Persons who participated in another trial within 4 months before administration of this test vaccine; or simultaneous participation in any other clinical trial;

<14> Persons in whom 200 ml of blood was collected (donation) within 1 month before administration of this test vaccine, or more than 400 ml of blood was collected within 3 months;

<15> Persons who have recently undergone blood transfusion in the last 3 months.

<16> Others who are not considered to be eligible by the investigator or those whose medical condition would, in the opinion of the investigator, make the subject unsuitable for the trial.

Observation/Examination and Timing

(1) Consultation and physical examination

Consultations; physical examination, including blood pressure (systolic/diastolic), pulse rate, body temperature, will be performed by the investigators on screening, before the first and second administration; 1 hour and; 1, 7, 14 and 21 days after each administration.

(2) Laboratory tests

13 ml of blood and 10 ml of urine (mid-stream urine) will be solicited on screening, before the first and second administration, and on 21 days post administration (final visit). Additionally, 10 ml of urine (mid-stream urine) will also be solicited 1 and 14 days after the first and second administration. Laboratory tests will measure the following parameters:

<1> Hematology

Leukocyte count, erythrocyte count, hemoglobin, hematocrit, platelet count

<2> Blood chemistry

Total protein, albumin, total bilirubin, AST, ALT, AL-P, γ-GTP, serum amylase, total cholesterol, uric acid, urea nitrogen, creatinine, Na, K, glucose

<3> Malaria smear

<4> Urinalysis and urine β-hCG
Qualitative glucose, bilirubin, ketone body, urinary occult blood reaction, pH, protein, urobilinogen, nitrite, leucocytes, specific gravity; β-hCG reaction for female subjects (adolescents/adults)

(3) Body weight and height
Body height will be measured on screening.
Body weight will be measured on screening, before first and second administration and 21 days post administration (final visit)

(4) Measurement of the anti-*P. falciparum* (SE36 protein) antibody titer on screening (Stage 1 only), before the first and second administration, and on 21 days post administration (final visit).

Endpoints

(1) Primary endpoints
The safety of BK-SE36 will be assessed by the presence or absence of adverse events. This information will be gathered from patient symptoms, vital signs and laboratory test results obtained 1 hour after administration; 1, 7, 14 and 21 ±1 days after each administration.

(2) Secondary endpoints
Changes in the anti-SE36 protein antibody titer at each time point: screening, before each administration and 21 ± 1 day post administration (final visit).

Principal Investigator for this Clinical Trial

Thomas Egwang, PhD
Director General/Scientific Director
Med Biotech Laboratories,
Plot 3438 Muyenga, Tank Hill By-pass,
PO Box 9364, Kampala, Uganda
Tel +254-712-504 010; +256-775-494 752
email: doublestrand2000@yahoo.com

Trial Site

Lira Medical Centre
P.O. Box 1075, Plot #15,
Moroto Road, Lira, Uganda
Tel +256-392-948 833; +256-772-419 397
(c/o Dr. Ogwang Ochoo Ben, Director)
http://www.lira-medical-centre.org/

REFERRED REPORTING TIMELINES IN THE PROTOCOL FOR THE PRINCIPAL INVESTIGATOR AND CO-PRINCIPAL INVESTIGATOR

1. <u>Protocol Amendments</u>:

During emergency, if necessary to protect life, changes can be effected immediately. The sponsor will be informed within 24 hours by telephone, email or fax. A written full explanation will be provided within 48 hours to the sponsor, MBL-IRC, UNCST and NDA.

To		Within	By
Sponsor	First notice	24 hours	Telephone, e-mail, fax
	Full explanation	48 hours	e-mail, with report
MBL-IRC UNCST NDA	First notice	48 hours	Telephone
	Full explanation	7 working days	Report

For safety reasons, but not pertaining to emergency cases, the amendment must be submitted in full and implementation should wait until approval of MBL-IRC, UNCST and NDA.

For items without safety impact, change(s) can be implemented 14 days after filing with NDA unless the investigator(s) receives a document stating otherwise.

2. <u>Protocol Violations and Deviations</u>: Upon discovery of the event, the sponsor will be informed within 24 hours by telephone, email or fax. A report will also be filed to MBL-IRC and UNCST within 7 days.

To		Within from first discovery	By
Sponsor	First report	24 hours	Telephone, e-mail, fax
	Full explanation	3 working days	e-mail, with report
MBL-IRC UNCST NDA*	First notice	7 working days	Report

*where health related

3. <u>SAE Reporting</u>: Upon occurrence of the event, the sponsor will be informed within 24 hours. A written report will also be filed within 3 working days to the sponsor; and within 7 days to MBL-IRC, UNCST and NDA. Additional follow-up reports should not be later than 15 days of the event.

To		Within from first discovery	By
Sponsor	First notice	24 hours	Telephone, e-mail, fax
	Full explanation	3 working days	e-mail with report
MBL-IRC UNCST NDA	First notice	7 calendar days	Report
	Additional follow up	15 calendar days	Report

4. End of Trial Summary Report: Principal investigator will report to the regulatory authorities within 3 months from the Last Subject Out date for both Stage 1 and 2.

5. Product Accountability and Disposal Report: Principal investigator will report to the regulatory authorities within 3 months from the Last Subject Out date for both Stage 1 and 2.

**Final Report: Principal investigator and sponsor will report to the regulatory authorities not later than 3 months from the Last Subject Out date of Stage 2.

1 INTRODUCTION AND RATIONALE

1.1 The Burden of Malaria

Malaria remains as the most important infectious disease globally. With about 2.2 billion of the world's population at risk, the incidence of malaria worldwide is estimated to be 500 million cases each year, killing between 1 and 3 million people or an estimated 7000 deaths per day.

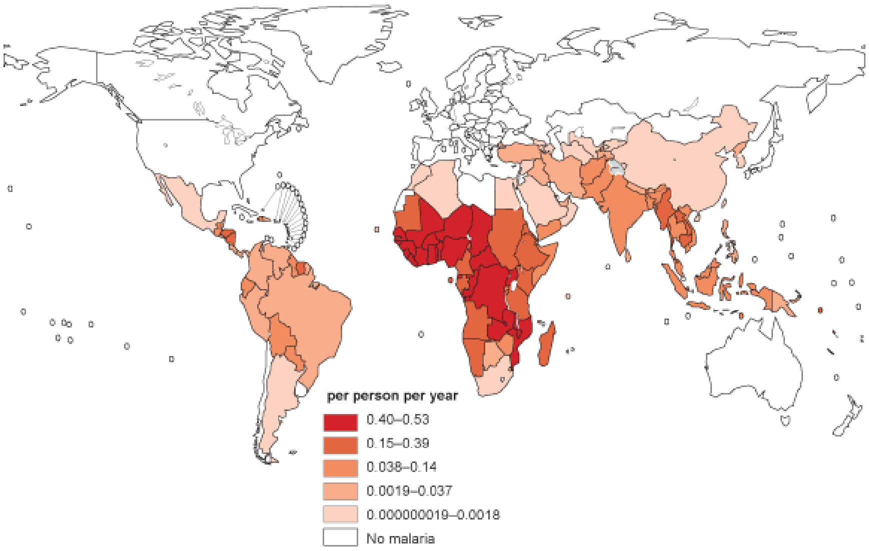

http://www.rbm.who.int/wmr2005/html/map3.htm

Estimated incidence of clinical malaria episodes - caused by any species - resulting from local transmission, country level averages, 2004.

Most of the mortality occurs among children under 5 years of age in sub-Saharan Africa. In Japan, some 100 imported malaria cases are reported annually and more than half of the members of the Japan Overseas Cooperation Volunteers who were sent to the tropics and subtropics had experience infection, some of whom died of malaria. Of the four species of malaria that infect humans, *Plasmodium (P.) falciparum* is responsible for the majority of these deaths.

Current strategies for malaria control include (1) preventive administration and treatment of patients with antimalarial drugs; and (2) mosquito vector control (indoor residual spraying, insecticide repellants, use of insecticide-treated bed nets).

However, the increasing loss of effectiveness of major antimalarials like chloroquine and sulfadoxine-pyrimethamine due to emergence of drug-resistant malaria parasites; and the emergence of *Anopheles* mosquitoes which have acquired resistance to insecticides necessitates the need for vaccine development.

Disease burden in Uganda. In Uganda, malaria poses a formidable burden in a population trying to cope with other pressing health problems like HIV/AIDS and tuberculosis (Okech et al., 2006). Twenty-five to thirty percent of all outpatient visits and 300 infant deaths daily are due to malaria. Stable malaria occurs in about 95% of the country, with high transmission rates in areas <1,200 m altitude and low to medium

transmission in areas between 1,200 and 1,600 m altitude. The emergence of drug resistant parasite strains has been reported (Talisuna et al., 2003) and, at present, insecticide-treated net coverage is limited to only 13% (Okello et al., 2006).

In Northern Uganda (Apac District; 1,150 m altitude), about 250 km from Kampala (Uganda capital city), the population prevalence of parasitaemia in 1995 was 62.1% with the predominant species being *P. falciparum* (100%) and *P. malariae* in the minority (Egwang et al., 2000). Majority of malaria clinical cases were children under ten years (85%). In 1999, parasite prevalence was reported to be 53% for all ages (79% for children aged 2 to 9 years) (Talisuna et al., 2003). A recent estimate from the Uganda Ministry of Health in 2007 showed that the district has the highest entomological inoculation rate, with about 1,600 bites per year (Okello et al., 2006; Gissel, 2008). Translating to disease burden, the incidence of clinical *P. falciparum* malaria in children <18 months and mortality in children <1 year of age increased (Okello et al., 2006). During the months of January-February and August 2007, there were 10,920-11,952 to 47,968 registered cases of malaria, with almost half of these from children aged four and below (Gissel, 2008). Malaria remains as one of the major causes of maternal mortality.

Lira District, 347 km north of Kampala, lies east of Apac. The district covers an area of 7,251 sq km with an average altitude of 1,170 m above sea level. The annual rainfall range is between 1,200 and 2,000 mm, which peak in the months of April, May, August and October. The average temperature is 30 ^{o}C. Similar to Apac, malaria is one of the biggest health challenges in the region, contributing to 28.8 percent of total time and productivity lost (http://www.lira.go.ug/). Seasonal and papyrus swamps, and nearby lakes contributes heavily to the persistence of anopheline mosquito breeding sites. Because of these breeding sites, the high entomological inoculation rate, and observed cumulative malaria attack rates during the rainy season, the adult and children population are predicted to be susceptible to malaria infection and/or parasitemic multiple times over a lifetime. There is no doubt whatever that the population is "malaria-exposed."

In a 2002 census, Lira district has a population of 757,763 people, representing 3.1% of the national population; Apac, 676,244. Annual population growth rate at each district varies between 3.41 and 3.5 percent per annum. In both districts, about 99% belongs to the Lango tribe; making it more or less a homogenous society. Luo is the main language spoken in the dialect.

Trial Site. Since 1995, the Department of Molecular Protozoology, Research Institute for Microbial Diseases (RIMD) and Med Biotech Laboratories (MBL) have been involved in malaria research in Apac District. Studies cover both molecular and immunology based epidemiology. Lira Medical Centre (LMC) has been chosen as the trial site based on infrastructure, logistics and the standard health care it provides to the community and neighboring counties. The Centre itself is readily accessible by foot or ground vehicle to all perspective trial volunteers. It has an outpatient and inpatient facility, surgical theatres, has a clinical laboratory equipped to carry out basic laboratory assays (blood counts and serum chemistries), a pharmacy, and is staffed 24 hours/day and 7 days/week. At present, the Centre is the primary provider of medical care in Lira and neighboring towns since its establishment in 2005. As a private, non-governmental organization hospital the Centre provides care free-of-charge to the community for immunization, tuberculosis drugs, blood transfusion, ante-natal care and counseling, routine malaria check for pregnant women, mosquito bed nets and vitamins. Two separate site assessments from the CRO dated March 25-29, 2008 and Aug. 18-22, 2008 list in detail the infrastructure/facilities that attest to LMC as a suitable site for the trial.

This trial is expected to enhance the local capacity of the community in providing sustainable progress in malaria control. LMC will be further strengthened as the primary provider of medical care in Lira district. Additional training of staff to conduct the trial under Good Clinical Practice (GCP) guidelines will enhance the capacity of the Centre to deliver medical services in Lira and surrounding districts.

1.2 Rationale for Selection of the Vaccine Candidate

The malaria parasite has a complex life cycle. The intraerythrocytic cycle (blood stage) is responsible for the clinical symptoms familiar to us as malaria. Vaccines that target the blood stage inhibit growth of the parasites by antigen-specific antibodies. Proteins that are both accessible and essential to parasite development are targets. Unlike vaccines for pre-erythrocytic stages, blood stage vaccines are expected to either reduce the parasite load by preventing invasion of red blood cells or limit parasite replication/growth after invasion, and thus prevent clinical disease.

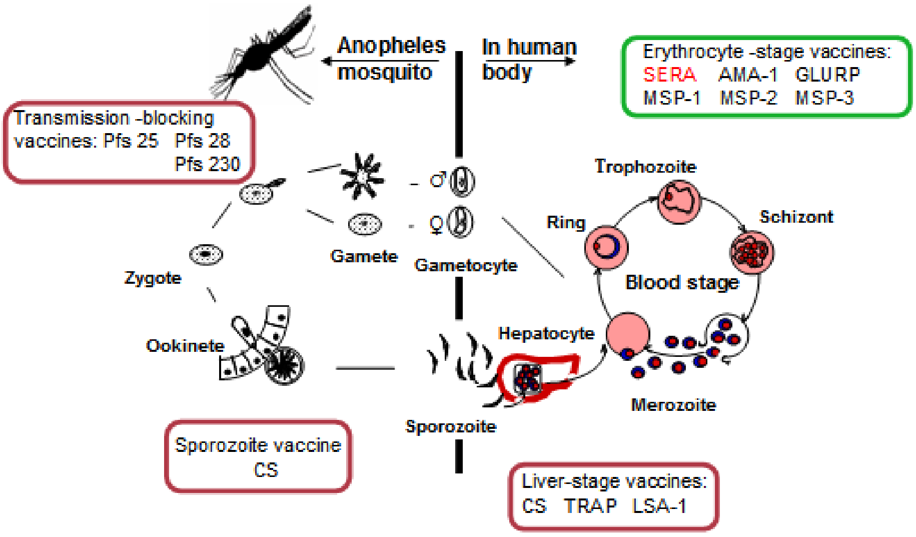

Plasmodium falciparum: life cycle, vaccine candidate antigens and target stages.

1.3 The Serine Repeat Antigen (SERA)

The serine repeat antigen belongs to a multigene family that is unique to *Plasmodium* (Aoki et al., 2002). All members are transcribed most actively at trophozoite and schizont stages, with the dominant expression of SERA5 (SERA). SERA protein has serine repeats in its N-terminal domain and is produced and accumulated in the parasitophorous vacuole in large amounts prior to schizont rupture (Fox and Bzik, 1994).

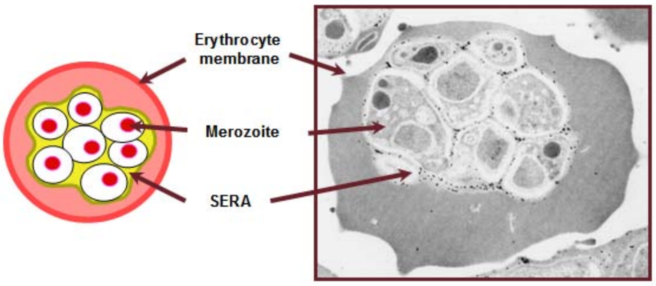

During merozoite release and reinvasion, SERA is proteolytically processed into a 47 kDa N-terminal (P47, marked I in the figure below), a 56 kDa central and an 18 kDa C-terminal (P56 and P18, II) (Debrant et al., 1992). P56 is further processed into two fragments with a size of 50 kDa and 6 kDa (P50 and P6, III). Fragments P47 and P18 are associated with merozoite surface whereas P50 is released (Li et al., 2002). Recombinant SE36 protein is identical to SE47' except that the serine repeats are removed from it.

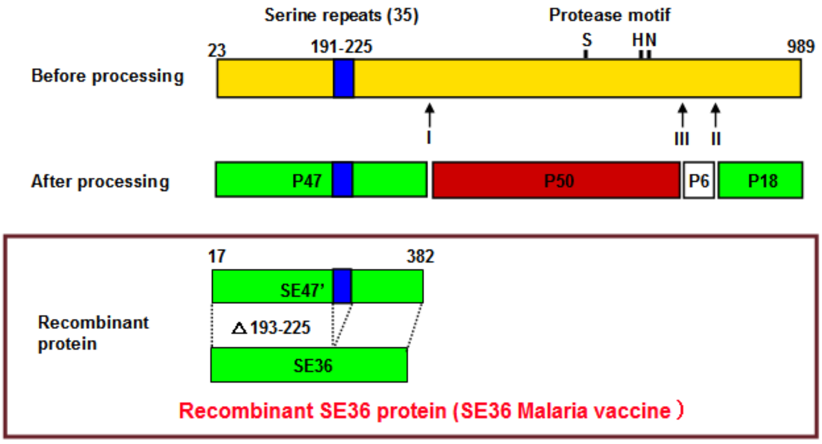

1.3.1 *Evidence of SERA as a vaccine candidate*

In vitro studies

Several lines of evidence suggest a possible role to promote merozoite egress: (1) SERA as a major parasitophorous vacuole protein during the schizont stage, (2) localization of P47 to merozoite surface, and (3) recombinant SE47' elicits specific antibodies that inhibit *in vitro* parasite growth (Pang et al., 1999).

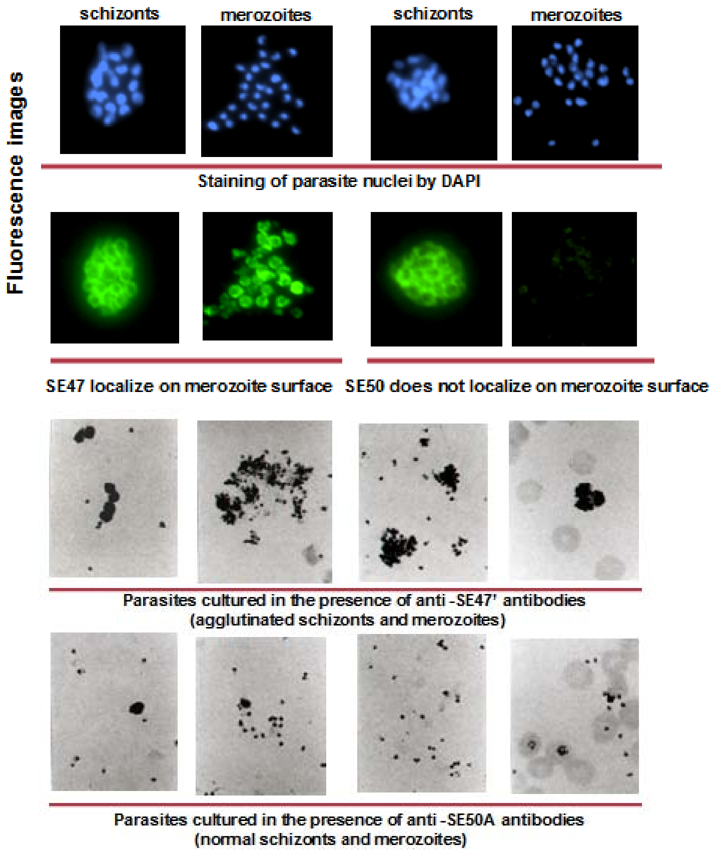

Immunoepidemiological data: uniqueness of SERA vs. other vaccine candidates

In the malaria-hyperendemic region of Apac District, Uganda, naturally induced antibody response to the N-terminal domain positively correlated with increased protective immunity in adults; and higher levels of IgG3 anti-SE47' were associated with the absence of fever and lower parasitemia in the peripheral blood of children aged under 15 years old (Okech et al., 2001). In addition, adult Ugandan serum with high antibody titer against SE47' inhibits parasite proliferation *in vitro*. This growth inhibition of naturally acquired antibodies to SE47' was dose-dependent.

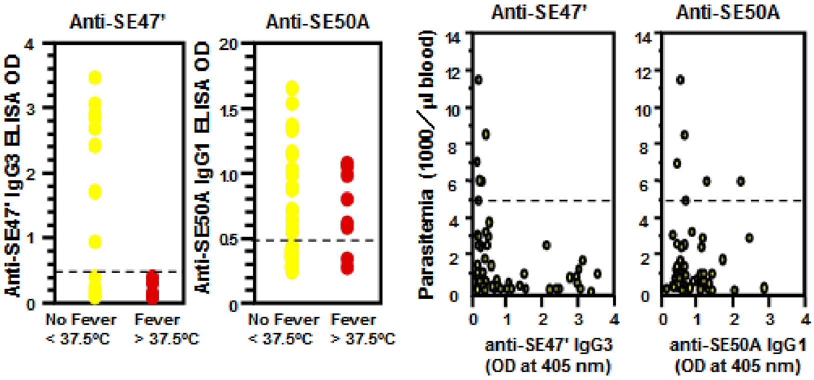

People with high antibody titer against SE47' does not have malaria fever nor high parasitemia.

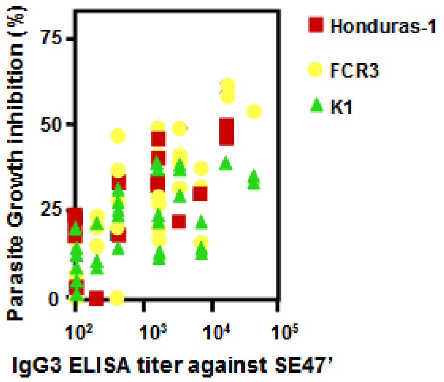

Ugandan serum with high antibody titer against SE47' inhibits the parasite proliferation in vitro

1.3.2 *From SE47' to SE36 Protein*

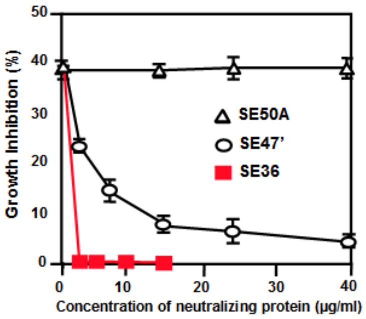

SE36 is superior to SE47'

Because SE47' protein is highly hydrophobic and difficult to purify, serine repeats were removed and SE36 protein with high hydrophilicity was constructed. After tuning the refolding process, SE36 protein could neutralize growth inhibition of Ugandan serum with lower protein concentration than SE47'. Thus, SE36 protein is superior to SE47'.

Likewise, in Uganda, higher levels of IgG anti-SE36 protein were associated with protection against severe malaria in children under 5 years old (Okech et al., 2006).

In about 100 residents from Solomon Islands, anti-SE36 protein antibody levels also correlate with lower parasitemia.

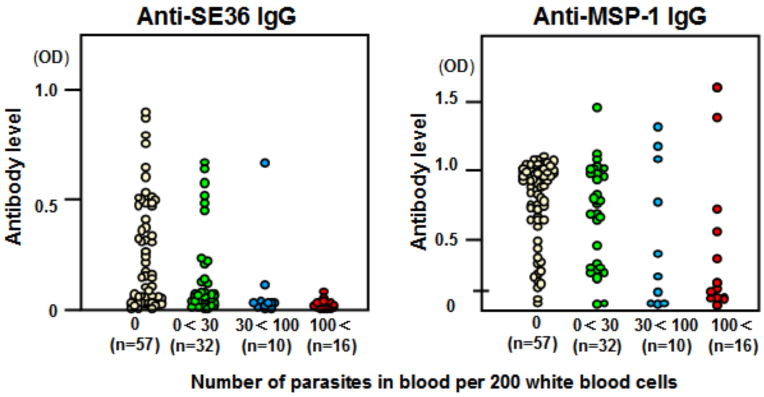

Correlation of anti-SE36 antibody levels with parasitemia in residents from the Solomon Islands

Pooled high antibody titer sera inhibited parasite growth in a dose-dependent manner. When the serum pool was reacted with SE36 protein to neutralize specific antibodies, the level of parasite growth inhibition was reduced to levels equivalent to low anti-SE36 protein titer group. These findings suggest that anti-SE36 protein antibodies are directly involved in the parasite growth inhibition.

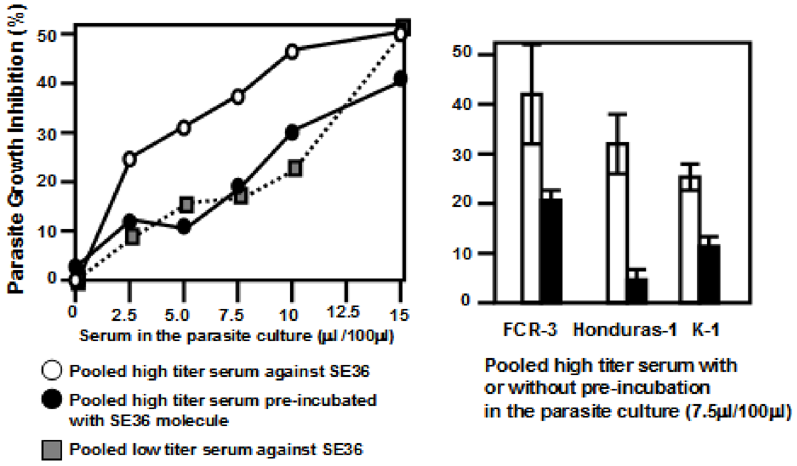

Pre-dominant part of protective malaria immunity is dependent on anti-SERA N-terminal domain antibodies

From these evidences, it is reasonable to postulate that vaccination with SE36 protein may protect an individual from illness due to the blood stage infection by *P. falciparum*.

1.4 Investigational Product Description

1.4.1 Generic name: Lyophilized recombinant precipitated tropical malaria vaccine (BK-SE36)

1.4.2 Description: BK-SE36 is a lyophilized preparation of the N-terminal domain of *P. falciparum* SERA. The protein is produced in and purified from *Escherichia* (*E.*) *coli* bacteria at the facilities of the Kanonji Institute, The Research Foundation for Microbial Diseases of Osaka University. The purified stock solution of SE36 protein was adjuvanted with aluminum hydroxide gel and the precipitated stock solution was then divided into several wells and lyophilized.

The protein content of BK-SE36 is approximately 100 μg/ml.
The aluminum content of BK-SE36 is approximately 1,000 μg/ml.

This candidate vaccine is intended to limit malaria morbidity and mortality, and possibly infection, by stimulating host immune responses against the serine repeat antigen of *P. falciparum*.

1.4.3 Active ingredient: Supplied as white, lyophilized powder, when reconstituted with 1.3 ml of the supplied diluent, the solution contains 100 μg of *P. falciparum* SE36 protein

Excipients: ≤ 2.85 mg dibasic sodium phosphate hydrate (Japanese Pharmacopoeia); ≤ 0.415 mg sodium dihydrogen phosphate dihydrate (Japanese Pharmaceutical Excipients); ≤ 8.08 mg sodium chloride (Japanese Pharmacopoeia); and 2.89 mg aluminum hydroxide gel (1.0 mg as aluminum) (Japan standard).

The priming volume that can be collected was established as 1 ml.

1.4.4 Preclinical studies with BK-SE36:

***In vitro* test**

Ugandan serum with high antibody titer against SE47' inhibits parasite proliferation. When sera were mixed with SE36 protein and heated, the inhibitory effect disappeared via an antibody-neutralizing response. This suggests that a human-specific antibody, reacting to SE36 protein, directly contributes to the inhibitory effect on parasite proliferation.

Immunological tests in animals

Clinical grade lots of BK-SE36 have been administered to mice, chimpanzees and squirrel monkeys. Excellent immune response without any overt signs of ill health or unusual responses have been observed:

Immunological test using mice

In mice, subcutaneous administration of BK-SE36 increased the anti-SE36 protein antibody titer in a dose-dependent manner.

Immunological test using chimpanzees

When BK-SE36 was administered to three chimpanzees thrice (weeks 0, 4, and 12) at 450, 50, and 10 μg per dose, there was a dose-dependent increase in the anti-SE36 protein-specific ELISA antibody titer. Two doses of 450 and 50 μg; and 3 doses of 10 μg resulted in high anti-SE36 protein titers. The titers remained high for more than 40 weeks, and boost administration after more than 2 years increased the titer to the maximum level within 1 week. Thus, SE36 protein is highly immunogenic in chimpanzees.

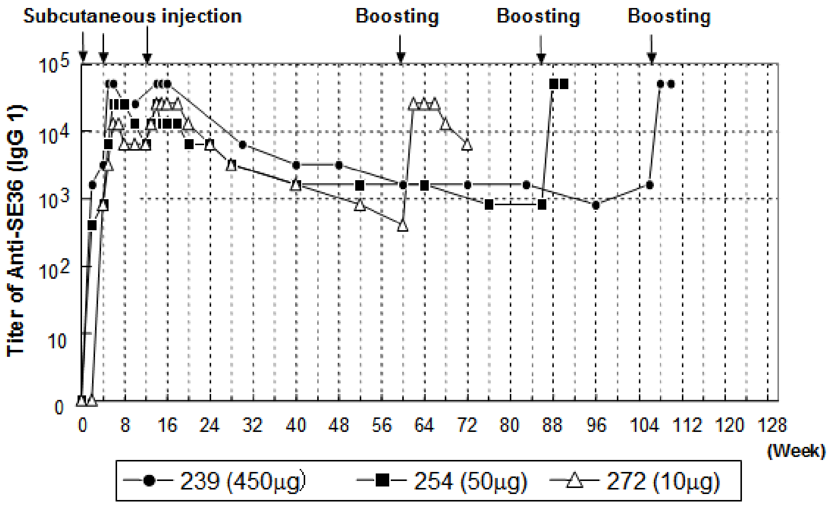

Change of titers of anti-SE36 antibodies in 3 chimpanzees post vaccination

Immunogenicity test of SE36 in chimpanzees by using alum as an adjuvant

Immunological test using squirrel monkeys: Efficacy in challenge infection

An immunological test using squirrel monkeys showed an increase in the anti-SE36 protein antibody titer in the BK-SE36 treated group. Squirrel monkeys subcutaneously immunized twice with 50 μg BK-SE36 in a 2-week interval, were challenged with *P. falciparum*-infected red blood cells 3 weeks after the final immunization. The parasitemia in the blood after challenge infection in the BK-SE36-treated group was markedly lower than that in the control group, showing that clinical formulation of SE36 protein has malaria vaccine efficacy.

Efficacy trial of SE36 vaccine (50μg/Alum) produced under GMP

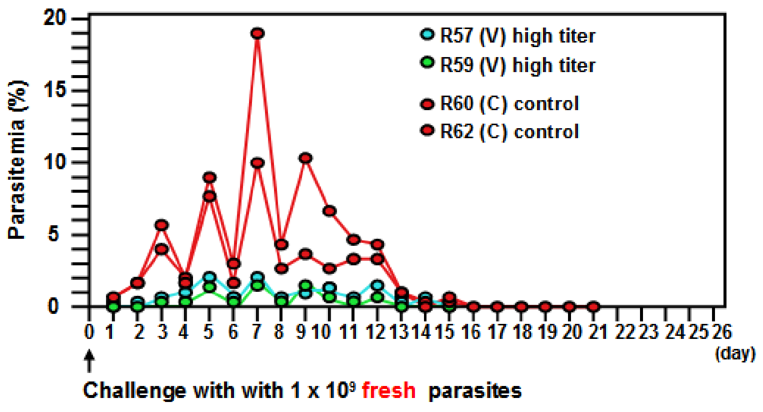

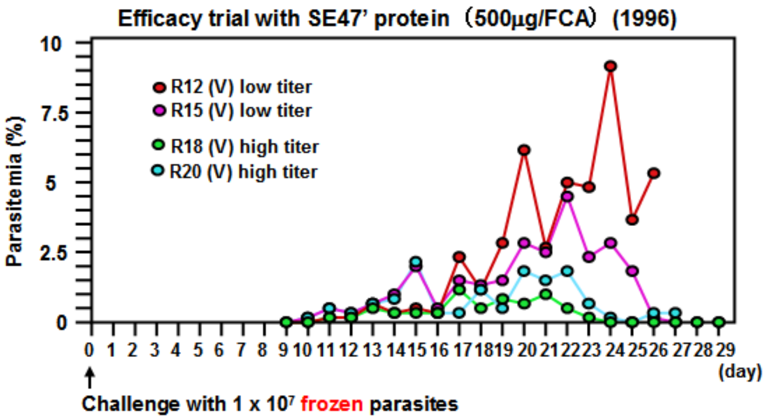

Clinical SE36 formulation has a malaria vaccine efficacy

Furthermore, challenge infection with *Plasmodium*-infected erythrocytes increased the anti-SE36 protein antibody titer in the BK-SE36 treated group. In the control group, this challenge inoculation-related production of anti-SE36 protein antibody was not observed.

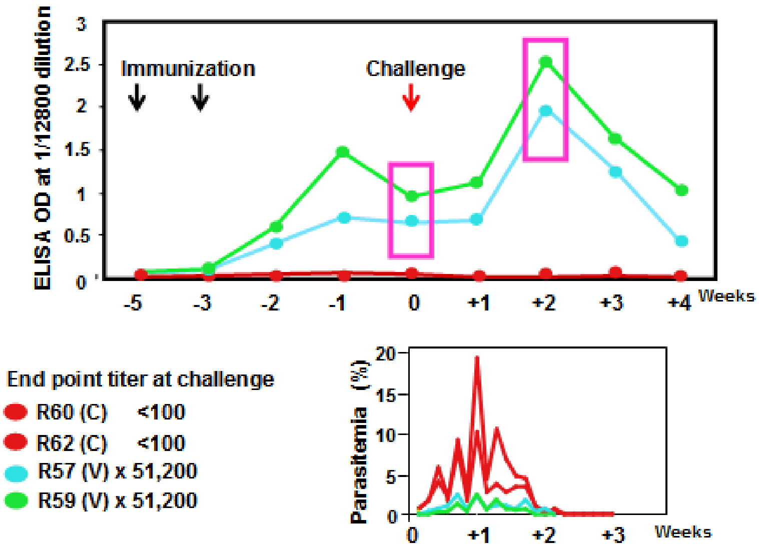

Vaccine induced IgG is boosted by the parasite infection

1.4.5 *Toxicology studies with BK-SE36*

BK-SE36 has undergone and passed General Safety Tests in rat, guinea pigs and dogs; irritability test in rabbits; genetic toxicity test in bacteria and mouse lymphoma cells; and reproductive and developmental toxicity tests in rats. Brief descriptions for each of the tests are presented below. In primate studies, clinical, biochemical and hematological examinations revealed no abnormalities.

<u>Pharmacological safety test</u>

There were no observed abnormalities when BK-SE36 at dose 10 times higher than the estimated clinical dose was subcutaneously administered to rats.

Table 1. Summary of the pharmacological safety tests

Test	*Species*	*Route and frequency of administration*	*Dose*	*Results*
Kidney function	Rat (*♂ 6/group x2)*	Subcutaneous, once	0.7 ml/kg	No influence
Respiratory function	Rat (*♂ 8/group x2)*	Subcutaneous, once	0.7 ml/kg	No influence

Test for ruling out abnormal toxic effects

In accordance with the general test methods described in the criteria for biological preparations, abnormal toxic effects using guinea pigs were monitored (♂3/group; ip, 5 ml). There were no abnormalities, except for a temporary weight loss and swelling in the area of administration where a white solid material due to aluminum (as adjuvant) was observed.

Toxicity test

Toxicity tests of: (1) single-dose subcutaneous administration of BK-SE36, at doses 10 and 100 times higher than the estimated clinical doses; (2) 4-week repeated-dose subcutaneous administration of BK-SE36 at a dose 10 times higher than the estimated clinical dose; (3) topical stimulation test involving intramuscular administration of BK-SE36 at 1.0 ml; (4) genetic toxicity test; and (5) reproductive and developmental toxicity tests showed no abnormalities (Table 2).

Table 2. Summary of the different toxicity tests.

Test	*Species*	*Route and frequency of administration*	*Dose*	*Results*
Single-dose administration	Rat (6-week old) ♂5, ♀5/ group (3 groups)	Subcutaneous, once	0.7 ml, 7.0 ml/kg (control group: 7.0 ml/kg saline)	No systemic toxicity except for excessive granulation at administration site (14-days observation period prior to autopsy)[1]
	Dog (7-8 month old) ♂2/ group (3 groups)	Subcutaneous, once	0.7 ml, 7.0 ml/kg (control group: 7.0 ml/kg saline)	No systemic toxicity. Transient panting, an increase in the pulse rate, and salivation were observed after administration at

					7.0 ml/kg group. (14-days observation period prior to autopsy)[2]
Repeat administration		Rat (6-week old) ♂10, ♀10/ grp (2 groups)	Subcutaneous, 4 times (once per week for 4 weeks)	0.7 ml/kg (control group: 0.7 ml/kg saline)	No systemic toxicity except for excessive granulation at administration site (28-days observation period prior to autopsy)[3]
Topical stimulation (Local irritability test)		Rabbit (15-week old) ♂6/ group (5 groups)	Intramuscular, once	1.0 ml/site (control group: saline; positive reference group: 1.7 and 0.425 % w/v acetic acid solution)	Topical pungency (grade 2) stronger than physiological saline and weaker than 0.425% w/v acetic acid solution (2 and 7-days observation period prior to autopsy)[4]
Genetic toxicity	Reversion test	Bacteria (5 strains)	1,300 to 20 µl/plate, 6 doses		No mutagenicity
	Gene mutation test	Mouse lymphoma cell	100 µl/ml - serially diluted at a ratio of 2; 6 doses		No mutagenicity in several generations
Reproductive and developmental toxicity test		Male rat	Subcutaneous, 4 times (twice before mating; on the day of mating; and 7 days after mating)	0.7 ml/kg	No influence
		Female rat	Subcutaneous, 4 times (twice before mating and twice in early pregnancy)	0.7 ml/kg	No influence

[1]On autopsy, dose-associated formation of subcutaneous ash gray or pale yellow-yellowish white nodes was noted at BK-SE36 administration sites. Histologically, granulations were observed as foreign body granulomas. Granulomatous, local, inflammatory reactions have also been reported for aluminum-based adjuvant (Goto and Akama, 1984).
[2]On autopsy, formation of subcutaneous ash gray or pale yellow-brown nodes was noted at the BK-SE36 administration site in one animal in the 0.7 ml/kg group and 2 animals in the 7 ml/kg group. Histologically, these were foreign body granulomas.

[3]On autopsy, a subcutaneous ash gray node was formed at the administration site in all males and females in the BK-SE36 treatment group. Histologically, these were foreign body granulomas that remitted with time.
[4]On assessment of BK-SE36 following the “Assessment Criteria based on the Test Methods Concerning Local Impairments by Administrations (draft; Ministry of Health and Welfare, 1979, 1984), irritation was grade-2 local impairment, similar to that caused by aluminum hydroxide gel.

Overall, subcutaneous ash gray or pale yellowish brown nodes were formed in the injected sites in the single-dose and repeated-dose toxicity tests, and these were histologically confirmed to be foreign body granulomas.

On the local irritability test, the color changes to pale red-reddish and pale yellowish white 2 days after administration. Histologically, moderate eosinophilic substances were present at a moderate level, and very mild to mild muscle fiber degeneration/necrosis, inflammatory cell infiltration, and hemorrhage were noted. Seven days after administration, the level of eosinophilic substances slightly decreased, and moderate foreign body giant cell infiltration and very mild muscle fiber regeneration were noted. Administration with aluminum hydroxide gel alone showed slightly narrower area of local changes around the administration site on both second and seventh day after administration, but the histological degree of impairments was similar. The area impaired using 0.425%, w/v acetic acid solution was similar 2 days after administration, but it became wider 7 days after administration. In the 1.7%, w/v acetic acid treatment group, the impaired area was wider than that in the BK-SE36 treatment group on both days.

Test in chimpanzees

When chimpanzees were subcutaneously immunized with BK-SE36 at 450 μg dose, no abnormality was noted in the general condition during the trial period, nor were there abnormalities in hematology or blood chemistry before or after administration.

1.5 Clinical Experience with BK-SE36

A Phase 1a clinical trial of BK-SE36 was undertaken by BIKEN and RIMD, Osaka University in 2005. The trial protocol (BK-SE36/001) was reviewed by the Institutional Review Boards of BIKEN and Osaka University and approval to conduct the trial received from the Ministry of Health, Labor and Welfare (MOHLW, Japan). The MOHLW designated the Tsukuba Clinical Pharmacology Center KAN-NONDAI Clinic in Tsukuba as the clinical trial site. The Institute has more than 10 years of GCP trial experience, with about 50 completed clinical studies. A CRO, Fuji Biomedix Co., Ltd., was contracted to manage aspects of data management, statistical analysis and reporting. A World Health Organization (WHO) team conducted one site visit to observe the conduct of the trial.

The trial was a randomized, single-blind, placebo controlled (physiological saline) study in healthy adults with no history of malaria exposure (malaria-naïve). Two doses (50 μg and 100 μg) were selected for clinical testing based on data generated from preclinical studies.

Each dose was subcutaneously administered on Days 0, 21 and 42. Administration in Group 2 (100 μg) started 14 days after safety assessment on Group 1(50 μg), first administration. Actual administration schedules were as follows:

3 administrations for low dose arm (50 μg) on Jan 26, Feb 16 and Mar 9. [Group 1]

3 administrations for high dose arm (100 μg) on Feb 14, Mar 7 and Mar 28. [Group 2]

Screening and selection were conducted where 46 were initially screened for Group 1, and 20 who met the inclusion/exclusion criteria were enrolled after obtaining informed consent and randomized to receive either test vaccine (15) or placebo (5). The placebo group received saline administration. Similarly, 38 were screened for Group 2, and 20 were enrolled and randomized. Subjects' age ranged between 20 to 34 years.

Table 3. Demographic characteristics of each cohort.

	Control group (Placebo)	*Group 1 (50μg)*	*Group 2 (100μg)*
Safety assessment	*n= 10*	*n=15*	*n=15*
Mean age (yrs)	22.6 ± 2.6	25.6 ± 4.3	24.5 ± 4.1
Mean height (cm)	171.56 ± 5.03	170.23 ± 3.68	172.61 ± 4.46
Mean body weight (kg)	64.52 ± 5.26	63.61 ± 5.39	61.87 ± 4.59
Efficacy assessment	*n= 10*	*n=15*	*n=14*
Mean age (yrs)	22.6 ± 2.6	25.6 ± 4.3	24.6 ± 4.2
Mean height (cm)	171.56 ± 5.03	170.23 ± 3.68	172.70 ± 4.61
Mean body weight (kg)	64.52 ± 5.26	63.61 ± 5.39	62.03 ± 4.72

The primary endpoint was for safety, the second was for immunogenicity. After the trial, subjects were followed-up for one year. For the collection of safety data, subjects visited the clinic at the baseline visit day, the day of each administration, one day and 2 weeks post administration. Each visit had a consultation, physical examination and laboratory tests, with the following observations recorded:

(a) Vital signs and physical examination

Body weight, blood pressure (systolic/diastolic), pulse rate, body temperature, and electrocardiograms were measured in the supine position at rest (a) before each administration; (b) 1 hour and 24 hours (except body weight); (c) 14 days after administration.

(b) Laboratory tests

13 ml of blood and 10 ml of urine (mid-stream urine) were collected (a) before each administration; (b) 24 hours, (c) 14 days after administration for the following tests:

Hematology

Leukocyte count, erythrocyte count, hemoglobin, hematocrit, platelet count, differential blood counts

Blood chemistry

Total protein, albumin, A/G, total bilirubin, AST, ALT, AL-P, LDH, cholinesterase, γ-GTP, LAP, serum amylase, total

cholesterol, neutral fat, uric acid, urea nitrogen, creatinine, Na, Cl, K, Ca, glucose, CRP

Urinalysis

Qualitative glucose, qualitative protein, urobilinogen, bilirubin, specific gravity, pH, ketone body, urinary occult blood reaction

Measurement of the antibody titer

No significant safety issues have been identified after three times administration of either of the two doses. No serious adverse events (SAEs) were observed and all adverse events (AEs) were resolved. The predominant adverse event was erythema and induration at the administration site (Table 4). To a lesser degree fever and fatigue were observed as systemic symptoms. The events were all mild and remitted, suggesting that these were not clinically problematic. Longest onset of AE (induration) was at 43 days after the third administration. There was one subject that had the longest AE (induration), which was observed 4 days post administration and resolved 60 days after, without therapy or limitation of daily activity. No side effects occurred in the control group. During the trial period, 19 (8 subjects, 80.0%), 80 (15 subjects, 100.0%), and 80 (14 subjects, 93.3%) clinical episodes were recorded for the control, Group 1 and Group 2, respectively. BK-SE36 was safe.

Table 4. Summary of adverse events. There were no significant differences among the proportion of volunteers with an injection site reaction of any severity between dose groups.

	Group1 (50mg)	*Group2 (100mg)*
Number of subjects (n)	*15*	*15*
Fatigue	*2*	*2*
Fever	*2*	*4*
Administration site reactions:		
Erythema	*14*	*10*
Induration	*15*	*10*
Itchiness	*0*	*4*
Pain	*0*	*1*
Swelling	*2*	*0*

Summary of antibody induction against SE36 protein:

Antibody prevalence. No subject was positive for anti-SE36 protein before the first administration. For Group 1 and Group 2, before the second administration, no subject (0/15, 0.0%) and 3 of the 14 subjects (21.4%) were positive, respectively. Before the third administration, 14 of the 15 subjects (93.3%) and all subjects (14/14, 100.0%) were positive, respectively. On post administration examination, all subjects (15/15, 100.0%; 14/14, 100.0%) were positive. Thus, sero-conversion rate was 100%. There were no significant differences in the antibody prevalence at any time point between the 2 groups (Fisher's exact test). Of note, antibody prevalence reached 100% in Group 1 after the third administration; but in Group 2, 100% sero-conversion was reached before the third administration.

Antibody titer. When changes in the antibody titer on post administration was compared with the value obtained before third administration, within Group 1, there was a significant difference (Wilcoxon signed rank test, p=0.0002); however, there was no significant difference within Group 2 subjects (Wilcoxon signed rank test, p=0.3575). Between groups (50 μg and 100 μg), there were significant differences in the antibody titer before the third administration and on post administration examination (Wilcoxon matched pairs test, p=0.0001 each). In Group 1 (50 μg), antibody titer rose after the second and third administration. In Group 2 (100 μg), no significant change was observed after third administration. The antibody titer in Group 2 was higher than in Group 1 before the third, and on post administration examination.

Based on the antibody prevalence and titer, 3 and 2 administrations may be necessary for BK-SE36 at doses of 50 and 100μg, respectively.

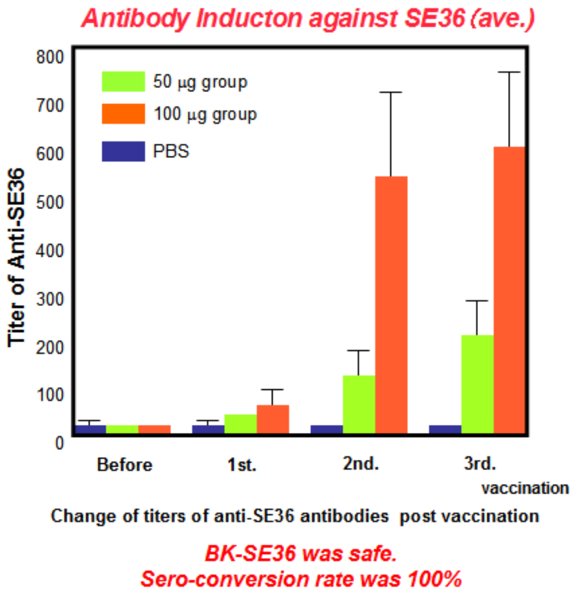

1.6 Clinical Experience with Other Alum-based Adjuvant and Possible Adverse Reactions

Aluminum hydroxide gel is a proven vaccine adjuvant and is used with existing precipitated vaccines (precipitated pertussis combined with diphtheria and tetanus vaccine, precipitated diphtheria and tetanus toxoid, precipitated tetanus toxoid, precipitated diphtheria toxoid for adults, and precipitated hepatitis B vaccine). Based on toxicity studies of BK-SE36 and findings of above precipitated vaccines, BK-SE36 may develop local reactions, such as flare, swelling, blister, pain, induration (hardening of normally soft tissue), pruritus (itchiness), and hot feeling. In existing vaccines, flare, swelling, blister, and pain after administration are transient, and usually disappear within 2 to 3 days, but induration may remain 1 to 2 weeks to 1 month. In Tetanus toxoid (Cody et al., 1981) and Recombivax hepatitis B (Merck, 2003) vaccines, local reactions such as pain, tenderness and swelling were experienced between 7.6% and 17.0% of volunteers in studies that included over 1,200 healthy adults. Fever is seen in 3.2% to 9.3%, headache in 4.1%, and other systemic symptoms such as fatigue, malaise, nausea, and diarrhea at lower frequencies.

Serious adverse events, like shock/anaphylaxis-like symptoms (urticaria, dyspnea, and vascular edema) rarely occur (less than 0.1%). Urticaria (raised red skin rashes) has been reported in 0.1% of individuals administered with Recombivax HB®. Precipitated pertussis combined with diphtheria and tetanus vaccine may cause acute thrombocytopenic purpura (purpura, nasal and oral mucosal hemorrhage), about several days to 3 weeks after administration in about 1 in 10 million administered persons.

For neurological adverse events of immunization (Expert Rev Vaccines, 2007: 6: 863-869; http://www.medscape.com/viewarticle/565691_3), the administration of DTP vaccine to infants has been associated with screaming, persistent crying, convulsions, encephalopathy and hypotonic, hyporesponsive episodes, but these signs have generally been linked to the pertussis component and not to the adjuvant. During the last 15 years, macrophagic myofasciitis (clinical presentations include myalgias, arthralgias, fatigue and multiple sclerosis; inflammatory myopathies); has been reported with aluminum adjuvanted vaccines, although the association of histologic lesions and the clinical symptoms is still considered an unproven hypothesis.

1.7 Clinical Experience with Recombinant Protein Vaccines

Recombinant protein vaccines are at best less reactogenic and more potent (for example, anthrax vaccine and hepatitis B virus vaccine; Ulmer et al., 2006). Hypersensitivity reactions are expected to occur within the first 24 hours after administration, and other severe local or systemic reactions within 72 hours, and possibly up to 3 weeks post administration (Georgitis and Fasano, 2001). Based on a number of malaria candidate vaccines that have undergone human trials (Nosten et al., 1996; Stoute et al., 1997; Doherty et al., 1999; Saul et al., 1999; Ockenhouse et al., 2006; Thera et al., 2008), local reactions are spontaneously resolved within weeks (pain, redness, swelling and/or tenderness). Systemic reactions include low-grade fever, chills and malaise; and more often these reactions are resolved in 12 days without therapy or limitation of daily activity. Allergic reactions (e.g. urticaria) and anaphylaxis typically occur after 2-3 administrations at intervals of 1 month or more, when vaccines are formulated with Alhydrogel or QS-21 adjuvants, and with high antigen concentrations (200 μg to 2000 μg per dose).

<u>Adverse drug reactions described in package inserts of drugs in the same therapeutic class.</u>

There is no approved drug in the same therapeutic class.

1.8 Clinical Development Plan

This protocol outlines a proposed Phase 1b clinical trial of BK-SE36. This will be the first trial of the vaccine candidate in a malaria-exposed population. It is both important and appropriate to test the safety of BK-SE36 in a population that has been regularly exposed to malaria because this is the ultimate target population, and because in theory natural infection before and after administration could either increase or decrease the risks of the vaccine. If shown to be safe and immunogenic, further trials will be planned to assess safety and efficacy in children and adult.

The goal of BK-SE36 is to reduce the severity of the clinical manifestations of *P. falciparum* infection, and thereby reduce morbidity and mortality. The presence of

infection, as determined either by blood smear or parasite DNA detection, should not be seen, therefore, as evidence of vaccine failure.

1.9 Justification for Administration Route, Dose and Frequency

1.9.1 Route of administration

Preclinical and safety studies of BK-SE36 have been carried out using the subcutaneous route of administration. The subcutaneous administration has been considered the general administration route for vaccines. Also, this route causes relatively milder reactions at the administration site than intramuscular administrations when using alum based adjuvant in mice (Goto and Akama, 1984).

1.9.2 Dose and frequency of administration

Based on primate studies, using chimpanzee and squirrel monkey, the estimated maximum clinical dose is 0.5 ml (50 μg) and 1 ml (100 μg), respectively, for children less than 3 years and above 3 years. Subcutaneous, 3 times administration with 21 day intervals was set in reference to the administration intervals of inactivated vaccines (within 1 to 4 weeks). The safety and immunogenicity of both doses and frequency of administration (3 times) were confirmed in the Phase 1a trial using healthy male Japanese adults. In addition, immunogenicity results from Phase 1a supports that 2 administrations can achieve 100% sero-conversion (*e.g.* using 100 μg dose, no significant change in the antibody titer was observed after the second and third administration). In this Phase 1b trial, since a long term strategy to use the minimum dose interval is more amenable for subject compliance, two administrations of BK-SE36 is proposed.

1.10 Rationale for Physiological Saline as Placebo

A control group is particularly useful in Phase 1 trials since background immunity and natural exposure to malaria may make it difficult to interpret immunogenicity data. Thus, the use of a control group will exclude influences of factors, such as environmental factors, other than the test vaccine. Using physiological saline as placebo, that permits the same administration period, it is hoped that a comparison of immune responses will result in a clearer interpretation of serological results.

1.11 Rationale for Trial Design: Two Stages. Stepwise. Single blind.

The overall goal of the clinical development plan is to generate proof of concept data that BK-SE36 can impact disease caused by *P. falciparum* parasite, resulting to a reduction in severe and mild cases of malaria in child to adult. Thus prior to the efficacy trials, it is necessary to collect adequate adult and children safety data and begin defining an appropriate children dose. The age limit (6 to 40 y old) covers an adequate age group susceptible to malaria. The safety concern also takes into account that in endemic areas, the adult population can be grouped into sero-positive or sero-negative against the SE36 protein.

Although BK-SE36 has been proven to be safe in 30 adults in Japan, natural malaria transmission may affect both the safety and immunogenicity of the vaccine, therefore it is more ethical to perform this Phase 1b trial first in healthy adults who can give full, informed independent consent (21 to 40 y old). Assuming BK-SE36 will be proven safe from Stage 1, it will be necessary to assess the safety and immunogenicity of this test vaccine in generally healthy children/young adults who are representative of the population which are vulnerable to malaria infection.

Regarding blinding, the single blind method was selected as a primary concern for safety and well-being of subjects. Bias or prejudgment about the effects of the test vaccine in the reporting of adverse events is removed by blinding the subjects. By unblinding the investigators, however, they can make objective assessment of local administration site reactogenicity and adverse events. The investigators judgment is also critical, especially in Stage 1, since incidence and intensity of individual symptoms over 21 days post administration will be crucial for guiding the start of Stage 2. To ensure adequate unbiased assessment, different trial staff will undertake hematological and urinary examinations; and antibody titer measurements. A separate randomization code will also be generated for serum samples. This code will correspond to the order in which these serum samples will be assayed for antibody titer.

The target number of subjects is set at an appropriate number to detect any possible adverse event against the need to limit the number of subjects involved for safety purposes. The trial is not powered to detect differences between groups, other than very large differences in the incidence/occurrence of adverse events (Please refer to Section 11.4). Since this is a biological product, allergic reactions may occur as accessory reactions. The uneven number of subjects allocated to test vaccine and placebo was set in consideration of large individual variation in allergic reactions.

Further clinical trial design and schedule will be discussed following Stage 2 if the safety profile remains promising among BIKEN, RIMD and MBL.

2 OBJECTIVES

2.1 Stage 1, 21 to 40 y old

2.1.1 Primary objective

To assess the safety and reactogenicity of BK-SE36 subcutaneously administered 2 times (at a 21 ± 1 day interval) to healthy male and female adults, sero-negative and sero-positive against the SE36 protein.

2.1.2 Secondary objective

To examine changes in the immune response as a result of 2 times administration of BK-SE36 in healthy male and female adults sero-negative and sero-positive against the SE36 protein.

2.2 Stage 2, 6 to 20 y old

2.2.1 Primary objective

To assess the safety and reactogenicity of 50 µg and 100 µg BK-SE36 subcutaneously administered 2 times (at a 21 ± 1 day interval) to healthy malaria-exposed 6 to 20 year olds.

2.2.2 Secondary objective

To examine changes in the immune response as a result of 2 times administration of 50 µg and 100 µg BK-SE36 in healthy malaria-exposed 6 to 20 year olds.

3 TRIAL DESIGN

OVERVIEW

- Phase 1b, 2 stages, stepwise:
  Stage 1: 21 to 40 y old; 2 cohorts; 1 dose (100 µg SE36)
  Sero-negative, male: BK-SE36, placebo
  female: BK-SE36, placebo
  Sero-positive, male: BK-SE36, placebo
  female: BK-SE36, placebo

  Stage 2: 6 to 20 y old; 3 cohorts; 2 doses (50 and 100 µg SE36)
  6 to 10 y old: 50 µg SE36, 100 µg SE36,
  0.5 ml placebo, 1.0 ml placebo;
  11 to 15 y old: 50 µg SE36, 100 µg SE36,
  0.5 ml placebo, 1.0 ml placebo;
  16 to 20 y old: 50 µg SE36, 100 µg SE36,
  0.5 ml placebo, 1.0 ml placebo
- One trial site: Lira Medical Centre (LMC)
- Single blind, randomized, placebo (physiological saline) controlled
- Screening will be incremental and will be done within 30 days prior to first administration
- Administration schedule: Trial days 1 and 22
- Route: Subcutaneous administration (upper arm)
- Stage 2 will only proceed after a safety assessment of Stage 1 (go or no-go)
- Total number of clinic visit per subject: 9 (excluding screening)
- Active surveillance (continuous/scheduled clinic visits) for adverse events: approximately 6 weeks per subject (corresponds to the total number of clinic visits; excluding screening)
- Post-trial follow-up (passive surveillance or voluntary clinic visits): 40 days after visit 10

- Follow-up of adverse events (AEs) and serious adverse events (SAEs) until resolution
- Overview of trial stages from regulatory approval to post trial follow-up is presented in the Appendix, Section 18.2, p. 91.

3.1 Primary and Secondary Endpoints

3.1.1 Primary endpoint

The safety of BK-SE36 will be assessed by the presence or absence of adverse events. This information will be gathered from patient symptoms, vital signs and laboratory test results.

Self assessment of local and systemic reactions, as well as, assessment by investigator - 60 minutes after, and 1 day, 7, 14 and 21 days after each administration.

Occurrence of clinically significant hematological and/or biochemical abnormalities (either by complete laboratory tests or urinalysis only) will be monitored 21 days after each administration.

3.1.2 Secondary endpoint

Changes in the anti-*P. falciparum* (SE36 protein) antibody titer. Blood samplings will be done before the first and second administration; and 21 days post administration (final visit).

note: Assays to measure the biological activity of induced antibodies (GIA [growth inhibition assay], ADCI [antibody-dependent cell-mediated inhibition assay] and epitope mapping [identification of minimum molecular structure for recognition of the immune system]) will be treated as a research output of this trial. The results of these assays will not be considered for the secondary endpoints, nor encoded into the final database. Tests will be conducted and summarized separately in Japan (BIKEN and RIMD) and France (Pastuer Institut) and are mentioned in this protocol solely to seek approval from pertinent IRC(s) and regulatory authorities to conduct these assays.

3.2 Trial Design. Phase 1b, Stage 1 and 2. Single-blind, randomized.

The overall trial design will be randomized, single-blind. There will be 2 stages: Stage 1 will be on healthy subjects, serologically negative and positive against the SE36 protein, age 21 to 40 years. Stage 2 will be on healthy subjects, age 6 to 20 years inclusive. In both stages, subjects who meet the screening criteria will be grouped to cohorts, randomized to treatment allocations and will be followed for 43 days. Subjects will not be informed of their treatment regimen. Evaluations for adverse events will be performed on trial days 1, 2, 8, 15, 22, 23, 29, 36, and 43 (and any unscheduled day). Administration will be performed on days 1 and 22.

3.2.1 Trial size, gender and age.

<u>Stage 1, 21 to 40 y old: 56 subjects:</u>

	BK-SE36		Placebo	
	Male	Female	Male	Female
Sero-negative	9	9	5	5
Sero-positive	9	9	5	5

In Stage 1, eligible volunteers will be grouped into either sero-negative or sero-positive cohorts. Sero-negative and -positive against the SE36 protein would be decided based on anti-SE36 protein ELISA assays done during screening. Cohorts will be further divided into groups of males and females, randomized to receive either 2 administrations of BK-SE36 (1 ml) or physiological saline (1 ml).

Stage 1 Flow diagram:

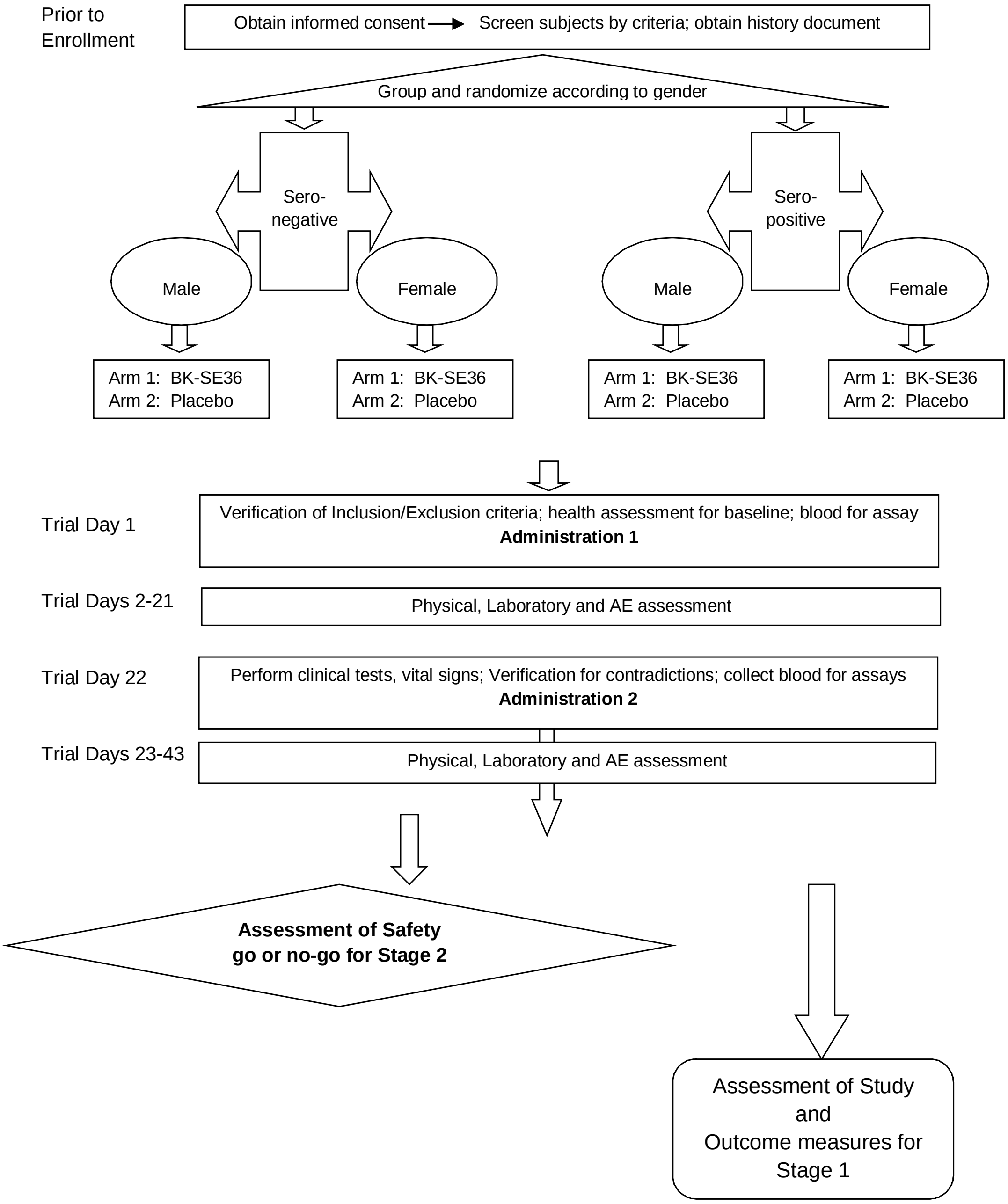

Safety assessment: 30±5 days after the second administration in Stage 1, the decision to proceed to Stage 2 will take into consideration AEs and SAEs observed until 21 days post administration (please refer to Section 13.3.2, p. 83 for go or no-go).

Stage 2, 6 to 20 y old: 84 subjects:

	BK-SE36		Placebo	
	50µg	100µg	0.5 ml	1.0 ml
6 to 10y	11	11	3	3
11 to 15y	11	11	3	3
16 to 20y	11	11	3	3

In Stage 2, eligible volunteers will be divided into three cohorts (according to age): 6 to 10 y, 11 to 15 y, 16 to 20 y. Subjects in each cohort will receive either BK-SE36 at ½ (0.5 ml) or full dose (1 ml); or physiological saline (0.5 or 1.0 ml). Care will be taken that not a single cohort would be slanted towards one gender. Each arm/group will receive 2 administrations.

Stage 2 Flow diagram:

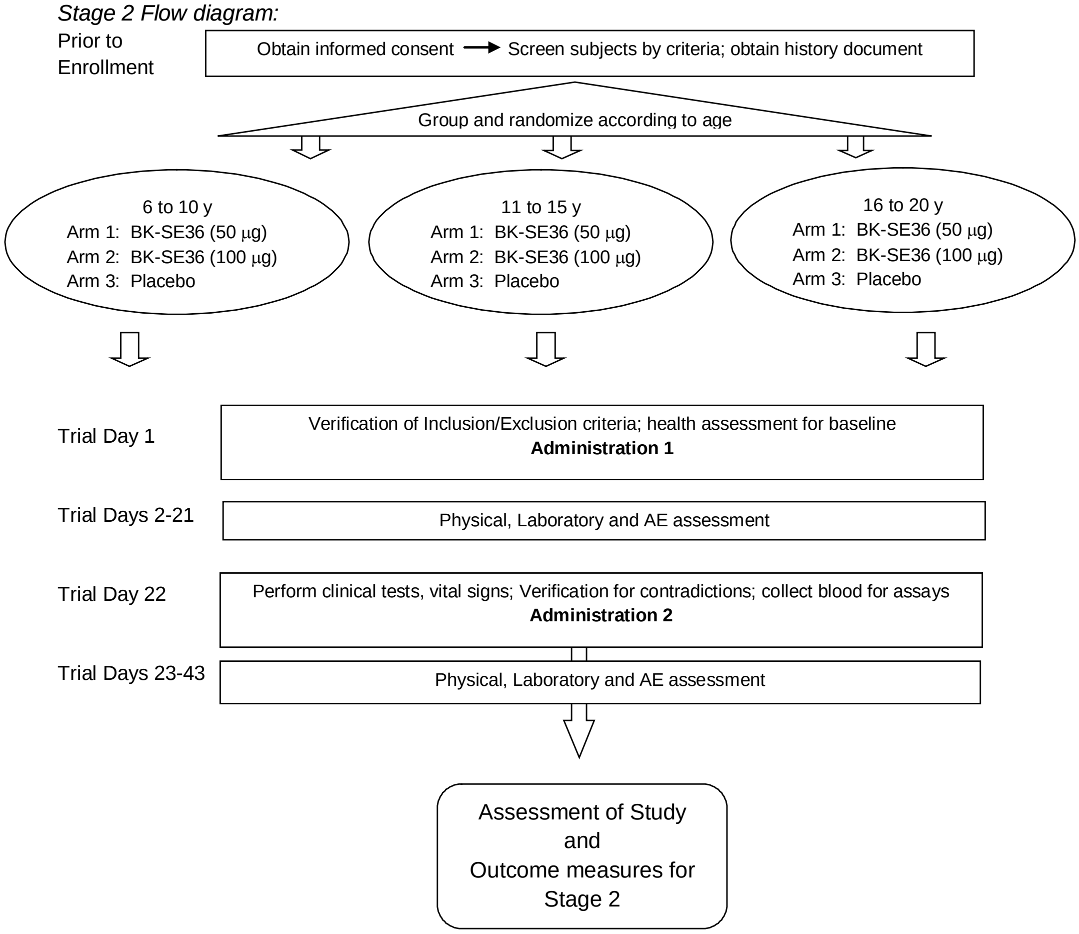

3.2.2 Randomization

Randomization to treatment allocations will be done using a computer-generated randomization list. Eligible volunteers will be assigned a unique “Subject code.” The randomization list will contain codes linking a subject code to a treatment number/assignment. The lists will be computer generated in blocks (per cohort) to ensure balance treatment allocation.

Sealed copies of the randomization lists and documentation of the procedure used to generate the lists will be provided to the investigators. Subject codes will be assigned in the order in which subjects provide written informed consent to be enrolled in the trial. The investigational product and dose that are assigned during the first administration will be maintained for the second administration. A trial staff will confirm that investigational products are administered following the randomization list. Access to the list will be exclusively limited to the principal investigator, and designated trial staff(s) at LMC.

A separate set of randomization numbers will also be generated for serum samples. The code numbers will be assigned when serum samples are transported from LMC to MBL. (Alternatively, randomization can be done when serum samples will be shipped from MBL to BIKEN).

3.2.3 Blinding

BK-SE36 (white, lyophilized powder) and diluent (water for administration, Japanese Pharmacopoeia) is packaged in separate vials placed in one container or ‘patient kit’. When reconstituted, BK-SE36 will appear milky white and opaque. The placebo, physiological saline, packed as received from the manufacturer (Otsuka), is colorless. Thus after preparation, the test vaccine and placebo would not have the same appearance, and blinding of the subjects will not be possible. Because of this, the investigational products preparation area and the administration area will be physically separated. Reconstitution and preparation of investigational products are detailed in the SOP [*General Events from Informed Consent to Post Administration*]. A designated trial staff will be exclusively dedicated to preparation and have with him/her an assistant to ensure that the proper investigational product is delivered for each subject. Both staff will refer and confirm that the randomization code of the subject matches the investigational product to be given. Administration can be carried out simultaneously in 2 separated compartments adjacent to the investigational product preparation area and are best done out of view of anyone other than the investigator and subject, so that each subject sees only the syringe he or she is injected with and never sees other subjects’ syringe. Between administration days, the randomization list will be stored in a locked cabinet.

Clinical technicians who will be involved in laboratory tests (hematology, blood chemistry, malaria smear, and urinalysis) will not be blinded to treatment group assignments, since once a sample is submitted for tests, knowing which investigational product was administered is unlikely to influence any outcome.

To ensure further unbiased assessment, serum samples collected for ELISA on trial days 1, 22 and 43 will be randomized, as described above. This guarantees that differences in preparation and measurements at different assay days are unlikely to have

any significant effect on ELISA titer measurements, particularly for pre and post-administration samples. Unblinding will only occur at the time of data entry.

4 TRIAL POPULATION

4.1 Inclusion Criteria

The standard and acceptable ranges of laboratory test values in healthy children and adults are set following the baseline ranges at LMC (for Stage 1) and those obtained from the clinical investigators (for Stage 2).

[Inclusion 1]: Stage 1, 21 to 40 y old

Healthy subjects are specified, and the inclusion criteria of malnutrition index and laboratory test values were included to reduce individual variation.

<1.1> Healthy adult. Ugandan males and females aged 21 to 40 (age on informed consent);

<1.2> Those who do not suffer from severe malnutrition (defined as an adult whose weight-for-height is below -3 standard deviation or less than 70% of the median of the NCHS/WHO normalized reference values);

<1.3> Those who are able to agree, comply with matters to be observed during participation in the trial, undergo consultation/examination, as described in this protocol, and report symptoms;

<1.4> Those who are considered to be eligible to participate in this trial based on screening:

<1.4.1> Vital signs and physical examination are within baseline range

<1.4.2> Hematology: Within 25% deviations from the upper and lower limits of the baseline range. The differential white blood count is not questioned when the white blood cell count is within the baseline range.

<1.4.3> Blood chemistry:

- AST, ALT, and creatinine: Within the baseline range
- Total bilirubin: Within 50% deviation from the upper limit.
- Serum electrolytes: Within the baseline range.
- Other blood chemistry items: Within 25% deviation from the upper and lower limits of the baseline range.

<1.4.4> Urinalysis: Within the normal range

[Inclusion criteria 2]: Stage 2, 6 to 20 y old

Healthy subjects are specified, and the inclusion criteria of malnutrition index and laboratory test values were included to reduce individual variation.

In this stage, there will be 3 different informed consent (IC) requirements:

IC for children aged 6 to 7 y will be solicited from parent(s)/guardian(s)
IC for ages 8 to 17 y will be solicited from child and parent(s)/guardian(s)
IC for ages 18 to 20 y will be solicited from the volunteers themselves

<2.1> Volunteers, irrespective of gender, aged 6 to 20 years (age on informed consent)

<2.2> Those who do not suffer from severe malnutrition, whose BMI is between 5th percentile to less than the 85th percentile for 6-19 y old; and between 18.5-25.0 for 20 y old;

<2.3> Those who can give affirmative agreement to participate in the trial. For children between 8 to 17 y, the child's assent takes precedence over the parent(s)/guardian(s) consent.

<2.4> Those who are able to agree, comply with matters to be observed during participation in the trial, undergo consultation/examination, as described in this protocol, and report symptoms;

<2.5> Those who are considered to be eligible to participate in this trial based on screening:

<2.5.1> Vital signs and physical examination are within baseline range

<2.5.2> Hematology: Within 25% deviations from the upper and lower limits of the baseline range. The differential white blood count is not questioned when the white blood cell count is within the baseline range.

<2.5.3> Blood chemistry:

- AST, ALT, and creatinine: Within the baseline range
- Total bilirubin: Within 50% deviation from the upper limit.
- Serum electrolytes: Within the baseline range
- Other blood chemistry items: Within 25% deviation from the upper and lower limits of the baseline range.

<2.5.4> Urinalysis: Within the normal range

4.2 Exclusion Criteria for both Stage 1 and 2

Any subject meeting any of the exclusion criteria at baseline will be excluded from trial participation.

<1> Persons with fever (37.5°C or higher) on administration of the test vaccine;

<2> Persons with a clear history of food/drug-related anaphylaxis;

<3> Females (adolescents/adults) who are pregnant or have a positive urine β-hCG on the day of, or prior to, administration;

<4> Females currently lactating or breast-feeding;

<5> Persons with acute or chronic cardiovascular, pulmonary, hepatic, renal, or neurological condition, which in the opinion of the investigator may increase the risk of the subject from participating in the trial;

<6> Persons with a history of fever within 2 days after preventive administration with other types of vaccine, or those in whom symptoms have suggested systemic allergy;

<7> Persons with a history of convulsion other than febrile convulsions in malaria in the past 6 months to 1 year

<8> Persons with any confirmed or suspected immunosuppressive or immunodeficient condition, including human immunodeficiency virus (HIV) infection. (No infectious disease testing will be conducted. HIV testing will not be done. Severe, suspected infectious diseases will be ruled out by investigators during physical examination/consultation, blood hematology/chemistry tests; although not conclusive of the causative agent.);

Additional oral confirmation: Subject informed the investigator that he/she has been tested positive for HIV/AIDS. (Information on the child's HIV status could be obtained from their parents or guardians.)

<9> Persons with a history or tentative diagnosis of drug allergy; especially to common drugs like penicillin, sulphonamides, etc.

<10> Persons with (history of) chronic alcohol consumption and/or illicit drug use;

<11> Persons who took any medication within 1 week before administration of this test vaccine (except for artemether/lumefantrine and dihydroartemisinin-piperaquine);

<12> Persons to whom any live vaccine was administered within 4 weeks before administration of this test vaccine, or inactivated vaccine/toxoid was administered within 1 week;

<13> Persons who participated in another trial within 4 months before administration of this test vaccine; or simultaneous participation in any other clinical trial;

<14> Persons in whom 200 ml of blood was collected (donation) within 1 month before administration of this test vaccine, or more than 400 ml of blood was collected within 3 months;

<15> Persons who have recently undergone blood transfusion in the last 3 months.

<16> Others who are not considered to be eligible by the investigator or those, whose medical condition would, in the opinion of the investigator, make the subject unsuitable for the trial.

[Justification for the exclusion criteria]

Behavioral and cognitive diseases may affect the ability of the volunteer to understand and cooperate with the protocol.

BK-SE36 has not yet been tested in pregnant women.

Persons with liver, renal and hematological diseases will be excluded as these may jeopardize the safety of the volunteer or would render them unable to comply with the protocol. Persons with clinical evidence of immunosuppressive disease will also be excluded but no testing for asymptomatic HIV infection will be done, based on the rationale that it is necessary to assess the safety and immunogenicity of this vaccine in generally healthy adults who are representative of the population from which they are drawn.

Those under different drug treatments will be excluded to rule out the influences of other drugs or vaccines on safety and immunogenicity assessment. Blood transfusion carries the risk of immunomodulation.

4.3 Treatments Potentially Interfering with Vaccine-induced Immunity

The following criteria will be checked at Visits 2, 6 (prior to each administration) and visit 10 (trial day 43). If any become applicable during the trial, it will be reported to the sponsor, and the sponsor will decide whether administration should be continued based on consultation with the principal investigator. In any case, the subject will be encouraged to remain in the safety evaluation for dose already received. A separate immunogenicity analysis may also be done that excludes these subjects.

<1> Use of investigational drug or vaccine other than the test vaccine during the trial.

<2> Administration of chronic (defined as more than 14 days) immunosuppressant(s) during the trial (pertains also to daily use of oral steroids and inhaled steroids; but daily use of topical steroids are allowed). These should be noted as concomitant medication(s).
<3> Occurrence of severe chronic disease (such as diabetes or tuberculosis) which might complicate interpretation of safety or immunogenicity data.
<4> Administration of immunoglobulins and/or other blood products during the trial period.

4.4 Contradictions to Administration

The following criteria will be checked prior to each administration and are contradictions to further administration. However, the subject will be encouraged to continue to participate in the scheduled trial visits for safety evaluation.

<1> Hypersensitivity reaction following administration of the test vaccine

- anaphylactic reactions
- significant Grade 3 or 4 reactions (including severe headache, severe pain, swelling, persistent high fever)
- Discontinuation of administration is appropriate based on the symptoms, frequency, and severity of an adverse event

<2> Pregnancy: positive urine β-hCG

4.5 Indications for Deferral of Administration

The following constitute grounds for temporary deferral of administration; if any of these events occurs at the time scheduled for administration, the subject may be administered at a later date, within the allowable time interval (1 week), or may be permanently discontinued from further administration at the discretion of the investigators. When an episode meets one of the criteria, it will be reported to the sponsor.

<1> Axillary temperature ≥37.5°C or evidence of clinical malaria at the time of administration will warrant deferral of administration until fever and symptoms resolve.

<2> Any other condition in the opinion of the investigator that poses a threat to the subject if administered with test vaccine or that may complicate interpretation of the safety following administration.

The subject will be followed until resolution of the event (for clinical malaria see *Section 6.2*). No further administration will be performed if the subject does not recover (axillary temperature <37.5°C and/or lack of symptoms) within 7 days of the originally scheduled administration date. The subject, however, will be encouraged to remain in the safety evaluation for the duration of the trial.

If a subject meets any of the above criteria for deferral on the day of the first administration, the investigator may elect to replace the subject (see also *Section 10.2.3*).

5 TRIAL INTERVENTION: INVESTIGATIONAL PRODUCTS

Sufficient bulk production for clinical trial is the responsibility of BIKEN (The Research Foundation for Microbial Diseases of Osaka University). Both the test vaccine and placebo will be labeled, packed and transported to LMC as detailed in the SOP [*Shipping Procedures for Investigational Products from BIKEN to LMC*]. Formulation, preparation and manufacturing of BK-SE36 is in accordance with the 'Standards for Manufacturing Control and Quality Control of Investigational Products, and Standards for Buildings and Facilities of Manufacturing Plants for Investigational Products (GMP for Investigational Products)'(Pharmaceutical Affairs Bureau Notification No. 480 dated March 31, 1997). Investigational products conform to established requirements for sterility, safety and identity.

Test Vaccine: BK-SE36.

When reconstituted with 1.3 ml of the supplied diluent (1.6 ml of water is supplied, Japanese Pharmacopoeia), the solution contains 100 μg/ml of SE36 protein. The following volumes of the reconstituted vaccine will result in administration of the indicated doses:

Administration of 1.0 ml will deliver a 100 μg dose of SE36.
Administration of 0.50 ml will deliver a 50 μg dose of SE36.

Administration will be done immediately after reconstitution.

Placebo: Physiological saline.

The placebo is a prepared colorless, saline solution for direct use (Otsuka Pharmaceutical Co., Ltd., Japan). An appropriate volume will be withdrawn to correspond to the volume of the test vaccine used in the treatment group.

Details of investigational products storage, accountability, reconstitution and disposition of used/unused vials are detailed in the relevant SOPs [*Investigational Products Storage and Accountability*; *Shipping Procedures for Investigational Products from BIKEN to LMC*; *General Events from Informed Consent to Post Administration*]. All investigators will be trained in the SOPs, copies of which will be available for inspection and review by pertinent authorities. General notes/precautions are summarized as below:

- Product Storage. Investigational products will be maintained in locked refrigerators (with temperature recording capability) at 5 ± 3 °C under light protected conditions and should NOT be frozen at any time. The fridge will be connected to an outlet that can be serviced by a generator in case of power failure. Keys will only be routinely possessed by the principal investigator, co-principal investigator and designated trial site staff.

- Product reconstitution. Vials will only be reconstituted for single, immediate use. Partially used BK-SE36 vials should not be administered to other subjects. For placebo, one saline bottle can be used for a number of subjects during one administration day. The opened bottle should NOT be stored for another days'

use. Used vials should be kept in locked cabinets. The refrigerator and designated cabinet will be used solely for storage of investigational products.

- Product Accountability and Disposition: Records will be kept that document receipt, release for administration, disposal or return to the sponsor of all investigational products.
- Used vials will be stored at LMC, and accounted for until monitoring. All vials will be kept, until such time as the principal investigator and sponsor agree that there are no concerns about the investigational products accountability and that they can be discarded. Vials may then be disposed of according to SOP.
- A disposal certificate will be obtained for the regulatory authority requirement. The accountability and disposal report will be submitted to the NDA within three months from the Last Subject Out date. Copies of these records will be provided to the sponsor for archiving. The report should include:

  Date the trial started and ended; and the license/certificate number
  Clinical Trial License/Certificate for LMC
  Date(s) and quantity received for each investigational product (test vaccine and placebo)
  Balance of the investigational products (test vaccine and placebo)
  Drug destruction certificate, and/or written evidence of re-export of unused drug supplies to Japan (BIKEN)

6 CONDUCT OF THE TRIAL

6.1 Recruitment, Consent, Enrollment, Randomization and Administration

Recruitment and screening will be incremental until the desired numbers of subjects are enrolled. All screening tests, however, will have to be completed within 30 days prior to first administration. In the event, therefore, that at Stage 1, the desired number of subjects has not been reached, a minimum of 20 subjects per cohort (sero-negative or sero-positive), with subjects randomized to receive either BK-SE36 (n=14) or placebo (n=6), would signal the scheduling of trial day 1 (or Administration 1, see *Section 7*). An alternative for Stage 2 if the desired age cohorts cannot be obtained, is a cohort of 33 volunteers for each dose of BK-SE36 (half and full-dose) and 9 volunteers for each dose of placebo. If this situation becomes applicable at any proposed stage of trial (Stage 1 or 2), it will be reported to the sponsor, and the sponsor, on consultation with the principal investigator, will decide the allocation to different treatment regimen.

Volunteers (adults and in the case of children, children with parents and/or guardians) will first be invited through a general announcement in the village to come to the Centre for screening. During this initial screening visit, the volunteer will be formally briefed in English, Luo/Swahili on the nature and purpose of the trial to obtain voluntary consent.

The following items will be explained by the investigator:

1. This trial is a clinical investigation rather than a provision of clinical treatment
2. Trial objectives

3 Trial methods (investigational view points of the trial and subject screening criteria)
4 Trial period = 43 days (excluding Screening). During this period, clinic visits are highly encouraged. In addition, a 40-day post-trial follow-up is available, wherein, subjects are invited to go to LMC whenever subject is sick. The doctor assess the event as “not related, unlikely, possible, probably or definitely related” to administration.
5 Planned number of subjects participating in the trial
6 Expected clinical benefits and risks, discomforts or inconveniences; unforeseeable risk to the embryo or fetus if the subject may become pregnant
7 Compensation and treatment for health problems that may occur in association with the trial
8 Subject’s voluntary participation in the trial; in case of trial amendments/changes, subject can reject or withdraw from the trial at any time. Subjects will not be unfavorably handled due to rejection or withdrawal, or lose benefits that they should receive.
9 When information that may affect subject’s will to continue the trial is available, the information will be promptly provided to subject. When his will to continue or withdraw from the trial is confirmed, the provided information to/and from the subject will be recorded in writing.
When the explanatory documents are revised due to new important information (change of the trial protocol, etc.) that may be relevant to the subject’s consent during the trial, the subject’s will to continue the trial will be confirmed. The trial will be thoroughly explained using the revised explanatory documents, and voluntary written consent will be directly obtained from the subjects.
10 Conditions and reasons for discontinuation of the trial; circumstances under which the investigator may terminate participation, whether or not the subject consents to such termination.
11 The monitor, auditor, ethics committee, and regulatory authorities can look through the original documents, in which privacy of subjects will be ensured. Their access to the documents is admitted when the subject fills in his name with signature or imprint (fingerprint) on the consent forms.
12 The extent to which privacy and confidentiality will be maintained. With regards to serum collection, how specimens will be managed and stored; and what tests will be conducted (a separate consent form is to be accomplished).
13 Details of payment to subjects.
14 Names, occupations, and contact addresses of the investigators.
15 Consultation window of the medical institution to be contacted or referred to when a subject needs further information concerning the trial and subject’s right, or when health problems associated with the trial occurs.
16 When necessary the provision of an impartial witness during the informed consent process for volunteers unable to read or write.
17 The trial has been approved by a recognized Ugandan based IRC (MBL-IRC).

Volunteers and/or parents will be encouraged to ask questions, or investigators will ask questions (to volunteers), to identify those areas of the informed consent that might need further review. This will help ensure sufficient understanding before the consent form is signed. Volunteers and/or parents or guardians sign the consent form, or those unable to read or write, may place an imprint of their finger in place of a signature.

For fingerprint accomplished forms, an impartial witness of the community should sign the consent form to attest that the volunteer and/or parent fully comprehend the contents and has done so voluntarily. The volunteer will receive a copy of the completed consent forms, a medical history will be elicited and complete physical examination and laboratory tests will be conducted. No questions relating to health will be asked of volunteers or parents prior to consenting and all screening tests (below) will be performed only after informed consent has been obtained.

<1> Consultation
<2> Physical examination including vital signs:
Blood pressure (systolic/diastolic), pulse rate and body temperature
<3> Laboratory tests
<3.1> Hematology
Leukocyte count, erythrocyte count, hemoglobin, hematocrit, platelet count
<3.2> Blood chemistry
Total protein, albumin, total bilirubin, AST, ALT, AL-P, γ-GTP, serum amylase, total cholesterol, uric acid, urea nitrogen, creatinine, Na, K, glucose
<3.3> Malaria smear
<3.4> <u>for Stage 1 only.</u> Antibody levels to SE36 protein will be measured by ELISA to determine grouping to sero-negative or sero-positive against the SE36 protein
<3.5> Urinalysis and urine β-hCG
Qualitative glucose, bilirubin, ketone body, urinary occult blood reaction, pH, protein, urobilinogen, nitrite, leucocytes, specific gravity; β-hCG reaction for female subjects (adolescents/adults)
<4> Body weight and height

A subject screening log, subject enrollment log and medical records will be prepared. All volunteers who gave informed consent and the date of screening visit will be recorded. All information gathered during screening will be filed together as a Screening source document.

After screening, volunteers determined to be eligible, based on the inclusion and exclusion criteria, will be invited to participate in the trial. Volunteers will be excluded from participation if they meet any of the exclusion criteria. Volunteers, who were evaluated by investigators as otherwise healthy but falls out of range from urinalysis, hematology or biochemical parameters can be re-evaluated again. If a particular exclusion criteria is met during screening, the investigator evaluates if this is clinically non-significant and/or the condition can be resolved prior to administration. If considered as healthy by doctors medical assessment, but falls out of screening range, the volunteer can be included after seeking a subject waiver. Volunteers excluded, because of a clinically relevant finding, will be referred to regular physicians at LMC. Basic first aid treatment can be provided to such ineligible volunteers; however, long-term treatment and care will not be covered. Reason(s)/explanation(s) as to exclusion would be noted in the Screening source document.

Eligible volunteers will be assigned to a unique code, and an ID picture will be taken for reference. Volunteers will not be considered enrolled until they have been assigned to a subject code. If an eligible volunteer has previous medical record with LMC (herein referred to as 'Carte'), the documentation of the patient's medical history and Carte will be labeled with a subject code seal. If LMC has no previous records for the subject, a Carte for that subject will be kept on file as active records of the Centre. The name of the subject will only appear at the Carte in LMC. Particular care will also be taken to record means in order to contact the subject to ensure that follow-up is possible should the subject fail to return for scheduled visits.

The principal investigator and co-principal investigator will ensure randomization of subjects who fulfill all the inclusion criteria and none of the exclusion criteria. A CRF will also be filled in for all subjects that were administered at Visit 2. A week before trial day 1 (Administration 1), randomized subjects will be invited to visit the Centre again to detect the presence or early signs of febrile diseases. If malaria symptoms as defined in *Section* 6.2 are present, a blood smear will be done to confirm the diagnosis prior to malaria treatment as described. (Alternatively, additional visit 1 maybe conducted before randomization).

Every effort will be made to carry out administration at a designated day. Administration will be on the upper arm, 2 times at 21 ± 1 day interval. For stage 2, this window period may be extended for an additional day (thus ± 2 days) . In all subjects, the first and second administrations will preferably be injected on opposite arms. If more than one administration must be done in the same arm, the administration site should be separated by 1-2 inches so that any local reactions can be differentiated. The site of each administration will be documented in the subject record. The subject will receive the same investigational product at the same dose in both first and second administrations.

Test vaccine and the placebo will be administered under the supervision of investigator(s) skilled in the management of anaphylactic reactions. The subjects will be observed closely for at least 60 minutes (longer, if necessary), with appropriate medical treatment readily available in the unlikely event of anaphylactic reaction following the administration of investigational product. As with other aluminum hydroxide-adsorbed vaccines, hypersensitivity reactions would be expected to occur within the first 24 hours after administration; thus, subjects will return again 1 day post administration. Signs and symptoms (local and systemic events: pain, swelling, bruising at administration site, fever, nausea) will be solicited from the subject and recorded according to adverse events recording procedures.

When shock and anaphylaxis-like symptoms occur, airway should be secured, and treatment should be given corresponding to symptoms, such as epinephrine administration to recover blood pressure. Epinephrine will be injected parenterally in standard recommended doses. When acute thrombocytopenic purpura is suspected, the condition should be closely observed by hematology, and appropriate treatment should be given, such as adrenocortical steroid administration. A kit containing necessary supplies for the management of anaphylaxis will be on-site. An investigator will be trained with emergency resuscitation procedures (CPR).

Subjects will return again to the Centre: 1, 7, 14 and 21±1 days after each administration for scheduled consultations; physical examination and laboratory tests (trial days 2, 8, 15, 22, 23, 29, 36 and 43). Every effort will be made to ensure subject and parental compliance with scheduled visits. If a subject does not appear for a trial visit, an investigator will attempt to contact the subject or parent on the same day.

The proposed schedule of visits, physical examinations and laboratory tests allow for a reasonable time frame to be able to recognize promptly, document and manage adverse events. All adverse events occurring will be followed until resolution. If a serious adverse event occurs, appropriate measures will be taken to notify the principal investigator, sponsor and pertinent authorities as described in *Sections* 9.5 and 9.6.

On the day of the second administration (Day 22), criteria for continued eligibility will again be reviewed and verified. Test findings and adverse events must not conflict the safety assessment criteria described in *Section 4 (4.1-4.5)*.

Investigators will confirm the investigational product assignment and dose using the subject code and photograph in the source document file. All administrations will be done in a fashion that maintains single blinding (*Section* 3.2.3)

All data, except the investigators evaluation for AE severity grade and causality, will be recorded on source documents and transcribed into the CRF by trained investigators.

6.2 Malaria Episodes and Management of Symptomatic Malaria

A clinical episode of falciparum malaria is defined as the presence of *P. falciparum* asexual parasites on Giemsa-stained thick blood smear in the presence of the following: (1) fever, defined as axillary temperature ≥37.5°C; or history of fever within 48 hours in the absence of other evident clinical conditions that could explain the fever; and/or (2) one or more of the following symptoms consistent with malaria including but not limited to headache, vomiting, abdominal pain, or myalgia with or without fever. Investigators will use their best clinical judgment when to treat malaria and will not be precluded from treating malaria by this definition.

Because the present trial is on safety, weekly temperatures will be monitored for subjects throughout the trial. For any readings ≥37.5°C, or for any presumptive malaria diagnosis, a blood smear will be taken to confirm the diagnosis and all subjects with asexual parasitemia will be treated. Artemether/lumefantrine (AL, Coartem®) will be used for subjects who acquired uncomplicated malaria during the trial. In addition to artemether/lumefantrine, and in the unlikely event of treatment failure with this drug, dihydroartemisinin-piperaquine (DP, Duo-Cotecxin®) will also be available as an alternative treatment for uncomplicated malaria [refer to SOP: *Management for Symptomatic Malaria*].

Subjects found to have clinical malaria will be treated before any administration. Subjects evaluated in this manner will be given the appropriate dose of investigational product if their clinical symptoms resolve within 4 days. Administration will be done 1

week delayed from original administration schedule. However, if the clinical symptoms do not resolve within 4 days the subject will not be administered further. The subject will, however, be followed through for safety until resolution of symptoms.

In subjects with manifestations of severe malaria, definitive clinical management will be done according to the standard of care at the Centre.

6.3 Concomitant Medication/Treatment

At each trial visit, the investigator will ask the subject about any medication taken, including traditional medicines. Concomitant medication, including any vaccine other than the test vaccine, and any medication relevant to the protocol, including any specifically contraindicated or administered during the period starting from one week before first administration until the final visit, will be recorded in the CRF with trade name and/or generic name of the medication, medical indication, start and end dates of treatment.

6.4 Health Care Provision

Being the primary provider for medical care in Lira, LMC has, and continues to maintain, medicines and treatment regimens that meet the standards for health care. Basic emergency services are available and standard referrals can be arranged.

Free medical treatment will be provided to all enrolled subjects during the whole trial duration (43 + 40 days). Medical care that the subject receives which is unrelated to administration will remain the primary responsibility of the subject, parent or guardian, although routine medical problems (common illnesses, including malaria) will generally be managed by the trial at no cost to the subject within the trial period. The pharmacy at LMC will be able to provide subjects with common over-the-counter analgesics such as paracetamol, as well as antibiotics for the treatment of minor infections free of charge. Medical care for ailments related to administration will be covered at least until the condition has resolved.

For ailments related to administration, when the condition cannot be managed adequately within the expertise or licensure of the Centre, subjects will be referred to the highest attainable standard of care within the country. The referral process will be adequately documented and will adhere to UNCST guidelines. Subjects will always be informed of all options available for management of their conditions.

6.5 Trial Staff

Although some supporting staff, nurses and laboratory technicians will be from LMC, a number of personnel will also be hired specifically for the trial. All trial staff will undergo GCP training and protocol specific procedures.

7 TRIAL SCHEDULE/TIME SEQUENCE

7.1 Detailed Description of Trial Visits

Meetings will be held first within communities/village-level heads and authorities; neighboring towns to explain the purpose of the trial. The target population will be invited to come to the Centre for screening as described.

**Days -30 to 0**	**Screening & Enrollment of Subjects**	(4 to 5 hours)

Visit 1 ***(can be spread out to several days, as necessary)***

- Written Informed Subject/Parental Consent- verification and completion.
- Provision of medical history by subject/subject's parent(s)/guardian.
- Health assessment (consultation, physical examination including vital signs, body height and weight, laboratory tests including urinalysis, urine β-hCG, malaria smear).

** <u>For Stage 1, ONLY</u>: *blood sampling to assay SE36 protein reactivity by ELISA*

- Check for inclusion and exclusion criteria.
- Assignment of Subject code. Take photograph.
- Randomization to investigational products.

**Day 1 ± 1 day**	**Administration 1**	(≈5 hours)

Visit 2

Before administration:

- Check subject's code to confirm identity.
- Review inclusion/exclusion criteria and check for contraindications/precautions.
  *For females (adolescents/adults), obtain a urine sample for β-hCG testing. Ensure that the test is negative before proceeding; a positive test will exclude the volunteer from the trial.
- Record any baseline data for general symptoms; record any complaints and examine administration site for any abnormalities.
- Complete physical examination. Record vital signs (axillary temperature must be <37.5°C, blood pressure, pulse rate).
- Body weight.
- Blood sampling for laboratory tests, malaria smear and anti-SE36 protein antibody titer baseline data.
- Urinalysis

Check records; confirm subject code number, investigational product and dose.

- Administration of investigational product; record date and time.

After administration:

- Observe for 60 minutes. Examine administration site.
- Record blood pressure, pulse rate, temperature (at start and end of 60 minute observation period).
- Record AEs/SAEs.
- Instruct subject (or parent(s)/guardians) to return to LMC immediately should subject manifest any sign or symptom perceived as serious. Instruct subject (and/or parent(s)/guardians) to return the next day.

**Day 2 ± 1 day**	**24h Post-administration, first follow-up visit**	(30 minutes)

Visit 3

- Consultation and symptom-directed physical examination.
- Examine administration site.
- Check concomitant medication, if any. Review health status.
- Vital signs (temperature, blood pressure, pulse rate).
- Record AEs/SAEs.
- Urinalysis.
- Instruct subject (or parent(s)/guardians) to return to LMC for any AEs/SAEs. Instruct subject (and/or parent(s)/guardians) for next scheduled visit.

**Day 8 ± 1 day**	**Day 7 Post-administration, second follow-up visit**	(30 minutes)

Visit 4

- Consultation and symptom-directed physical examination.
- Examine administration site.
- Check concomitant medication, if any. Review health status.
- Vital signs (temperature, blood pressure, pulse rate).
- Record AEs/SAEs.
- Instruct subject (or parent(s)/guardians) to return to LMC for any AEs/SAEs. Instruct subject (and/or parent(s)/guardians) for next scheduled visit.

**Day 15 ± 1 day**	**Day 14 Post-administration, third follow-up visit**	(1 hour)

Visit 5

- Consultation and symptom-directed physical examination.
- Examine administration site.
- Check concomitant medication, if any. Review health status.
- Vital signs (temperature, blood pressure, pulse rate).
- Record AEs/SAEs.
- Urinalysis.
- Instruct subject (or parent(s)/guardians) to return to LMC for any AEs/SAEs. Instruct subject (and/or parent(s)/guardians) for next scheduled visit.

**Day 22 ± 1 day**	**Administration 2**	(4 to 5 hours)

Visit 6

Before administration:

- Consultation, review of health status during the past week; record any complaints and examination of administration site for any abnormalities.
- Check concomitant medications, if any.
- Complete physical examination. Record vital signs (temperature, blood pressure, pulse rate).
- Body weight.
- Blood sampling for laboratory tests, malaria smear and anti-SE36 protein antibody titer.
- Urinalysis.
- Review inclusion/exclusion criteria; Check for contradictions/precautions prior to administration
  *For females (adolescents/adults), obtain a urine sample for β-hCG testing. Ensure that the test is negative before proceeding; a positive test will make the subject ineligible for Administration 2

Check records; confirm subject code number, investigational product and dose.

- Administration of investigational product; record date and time.

After administration:

- Observe for 60 minutes. Examine administration site.
- Record blood pressure, pulse rate, temperature (at start and end of 60 minute observation period)
- Record AEs/SAEs.
- Instruct subject (or parent(s)/guardians) to return to LMC immediately should subject manifest any sign or symptom perceived as serious. Instruct subject (and/or parent(s)/guardians) to return the next day.

**Day 23 ± 1 day**	**24h Post-administration 2, fourth follow-up visit**	(30 minutes)

Visit 7

- Consultation and symptom-directed physical examination.
- Examine administration site.
- Check concomitant medication, if any. Review health status.
- Vital signs (temperature, blood pressure, pulse rate).
- Record AEs/SAEs.
- Urinalysis.
- Instruct subject (or parent(s)/guardians) to return to LMC for any AEs/SAEs. Instruct subject (and/or parent(s)/guardians) for next scheduled visit.

**Day 29 ± 1 day**	**Day 7 Post-administration 2, fifth follow-up visit**	(30 minutes)

Visit 8

- Consultation and symptom-directed physical examination.
- Examine administration site.
- Check concomitant medication, if any. Review health status.
- Vital signs (temperature, blood pressure, pulse rate).
- Record AEs/SAEs.
- Instruct subject (or parent(s)/guardians) to return to LMC for any AEs/SAEs. Instruct subject (and/or parent(s)/guardians) for next scheduled visit.

**Day 36 ± 1 day**	**Day 14 Post-administration 2, sixth follow-up visit**	(1 hour)

Visit 9

- Consultation and symptom-directed physical examination.
- Examine administration site.
- Check concomitant medication, if any. Review health status.
- Vital signs (temperature, blood pressure, pulse rate).
- Record AEs/SAEs.
- Urinalysis.
- Instruct subject (or parent(s)/guardians) to return to LMC for any AEs/SAEs. Instruct subject (and/or parent(s)/guardians) for next scheduled visit.

**Day 43 ± 1 day**	**Day 21 Post-administration 2, final visit**	(1 to 2 hours)

Visit 10

- Consultation, review health status: record any adverse events occurring after the last vaccine dose.
- Examine administration site.
- Check concomitant medications, if any.
- Complete physical examination. Record vital signs (temperature, blood pressure, pulse rate).
- Body weight.
- Blood sampling for laboratory tests, malaria smear and anti-SE36 protein antibody titer.
- Urinalysis and urine β-hCG
- Record AEs/SAEs.

**Additional follow-up will consist of visits to LMC whenever the subject is sick during the 43-day trial period. During each visit the subject will be evaluated and appropriately treated.*
***Concomitant medications include any vaccine or medicines administered during the trial. These should be recorded in the CRF with trade name or generic name, medical indication, start and end dates.*

**Day 44-84**	**40 day post-trial follow-up after visit 10**

After visit 10, subjects are invited to come to the Centre at any time, within 40 days, whenever they are sick. The investigators will assess the relationship of the adverse event to administration and all symptoms will be described in the CRF.

7.2 Outline of Trial Procedures

Stage 1. 21 to 40 y old

Visit[a]	1	2	3	4	5	6	7	8	9	10
**Trial Day (relative to administration day)**	0[b]	1	2	8	15	22	23	29	36	43
Village and family information; and discussion	x									
Written Informed Consent, Subject code, Photograph	x									
Verification of Eligibility Criteria: Inclusion/Exclusion	x	x				x				
Medical History	x									
Consultation	x	x	x	x	x	x	x	x	x	x
Physical examination	x	x	x	x	x	x	x	x	x	x
Vital signs	x	x	x	x	x	x	x	x	x	x
Laboratory tests (complete)[c]	x	x[d]				x[d]				x
Urinalysis only			x		x		x		x	
Body weight	x	x				x				x
Body height	x									
Serum for Antibody Response/Biological activity assay	x	x[d]				x[d]				x
Check contraindications/precautions		x				x				
Administration of investigational products		x				x				
Assess Adverse Events (AEs)		x[e]	x	x	x	x[e]	x	x	x	x
Assess Serious AEs (SAEs)		x[e]	x	x	x	x[e]	x	x	x	x
Concomitant Medications[f]			x	x	x	x	x	x	x	x
Review of Health Status[g]			x	x	x	x	x	x	x	x
Scheduled blood volumes	13	13	0	0	0	13	0	0	0	13
**Cumulative Blood** Vol. (ml)	13	26				39				52

[a]Trial days are relative to administration time point. Visit numbers and trial days are the same for all subjects.
[b]Performed within 30 days prior to administration; 0= recruitment, screening days. Visit 1 can be spread out to several days as necessary to complete recruitment and screening procedures.
[c]Laboratory tests (complete): hematology (blood counts); blood chemistry (total protein, albumin, total bilirubin, AST, ALT, AL-P, γ-GTP, serum amylase, total cholesterol, uric acid, urea nitrogen, creatinine, Na, K, glucose); malaria smear; urinalysis and urine β-hCG (qualitative glucose, bilirubin, ketone body, urinary occult blood reaction, pH, protein, urobilinogen, nitrite, leucocytes, specific gravity; pregnancy tests for female subjects (urine β-hCG)
[d]Blood sampling just before administration
[e]Immediately after administration and 60 min post administration
[f]Concomitant medication subject might take. As a rule, use of other drugs is prohibited from 1 week before administration until post administration.
[g]Record any new onset of chronic or acute diseases or medically significant conditions, unscheduled clinic visits and any new treatments since previous scheduled visit
----
** Within 40 days after visit 10, the subject will be invited to come to the Centre at any time they are sick. Investigators will assess the relationship of the adverse event to administration and all symptoms will be described in the CRF.

Stage 2. 6 to 20 y old

**Visit**[a]	**1**	**2**	**3**	**4**	**5**	**6**	**7**	**8**	**9**	**10**
**Trial Day (relative to administration day)**	**0**[b]	**1**	**2**	**8**	**15**	**22**	**23**	**29**	**36**	**43**
Village and family information; and discussion	x									
Written Informed Consent (Subject and/or Parent), Subject code, Photograph	x									
Verification of Eligibility Criteria: Inclusion/Exclusion	x	x				x				
Medical History	x									
Consultation	x	x	x	x	x	x	x	x	x	x
Physical examination	x	x	x	x	x	x	x	x	x	x
Vital signs	x	x	x	x	x	x	x	x	x	x
Laboratory tests (complete)[c]	x	x[d]				x[d]				x
Urinalysis only			x		x		x		x	
Body weight	x	x				x				x
Body height	x									
Serum for Antibody Response/Biological activity assay		x[d]				x[d]				x
Check contraindications/precautions		x				x				
Administration of investigational products		x				x				
Assess Adverse Events (AEs)		x[e]	x	x	x	x[e]	x	x	x	x
Assess Serious AEs		x[e]	x	x	x	x[e]	x	x	x	x
Concomitant Medications[f]			x	x	x	x	x	x	x	x
Review of Health Status[g]			x	x	x	x	x	x	x	x
Scheduled blood volumes	8	13	0	0	0	13	0	0	0	13
**Cumulative Blood** Vol. (ml)	8	21				34				47

[a]Trial days are relative to administration time point. Visit numbers and trial days are the same for all subjects.
[b]Performed within 30 days prior to administration; 0= recruitment, screening days. Visit 1 can be spread out to several days as necessary to complete recruitment and screening procedures.
[c]Laboratory tests (complete): hematology (blood counts); blood chemistry (total protein, albumin, total bilirubin, AST, ALT, AL-P, γ-GTP, serum amylase, total cholesterol, uric acid, urea nitrogen, creatinine, Na, K, glucose); malaria smear; urinalysis and urine β-hCG (qualitative glucose, bilirubin, ketone body, urinary occult blood reaction, pH, protein, urobilinogen, nitrite, leucocytes, specific gravity; pregnancy tests for female (adolescents) subjects (urine β-hCG).
[d]Blood sampling just before administration
[e]Immediately after administration and 60 min post administration
[f]Concomitant medication subject might take. As a rule, use of other drugs is prohibited from 1 week before administration until post administration.
[g]Record any new onset of chronic or acute diseases or medically significant conditions, unscheduled clinic visits and any new treatments since previous scheduled visit

----

** Within 40 days after visit 10, the subject will be invited to come to the Centre at any time they are sick. Investigators will assess the relationship of the adverse event to administration and all symptoms will be described in the CRF.

8 TRIAL PARAMETERS AND LABORATORY TESTS

8.1 Safety

Consultation and physical examination will assess the safety of the test vaccine by recording vital signs (blood pressure, pulse rate, body temperature), systemic (loss of appetite, drowsiness, irritability/fussiness), and local events (administration site pain or necrosis, administration site swelling). Aside from the baseline data gathered during screening and before administration, subjects will be observed 1 hour; and 1, 7, 14 and 21 days after each administration. Weekly monitoring of malaria infection will be done either by axillary temperature readings or when clinical investigators deemed fit. If axillary temperature is ≥37.5°C, or presumptive malaria diagnosis is made, thick and thin blood smears will be made.

Thick blood smears will be evaluated for the presence of parasitemia (asexual forms only) and gametocytes. Parasite and gametocyte densities will be calculated from thick blood smears by counting the number of asexual parasites and gametocytes, respectively, per 200 leukocytes (or per 500, if the count is <10 parasites or gametocytes/200 leukocytes), assuming a leukocyte count of 8,000/μl. A thick blood smear will be considered negative when the examination of 500 WBCs does not reveal asexual parasites or gametocytes. Thin blood smears will be evaluated to determine parasite species.

Safety will also be evaluated using hematology, blood chemistry and urinalysis results. Tests are for hematologic disease (blood counts), liver function (total protein, albumin, bilirubin, AST, ALT, AL-P, γ-GTP), lipid profiles (cholesterol), pancreatic function (serum amylase, glucose), kidney/renal function (uric acid, urea nitrogen, creatinine, urinalysis tests) and serum electrolyte changes (Na and K). Laboratory tests will be done during screening, before the first and second administration; 1 and 14 days (for urinalysis only), and 21 days after each administration. These tests will be performed on-site at LMC clinical laboratory.

Blood volumes. The total volume of blood to be drawn over the 43-day trial duration is detailed below. However, additional blood may be obtained as deemed necessary by one of the investigators or clinicians to evaluate any illness or condition.

Stage 1, 21 to 40 y old
Total blood sample volume: 52 ml
Total number of blood sampling times: 4 times
[Details]
1. Screening: 13 ml (laboratory tests + antibody measurement)
2-4. Laboratory tests: 24 (8 ml x 3 times*)
Antibody measurement: 15 ml (5 ml x 3 times*)

Stage 2, 6 to 20 y old
Total blood sample volume: 47 ml
Total number of blood sampling times: 4

[Details]
1. Screening: 8 ml (laboratory tests)
2-4. Laboratory tests: 24 (8 ml x 3 times*)
Antibody measurement: 15 ml (5 ml x 3 times*)
**[3 times = before first and second administration; and 21 days post administration]*

8.2 Immunogenicity

Aside from safety, the presence of antibodies against *P. falciparum* SE36 protein will be determined during screening, the time points before administration and 21± 1 day post administration. These sampling times are set to confirm the antibody titer elevation due to administration. Separation of serum/plasma from the venous blood will be performed at LMC and serum samples will be aliquoted, transported and stored at MBL.

Anti-SE36 protein ELISAs will be performed both at MBL, Kampala and at BIKEN, Japan. SOP for antibody titers and prevalence determinations [*Measuring Antibody Titers*], as established in BIKEN, was already provided to MBL. Only the ELISA result at MBL will be used for grouping subjects into sero-negative or sero-positive to the SE36 protein. Only SE36 protein ELISA results obtained in BIKEN will be used for the analysis of vaccine-induced immunity. Assay results obtained by MBL at screening will be compared to ELISA values obtained in BIKEN as part of an on-going transfer of technologies.

<u>Optional Immunological Readouts.</u> The biological activity of induced antibodies (GIA, ADCI, epitope mapping) will also be evaluated. Results from these tests will be treated as research output of this trial. If other assays, which might be useful in evaluating the immune response to the test vaccine, are to be done in the future, approval will be requested from MBL-IRC and UNCST.

9 ADVERSE EVENTS

It is the responsibility of the investigators to document all adverse events according to the detailed guidelines set out below. The subjects will be instructed to contact the investigator immediately should they manifest any signs or symptoms that they perceive as serious during the trial.

Non-serious adverse events (AEs) and serious AEs (SAEs) will be evaluated as distinct events given their different medical nature. If an event meets the criteria to be determined “serious”, it will be investigated by the principal investigator to the extent that ALL relevant contributing factors can be made.

9.1 Definition of Adverse Events

Adverse events (AEs) include all unfavorable and unintended signs (including laboratory test abnormalities), symptoms, and diseases regardless of the presence or absence of a causal relationship with the test vaccine. All AEs must be graded according

to intensity (Table 5, 6) and relationship to the test vaccine (Table 8) as described in *Section 9.3*.
An adverse event shall be deemed unexpected if:

a. It is previously unobserved or undocumented in Phase 1a or in other vaccines using alum-based adjuvant
b. The nature of severity is not consistent with information in the investigators brochure or other safety information known at the time;
c. The event is observed with higher frequency or severity than previously documented.

Unexpectedness shall not include events that may reasonably be extrapolated from *in vitro* and animal studies.

9.2 Definition of Serious Adverse Events

A serious adverse event (SAE) is any untoward medical phenomenon that results in death, threat to life, persistent or significant disability or incapacity, hospitalization or its prolongation, or congenital anomaly or birth defect in the offspring of a subject. In addition, an important medical event that may jeopardize the subject or may require intervention to prevent one of the other outcomes listed above is considered as a serious adverse event.

Additional definitions:

Life threatening: A SAE is life threatening if the subject was at risk of death at the time of the development of event; it does not refer to an event that hypothetically might have caused death if it were severer.

Disabling or incapacitating: A SAE is incapacitating or disabling if the event results in a substantial disruption of the subject's ability to carry out normal physical and mental functions. This definition is not intended to include experiences of relatively minor medical significance such as headache, nausea, vomiting, diarrhea, influenza, and accidental trauma (e.g. sprained ankle).

Hospitalization: In general, hospitalization signifies that the subject has been kept (usually involving at least an overnight stay) at the hospital or emergency ward for treatment that would not be appropriate in the physician’s office or other out-patient clinic.

9.3 Identification of Adverse Events

At each follow-up visit (Days 1, 2, 8, 15, 22, 23, 29, 36, 43, and any unscheduled day), investigators will assess subjects for any physical, or laboratory change/abnormality. Subjects will be closely monitored for 60 minutes following each administration. Local signs and symptoms that occur after administration of the investigational products will be handled as adverse event. In addition, any new event, or an event present at baseline that is increasing in severity, will be considered an adverse event. The nature of the event, date and time (where appropriate) of onset, outcome, intensity, and relationship to

administration will be recorded. Any corrective treatment will be noted in the CRF. Adverse events already documented in the CRF, i.e., at previous assessment and designated as ongoing will be reviewed at subsequent visits, as necessary. If these events have resolved, the documentation in the CRF will be completed, including the date when the adverse event resolved. If an adverse event changes in frequency or intensity during the trial, a new record of the event will be started.

A severity grading scale, based on toxicity grading scales adopted and modified from WHO (2003), will be used to grade severity of symptoms, physical examination findings, and laboratory tests (Table 6). The causal relationship table (5 categories shown in Table 8) will also be used.

For items without established standard values, a guide to grading severity grade is provided in Table 6. Investigators will judge the event with discretion. For blood pressure, pulse rate, and body temperature, values that deviate from the standard values are regarded as abnormal variations. When an abnormal value noted after administration is not regarded as an abnormal variation, the reason for the judgment will be described in the CRF. When a change in value, is judged clinically problematic by the investigators, although the value is within the standard range before and after administration, it will be regarded as an abnormal variation and handled as an adverse event.

9.4 Following-up of Adverse Events and Assessment of Outcome

Investigators will follow up subjects with non-serious adverse events until the subject completes the trial. For each possible adverse event identified and graded as moderate, severe or life threatening, however, an adverse event report form will be completed (as detailed below). Clinically significant laboratory abnormalities will be followed up until they have returned to normal or the condition has stabilized. The following information will be recorded for all adverse events that are reported:

- Description of event
- Date of event onset
- Date event reported
- Maximum severity of the event
- Maximum suspected relationship of the event to test vaccine
- Is the event serious?
- Initials of the person reporting the event
- Was the event temporary or recurring in nature?
- Outcome
- Date the event resolved

9.5 Reporting of Serious Adverse Events

In the event that one or more serious adverse reactions probably or suspected of being related to vaccination are detected following any administration in any of the cohorts, the principal investigator informs the sponsor within 24 hours by email, fax or telephone followed with a written report within 3 working days [refer to SOP for *SAE Report*]. The IRC and regulatory committees will also be notified no later than 7 days

upon such event(s). Every serious adverse event that is not resolved at the time the initial written report is filed will have a follow-up information submitted not be later than 8 days after the first report.

In addition, according to the UNCST guidelines for adverse events, the following shall be reported promptly to regulatory bodies:

a. All serious adverse events irrespective of relationship to the test vaccine
b. All unexpected events of greater than moderate severity irrespective of relationship to test vaccine
c. All events associated with protocol violations irrespective of severity and relationship to test vaccine
d. When criteria for stopping or pausing of a trial as stipulated in the protocol are met
e. Any event mandated by regulatory authorities.

9.6 Treatment and Actions Taken Upon Occurrence of AEs and SAEs

When an adverse event occurs after administration of the test vaccine, the investigators will immediately perform appropriate treatment as needed, conduct follow-ups until the condition returns to the normal (within the standard range) or pretreatment state (pretreatment value). Details of any treatment will be recorded. All adverse events, including serious adverse events after administration, will be documented on the appropriate case report form. Investigators will be available to the subjects 24 hours a day for the duration of the trial.

9.7 Discontinuation Criteria Due to Adverse Events

The trial will be immediately discontinued when the investigators consider that continuation of the trial is not appropriate due to an adverse event meeting the criteria described below. The investigators will immediately inform it to the subject, and guarantee appropriate treatment and processing after the discontinuation.

<u>[Discontinuation criteria for individual subjects]</u>
(1) Occurrence of a serious adverse event
(2) Severe local reactions, such as severe flare/redness centering the administration site and swelling extending to the forearm
(3) Grade 3 or 4 laboratory abnormality test values based on Table 6
(4) When the investigators consider that continuation of the trial is problematic

9.8 Stopping Criteria

Discontinuation/temporary suspension of the trial is appropriate based on the symptoms, frequency and severity of an adverse event for which a causal relationship to the investigational product cannot be ruled out. Trial interventions would be temporarily suspended until a safety review is convened. The reason (*e.g.* occurrence of a serious adverse event for which a causal relationship cannot be ruled out) and all the events

leading to discontinuation will be recorded.

When the trial has to be discontinued, the principal investigator will immediately report it to the sponsor. The principal investigator will also inform in writing the Ethics committee (MBL-IRC) and regulatory bodies (UNCST and NDA). The principal investigator will promptly inform the subjects, and perform appropriate treatment.

9.9 Assessment of AEs and SAEs

Table 5. Severity of adverse events

0: Absent	Normal; No adverse event
1: Mild	Awareness of sign or symptom, but transient and easily tolerable; mild discomfort; no limitation in activity; no treatment or medical intervention is necessary
2: Moderate	Sufficiently discomforting to interfere with normal everyday activities; some assistance maybe needed; no or minimal medical intervention is required (outpatient treatment)
3: Severe	Prevents normal, everyday activities; some assistance usually required; treatment/hospitalization is necessary, may or may not necessitate administration of corrective therapy
4: Very severe	Extreme limitation in activity, significant assistance is required; significant medical intervention/therapy required; requires in-patient hospitalization or prolongation of existing hospitalization<br>All events that are life threatening and usually require emergency procedures

Table 6. Table for grading the severity of AEs and SAEs in adults and children.

(Adopted and modified from the Division of AIDS table for grading the severity of adult and pediatric adverse events; publish date: December. 2004; http://www3.niaid.nih.gov/research/resources/DAIDSClinRsrch/PDF/Safety/DAIDSAEGradingTable.pdf)

<u>Grading Adult and Pediatric AEs</u>

The DAIDS AE grading table includes parameters for grading both Adult and Pediatric AEs. When a single set of parameters is not appropriate for grading specific types of AEs for both Adult and Pediatric populations, separate sets of parameters for Adult and/or Pediatric populations (with specified respective age ranges) are given in the table. If there is no distinction in the table between Adult and Pediatric values for a type of AE, then the single set of parameters listed is to be used for grading the severity of both Adult and Pediatric events of that type.

<u>Determining Severity Grade</u>

If the severity of an AE could fall under either one of two grades (e.g., the severity of an AE could be either Grade 2 or Grade 3), select the higher of the two grades for the AE.

Definitions

Basic Self-care Functions	*Adult*<br>Activities such as bathing, dressing, toileting, transfer/movement, continence and feeding.<br>*Young Children*<br>Activities that are age and culturally appropriate (e.g., feeding self with culturally appropriate eating implement).
LLN	Lower limit of normal
Medical Intervention	Use of pharmacologic or biologic agent(s) for treatment of an AE.
NA	Not Applicable
Operative Intervention	Surgical OR other invasive mechanical procedures.
ULN	Upper limit of normal
Usual Social & Functional Activities	*Adult*<br>Adaptive tasks and desirable activities, such as going to work, shopping, cooking, use of transportation, pursuing a hobby, etc.<br>*Young Children*<br>Activities that are age and culturally appropriate (e.g., social interactions, play activities, learning tasks, etc.).

**Additional input was also obtained from: IAVI's - A002 (TGC - 14F01) severity table: *A phase 2, randomized, placebo-controlled, double-blind trial to evaluate the safety and immunogenicity of* tgAAC09, *an HIV vaccine containing Clade* C gag-PR-ΔRT DNA *in an Adeno-Associated Virus (AAV) Capsid, administered twice, at three dosage levels and two dosing intervals.*

CLINICAL				
**PARAMETER**	**GRADE 1 MILD**	**GRADE 2 MODERATE**	**GRADE 3 SEVERE**	**GRADE 4 POTENTIALLY LIFE-THREATENING**
**ESTIMATING SEVERITY GRADE**				
Clinical adverse event NOT identified elsewhere in this AE grading table	Symptoms causing no or minimal interference with usual social & functional activities.<br><br>Mild discomfort.<br>No need for medical intervention.	Symptoms causing greater than minimal interference with usual social & functional activities.<br><br>Moderate discomfort.<br>May require no or minimal medical intervention.	Symptoms causing inability to perform usual social & functional activities.<br><br>Severe discomfort.<br>Requires medical intervention.	Symptoms causing inability to perform basic self-care functions OR Medical or operative intervention indicated to prevent permanent impairment, persistent disability, or death
**ADMINISTRATION SITE REACTIONS**				
Administration site **pain** (pain without touching) Or **Tenderness** (pain when area is touched)	Pain/tenderness causing no or minimal limitation of use of arm	Pain/tenderness limiting use of arm OR Pain/tenderness causing greater than minimal interference with usual social & functional activities<br>(May require single dose of analgesic)	Pain/tenderness causing inability to perform usual social & functional activities. Requires medical intervention (e.g. repeated doses of analgesic)	Pain/tenderness causing inability to perform basic self-care function OR Hospitalization (other than emergency room visit) indicated for management of pain/tenderness

PARAMETER	GRADE 1 MILD	GRADE 2 MODERATE	GRADE 3 SEVERE	GRADE 4 POTENTIALLY LIFE-THREATENING
**Erythema (redness) or skin discoloration**	Light red blush up to 25% of the circumference of the upper arm	Marked redness involving up to 50% of the circumference of the arm	Brick red involving >50% of the circumference of the upper arm	NA
**Edema (swelling)**	Light edema involving up to 25% of the circumference of the upper arm	Marked edema involving up to 50% of the circumference of the arm	Significant edema >50% of the circumference of the upper arm	NA
**Induration/nodule formation**	Hardening under the skin <1.5 cm in diameter	Hardening under the skin 1.5-3.0 cm in diameter	Hardening under the skin >3.0 cm in diameter	NA
**Skin damage (vesicle, ulcer)**	Vesicles or superficial disruption of epithelium < 1 cm	Vesicles or superficial disruption of epithelium 1-2 cm	Full thickness disruption of the epithelium (ulceration) > 2 cm	Necrosis (involving dermis and deeper tissue)
**Formation of crust or scab**	Crust, scab or scar ≤ 2cm	Crust, scab or scar 2-4cm	Crust, scab or scar > 4cm	NA
**Lympadenopathy**	Local, > 1 node/ >2cm	Regional with associated graded pain	Generalized and/or with associated graded pain	Generalized with constitutional symptoms (i.e. fever, headache, etc) requiring hospitalization
**SYSTEMIC REACTOGENICITY** SIGNS/SYMPTOMS				
**Headache**	Minimal headache causing no interference with usual social & functional activities	Moderate headache causing some interference with daily activities. May require single dose of analgesic.	Significant headache causing marked limitation of daily activities (e.g. unable to work). Requires medical intervention (e.g. repeated doses of analgesic)	Symptoms causing inability to perform basic self-care functions OR Hospitalization indicated (other than emergency room visit) OR Headache with significant impairment of alertness or other neurologic function
**Chills**	Symptoms causing no or minimal interference with usual social & functional activities	Symptoms causing greater than minimal interference with usual social & functional activities	Symptoms causing inability to perform usual social & functional activities	NA

PARAMETER	GRADE 1 MILD	GRADE 2 MODERATE	GRADE 3 SEVERE	GRADE 4 POTENTIALLY LIFE-THREATENING
**Fatigue Malaise**	Symptoms causing no or minimal interference with usual social & functional activities	Symptoms causing greater than minimal interference with usual social & functional activities	Symptoms causing inability to perform usual social & functional activities	Incapacitating fatigue/ malaise symptoms causing inability to perform basic self-care functions
**Myalgia (non-administration site)**	Muscle pain causing no or minimal interference with usual social & functional activities	Muscle pain causing greater than minimal interference with usual social & functional activities	Muscle pain causing inability to perform usual social & functional activities	Disabling muscle pain causing inability to perform basic self-care functions
**Arthralgia**	Joint pain causing no or minimal interference with usual social & functional activities	Joint pain causing greater than minimal interference with usual social & functional activities	Joint pain causing inability to perform usual social & functional activities	Disabling joint pain causing inability to perform basic self-care functions
**Fever (nonaxillary)**	37.7 – 38.6°C	38.7 – 39.3°C	39.4 – 40.5°C	> 40.5°C
**Nausea**	Transient (< 24 hours) or intermittent nausea with no or minimal interference with oral intake	Persistent nausea resulting in decreased oral intake for 24 – 48 hours	Persistent nausea resulting in minimal oral intake for > 48 hours OR Aggressive rehydration indicated (e.g., IV fluids)	Life-threatening consequences (e.g., hypotensive shock)
**Vomiting**	Transient or intermittent vomiting with no or minimal interference with oral intake	Frequent episodes of vomiting with no or mild dehydration	Persistent vomiting resulting in orthostatic hypotension OR Aggressive rehydration indicated (e.g., IV fluids)	Life-threatening consequences (e.g., hypotensive shock)
**Pruritis associated with administration** See also:Pruritis (itching - no skin lesions)	Itching localized to administration site AND Relieved spontaneously or with < 48 hours treatment	Itching beyond the administration site but not generalized OR Itching localized to administration site requiring ≥ 48 hours treatment	Generalized itching causing inability to perform usual social & functional activities	NA
**Pruritis** (itching – no skin lesions)	Itching causing no or minimal interference with usual social & functional activities	Itching causing greater than minimal interference with usual social & functional activities	Itching causing inability to perform usual social & functional activities	NA

PARAMETER	GRADE 1 MILD	GRADE 2 MODERATE	GRADE 3 SEVERE	GRADE 4 POTENTIALLY LIFE-THREATENING
**Rash**	Localized maculopapular (non-urticarial) rash at the injection site area causing no interference with daily activities	Diffuse maculopapular (non-urticarial) rash at the injected arm (beyond injection site) causing some limitation of daily activities	Diffuse macular, maculopapular, or morbilliform rash with vesicles or limited number of bullae OR Superficial ulcerations of mucous membrane limited to one site	Extensive or generalized bullous lesions OR Stevens-Johnson syndrome OR Ulceration of mucous membrane involving 2 or more distinct mucosal sites OR Toxic epidermal necrolysis (TEN)
**Allergic reaction**	Localized urticaria at injection site causing no interference with daily activities	Diffuse urticaria at the injected arm (beyond injection site) and causing some limitation of daily activities	Systemic urticaria or angioedema causing marked limitation of daily activities. Requires medical intervention	Acute anaphylaxis OR Life-threatening bronchospasm OR laryngeal edema
**OTHER CLINICAL OBSERVATIONS**				
**Hypertension**				
**Adult > 17 years** (with repeat testing at same visit)	> 140 – 159 mmHg systolic<br>OR<br>> 90 – 99 mmHg diastolic	> 160 – 179 mmHg systolic<br>OR<br>> 100 – 109 mmHg diastolic	> 180 mmHg systolic<br>OR<br>> 110 mmHg diastolic	Life-threatening consequences (e.g., malignant hypertension) OR Hospitalization indicated (other than emergency room visit)
**Pediatric ≤ 17 years** (with repeat testing at same visit)	NA	91st – 94th percentile adjusted for age, height, and gender (systolic and/or diastolic)	≥ 95th percentile adjusted for age, height, and gender (systolic and/or diastolic)	Life-threatening consequences (e.g., malignant hypertension) OR Hospitalization indicated (other than emergency room visit)
**Hypotension**	NA	Symptomatic, corrected with oral fluid replacement	Symptomatic, IV fluids indicated	Shock requiring use of vasopressors or mechanical assistance to maintain blood pressure
**Cardiac Arrhythmia**	Asymptomatic with transient dysrhythmia causing no interference with daily activities.<br>No treatment required.	Notable symptoms causing some interference with daily activities.<br>Non-urgent treatment required	Symptomatic and incompletely controlled by medical or invasive treatment.	NA
**Hemorrhage, blood loss**	Asymptomatic and requiring no therapy	Mildly symptomatic	Gross blood loss **AND/OR** 1–2 units transfused	NA

PARAMETER	GRADE 1<br>MILD	GRADE 2<br>MODERATE	GRADE 3<br>SEVERE	GRADE 4<br>POTENTIALLY LIFE-THREATENING
**Constipation**	Minimally symptomatic. No medical intervention required.	Significant abdominal pain with impaction requiring prescription	Requiring disimpaction<br>**AND/OR**<br>Hospital treatment	NA
**Diarrhea**	Transient or intermittent episodes of unformed stools OR Increase of ≤ 3 stools over baseline per 24-hour period	Persistent episodes of unformed to watery stools OR Increase of 4 – 6 stools over baseline per 24-hour period	Bloody diarrhea OR Increase of ≥ 7 stools per 24-hour period OR IV fluid replacement indicated	Life-threatening consequences (e.g., hypotensive shock)
**Oral Discomfort/Dysphagia**	Mild discomfort, no difficulty swallowing	Difficulty swallowing but able to eat and drink	Unable to swallow solids;<br>Loss of appetite associated with significant weight loss	Life-threatening consequences OR Aggressive intervention indicated [e.g., tube feeding or total parenteral nutrition (TPN)
**Alteration in personality-behavior or in mood** (e.g., agitation, anxiety, depression, mania, psychosis)	Alteration causing no or minimal interference with usual social & functional activities	Alteration causing greater than minimal interference with usual social & functional activities	Alteration causing inability to perform usual social & functional activities	Behavior potentially harmful to self or others (e.g., suicidal and homicidal ideation or attempt, acute psychosis) OR Causing inability to perform basic self-care functions
**Paresthesia (burning, tingling, etc.)**	Minimal discomfort resulting in minimal or no interference with daily activities	Notable symptoms resulting in greater than minimal changes in daily activities	Marked and persistent discomfort resulting in significant incapacity<br>**AND/OR**<br>Narcotic analgesia required for symptomatic improvement	NA
**Infection** (any other than HIV infection)	Localized, no systemic antimicrobial treatment indicated AND Symptoms causing no or minimal interference with usual social & functional activities	Systemic antimicrobial treatment indicated OR Symptoms causing greater than minimal interference with usual social & functional activities	Systemic antimicrobial treatment indicated AND Symptoms causing inability to perform usual social & functional activities OR Operative intervention (other than simple incision and drainage) indicated	Life-threatening consequences (e.g., septic shock)

PARAMETER	GRADE 1<br>MILD	GRADE 2<br>MODERATE	GRADE 3<br>SEVERE	GRADE 4<br>POTENTIALLY LIFE-THREATENING
**Neuromuscular weakness** (including myopathy & neuropathy)	Asymptomatic with decreased strength on exam OR Minimal muscle weakness causing no or minimal interference with usual social & functional activities	Muscle weakness causing greater than minimal interference with usual social & functional activities	Muscle weakness causing inability to perform usual social & functional activities	Disabling muscle weakness causing inability to perform basic self-care functions OR Respiratory muscle weakness impairing ventilation
**Dyspnea** or respiratory distress				
**Adult ≥ 14 years**	Dyspnea on exertion with no or minimal interference with usual social & functional activities	Dyspnea on exertion causing greater than minimal interference with usual social & functional activities	Dyspnea at rest causing inability to perform usual social & functional activities	Respiratory failure with ventilatory support indicated
**Pediatric < 14 years**	Wheezing OR minimal increase in respiratory rate for age	Nasal flaring OR Intercostal retractions OR Pulse oximetry 90 – 95%	Dyspnea at rest causing inability to perform usual social & functional activities OR Pulse oximetry < 90%	Respiratory failure with ventilatory support indicated
**Cough**	Transient cough resulting in minimal or no interference with daily activities	Recurrent or persistent cough resulting in greater than minimal interference with daily activities	Uncontrolled cough causing significant interference with daily activities	NA
**Arthritis**	Mild pain with no joint swelling.<br>No interference with daily activities.	Moderate pain with inflammation, erythema, or joint swelling.<br>Some interference with daily activities.	Severe pain with inflammation, erythema, or joint swelling causing significant incapacity.	NA
**Skin** (general)	Localized, asymptomatic.	Diffuse with notable symptoms and/or some interference with daily activities.	Generalized, marked symptoms and/or significantly interference with daily activities.	NA

PARAMETER		GRADE 1 MILD	GRADE 2 MODERATE	GRADE 3 SEVERE	GRADE 4 POTENTIALLY LIFE-THREATENING
**LABORATORY**<br>Values indicated in the first column [parameter] are reference ranges of these tests as applicable for Stage 1. Reference ranges applicable for Stage 2 are appended in *Supplemental Reference 1*.					
**HEMATOLOGY**					
Hemoglobin (Hgb)					
	Male (14 - 17.4 g/dl)<br>*Female (12 - 16 g/dl)*	10.0 – 10.9 g/dl OR Any decrease 2.5 – 3.4 g/dl	9.0 – 9.9 g/dl OR Any decrease 3.5 – 4.4 g/dl	7.0 – 8.9 g/dl OR Any decrease ≥ 4.5 g/dl	< 7.0 g/dL
Platelets, decreased (150 - 400 x 10^9/l)		100.000 x 10^9 – 124.999 x 10^9/l	50.000 x 10^9 – 99.000 x 10^9/l	25.000 x 10^9 – 49.999 x 10^9/l	< 25.000 x 10^9/l
Platelets, elevated		NA	550-600 x 10^9/l	> 600 x 10^9/l	NA
WBC, decreased Adult (5 - 10 x 10^9/l)		2.0 x 10^9 – 2.5 x 10^9/l	1.5 x 10^9 – 1.9 x 10^9/l	1.0 x 10^9 – 1.4 x 10^9/l	< 1.0 x 10^9/l
**CHEMISTRIES**					
Albumin, serum, low (3.8 - 5.1 g/dl)		3.0 g/dl – < LLN	2.0 – 2.9 g/dl	< 2.0 g/dl	NA
Alkaline Phosphatase<br>Male (80 - 306 U/l)<br>Female (64 - 306 U/l)		1.25 – 2.5 x ULN	2.6 – 5.0 x ULN	5.1 – 10.0 x ULN	> 10.0 x ULN
ALT (SGPT)<br>Male (up to 42 U/l)<br>Female (up to 32 U/l)		1.25 – 2.5 x ULN	2.6 – 5.0 x ULN	5.1 – 10.0 x ULN	> 10.0 x ULN
AST (SGOT)<br>Male (up to 37 U/l)<br>Female (up to 31 U/l)		1.25 – 2.5 x ULN	2.6 – 5.0 x ULN	5.1 – 10.0 x ULN	> 10.0 x ULN
Bilirubin (Total) (up to 1.1 mg/dl)		1.1 – 1.5 x ULN	1.6 – 2.5 x ULN	2.6 – 5.0 x ULN	> 5.0 x ULN
Cholesterol (fasting)		*LMC values: up to 30 y: (up to 180mg/dl); above 30 y: (up to 200 mg/dl)*			
	Adult ≥ 18 years	>180 – 239 mg/dl	240 – 300 mg/dl	> 300 mg/dl	NA
	Pediatric < 18 years	170 – 199 mg/dl	200 – 300 mg/dl	> 300 mg/dl	NA
Creatinine<br>Male (0.6 - 1.1 mg/dl)<br>Female (0.5 - 0.9 mg/dl)		1.1 – 1.3 x ULN	1.4 – 1.8 x ULN	1.9 – 3.4 x ULN	≥ 3.5 x ULN

PARAMETER		GRADE 1 MILD	GRADE 2 MODERATE	GRADE 3 SEVERE	GRADE 4 POTENTIALLY LIFE-THREATENING
Glucose, serum, high		LMC values: random blood sugar (70-100 mg/dl); fasting blood sugar (75-115 mg/dl)			
	Nonfasting	116 – 160 mg/dl	161 – 250 mg/dl	251 – 500 mg/dl	> 500 mg/dl
	Fasting	110 – 125 mg/dl	126 – 250 mg/dl	251 – 500 mg/dl	> 500 mg/dl
Glucose, serum, low					
	Adult and Pediatric ≥ 1 month	55 – 64 mg/dl	40 – 54 mg/dl	30 – 39 mg/dl	< 30 mg/dl
Pancreatic amylase (up to 90 U/l)		1.1 – 1.5 x ULN	1.6 – 2.0 x ULN	2.1 – 5.0 x ULN	> 5.0 x ULN
Potassium, serum, high (3.6 - 5.5 mmol/l)		5.6 – 6.0 mmol/l	6.1 – 6.5 mmol/l	6.6 – 7.0 mmol/l	> 7.0 mmol/l
Potassium, serum, low (3.6 - 5.5 mmol/l)		3.2 – 3.4 mmol/l	3.0 – 3.1 mmol/l	≤ 2.9 mmol/l	NA
Sodium, serum, high (135 - 155 mmol/l)		146 – 150 mmol/l	151 – 154 mmol/l	155 – 159 mmol/l	≥ 160 mmol/l
Sodium, serum, low (135 - 155 mmol/l)		130 – 135 mmol/l	125 – 129 mmol/l	121 – 124 mmol/l	≤ 120 mmol/l
Uric acid<br>*Male* (3.4 - 7.0 mg/dl)<br>*Female* (2.4 - 5.7 mg/dl)		7.5 – 10.0 mg/dl	10.1 – 12.0 mg/dl	12.1 – 15.0 mg/dl	> 15.0 mg/dl
Urea nitrogen (BUN)* (10 - 50 mg/dl)		1.25 - 2.5x ULN	2.6 - 5x ULN	5.1 - 10x ULN	>10x ULN
γ-GTP*<br>*Male* (11 - 61 U/l)<br>*Female* (9 - 39 U/l)		1.25 - 2.5x ULN	2.6 - 5x ULN	5.1 - 10x ULN	>10x ULN
Total protein, hypoproteinemia** (6.6 - 8.7 g/dl)		5.5 – 6.0 g/dl	5.0 – 5.4 g/dl	<5.0 g/dl	NA

**NIH/NIAID/DMID Toxicity Table May 2001, http://www.icssc.org/Documents/Resources/AEManual2003AppendicesFebruary_06_2003%20final.pdf
**Food and Drug Administration. Center for Biologics Evaluation and Research. September 2007. Toxicity Grading Scale for Healthy Adult and Adolescent Volunteers Enrolled in Preventive Vaccine Clinical Trials. http://www.fda.gov/cber/gdlns/toxvac.htm#iii

LABORATORY

PARAMETER	GRADE 1 MILD	GRADE 2 MODERATE	GRADE 3 SEVERE	GRADE 4 POTENTIALLY LIFE-THREATENING
**Laboratory values not otherwise specified in this table**	abnormal, but requiring no immediate intervention; for follow-up	sufficiently abnormal to require medical evaluation and perhaps mild therapeutic intervention, but not of sufficient severity to warrant immediate changes in protocol	sufficiently severe to require medical evaluation and treatment, including possible temporary suspension of trial	life-threatening severity; requires immediate medical evaluation, treatment; trial must be stopped immediately until all issues are resolved

Table 7. Urinary test results that would suggest confirmatory testing. At the discretion of the investigator, when strip test result of a subject is equivalent to any of the values reflected below, further quantitative laboratory test can be done.

**Test**<br>(Normal values are reflected in parenthesis)	**Strip Result**
**Urine color ***<br>(colorless to pale yellow)	amber to shades of red, black, green
**Urine turbidity**<br>(clear)	hazy/cloudy/turbid
**Glucose**<br>(Negative, <30 mg/dl)	2+, >100 mg/dl
**Bilirubin**<br>(Negative, 0.2 mg/dl)	1+, >1 mg/dl
**Ketone**<br>(Negative, <5 mg/dl)	1+, >5 mg/dl
**Blood**<br>(Negative)	1+
**pH, 5 to 6 (early morning urine)**<br>**up to 7.4 (daytime)**	persistently acidic (<7) or<br>persistently alkaline (>7)
**Protein**<br>(Negative)	1+, >25 mg/dl
**Urobilinogen**<br>(< 1 mg/dl)	1+, ≥1 mg/dl
**Nitrite**<br>(Negative)	positive
**Leukocytes**<br>(Negative)	1+, >20 Leu/µl
**Specific gravity**<br>(1.005-1.025)	<1.010 or ≥1.030

*by visual inspection

Table 8. Causal relationship with the test vaccine

1: Not related	A certain cause other than that attributed to the test vaccine is present, and the relation to the test vaccine can be excluded. (*e.g.*, alternative causes can be natural history of underlying diseases, concurrent therapy, etc.)
2: Unlikely	The presence of a cause other than the test vaccine is likely. A temporal association may exist, but the vaccine is not likely to have a reasonable association with the observed event (improbable but not impossible)
3: Possible	Both the test vaccine and other cause can equally be the reason of the event
4: Probably	Most causes other than the test vaccine are excluded; but definite proof of causality is not evident
5: Definitely	Event is directly caused by the test vaccine.<br>Reactions of a similar nature have been previously observed; the event has been reported.<br>All local (administration site) reactions are considered casually related to administration.

10 SUBJECT COMPLIANCE AND WITHDRAWAL

10.1 Responsibility of the Principal Investigator and Co-Principal Investigator with Regards to Subject Management

Subjects participating in the trial will be under the management of the principal investigator and co-principal investigator under the following conditions:

Trial period

The trial period is designated as the period between the days of giving consent (screening day) to final visit. When a follow-up examination is performed after the final visit schedule, the period will be prolonged to the final follow-up examination.

Health management

- Subjects will be instructed to pay attention to their health during the trial period.
- During the trial period, subjects will be instructed to avoid hard labor that they usually do not do.

Days when laboratory tests (blood sampling) are scheduled

- Subjects will be asked to fast from 22:00 of the previous day until completion of laboratory tests on the following day.
- Ingestion of foods and beverages containing alcohol will be prohibited from 22:00 on the previous day until completion of laboratory tests.
- Ingestion of foods and beverages containing caffeine (coffee, black tea, green tea, chocolate, etc.) will be prohibited from 22:00 on the previous day until completion of laboratory tests.

Days between administration/during the trial period

- Subjects will be advised to avoid excessive daily alcohol consumption.
- Subjects will be advised to limit cigarette smoking
- Subjects will be advised to avoid hard labor that they usually do not do.
- Concomitant drugs and treatments
  The use of other drugs will be prohibited throughout the trial period. When another drug or treatment is inevitably administered for incidental diseases and adverse events, name of the drug, administration method and dosage, duration of medication, reason for the use, and details of the concomitant treatment will be described and recorded.
- Bathing and shower
  Subjects can take bath and shower on administration days, but will be instructed to avoid excess stimulation to the administration site.
- Others
  i) Subjects will be instructed to pay attention to health management from 1 week before the first administration.
  ii) Investigators will attempt to contact subjects who missed scheduled trial visits.

10.2 Subject Withdrawal/Drop-out

10.2.1 Withdrawal by subject

When a subject withdraws from the trial, other than discontinuation due to adverse events (e.g. withdrawal of consent, noncompliant with protocol), the trial of the subject will be immediately discontinued. The subject is not obliged to clarify the reason for the withdrawal, but the investigators will make efforts to clarify the reason, while fully respecting the subject's right.

When a subject is withdrawn from the trial at the discretion of the investigator, the subject will be followed until resolution of the event as with any adverse event.

10.2.2 Ineligibility to be a subject

This is defined as the inability of the subject to perform necessary assessment, observation, and tests due to subject's convenience, found after initiation of the trial (e.g. migration from the area). The trial of the subject will be immediately discontinued.

10.2.3 Subject replacement

When the investigators find a subject to be ineligible (including those who withdraw from the participating the trial) after obtaining consent but before the first administration, the subject will be replaced with a reserved person who gave written consent at the trial explanation meeting (screening visit). The investigators will promptly inform it to the subject and the reserved person. This case would also apply for any female subject who will have a positive urine β-hCG (pregnant) and/or for subjects who meets any of the deferral criteria on Day 1, prior to first administration.

10.2.4 Subject drop-out

From the perspective of data analysis a "drop-out" is any subject who did not come back for the concluding visit foreseen in the protocol. A subject who returns for the concluding visit foreseen in the protocol is considered to have completed the trial. Investigators will attempt to contact subjects who missed scheduled visits.

11 DATA COLLECTION, MANAGEMENT AND ANALYSIS

11.1 Collection

11.1.1 Primary endpoints

Trial Days 1, 2, 8, 15, 22, 23, 29, 36, 43: 9 visits

- Occurrence of systemic symptoms (chills, fever, malaise)
- Occurrence of abnormalities on physical examination (including vital signs and organ systems)

- Reactogenicity assessments (administration site reactions *e.g.* pain, tenderness)
- Occurrence of any AEs or SAEs

Trial Days 1, 22, 43

- Occurrence of symptoms/abnormality on hematology tests, blood chemistry

Trial Days 1, 2, 15, 22, 23, 36, 43

- Occurrence of abnormality in urinalysis results

11.1.2 Secondary endpoints

Trial Days 1, 22, 43

- Anti-SE36 antibody titers at time points during which blood samples are taken for serology

11.2 Trial Groups/Datasets to be Evaluated

11.2.1 Total cohort

Total cohort will include all subjects in the trial.

11.2.2 Safety cohort

The safety cohort will consist of all subjects who have received at least one dose of investigational product. The presentation of safety data will explore separately the adverse events among subjects who received both administrations, and among those who receive only one.

11.2.3 Immunogenicity cohort

The immunogenicity cohort will include all subjects for whom assay results are available for antibodies against SE36 protein.

11.3 Data Management Procedures

Case report forms (CRFs) will be used to record data of subjects administered with investigational product. In addition, supplementary documents (laboratory test reports, medical records, etc) may form part of the source document. The principal investigator and co-principal investigator is responsible for the accuracy and completeness of the data reported.

11.3.1 Original documents: direct access and record keeping

Original documents include all original records of clinical findings, observation, and other activities concerning the trial and all information recorded in guaranteed copies, and are necessary for the reproduction and assessment of facts and courses of the trial

(raw data, e.g., findings of investigator-- is included in the original documents).

Original documents that will be used for preparation of case reports are shown below:
1) Allocation table
2) Forms for delivery of the investigational products
3) Record table of investigational products administration
4) Explanatory documents for obtaining consent
5) Screening source document
6) Table of symptoms
7) Record table of findings by investigator
8) Vital sign record table
9) Report of laboratory test results (chart)
10) Subject observation record (nurse record)
11) Urinalysis results
12) Others (records based on which case report will be prepared, such as temporary record table of findings and directions)

Regarding items directly described in the case report that requires judgment by an investigator (AE severity grading and causality; or when applicable, concomitant medications), the case report is handled as an original document.

The investigators and LMC will provide a direct access to all trial-related records including the original documents when needed for monitoring by the sponsor and QUINTILES; and inspection by the audit, Ethics Committee, and Regulatory Authorities.

A final report will be submitted to the sponsor after trial completion.

11.3.2 Case report form

Prior to screening, the investigator will designate and provide a list showing signature and hand-written initials of all trial staff authorized to make or change entries in the CRFs.

CRFs will be supplied by the CRO (QUINTILES) for recording all data. It is the responsibility of the principal investigator and co-principal investigator to ensure that CRFs are legible and completely filled in with a permanent ink or pen.

Errors must be corrected by drawing a single line through the incorrect entry and writing in the new value/data positioned as close to the original as possible. The correction must be initialed, dated and justified, where necessary. The original entry must not be obliterated, overwritten or erased when a correction is made.

Every effort will be made by the investigator or designated staff to complete the relevant sections of the case report as soon as feasible following a subject visit. Similarly, when a subject completes the trial, every effort will be made to complete the CRF as soon as the last data become available.

Immediately, when the subject has completed/withdrawn from the trial and the case report form is completed, the co-principal investigator will sign the conclusion pages

of the source document to confirm that they have reviewed the data and the data are completed and accurate.

Completed CRFs will be reviewed by QUINTILES. Errors detected by subsequent review may necessitate clarification or correction of errors and documentation and approval by the investigator. The investigator should assist in clarification or correction of errors detected after trial finalization within 48 hours of them being brought to the attention of the investigator.

11.4 Statistical Methods and Consideration

Estimated sample size

This phase 1b trial is not powered to detect differences between groups. Even if comparative statistics for safety variables are computed, the trial will have low power to detect anything other than very large differences in the incidence of local and general side effects between groups. This is done weighing the need to detect any possible adverse event against the need to limit the number of subjects involved for safety purposes. The samples size, however, in each group is widely accepted and used for the initial assessment of the safety, tolerance and immunogenicity of an investigational product. Based on an analysis of the human antibody responses to a number of malaria antigens that have been tested in clinical trials (including RTS,S) [Stoute et al., 1977; Saul et al., 1999], the observed coefficient of variation in the range of antibody concentrations has been found to be relatively constant at approximately 1.2-1.4. A sample size of 10 subjects per dose group would permit detection of at least five-fold difference in antibody concentration between groups using a Mann-Whitney test, assuming a significance level of 0.05. A group size of 10 subjects per dose gives 0.80 probability for detecting one or more severe adverse event.

Regarding the details of statistical analysis, a statistical analysis plan will be separately prepared before database lock, and analysis will be performed following the plan.

11.4.1 Other assessments

The assignment of subjects to analysis samples, the disposition of subjects with respect to premature termination, reason for premature termination, investigational product exposure and treatment compliance will be summarized by Stage and dose group.

Demographic characteristics (age, gender, etc) and other baseline characteristics of each cohort will be tabulated. The mean (with range and standard deviation) of the enrolled subjects, by gender, dose group, and in aggregate, will be reported.

Medical history, including coding data will be listed.

Concomitant medication, including coding data will be listed.

11.4.2 Safety

Adverse events:

An AE that starts during a unique treatment or that already exists before the start of that unique treatment but worsens during the treatment, including any post-administration period will be considered as treatment emergent for that unique treatment. The definition of treatment emergent will be based on unique treatments.

AEs will be reported on a per-subject basis, i.e. counting subjects rather than events. This means that if a subject suffers the same AEs repeatedly during the trial, the subject will be counted only once. However, the number of repeated events per subject will also be counted. Only treatment emergent AEs will be reported. In the listings, however, all occurrences of the AEs will be presented.

The overall percentage and frequency of adverse events will be tabulated. The incidence, intensity, and relationship of treatment emergent AEs (individual symptoms) per primary SOC (System Organ Class) and per PT (preferred term) by primary SOC will be calculated per cohort, age group and/or dose.

Consultation and physical examination:

Vitals sign measurements (blood pressure, pulse rate, temperature and body weight) will be presented in listings and will be summarized together with the changes from baseline.

Systemic and local events will be presented in listings and will be summarized.

Physical examination data will be listed.

Safety laboratory variables:

Hematology, blood chemistry and urinalysis results will be summarized. Out of range values will be flagged in the individual subject listing, while a separate listing will be provided for clinically significant values.

11.4.3 Efficacy

Anti-SE36 Protein

For each gender, age and dose group, anti-SE36 protein antibody titers and antibody prevalence during Screening, Day 1, Day 22 and Day 43 will be individually listed and summarized. Changes from baseline calculated per visit will be listed and summarized. Individual and mean plots for observed values will be provided.

11.4.4 Handling of cases

When data is missing, not adopted, or abnormal due to deviation from the protocol and premature termination of the trial on analysis and assessment of the primary and secondary endpoints, the sponsor will consult medical experts and the investigators, and decide how to handle the data.

Any missing, unused or spurious data, or previous reports of any deviations from the

original statistical plan, will be cited and accounted for in the appropriate analysis as they become apparent to investigators or statisticians.

11.5 Retention of Records

Documents concerning this trial, such as medical records, test data, Ethics Committee review records, trial request/contract documents, records concerning consent of subjects, and investigational products management table, will be appropriately retained by the person responsible for record keeping appointed by the principal investigator. The documents will be retained until the later date of (1) and (2) below, and retention later than the date will be decided based on consultation. The sponsor will inform the head of the medical institution, in writing, with acquisition of approval or discontinuation:

1) Five years after final marketing approval of the test vaccine (when discontinuation of the development is informed of, 3 years after the decision of the discontinuation)
2) Three years after the discontinuation or completion of the trial.

The sponsor is required to inform the principal investigator as to when such documents need no longer be retained. Storage of all trial-related documents will be such that confidentiality will be strictly maintained.

Documents concerning this trial, such as the protocol, case reports, clinical reports, trial contract documents, and quality test results of the test vaccine, will be retained by the sponsor. Likewise, the documents will be retained until the later date of (1) and (2) above.

12 QUALITY CONTROL AND QUALITY ASSURANCE

SOPs and appropriate forms for quality management will be in used and kept on file for all appropriate personnel. GCP and trial specific trainings to cover aspects of conduct, monitoring, auditing, recording, analyses, and reporting of clinical trials will be conducted at LMC and records/documentation of training will be kept on file. To confirm the conformity of the conduct, data preparation, recording, and reporting of this trial with the protocol and the:

"*Departmental Regulations for Standards of Implementation of Clinical Trial with Drugs and Medicines (GCP),*" (Japan Ministry of Health and Welfare Ordinance No. 28, March 27, 1997);
"Departmental Regulations for Revised Standards of Implementation of Clinical Trial with Drugs and Medicines (GCP)," (Japan Ministry of Health, Labour and Welfare Ordinance No. 106, June 12, 2003);
"*National Guidelines for Research Involving Humans as Research Participants,*" Ugandan National Council for Science and Technology (UNCST) (Kampala, Uganda; March 2007);
"*Guidelines for the Conduct of Clinical Trials,*" National Drug Authority (NDA) (Kampala, Uganda; December 2007);
and ICH Guidelines for clinical Trials E6 (R1) CPMP/ICH/135/95 (updated 2002);

the sponsor and QUINTILES will perform quality control assessments/monitoring at any time during the trial period. Data will be evaluated for compliance with protocol and accuracy in relation to source documents.

The trial will be conducted at a single site, LMC, with the exception of ELISA studies which will be conducted at MBL and BIKEN. Not to be used as endpoints but as research outputs, biological activity assays (GIA, ADCI, epitope mapping) will be conducted at BIKEN, RIMD and/or Pasteur Institut.

All records will be made available to monitors, including regulatory files, CRFs and other source documents, SOP and quality assurance documentation. Quality control checks will also be done on data entry and any missing data or data anomalies will be communicated to MBL and LMC for clarification/resolution.

13 ETHICAL CONSIDERATIONS

13.1 Statement of Compliance and Ethical Reviews

Preparation and manufacturing of BK-SE36 is in accordance with the 'Standards for Manufacturing Control and Quality Control of Investigational Products, and Standards for Buildings and Facilities of Manufacturing Plants for Investigational Products (GMP for Investigational Products)' (Pharmaceutical Affairs Bureau Notification No. 480 dated March 31, 1997); Otsuka Physiological Saline is manufactured in accordance with the Pharmaceutical Affairs Law of Japan (Standard Commodity Classification No. 873311).

This trial will be carried out in accordance with the ICH HARMONISED TRIPARTITE GUIDELINE FOR GOOD CLINICAL PRACTICE E6(R1) (ICH-GCP) standards and regulatory authorities' requirements. The protocol is based on the ethical principles stated in the *Declaration of Helsinki*: "Recommendations Guiding Medical Doctors in Biomedical Research Involving Human Subjects," adopted at the General Assembly of the World Medical Association held in Helsinki in 1964 (revised 2000 at Edinburgh, Scotland).

BIKEN-IRC, RIMD-IRC and MBL-IRC will review and approve the protocol prior to submission to regulatory authorities, UNCST and NDA. The decision(s) concerning this protocol and the conduct of the trial will be made in writing and kept on file.

13.2 Institutional Review Committee

The investigator should not implement any deviation from, or changes in this protocol without agreement with the sponsor, and prior review and documented approval/favorable opinion from the MBL-IRC and regulatory bodies, except when necessary to eliminate immediate hazards to subjects.

Any amendment/modification to the protocol will be adhered to, by and will apply to all subjects. Written MBL-IRC, UNCST and NDA approvals of protocol amendments are required prior to implementation.

The principal investigator and co-principal investigator will inform the IRC, UNCST and NDA of the following:

- All subsequent protocol amendments, informed consent changes or revisions
- Serious and/or unexpected adverse events occurring during the trial
- New information, including any provided by the sponsor, that may affect adversely the safety of the subjects, or the conduct of the trial
- When the trial has been completed

13.3 Other Committees

13.3.1 Community Advisory Board

A Community Advisory Board (CAB) will be established by the study investigators. CAB will consist of community leaders and representatives of the study population. The board will serve as forum for facilitating dialogue between community members, study volunteers and investigators and will be tapped, most especially in sensitizing the community for the trial during the informed consent process and in achieving successful volunteer recruitment and retention.

13.3.2 Data and Safety Monitoring Board

A Data and Safety Monitoring Board will be assembled by the sponsor. Interpretation of results and decisions about discontinuation of the trial will be made by the members of the DSMB. The principal investigator is responsible for ensuring that DSMB is aware of all new safety information. In addition, the board will be provided with the following: (1) prompt reports of SAE occurring during the trial; (2) protocol amendments/modifications, informed consent changes; and (3) any new information that may adversely affect the safety of the subjects or conduct of the trial. 30±5 days after the second administration in Stage 1, the principal investigator and co-principal investigator, will submit a tabulated, summarized (and verified by the CRO) safety assessment of adverse events observed; test results and comments, as needed. The incidence and intensity of all symptoms over 21 days post administration (43 days of safety data) will be evaluated per subject. Data from the following tests will be included: vital signs, systemic events, local events, hematology, blood chemistry and urinalysis. This report will form the basis for decisions to proceed to Stage 2. The trial will not proceed to the next stage unless explicitly agreed to by a majority of the members.

Interim/cumulative data for the secondary endpoint (immune response to BK-SE36) will not be provided to the board as the sponsor does not believe that there will be grounds for stopping the trial on the basis of antibody induction against SE36 protein.

13.4 Informed Consent

The principal investigator, in cooperation with the sponsor, will prepare (and revise, as needed) explanatory documents to obtain consent for participation in this trial. The prepared or revised documents will be in conformity with GCP guidelines and regulatory regulations referred above, and subject to approval by the Ethics Committee and the regulatory authorities before use.

Consent forms will be translated to Luo and Swahili and will be translated back to English by an independent translator without reference to the original text. The original and back-translated versions will be compared side-by-side for verification. Impartial witnesses will also be used to attest that volunteers who cannot read or write have understood the contents of the informed consent.

13.5 Risks and Potential Benefits

13.5.1 Administration

Risks associated with any administration include local inflammatory reactions, such as administration site pain, swelling and some limitation of arm movement. Systematic effects may also include flu-like symptoms: fever, chills, nausea, headache, malaise, myalgia and arthralgia. To date, all symptoms associated with BK-SE36 have been transient, mainly mild to moderate in intensity. While exceedingly rare, allergic reactions, to include life-threatening anaphylaxis, are associated with some vaccine preparations and must therefore be considered as potential risk.

Risks associated with drawing blood include fainting, infection and bruising.

As outlined above, the subjects will be monitored closely during the trial. The investigational products will be administered by experienced investigators trained in GCP, with drugs and equipments available for the treatment of anaphylaxis.

13.5.2 Precautions

The effect of BK-SE36 on the unborn fetus is unknown. Female subjects (adolescents/adults) will be cautioned and advised to exercise adequate birth control methods until final visit (21 days after second administration).

Female subjects who became pregnant during the trial will be instructed to notify the investigators if they become pregnant. Although not considered as an adverse event, pregnancy that occurs after first administration of the test vaccine will be reported in the same way as an adverse event and, with permission from the subject, followed to term. Any premature termination will be reported, and the health status of the mother and child including date of delivery and the child’s gender and weight will be reported in the Pregnancy Report and Outcome Form.

13.5.3 Benefits

Subjects will not receive any direct benefit from participation in this trial. It is hoped that information gained will contribute to the development of a safe and effective malaria vaccine.

13.5.4 Medical treatment for subjects

In the event of a medical emergency, the investigators shall perform any medical procedures that are deemed medically appropriate.

Free medical treatment will be provided for all subjects during the trial stages and clinic visits. Medical care for ailments not related to trial intervention will not extend beyond the trial period. Medical care for ailments related to trial intervention will extend, at minimum, until the condition has resolved or stabilized.

13.5.5 Payment

Subjects will be paid via LMC based on Ugandan standards. Compensation will cover for time spent, meal(s) and travel costs at each trial visit. Details will be available in the ICD [*Informed consent documents*].

13.5.6 Compensation for health problems of subjects

The sponsor is legally responsible for health problems due to administration and death of subjects concerning this trial, excluding the cases described below, for which LMC will take the responsibility:

1) Noncompliance with the protocol
2) Noncompliance with the relevant legal regulations
3) Carelessness and illegal acts

When a problem or a lawsuit occurs, the investigators will inform it to the sponsor.

When this trial induces a serious adverse event, and compensation by LMC is necessary, the sponsor will take the whole responsibility, excluding those for which the responsibility (items 1-3 above) should be held by the medical institution.

14 CONFIDENTIALITY

Subjects will be assigned a unique subject code. All results will be linked to this number. Furthermore, all laboratory samples will be identified by coded number only to maintain subject confidentiality. Trial records will only be available to staff members and will be kept in locked cabinets at the trial site. Access to files may be reviewed by representatives from Sponsor, QUINTILES, MBL-IRC, UNCST and NDA, for

monitoring audit and inspection. Information acquired during monitoring will not be leaked to unrelated persons.

15 FINANCING AND INSURANCE

This trial will be financed by The Research Foundation for Microbial Diseases of Osaka University (BIKEN). An agreement for cooperation with MBL will be sought prior to trial start for additional financial support in the form of rental fee payments for Apac and Kampala MBL laboratories, equipment procurement and ELISA test run procedures/SOP validation. Major clinical and research trial cost will be provided by BIKEN through an independent contract research organization. BIKEN has outsourced the specialty services of QUINTILES. The sponsor also has, and will maintain from screening to last subject out, a clinical trial liability insurance policy sufficient to cover the cost of reasonable medical care required to treat or stabilize adverse reactions suffered by subjects who were administered with BK-SE36 in accordance with the approved protocol.

16 PUBLICATION POLICY

For publication of the results obtained in this trial in academic meetings and journals, the data to be presented and the authorship will be discussed between the investigators and sponsor, and approved by the sponsor prior to any official communication. This allows the sponsor to protect proprietary information and to provide comments based on information from other studies that may not yet be available to the authors of the manuscript. The principal investigator shall submit to UNCST a copy of the prepared manuscript or publication arising from this clinical trial. UNCST reserves the right to review the manuscript or publications and provide their comments for the author's consideration.

17 REFERENCES

[1] Aoki S., Li J., Itagaki S., Okech B.A., Egwang T.G., Matsuoka H., Palacpac N.M., Mitamura T., Horii T. 2002. Serine repeat antigen (SERA5) is predominantly expressed among the SERA multigene family of *Plasmodium falciparum* and the acquired antibody titers correlate with serum inhibition of the parasite growth. *J Biol Chem.* **277**(49): 47533-47540.

[2] Cody C.L., Baraff L.J., Cherry J.D., Marcy S.M., Manclark, C.R. 1981. Nature and rates of adverse reactions associated with DTP and DT immunizations in infants and children. *Pediatrics* **68**: 650-660.

[3] Debrant A., Maes P., Delplace P., Dubremetz J.F., Tartar A., Camus D. 1992. Intramolecular mapping of *Plasmodium falciparum* P126 proteolytic fragments by N-terminal amino acid sequencing. *Mol. Biochem. Parasitol.* **53**: 89–96.

[4] Doherty J.F., Pinder M., Tornieporth N., Carton C., Vigneron L., Milligan P., Ballou W.R., Holland C.A., Kester K.E., Voss G., Momin P., Greenwood B.M., McAdam

K.P.W.J., Cohen J. 1999. A phase I safety and immunogenicity trial with the candidate malaria vaccine RTS,S/SBAS2 in semi-immune adults in The Gambia. *Am. J. Trop. Med. Hyg.* **61**: 865-868.

[5] Egwang T.G., Apio B., Riley E., Okello D. 2000. *Plasmodium falciparum* malariometric indices in Apac district, northern Uganda. *East Afr Med J* **77**: 413–416.

[6] Fox B.A. and Bzik D.J. 1994. Analysis of stage-specific transcripts of the *Plasmodium falciparum* serine repeat antigen (SERA) gene and transcription from the SERA locus. *Mol. Biochem. Parasitol.* **68**: 133–144.

[7] Georgitis J.W., Fasano M.B. 2001. Allergenic components of vaccines and avoidance of vaccination-related adverse events. *Curr Allergy Rep* **1**:11-17.

[8] Goto N. and Akama K. 1984. Local histopathological reactions to aluminum-adsorbed tetanus toxoid. *J. Naturwissenschaften* **71**(8): 427-428.

[9] Li J., Mitamura T., Fox B.A., Bzik D.J., Horii T. 2002. Differential localization of processed fragments of *Plasmodium falciparum* serine repeat antigen and further processing of its N-terminal 47 kDa fragment. *Parasitol. Int.*, **51**(4): 343-352.

[10] Merck and Co., Inc. 2003. Recombivax HB® Product Insert. Whitehouse Station, NJ.

[11] Nosten F., Luxemburger C., Kyle D.E., Ballou W.R., Wittes J., Waha E., Chongsuphajaisiddhi T., Gordon D.M., White N.J., Sadoff J.C., Heppner D.G., Shoklo SPf66 Malaria Vaccine Trial Group. 1996. Randomised double-blind placebo-controlled trial of SPf66 malaria vaccine in children in northwestern Thailand. *Lancet* **348**:701-707.

[12] Okech B.A., Nalunkuma A., Okello D., Pang X.L., Suzue K., Li J., Horii T., Egwang T.G. 2001. Natural human immunoglobulin G subclass responses to *Plasmodium falciparum* serine repeat antigen in Uganda. *Am J Trop Med Hyg* **65**:912–917.

[13] Okech B., Mujuzi G., Ogwal A., Shirai H., Horii T., Egwang T.G. 2006. High titers of IgG antibodies against *Plasmodium falciparum* serine repeat antigen 5 (SERA5) are associated with protection against severe malaria in Ugandan children. *Am J Trop Med Hyg* **74**:191-197.

[14] Ockenhouse C.F., Angov E., Kester K.E., Diggs C., Soisson L., Cummings J.F., Stewart A.V., Palmer D.R., Mahajan B., Krzych U., Torneiporth N., Delchambre M., Vanhandenhove M., Ofori-Anyinam O., Cohen J., Lyon J.A., Heppner D.G, MSP-1 Working Group. 2006. Phase I safety and immunogenicity trial of FMP1/AS02A, a *Plasmodium falciparum* MSP-1 asexual blood stage vaccine. *Vaccine* **24**: 3009-3017.

[15] Okello P.E., Bortel W.V., Byaruhanga A.M., Correwyn A., Roelants P., Talisuna A., D'Alessandro U., Coosemans, M. 2006. Variation in malaria transmission intensity in seven sites throughout Uganda. *Am. J. Trop. Med. Hyg.* **75**: 219-225.
[16] Pang X.L., Mitamura T., Horii T. 1999. Antibodies reactive with the N-terminal domain of *Plasmodium falciparum* serine repeat antigen inhibit cell proliferation by

agglutinating merozoites and schizonts. *Infect. Immun.* **67**: 1821–1827.

[17] Saul A., Lawrence G., Smillie A., Rzepczyk C.M., Reed C., Taylor D., Anderson K., Stowers A., Kemp R., Allworth A., Anders R.F., Brown G.V., Pye D., Schoofs P., Irving D.O., Dyer S.L., Woodrow G.C., Briggs W.R.S., Reber R., Sturchler D. 1999. Human phase 1 vaccine trials of 3 recombinant asexual stage malaria antigens with Montanide® ISA 720 adjuvant. *Vaccine* **17**:3145-3159.

[18] Stoute J.A., Slaoui M., Heppner D.G., Momin, P., Kester K.E., Desmons P., Wellde B.T., Garçon N., Krzych U., Marchand M., Ballou W.R., Cohen J.D. 1997. A preliminary evaluation of a recombinant circumsporozoite protein vaccine against *Plasmodium falciparum* malaria. RTS,S Malaria Vaccine Evaluation Group. *N. Engl. J. Med.* **336**: 86-91.

[19] Talisuna A.O., Langi P., Mutabingwa T.K., Marck E.V., Speybroeck N., Egwang T.G., Watkins W.W., Hastings I.M., D'Alessandro U. 2003. Intensity of transmission and spread of gene mutations linked to chloroquine and sulphadoxine-pyrimethamine resistance in falciparum malaria. *Int. J Parasitol.* **33**: 1051-1058.

[20] Thera M.A., Doumbo O.K., Coulibaly D., Diallo D.A., Kone A.K., Guindo A.B., Traore K., Dicko A., Sagara I., Sissoko M.S., Baby M., Sissoko M., Diarra I., Niangaly A., Dolo A., Daou M., Diawara S.I., Heppner D.G., Stewart V.A., Angov E., Bergmann-Leitner E.S., Lanar D.E., Dutta S., Soisson L., Diggs C.L., Leach A., Owusu A., Dubois M.C., Cohen J., Nixon J.N., Gregson A., Takala S.L., Lyke K.E., Plowe, C.V. 2008. Safety and immunogenicity of an AMA-1 malaria vaccine in Malian adults: Results of a phase 1 randomized controlled trial. *PLos ONE* **3**: e1465.

[21] Ulmer J.B., Valley U., Rappuoli R. 2006. Vaccine manufacturing: challenges and solutions. *Nature Biotech.* **24**: 1377-1383.

WWW RESOURCE LINKS

World Malaria Report 2005. Roll Back Malaria, World Health Organization and UNICEF. *http://www.rbm.who.int/wmr2005/html/map3.htm*

Lira and Apac District information site: http://www.apac.go.ug/

Gissel L.E. 2008. Apac has the highest rate of malaria in the world. But from here it gets difficult…. http://apacintheworld.wordpress.com/

WHO Toxicity Grading Scale for determining the severity of Adverse Events
http://www.icssc.org/Documents/Resources/AEManual2003AppendicesFebruary_06_2003%20final.pdf

Guidance for Industry. Toxicity Grading Scale for Healthy Adult and Adolescent Volunteers Enrolled in Preventive Vaccine Clinical Trials.
2007. http://www.fda.gov/cber/gdlns/toxvac.htm#iii

SUPPLEMENTAL REFERENCE 1.

The standard and acceptable ranges of laboratory test values for Stage 2.

["Children" is defined as 6-17 years old; "Adult" is defined as 18-20 y old]

[shaded areas denote that the acceptable range was derived from LMC reference ranges]

Acceptable vital sign ranges:

Age	Blood pressure (mmHg)	
	Systolic	Diastolic
6 - 15 yrs	80-130	65-80
16 - 20 yrs	90-139	55-89
>20 y old	95-139	55-89

Age	Pulse rate Normal Range (HR/min)		
	2%	Mean	98%
5 - 7 yrs	65	100	133
8 -11 yrs	62	91	130
12 - 15 yrs	60	85	119
16 – 20 yrs	55	78	100
> 20 yrs	60		100

Hematology ranges:

	Normal Value		Protocol description	Screening normal value
Leukocyte count (WBC)	Adult: 4.0-10.0	$\times 10^9$/L	Within 25% deviations from the upper and lower limits of the baseline range. The differential white blood count is not questioned when the white blood cell count is within the baseline range.	3.0-12.5
	Children: 4.0 - 13.5			3.0-16.9
Erythrocyte count (RBC)	Male: 4.50-5.50	$\times 10^{12}$/L		3.38-6.88
	Female: 4.00-5.00			3.00-6.25
	Children: 3.30 – 5.40			2.48-6.75
Hemoglobin	Male: 14.0-17.4	g/dL		10.5-21.8
	Female: 12.0-16.0			9.0-20.0
	Children: 9.5-14.8			7.1-18.5
Hematocrit(HCT)	Male: 45.0-52.0	%		33.8-65.0
	Female: 36.0-48.0			27.0-60.0
	Children: 27.0 - 44.0			20.3-55.0
Platelet count(PLT)	130.0 - 450.0	$\times 10^9$/L		97.5-562.5

Biochemistry ranges:

	Normal Value		Protocol description	Screening normal value
Total protein	6.6-8.7	g/dL	Within 25% deviation from the upper and lower limits of the baseline range.	5.0-10.9
Albumin	3.80-5.10	g/dL		2.85-6.38
Total bilirubin	up to 1.2	mg/dL	Within 50% deviation from the upper limit.	up to 1.8
AST	Male: up to 37.0	U/L	Within the baseline range	up to 37.0
	Female: up to 31.0			up to 31.0
ALT	Male: up to 42	U/L		up to 42
	Female: up to 32			up to 32
AL-P	Male: up to 306.0	U/L	Within 25% deviation from the upper and lower limits of the baseline range.	up to 382.5
	Female: up to 306.0			
	6-15y: up to 644.0			up to 805.0
	15-17y: up to 483.0			up to 603.8
γ-GTP	Male: up to 61.0	U/L		up to 76.3
	Female: up to 39.0			up to 48.8
Serum amylase	up to 90.0	U/L		up to 112.5
Total cholesterol	up to 30y: up to 180.0	mg/dL		up to 225.00
	above30y: up to 200.0			up to 250.00
Uric acid	Male: 3.4-7.0	mg/dL		2.6-8.8
	Female: 2.4-5.7			1.8-7.1
Urea nitrogen	Adult: 10.0-50.0	mg/dL		7.50-62.50
	Children: 7.0-22.0			5.3-27.5
Creatinine	Male: 0.6-1.1	mg/dL	Within the baseline range	0.6-1.1
	Female: 0.5-0.9			0.5-0.9
Na	135.0-155.0	mmol/L		135.0-155.0
K	3.60-5.50	mmol/L		3.60-5.50
Glucose	random blood: 70.00-100.00	mg/dL	Within 25% deviation from the upper and lower limits of the baseline range.	52.50-125.00
	fasting blood: 75.00-115.00			56.25-143.75

References;
1. Sitefano B.Tugume et al; Hematological reference ranges among healthy Ugandans; Clinical and diagnostic laboratory Immunology, Mar. 1995, p. 233 - 235
2. LeighAnne Eller et al; Reference intervals in healthy Adult Ugandan blood donors and their impact on conducting international vaccine trials; PloS312):e3919.doi:10.1371/journal.pone.0003919, 2008
3. LMC reference ranges
4. MUWRP lab reference ranges and MU-UCSF SOP for vital signs

18 APPENDICES

18.1 Flow chart of all trial stages from regulatory approval to post trial follow-up

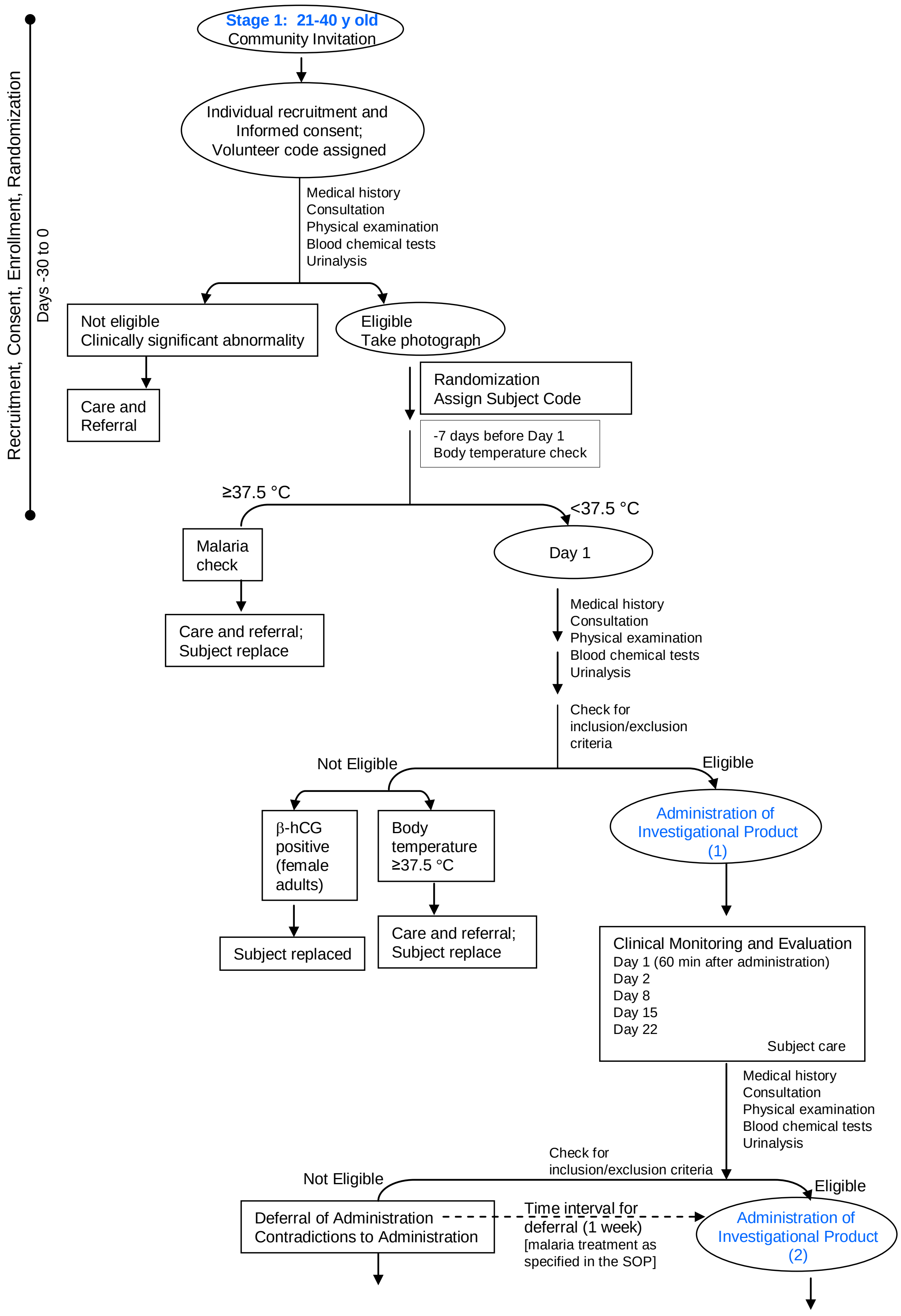

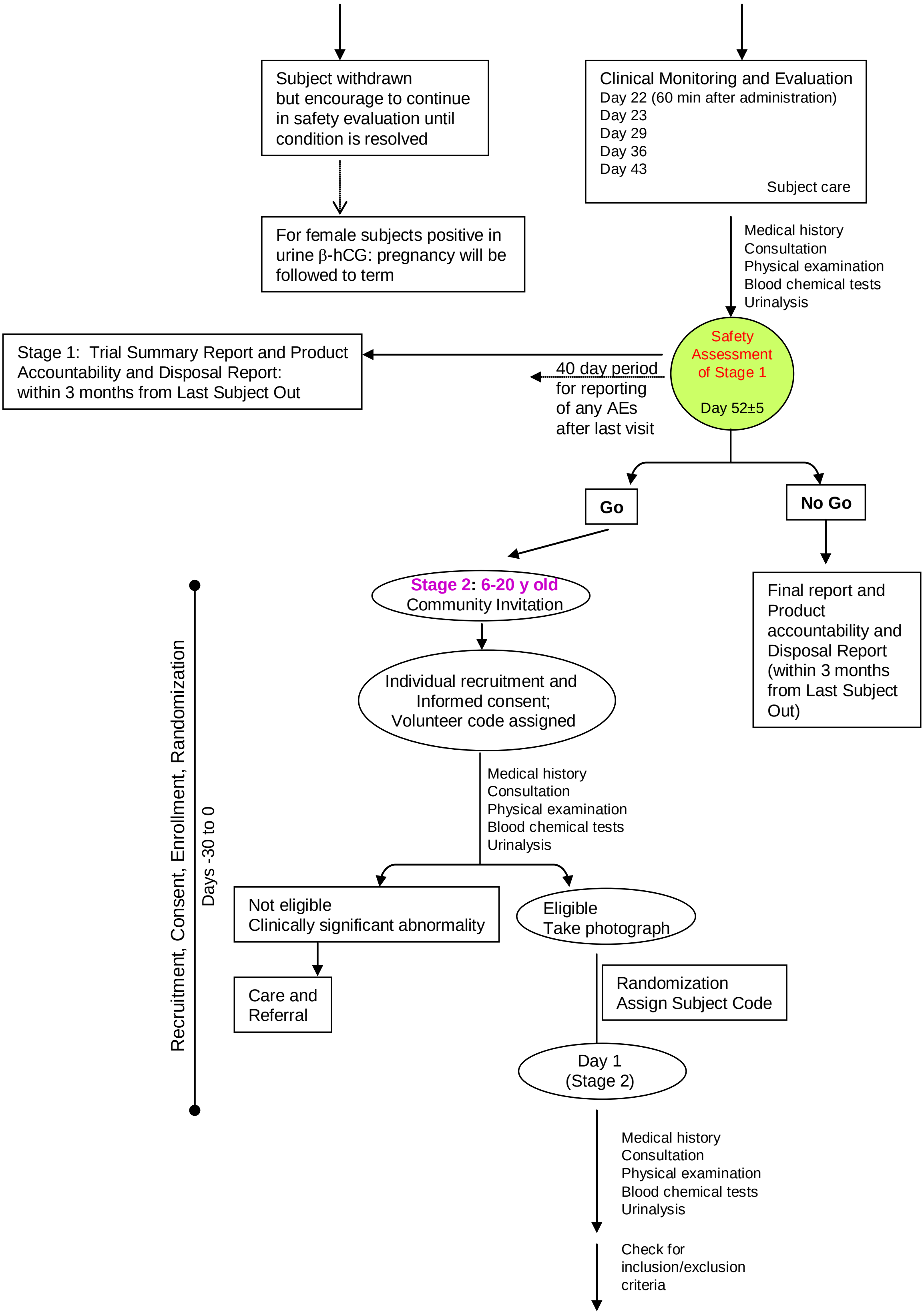
Subject withdrawn but encourage to continue in safety evaluation until condition is resolved
For female subjects positive in urine β-hCG: pregnancy will be followed to term
Clinical Monitoring and Evaluation
Day 22 (60 min after administration)
Day 23
Day 29
Day 36
Day 43
Subject care
Medical history
Consultation
Physical examination
Blood chemical tests
Urinalysis
Safety Assessment of Stage 1
Day 52±5
Stage 1: Trial Summary Report and Product Accountability and Disposal Report: within 3 months from Last Subject Out
40 day period for reporting of any AEs after last visit
Go
No Go
Final report and Product accountability and Disposal Report (within 3 months from Last Subject Out)
Stage 2: 6-20 y old
Community Invitation
Individual recruitment and Informed consent; Volunteer code assigned
Recruitment, Consent, Enrollment, Randomization
Days -30 to 0
Medical history
Consultation
Physical examination
Blood chemical tests
Urinalysis
Not eligible
Clinically significant abnormality
Care and Referral
Eligible
Take photograph
Randomization
Assign Subject Code
Day 1
(Stage 2)
Medical history
Consultation
Physical examination
Blood chemical tests
Urinalysis
Check for inclusion/exclusion criteria

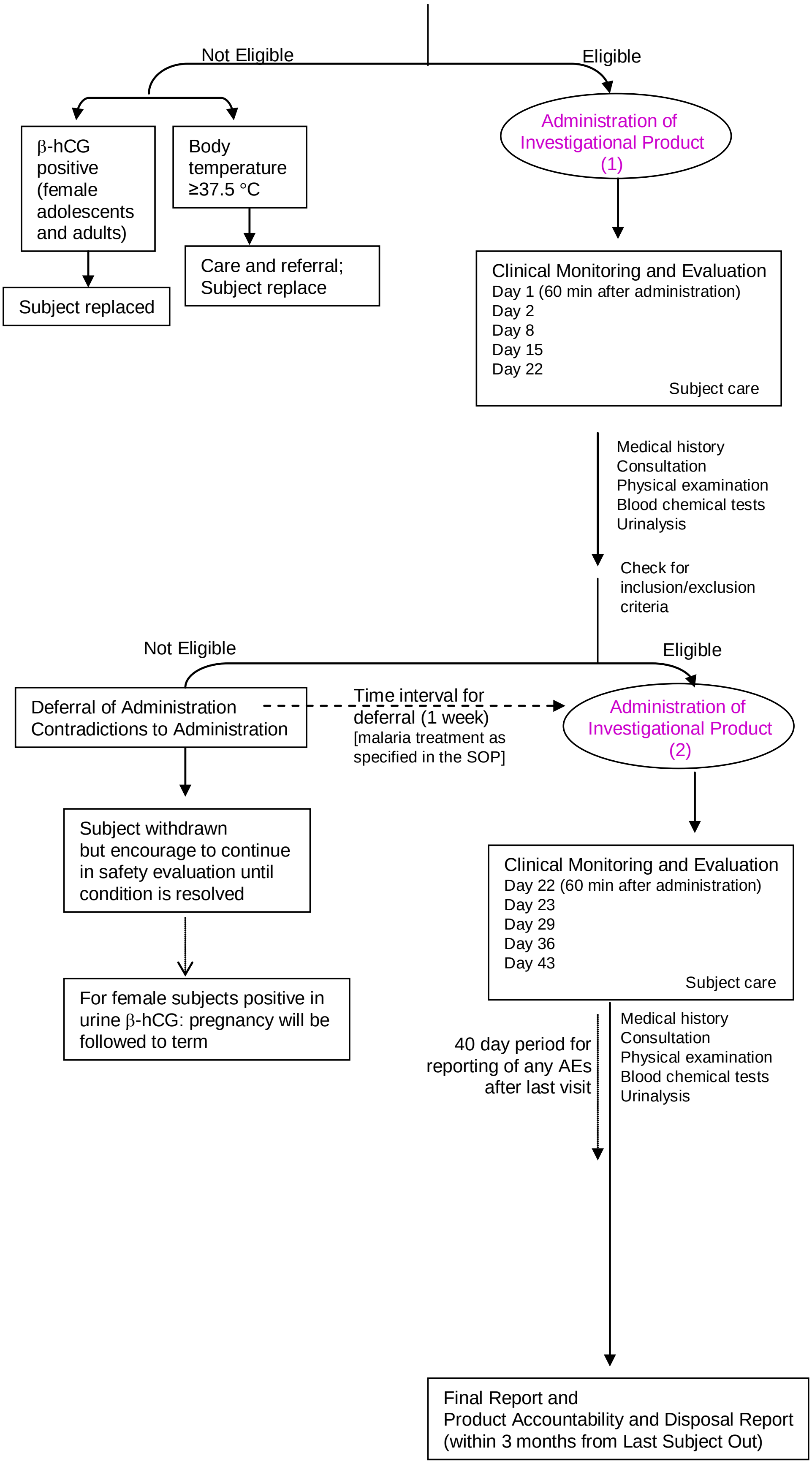
Not Eligible
Eligible
β-hCG positive (female adolescents and adults)
Body temperature ≥37.5 °C
Administration of Investigational Product (1)
Subject replaced
Care and referral; Subject replace
Clinical Monitoring and Evaluation
Day 1 (60 min after administration)
Day 2
Day 8
Day 15
Day 22
Subject care
Medical history
Consultation
Physical examination
Blood chemical tests
Urinalysis
Check for inclusion/exclusion criteria
Not Eligible
Eligible
Deferral of Administration
Contradictions to Administration
Time interval for deferral (1 week)
[malaria treatment as specified in the SOP]
Administration of Investigational Product (2)
Subject withdrawn but encourage to continue in safety evaluation until condition is resolved
Clinical Monitoring and Evaluation
Day 22 (60 min after administration)
Day 23
Day 29
Day 36
Day 43
Subject care
For female subjects positive in urine β-hCG: pregnancy will be followed to term
40 day period for reporting of any AEs after last visit
Medical history
Consultation
Physical examination
Blood chemical tests
Urinalysis
Final Report and
Product Accountability and Disposal Report
(within 3 months from Last Subject Out)

18.2 Informed Consent Forms

18.2.1 ICF for Stage 1: Adults (21 to 40 y old)

TITLE: **Single blind, randomized, controlled, Phase 1b trial of the safety and immunogenicity of lyophilized recombinant precipitated tropical malaria vaccine (BK-SE36), in Uganda**

INSTITUTIONS:
1. Lira Medical Centre (LMC), Lira, Uganda
2. Med Biotech Laboratories (MBL), Kampala, Uganda
3. The Research Foundation for Microbial Diseases of Osaka University (BIKEN), Japan
4. Research Institute for Microbial Diseases (RIMD), Osaka University, Japan

PRINCIPAL INVESTIGATOR:

Thomas Egwang, PhD
Director General/Scientific Director
Med Biotech Laboratories, Plot 3438 Muyenga, Tank Hill By-pass,
PO Box 9364, Kampala, Uganda *Tel* +254-712-504 010; +256-775-494 752

CLINICAL TRIAL SITE:

Lira Medical Centre
P.O. Box 1075, Plot #15, Moroto Road, Lira, Uganda
Tel +256-392-948 833; +256-772-419 397
(c/o Dr. Ogwang Ochoo Ben, Director, LMC)
http://www.lira-medical-centre.org/

MBL-IRC:

Professor Edward K. Kirumira (Chairman)
Dean, Faculty of Social Science, Makerere University, Kampala
Tel +256 752 767 439
kirumira@starcom.co.ug

Flavia Zalwango K (Secretary)
Plan International, Uganda,
Tel +256 782 094 273
flavia.zalwango@plan-international.org, zaly14@yahoo.com

(ADULTS: 21 TO 40 Y OLD)

PARTICIPANT INFORMATION:

We would like to invite you to participate in a medical research trial. It is important that you understand the following information that applies to all volunteers in our studies:

1. Participation is entirely voluntary
2. If you refuse to take part in this trial it will not affect your current or future medical care in LMC
3. You may withdraw from participation at anytime
4. Your refusal to participate will involve no penalty or loss of benefits to which you are otherwise entitled. If you decide to withdraw from the trial later, please inform any member of the trial staff or meet with the principal investigator
5. After this explanation, please feel free to ask anything you do not understand
6. During participation in the trial, you will be informed of any new findings that may affect your willingness to participate
7. It would be advisable for someone with HIV not to participate in the trial.

INTRODUCTION/PURPOSE:

Malaria is a disease that affects many people throughout the world, including Uganda. It is caused by parasites that are transmitted by mosquito bites. Inside the human body, the parasites multiply in the liver, and then infect red blood cells. Symptoms of malaria include fever, headache and vomiting, and if not treated can quickly become life-threatening. In many parts of the world, the parasites have developed resistance to a number of malaria medicines.

Investigators at the Research Institute for Microbial Diseases in collaboration with The Research Foundation for Microbial Diseases of Osaka University have developed an experimental vaccine against malaria called BK-SE36. BK-SE36 contains a protein derived from malaria parasites that can prevent people from getting sick with malaria but it is not approved yet for general use. Normally, before a vaccine can be sold to market, it has to be tested in several clinical trials to see the safety and the ability of the vaccine to work (or its effect on the body). These trials are done with extreme care/concerns, strictly following guidelines to be able to evaluate thoroughly the new vaccine. Preliminary tests of this vaccine in 30 adults in Japan have shown that this vaccine is safe. No serious adverse events were observed and all adverse events were resolved. The predominant adverse event has been local swelling, although, erythema and induration at the administration site has been noted. The events were all mild and remitted. Longest onset of adverse event (induration) was at 43 days after the third administration. There was one subject that had the longest adverse event (induration), which was observed 4 days post administration and resolved 60 days after, without therapy or limitation of daily activity. In this trial, we would like to test BK-SE36 vaccine in adults in Uganda to make sure that it is safe to people who have already been exposed to malaria. To do this, we will compare its safety to physiological saline, a proven safe medicine used for intravenous infusion.

This trial has been approved by the MBL-Institutional Review Committee (MBL-IRC). MBL-IRC is an independent committee in Uganda that will ensure the protection of people's right, safety and well-being in this trial. We hope that information from this trial can be used to develop a vaccine that in the future will help protect people from getting sick with malaria.

PROCEDURES TO BE FOLLOWED:

If you will participate in this research trial, we will set a date for you to be back for an appointment with one of the doctors. During that appointment (Screening day):
1. we will ask you for medical history; and
2. ask you to undergo a medical examination. We will do a physical examination and do tests on blood and urine to look for any signs of illness in the blood, kidneys or liver. We will draw about 2.6 teaspoons of blood (13 ml) to evaluate your health condition. You will be told of all the test results and the possible meaning of these results. If we find anything wrong that will not allow you to participate in the trial, we will refer you to the other doctors at LMC.

If your results are good, and if you agree to continue, you will be scheduled to receive 2 doses of either the experimental vaccine BK-SE36 or physiological saline at a later date. Prior to receiving the first dose of vaccine, a picture will also be taken for file. The photograph will only be used for identification purposes. You will also be given a "Schedule Management" booklet that you will need to bring every time you visit LMC. We may also invite you a week before the scheduled vaccination to check your health for any signs of malaria. On the day of vaccination, we will again examine you including checks for blood and urine.

You will not know which vaccine you will get until the end of the trial. Which vaccine you get will be determined by chance, like when you toss a coin and you do not know whether it will land on the side with a face or on the other side. This is done to make sure that there will be no bias for evaluations.

Once the first dose (1.0 ml) of vaccine is given, the second dose (1.0 ml) will be given about 21 days later. Vaccination will be by subcutaneous injection on the upper arm. After each vaccination, you will be asked to stay at LMC for 60 minutes for further observation. We will also ask you to come back again after 1, 7, 14 and 21 days after each vaccination (a total of 9 clinic visits excluding screening days). These visits will allow us to see you, to know how you are feeling and for brief medical examination. In addition, at any time during 40 days after visit 10, you are invited to go to LMC whenever you are sick. The doctor will determine if it is due to vaccination and will record all symptoms.

During the trial, we will need to draw blood for a total of four times. This will be done to make sure you remain healthy and to measure the effect of the vaccine. Some of the blood samples obtained will be stored to do other tests that will help us see the effect of the vaccine. We need a separate consent for this. The total amount of blood will be about 3.5 tablespoons (or 10.4 teaspoons= 52 ml). If you become ill, we may need to draw additional blood to run further tests. If you become ill from malaria, we also have to prick to confirm diagnosis before we do treatment.

POTENTIAL RISKS AND DISCOMFORTS

1. Blood sampling. Drawing blood might cause discomfort and occasional bruising at the site. The amount of blood to be removed will be too small to affect your health.

2. Vaccination side-effects. For vaccination you should expect soreness, swelling, and in some cases, redness at the site of injection. In addition, muscle soreness, itching, irritability or fussiness, low-grade fever, fatigue, headache, nausea and dizziness are possible. Some days of local pain and limitation in arm movement may also be observed. These are usually transient and will disappear in time. If any of these symptoms occur, we may give you medication to help provide relief. As with any vaccine, there is a small possibility that you may have an allergic reaction. These may be mild, such as rash, or may be severe and life threatening. A life threatening reaction is extremely rare; but medicine and equipment will be available at LMC.

   There may be other reaction(s) to the malaria vaccine that at this time is unknown. If new information about the safety of the vaccine becomes available, we will do our best to inform you at once.

   For any vaccine side effects, treatment will be provided until, at least, all symptoms are resolved.

3. The risks of donating urine are minimal.

4. For female volunteers. Adolescents and adults are cautioned of the unknown risk of BK-SE36 to the unborn child and will be advised to use adequate birth control methods during participation in the trial. If you are interested in birth control methods we will refer you for consultation at LMC.

   A pregnancy test will be done on your urine during screening and before each vaccination. If the pregnancy test becomes positive before the trial starts (before your first vaccination), you will not be included in this trial. If you become positive after the first vaccination, we will no longer give you any vaccine. However, we will continue to check to see if you are well. With your permission we will follow your pregnancy to term. Your health and your child's health status including date of delivery and child's gender and weight will be noted.

BENEFITS:

1. For now, you may not have any direct benefit. BK-SE36 is a candidate malaria vaccine that might help protect persons from malaria, but it is not proven yet.
2. You will receive clinical care at any time during the trial period (43 + 40 days). This will include care for unscheduled sick visits.
3. Community and national benefit. This will be the first malaria clinical vaccine trial that will be conducted in Uganda. As such the opportunity will offer the firsts in creating a clinical research environment that will benefit not only the participants and the districts serviced by Lira Medical Centre, but the information gained would provide an important addition to the current methods for malaria control and eradication, not only in Uganda but worldwide.

PRECAUTIONS TO FOLLOW:
Once you are evaluated for possible participation, with the exception of true emergencies, we prefer that your medical care will be done at LMC. If a visit to another clinic or doctor will be done, please show the "Schedule Management" booklet that will be given to you. We ask your cooperation for this arrangement until the end of the trial. Note: The malaria vaccine has not been proven to prevent malaria, so please continue to practice your regular malaria prevention methods.

DURATION OF PARTICIPATION: 43 (excluding screening) + 40 days
NUMBER OF SUBJECTS IN THE TRIAL:
21 to 40 years old: 40 to 56 (20 to 28 males/females)

COST AND COMPENSATION:
All health examination, blood and urine tests at screening will be free. If after these initial tests you would not be able to participate due to health reasons, we will refer you to physicians at LMC. The initial referral and first aid medicines will also be free. However, if long term illness is discovered, long term treatment and care will not be covered.
Upon participation to this trial (when you are assigned a subject code), you do not need to pay for any clinic visits or treatments until the trial ends (43 + 40 days). Treatment will include any vaccine side-effects, such as pain or swelling, with analgesics or other treatments, and will extend until you are well. Treatment for malaria and other illnesses will also be free during the trial period. If you think you have a medical problem, we welcome you to come consult the LMC trial staff. If needed, referral will be done after consultation with physicians and you will be informed of the best possible option(s). Referrals will be documented.

You will also receive, based on Ugandan standards, an equivalent of US$10 (about 21,000.00 Ugandan shillings, based on the rate specified in Bank of Uganda; intermediate value) per visit for participating in this trial. In case of lower exchange rates the fix cost of 21,000.00 Ugandan shillings will be paid for a particular visit.

CONFIDENTIALITY:
We promise that all will be done to keep the results of this trial confidential. In any report, your name will not appear but only your subject/volunteer code. A copy of this signed consent form will be placed in our file and a copy will be given to you. All files will be kept in locked cabinets and will only be seen by trial staff (including the Sponsor Institute, Sponsor representative company) and representatives from Ugandan authorities (MBL-IRC, UNCST and NDA) for the verification of clinical trial procedures and/or data.

CIRCUMSTANCES UNDER WHICH PARTICIPATION MAY BE TERMINATED WITHOUT CONSENT:
The doctor may decide to stop participation due to:
1. Health conditions that might make your participation dangerous to your health
2. Any other conditions that might make continued participation dangerous to your health

ALTERNATIVE TO PARTICIPATION:
You may not participate. Medical care and health examination is available at LMC.

Also, during the trial, we will make our best effort to tell you at once for any new information that may influence your willingness to continue participation.

CONSENT:
Do you have questions taking part in this trial? If you have any questions or concerns about taking part in this trial at a later date, or if you feel that you have been injured by taking part in this trial you may contact Prof. Thomas Egwang, *Tel. no.*: +254-712-504 010; +256-775-494 752. Any trial staff at LMC can also help you contact the Investigators. For any issues regarding your rights and welfare, please contact Prof. Edward Kirumira (Chairman MBL-IRC) on +256 752 767 439; +256 414 545 040. LMC contact numbers are: +256 392 948833 / +256 772 419397. You may come to the clinic anytime for the duration of this trial.

WHAT YOUR SIGNATURE OR THUMBPRINT MEANS
Your signature or thumbprint below means that you understand the information given to you in this consent form and that you agree to participate in this trial. You will be asked to sign another informed consent form for the long term storage and use of blood samples.

Name of volunteer (printed)

Gender Age on Informed Consent Address

Signature of volunteer Date/Time

Thumbprint of volunteer if unable to sign:

Name and Signature of Investigator Date/Time

Language/Dialect for Informed Consent Subject Code

18.2.2 ICF for Stage 2: Adults (18 to 20 y old)

TITLE: Single blind, randomized, controlled, Phase 1b trial of the safety and immunogenicity of lyophilized recombinant precipitated tropical malaria vaccine (BK-SE36), in Uganda

INSTITUTIONS:

1. Lira Medical Centre (LMC), Lira, Uganda
2. Med Biotech Laboratories (MBL), Kampala, Uganda
3. The Research Foundation for Microbial Diseases of Osaka University (BIKEN), Japan
4. Research Institute for Microbial Diseases (RIMD), Osaka University, Japan

PRINCIPAL INVESTIGATOR:

Thomas Egwang, PhD
Director General/Scientific Director
Med Biotech Laboratories, Plot 3438 Muyenga, Tank Hill By-pass,
PO Box 9364, Kampala, Uganda *Tel* +254-712-504 010; +256-775-494 752

CLINICAL TRIAL SITE:

Lira Medical Centre
P.O. Box 1075, Plot #15, Moroto Road, Lira, Uganda
Tel +256-392-948 833; +256-772-419 397
(c/o Dr. Ogwang Ochoo Ben, Director, LMC)
http://www.lira-medical-centre.org/

MBL-IRC:

Professor Edward K. Kirumira (Chairman)
Dean, Faculty of Social Science, Makerere University, Kampala
Tel +256 752 767 439
kirumira@starcom.co.ug

Flavia Zalwango K (Secretary)
Plan International, Uganda,
Tel +256 782 094 273
flavia.zalwango@plan-international.org, zaly14@yahoo.com

BK-SE36 Phase 1b

(ADULTS: 18 TO 20 Y OLD)

PARTICIPANT INFORMATION:

We would like to invite you to participate in a medical research trial. It is important that you understand the following information that applies to all volunteers in our studies:

1. Participation is entirely voluntary
2. If you refuse to take part in this trial it will not affect your current or future medical care in LMC
3. You may withdraw from participation at anytime
4. Your refusal to participate will involve no penalty or loss of benefits to which you are otherwise entitled. If you decide to withdraw from the trial later, please inform any member of the trial staff or meet with the principal investigator
5. After this explanation, please feel free to ask anything you do not understand
6. During participation in the trial, you will be informed of any new findings that may affect your willingness to participate
7. It would be advisable for someone with HIV not to participate in the trial.

INTRODUCTION/PURPOSE:

Malaria is a disease that affects many people throughout the world, including Uganda. It is caused by parasites that are transmitted by mosquito bites. Inside the human body, the parasites multiply in the liver, and then infect red blood cells. Symptoms of malaria include fever, headache and vomiting, and if not treated can quickly become life-threatening. In many parts of the world, the parasites have developed resistance to a number of malaria medicines.

Investigators at the Research Institute for Microbial Diseases in collaboration with The Research Foundation for Microbial Diseases of Osaka University have developed an experimental vaccine against malaria called BK-SE36. BK-SE36 contains a protein derived from malaria parasites that can prevent people from getting sick with malaria but it is not approved yet for general use. Normally, before a vaccine can be sold to market, it has to be tested in several clinical trials to see the safety and the ability of the vaccine to work (or its effect on the body). These trials are done with extreme care/concerns, strictly following guidelines to be able to evaluate thoroughly the new vaccine. Preliminary tests of this vaccine in 30 adults in Japan have shown that this vaccine is safe. No serious adverse events were observed and all adverse events were resolved. The predominant adverse event has been local swelling, although, erythema and induration at the administration site has been noted. The events were all mild and remitted. Longest onset of adverse event (induration) was at 43 days after the third administration. There was one subject that had the longest adverse event (induration), which was observed 4 days post administration and resolved 60 days after, without therapy or limitation of daily activity. In this trial, we would like to test BK-SE36 vaccine in young adults and children in Uganda to make sure that it is safe to people who have already been exposed to malaria, and to find out

the best dose to use for children. To do this, we will compare its safety to physiological saline, a proven safe medicine used for intravenous infusion.

This trial has been approved by the MBL-Institutional Review Committee (MBL-IRC). MBL-IRC is an independent committee in Uganda that will ensure the protection of people's right, safety and well-being in this trial. We hope that information from this trial can be used to develop a vaccine that in the future will help protect people from getting sick with malaria.

PROCEDURES TO BE FOLLOWED:
If you will participate in this research trial, we will set a date for you to be back for an appointment with one of the doctors. During that appointment (screening day):
1. we will ask you for medical history; and
2. ask you to undergo a medical examination. We will do a physical examination and do tests on blood and urine to look for any signs of illness in the blood, kidneys or liver. We will draw about 1.6 teaspoon of blood (8 ml) to evaluate your health condition. You will be told of all the test results and the possible meaning of these results. If we find anything wrong that will not allow you to participate in the trial, we will refer you to the other doctors at LMC.

If your results are good, and if you agree to continue, you will be scheduled to receive 2 doses of either the experimental vaccine BK-SE36 or physiological saline at a later date. Prior to receiving the first dose of vaccine, a picture will also be taken for file. The photograph will only be used for identification purposes. You will also be given a "Schedule Management" booklet that you will need to bring every time you visit LMC. We may also invite you a week before the scheduled vaccination to check your health for any signs of malaria. On the day of vaccination, we will again examine you including checks for blood and urine.

You will not know which vaccine and what dose (1.0 ml or 0.5 ml) you will get until the end of the trial. Which vaccine and dose you get will be determined by chance, like when you toss a coin and you do not know whether it will land on the side with a face or on the other side. This is done to make sure that assessments are done unbiased.

Once the first dose of vaccine is given, the second dose will be given about 21 days later. Vaccination will be by subcutaneous injection on the upper arm. After each vaccination, you will be asked to stay at LMC for 60 minutes for further observation. We will also ask you to come back again after 1, 7, 14 and 21 days after each vaccination (a total of 9 clinic visits excluding screening days). These visits will allow us to see you, to know how you are feeling and for brief medical examination. In addition, at any time during 40 days after visit 10, you are invited to go to LMC whenever you are sick. The doctor will determine if it is due to vaccination and will record all symptoms.

During the trial, we will need to draw blood for a total of four times. This will be done to make sure you remain healthy and to measure the effect of the vaccine. Some of the blood samples obtained will be stored to do other tests that will help us see the effect of the vaccine. We need a separate consent for this. The total amount of blood will be about 3.1 tablespoons (or 9.4 teaspoons= 47 ml). If you become ill, we may

need to draw additional blood to run further tests. If you become ill from malaria, we also have to prick to confirm diagnosis before we do treatment.

POTENTIAL RISKS AND DISCOMFORTS

1. Blood sampling. Drawing blood might cause discomfort and occasional bruising at the site. The amount of blood to be removed will be too small to affect your health.

2. Vaccination side-effects. For vaccination you should expect soreness, swelling, and in some cases, redness at the site of injection. In addition, muscle soreness, itching, irritability or fussiness, low-grade fever, fatigue, headache, nausea and dizziness are possible. Some days of local pain and limitation in arm movement may also be observed. These are usually transient and will disappear in time. If any of these symptoms occur, we may give you medication to help provide relief. As with any vaccine, there is a small possibility that you may have an allergic reaction. These may be mild, such as rash, or may be severe and life threatening. A life threatening reaction is extremely rare; but medicine and equipment will be available at LMC.

   There may be other reaction(s) to the malaria vaccine that at this time is unknown. If new information about the safety of the vaccine becomes available, we will do our best to inform you at once.

   For any vaccine side effects, treatment will be provided until, at least, all symptoms are resolved.

3. The risks of donating urine are minimal.

4. For female volunteers. Adolescents and adults are cautioned of the unknown risk of BK-SE36 to the unborn child and will be advised to use adequate birth control methods during participation in the trial. If you are interested in birth control methods we will refer you for consultation at LMC.

   A pregnancy test will be done on your urine during screening and before each vaccination. If the pregnancy test becomes positive before the trial starts (before your first vaccination), you will not be included in this trial. If you become positive after the first vaccination, we will no longer give you any vaccine. However, we will continue to check to see if you are well. With your permission we will follow your pregnancy to term. Your health and your child's health status including date of delivery and child's gender and weight will be noted.

BENEFITS:

1. For now, you may not have any direct benefit. BK-SE36 is a candidate malaria vaccine that might help protect persons from malaria, but it is not proven yet.
2. You will receive clinical care at any time during the trial period (43 + 40 days). This will include care for unscheduled sick visits.
3. Community and national benefit. This will be the first malaria clinical vaccine trial that will be conducted in Uganda. As such the opportunity will offer the firsts in creating a clinical research environment that will benefit not only the participants and the districts serviced by Lira Medical Centre, but the information

gained would provide an important addition to the current methods for malaria control and eradication, not only in Uganda but worldwide.

PRECAUTIONS TO FOLLOW:
Once you are evaluated for possible participation, with the exception of true emergencies, we prefer that your medical care will be done at LMC. If a visit to another clinic or doctor will be done, please show the "Schedule Management" booklet that will be given to you. We ask your cooperation for this arrangement until the end of the trial. Note: The malaria vaccine has not been proven to prevent malaria, so please continue to practice your regular malaria prevention methods.

DURATION OF PARTICIPATION: 43 (excluding screening) + 40 days
NUMBER OF SUBJECTS IN THE TRIAL:
6 to 20 years old: 84 (28 volunteers for each of the following age groups: 6 to 10 y old, 11 to 15 y old, and 16-20 y old)

COST AND COMPENSATION:
All health examination, blood and urine tests at screening will be free. If after these initial tests you would not be able to participate due to health reasons, we will refer you to physicians at LMC. The initial referral and first aid medicines will also be free. However, if long term illness is discovered, long term treatment and care will not be covered.
Upon participation to this trial (when you are assigned a subject code), you do not need to pay for any clinic visits or treatments until the trial ends (43 + 40 days). Treatment will include any vaccine side-effects, such as pain or swelling, with analgesics or other treatments, and will extend until you are well. Treatment for malaria and other illnesses will also be free during the trial period. If you think you have a medical problem, we welcome you to come consult the LMC trial staff. If needed, referral will be done after consultation with physicians and you will be informed of the best possible option(s). Referrals will be documented.

You will also receive, based on Ugandan standards, an equivalent of US$10 (about 21,000.00 Ugandan shillings, based on the rate specified in Bank of Uganda; intermediate value) per visit for participating in this trial. In case of lower exchange rates the fix cost of 21,000.00 Ugandan shillings will be paid for a particular visit.

CONFIDENTIALITY:
We promise that all will be done to keep the results of this trial confidential. In any report, your name will not appear but only your subject/volunteer code. A copy of this signed consent form will be placed in our file and a copy will be given to you. All files will be kept in locked cabinets and will only be seen by trial staff (including the Sponsor Institute, Sponsor representative company) and representatives from Ugandan authorities (MBL-IRC, UNCST and NDA) for the verification of clinical trial procedures and/or data.

CIRCUMSTANCES UNDER WHICH PARTICIPATION MAY BE TERMINATED WITHOUT CONSENT:

The doctor may decide to stop participation due to:
1. Health conditions that might make your participation dangerous to your health

2. Any other conditions that might make continued participation dangerous to your health

ALTERNATIVE TO PARTICIPATION:
You may not participate. Medical care and health examination is available at LMC. Also during the trial, we will make our best effort to tell you at once for any new information that may influence your willingness to continue participation.

CONSENT:
Do you have questions taking part in this trial? If you have any questions or concerns about taking part in this trial at a later date, or if you feel that you have been injured by taking part in this trial you may contact Prof. Thomas Egwang, *Tel. no.*: +254-712-504 010; +256-775-494 752. Any trial staff at LMC can also help you contact the Investigators. For any issues regarding your rights and welfare, please contact Prof. Edward Kirumira (Chairman MBL-IRC) on +256 752 767 439; +256 414 545 040. LMC contact numbers are: +256 392 948833 / +256 772 419397. You may come to the clinic anytime for the duration of this trial.

BK-SE36 Phase 1b

WHAT YOUR SIGNATURE OR THUMBPRINT MEANS

Your signature or thumbprint below means that you understand the information given to you in this consent form and that you agree to participate in this trial. You will be asked to sign another informed consent form for the long term storage and use of blood samples.

Name of Volunteer (printed)

Gender Age on Informed Consent Address

Signature of Volunteer Date/Time

Thumbprint of volunteer if unable to sign:

Name and Signature of Investigator Date/Time

Language/Dialect for Informed Consent Subject Code

18.2.3 ICF for Stage 2: Parent(s)/Guardian(s) of Volunteers (6 to 7 y old)

TITLE: **Single blind, randomized, controlled, Phase 1b trial of the safety and immunogenicity of lyophilized recombinant precipitated tropical malaria vaccine (BK-SE36), in Uganda**

INSTITUTIONS:

1. Lira Medical Centre (LMC), Lira, Uganda
2. Med Biotech Laboratories (MBL), Kampala, Uganda
3. The Research Foundation for Microbial Diseases of Osaka University (BIKEN), Japan
4. Research Institute for Microbial Diseases (RIMD), Osaka University, Japan

PRINCIPAL INVESTIGATOR:

Thomas Egwang, PhD
Director General/Scientific Director
Med Biotech Laboratories, Plot 3438 Muyenga, Tank Hill By-pass,
PO Box 9364, Kampala, Uganda *Tel* +254-712-504 010; +256-775-494 752

CLINICAL TRIAL SITE:

Lira Medical Centre
P.O. Box 1075, Plot #15, Moroto Road, Lira, Uganda
Tel +256-392-948 833; +256-772-419 397
(c/o Dr. Ogwang Ochoo Ben, Director, LMC)
http://www.lira-medical-centre.org/

MBL-IRC:

Professor Edward K. Kirumira (Chairman)
Dean, Faculty of Social Science, Makerere University, Kampala
Tel +256 752 767 439
kirumira@starcom.co.ug

Flavia Zalwango K (Secretary)
Plan International, Uganda,
Tel +256 782 094 273
flavia.zalwango@plan-international.org, zaly14@yahoo.com

[PARENT(S)/GUARDIAN(S): 6 TO 7 Y OLD]

PARTICIPANT INFORMATION:

We would like to invite your child to participate in a medical research trial. As a parent/guardian we ask your understanding for the following information that applies to all volunteers in our studies:

1. Participation is entirely voluntary
2. If you do not like your child to take part in this trial it will not affect your child's current or future medical care in LMC
3. You may withdraw your child's participation at anytime
4. Your refusal for your child's participation will involve no penalty or loss of benefits to which your child is entitled. If you decide to withdraw your child from the trial later, please inform any member of the trial staff or meet with the principal investigator
5. After this explanation, please feel free to ask anything you do not understand
6. During participation in the trial, you will be informed of any new findings that may affect your willingness to let your child participate
7. If your child has HIV, it would be advisable for him/her not to participate in the trial.

INTRODUCTION/PURPOSE:

Malaria is a disease that affects many people throughout the world, including Uganda. It is caused by parasites that are transmitted by mosquito bites. Inside the human body, the parasites multiply in the liver, and then infect red blood cells. Symptoms of malaria include fever, headache and vomiting, and if not treated can quickly become life-threatening. In many parts of the world, the parasites have developed resistance to a number of malaria medicines.

Investigators at the Research Institute for Microbial Diseases in collaboration with The Research Foundation for Microbial Diseases of Osaka University have developed an experimental vaccine against malaria called BK-SE36. BK-SE36 contains a protein derived from malaria parasites that can prevent people from getting sick with malaria but it is not approved yet for general use. Normally, before a vaccine can be sold to market, it has to be tested in several clinical trials to see the safety and the ability of the vaccine to work (or its effect on the body). These trials are done with extreme care/concerns, strictly following guidelines to be able to evaluate thoroughly the new vaccine. Preliminary tests of this vaccine in 30 adults in Japan have shown that this vaccine is safe. No serious adverse events were observed and all adverse events were resolved. The predominant adverse event has been local swelling, although, erythema and induration at the administration site has been noted. The events were all mild and remitted. Longest onset of adverse event (induration) was at 43 days after the third administration. There was one subject that had the longest adverse event (induration), which was observed 4 days post administration and resolved 60 days after, without therapy or limitation of daily activity. In this trial, we would like to test BK-SE36 vaccine in young adults and children in Uganda to make sure that it is safe to people who have already been exposed to malaria, and to find out the best dose to use for children. To do this, we will compare its safety to physiological saline, a proven safe medicine used for intravenous infusion.

This trial has been approved by the MBL-Institutional Review Committee (MBL-IRC). MBL-IRC is an independent committee in Uganda that will ensure the protection of people's right, safety and well-being in this trial. We hope that information from this trial can be used to develop a vaccine that in the future will help protect people from getting sick with malaria.

PROCEDURES TO BE FOLLOWED:
If you will allow your child to participate in this research trial, we will set a date for your child to be back for an appointment with one of the doctors. During that appointment (screening day):
1. we will ask for your child's medical history; and
2. ask your child to undergo a medical examination. We will do a physical examination and do tests on blood and urine to look for any signs of illness in the blood, kidneys or liver. We will draw about 1.6 teaspoon of blood (8 ml) to evaluate your child's health condition. You will be told of all the test results and the possible meaning of these results. If we find anything wrong that will not allow your child to participate in the trial, we will refer your child to the other doctors at LMC.

If your child's results are good, and if you agree for him (or her) to continue, he (or she) will be scheduled to receive 2 doses of either the experimental vaccine BK-SE36 or physiological saline at a later date. Prior to receiving the first dose of vaccine, a picture of your child will also be taken for file. The photograph will only be used for identification purposes. You will also be given a "Schedule Management" booklet that you will need to bring every time you and your child visit LMC. We may also invite you and your child a week before the scheduled vaccination to check his (or her) health for any signs of malaria. On the day of vaccination, we will again examine him (or her) including checks for blood and urine.

You will not know which vaccine and what dose (1.0 ml or 0.5 ml) your child will get until the end of the trial. Which vaccine and dose your child will get will be determined by chance, like when you toss a coin and you do not know whether it will land on the side with a face or on the other side. This is done to make sure that assessments are done unbiased.

Once the first dose of vaccine is given, the second dose will be given about 21 days later. Vaccination will be by subcutaneous injection on the upper arm. After each vaccination, you and your child will be asked to stay at LMC for 60 minutes for further observation. We will also ask you and your child to come back again after 1, 7, 14 and 21 days after each vaccination (a total of 9 clinic visits excluding screening days). These visits will allow us to see your child, to know how he (or she) is feeling and for brief medical examination. In addition, at any time during 40 days after visit 10, you can bring your child to LMC whenever he (or she) is sick. The doctor will determine if it is due to vaccination and will record all symptoms.

During the trial, we will need to draw blood for a total of four times. This will be done to make sure your child remain healthy and to measure the effect of the vaccine. Some of the blood samples obtained will be stored to do other tests that will help us see the effect of the vaccine. We need a separate consent for this. The total amount of blood will be about 3.1 tablespoons (or 9.4 teaspoons= 47 ml). If your child becomes ill, we may need to draw additional blood to run further tests. If your child

becomes ill from malaria, we also have to prick to confirm diagnosis before we do treatment.

POTENTIAL RISKS AND DISCOMFORTS

1. Blood sampling. Drawing blood might cause discomfort and occasional bruising at the site. The amount of blood to be removed will be too small to affect your child's health.

2. Vaccination side-effects. For vaccination you should expect to see soreness, swelling, and in some cases, redness at the site of injection. In addition, muscle soreness, itching, irritability or fussiness, low-grade fever, fatigue, headache, nausea and dizziness are possible. Some days of local pain and limitation in arm movement may also be observed. These are usually transient and will disappear in time. If any of these symptoms occur, we may give your child medication to help provide relief. As with any vaccine, there is a small possibility that your child may have an allergic reaction. These may be mild, such as rash, or may be severe and life threatening. A life threatening reaction is extremely rare; but medicine and equipment will be available at LMC.

   There may be other reaction(s) to the malaria vaccine that at this time is unknown. If new information about the safety of the vaccine becomes available, we will do our best to inform you at once.

   For any vaccine side effects, treatment will be provided until, at least, all symptoms are resolved.

3. The risks of donating urine are minimal.

BENEFITS:

1. For now, your child may not have any direct benefit. BK-SE36 is a candidate malaria vaccine that might help protect persons from malaria, but it is not proven yet.
2. Your child will receive clinical care at any time during the trial period (43 + 40 days). This will include care for unscheduled sick visits.
3. Community and national benefit. This will be the first malaria clinical vaccine trial that will be conducted in Uganda. As such the opportunity will offer the firsts in creating a clinical research environment that will benefit not only the participants and the districts serviced by Lira Medical Centre, but the information gained would provide an important addition to the current methods for malaria control and eradication, not only in Uganda but worldwide.

PRECAUTIONS TO FOLLOW:

Once your child is evaluated for possible participation, with the exception of true emergencies, we prefer that your child's medical care will be done at LMC. If a visit to another clinic or doctor will be done, please show the "Schedule Management" booklet that will be given to you. We ask your cooperation for this arrangement until the end of the trial. Note: The malaria vaccine has not been proven to prevent malaria, so please continue to practice your regular malaria prevention methods.

DURATION OF PARTICIPATION: 43 (excluding screening) + 40 days

NUMBER OF SUBJECTS IN THE TRIAL:

6 to 20 years old: 84 (28 volunteers for each of the following age groups: 6 to 10 y old, 11 to 15 y old, and 16-20 y old)

COST AND COMPENSATION:

All health examination, blood and urine tests at screening will be free. If after these initial tests your child would not be able to participate due to health reasons, we will refer your child to physicians at LMC. The initial referral and first aid medicines will also be free. However, if long term illness is discovered, long term treatment and care will not be covered.
Upon participation to this trial (when your child is assigned a subject code), you do not need to pay for any clinic visits or treatments until the trial ends (43 + 40 days). Treatment will include any vaccine side-effects, such as pain or swelling, with analgesics or other treatments, and will extend until your child is well. Treatment for malaria and other illnesses will also be free during the trial period. If your child has a medical problem, we welcome him or her to come consult the LMC trial staff. If needed, referral will be done after consultation with physicians and you will be informed of the best possible option(s). Referrals will be documented.

Your child will also receive, based on Ugandan standards, an equivalent of US$10 (about 21,000.00 Ugandan shillings, based on the rate specified in Bank of Uganda; intermediate value) per visit for participating in this trial. In case of lower exchange rates the fix cost of 21,000.00 Ugandan shillings will be paid for a particular visit.

CONFIDENTIALITY:

We promise that all will be done to keep the results of this trial confidential. In any report, your child's name will not appear but only his/her subject/volunteer code. A copy of this signed consent form will be placed in our file and a copy will be given to you. All files will be kept in locked cabinets and will only be seen by trial staff (including the Sponsor Institute, Sponsor representative company) and representatives from Ugandan authorities (MBL-IRC, UNCST and NDA) for the verification of clinical trial procedures and/or data.

CIRCUMSTANCES UNDER WHICH PARTICIPATION MAY BE TERMINATED WITHOUT CONSENT:

The doctor may decide to stop participation due to:

1. Health conditions that might make your child's participation dangerous to his (or her) health
2. Any other conditions that might make continued participation dangerous to your child's health

ALTERNATIVE TO PARTICIPATION:

Your child may not participate. Medical care and health examination is available at LMC. Also during the trial, we will make our best effort to tell you at once for any new information that may influence your willingness to let your child continue participation.

CONSENT:
Do you have questions taking part in this trial? If you have any questions or concerns about taking part in this trial at a later date, or if you feel that you have been injured by taking part in this trial you may contact Prof. Thomas Egwang, *Tel. no.*: +254-712-504 010; +256-775-494 752. Any trial staff at LMC can also help you contact the Investigators. For any issues regarding your rights and welfare, please contact Prof. Edward Kirumira (Chairman MBL-IRC) on +256 752 767 439; +256 414 545 040. LMC contact numbers are: +256 392 948833 / +256 772 419397. You may come to the clinic anytime for the duration of this trial.

WHAT YOUR SIGNATURE OR THUMBPRINT MEANS

Your signature or thumbprint below means that you understand the information given to you in this consent form and that you agree for your child to participate in this trial. You will be asked to sign another informed consent form for the use and long term storage of blood samples.

Name of Volunteer (printed)

Gender Age on Informed Consent Address

Name of Parent/Guardian Relationship to Volunteer

Signature of Parent/Guardian Date/Time

Thumbprint of Parent/Guardian if unable to sign:

Name and Signature of Investigator Date/Time

Language/Dialect for Informed Consent Subject Code

18.2.4 ICF for Stage 2: Children AND Parent(s)/Guardian(s) (8 to 17 y old)

TITLE: **Single blind, randomized, controlled, Phase 1b trial of the safety and immunogenicity of lyophilized recombinant precipitated tropical malaria vaccine (BK-SE36), in Uganda**

INSTITUTIONS:

1. Lira Medical Centre (LMC), Lira, Uganda
2. Med Biotech Laboratories (MBL), Kampala, Uganda
3. The Research Foundation for Microbial Diseases of Osaka University (BIKEN), Japan
4. Research Institute for Microbial Diseases (RIMD), Osaka University, Japan

PRINCIPAL INVESTIGATOR:

Thomas Egwang, PhD
Director General/Scientific Director
Med Biotech Laboratories, Plot 3438 Muyenga, Tank Hill By-pass,
PO Box 9364, Kampala, Uganda *Tel* +254-712-504 010; +256-775-494 752

CLINICAL TRIAL SITE:

Lira Medical Centre
P.O. Box 1075, Plot #15, Moroto Road, Lira, Uganda
Tel +256-392-948 833; +256-772-419 397
(c/o Dr. Ogwang Ochoo Ben, Director, LMC)
http://www.lira-medical-centre.org/

MBL-IRC:

Professor Edward K. Kirumira (Chairman)
Dean, Faculty of Social Science, Makerere University, Kampala
Tel +256 752 767 439
kirumira@starcom.co.ug

Flavia Zalwango K (Secretary)
Plan International, Uganda,
Tel +256 782 094 273
flavia.zalwango@plan-international.org, zaly14@yahoo.com

(CHILDREN AND PARENT(S)/GUARDIAN(S): 8 TO 17 Y OLD)

PARTICIPANT INFORMATION:

We would like to invite your child to participate in a medical research trial. It is important that you and your child understand the following information that applies to all volunteers in our studies:

1. Participation is entirely voluntary
2. We need BOTH you and your child to agree for participation to the trial. Your child's agreement is important so that even if you do agree but your child will not agree we cannot allow participation to this trial
3. If you or your child do not like to take part in this trial it will not affect your child's current or future medical care in LMC
4. You or your child may withdraw from participation at anytime
5. Your or your child's refusal to participate will involve no penalty or loss of benefits to which your child is entitled. If you or your child will decide to withdraw from the trial later, please inform any member of the trial staff or meet with the principal investigator
6. After this explanation, we invite you and your child to ask anything that will help both of you to understand more about this trial
7. During participation in the trial, you and your child will be informed of any new findings that may affect both of your willingness to participate
8. If your child has HIV, it is advisable for him (or her) not to participate in this trial.

INTRODUCTION/PURPOSE:

Malaria is a disease that affects many people throughout the world, including Uganda. It is caused by parasites that are transmitted by mosquito bites. Inside the human body, the parasites multiply in the liver, and then infect red blood cells. Symptoms of malaria include fever, headache and vomiting, and if not treated can quickly become life-threatening. In many parts of the world, the parasites have developed resistance to a number of malaria medicines.

Investigators at the Research Institute for Microbial Diseases in collaboration with The Research Foundation for Microbial Diseases of Osaka University have developed an experimental vaccine against malaria called BK-SE36. BK-SE36 contains a protein derived from malaria parasites that can prevent people from getting sick with malaria but it is not approved yet for general use. Normally, before a vaccine can be sold to market, it has to be tested in several clinical trials to see the safety and the ability of the vaccine to work (or its effect on the body). These trials are done with extreme care/concerns, strictly following guidelines to be able to evaluate thoroughly the new vaccine. Preliminary tests of this vaccine in 30 adults in Japan have shown that this vaccine is safe. No serious adverse events were observed and all adverse events were resolved. The predominant adverse event has been local swelling, although, erythema and induration at the administration site has been noted. The events were all mild and remitted. Longest onset of adverse event (induration) was at 43 days after the third administration. There was one subject that had the longest adverse event (induration), which was observed 4 days post administration and resolved 60 days after, without

therapy or limitation of daily activity In this trial, we would like to test BK-SE36 vaccine in young adults and children in Uganda to make sure that it is safe to people who have already been exposed to malaria, and to find out the best dose to use for children. To do this, we will compare its safety to physiological saline, a proven safe medicine used for intravenous infusion.

This trial has been approved by the MBL-Institutional Review Committee (MBL-IRC). MBL-IRC is an independent committee in Uganda that will ensure the protection of people's right, safety and well-being in this trial. We hope that information from this trial can be used to develop a vaccine that in the future will help protect people from getting sick with malaria.

PROCEDURES TO BE FOLLOWED:

If you and your child would like to participate, we will set a date for your child to be back for an appointment with one of the doctors. During that appointment (screening day):
1. we will ask you or your child for his (or her) medical history; and
2. ask your child to undergo a medical examination. We will do a physical examination and do tests on blood and urine to look for any signs of illness in the blood, kidneys or liver. We will draw about 1.6 teaspoon of blood (8 ml) to evaluate your child's health condition. You will be told of all the test results and the possible meaning of these results. If we find anything wrong that will not allow your child to participate in the trial, we will refer your child to the other doctors at LMC.

If your child's results are good, and if you and he (or she) agrees to continue, he (or she) will be scheduled to receive 2 doses of either the experimental vaccine BK-SE36 or physiological saline at a later date. Prior to receiving the first dose of vaccine, a picture of your child will also be taken for file. The photograph will only be used for identification purposes. Your child will also be given a "Schedule Management" booklet that he or she needs to bring at every LMC visit. We may also invite your child a week before the scheduled vaccination to check his (or her) health for any signs of malaria. On the day of vaccination, we will again examine him (or her) including checks for blood and urine.

You and your child will not know which vaccine and what dose (1.0 ml or 0.5 ml) he or she will get until the end of the trial. Which vaccine and dose your child will get will be determined by chance, like when you toss a coin and you do not know whether it will land on the side with a face or on the other side. This is done to make sure that assessments are done unbiased.

Once the first dose of vaccine is given, the second dose will be given about 21 days later. Vaccination will be by subcutaneous injection on the upper arm. After each vaccination, your child will be asked to stay at LMC for 60 minutes for further observation. We will also ask your child to come back again after 1, 7, 14 and 21 days after each vaccination (a total of 9 clinic visits excluding screening days). These visits will allow us to see your child, to know how he (or she) is feeling and for brief medical examination. In addition, at any time during 40 days after visit 10, your child can come to LMC whenever he (or she) is sick. The doctor will determine if it is due to vaccination and will record all symptoms.

During the trial, we will need to draw blood for a total of four times. This will be done to make sure your child remain healthy and to measure the effect of the vaccine. Some of the blood samples obtained will be stored to do other tests that will help us see the effect of the vaccine. We need a separate consent for this. The total amount of blood will be about 3.1 tablespoons (or 9.4 teaspoons= 47 ml). If your child becomes ill, we may need to draw additional blood to run further tests. If your child becomes ill from malaria, we also have to prick to confirm diagnosis before we do treatment.

POTENTIAL RISKS AND DISCOMFORTS

1. Blood sampling. Drawing blood might cause discomfort and occasional bruising at the site. The amount of blood to be removed will be too small to affect your child's health.

2. Vaccination side-effects. For vaccination you and your child should expect to see soreness, swelling, and in some cases, redness at the site of injection. In addition, muscle soreness, itching, irritability or fussiness, low-grade fever, fatigue, headache, nausea and dizziness are possible. Some days of local pain and limitation in arm movement may also be observed. These are usually transient and will disappear in time. If any of these symptoms occur, we may give your child medication to help provide relief. As with any vaccine, there is a small possibility that your child may have an allergic reaction. These may be mild, such as rash, or may be severe and life threatening. A life threatening reaction is extremely rare; but medicine and equipment will be available at LMC.

   There may be other reaction(s) to the malaria vaccine that at this time is unknown. If new information about the safety of the vaccine becomes available, we will do our best to inform you and your child at once.

   For any vaccine side effects, treatment will be provided until, at least, all symptoms are resolved.

3. The risks of donating urine are minimal.

4. For female volunteers. Adolescents are cautioned of the unknown risk of BK-SE36 to the unborn child and will be advised to use adequate birth control methods during participation in the trial. If you are interested in birth control methods we will refer you for consultation at LMC.

   A pregnancy test will be done on your urine during screening and before each vaccination. If the pregnancy test becomes positive before the trial starts (before your first vaccination), you will not be included in this trial. If you become positive after the first vaccination, we will no longer give you any vaccine. However, we will continue to check to see if you are well. With your permission we will follow your pregnancy to term. Your health and your child's health status including date of delivery and child's gender and weight will be noted.

BENEFITS:

1. For now, your child may not have any direct benefit. BK-SE36 is a candidate malaria vaccine that might help protect persons from malaria, but it is not proven yet.
2. Your child will receive clinical care at any time during the trial period (43 + 40 days). This will include care for unscheduled sick visits.
3. Community and national benefit. This will be the first malaria clinical vaccine trial that will be conducted in Uganda. As such the opportunity will offer the firsts in creating a clinical research environment that will benefit not only the participants and the districts serviced by Lira Medical Centre, but the information gained would provide an important addition to the current methods for malaria control and eradication, not only in Uganda but worldwide.

PRECAUTIONS TO FOLLOW:

Once your child is evaluated for possible participation, with the exception of true emergencies, we prefer that your child's medical care will be done at LMC. If a visit to another clinic or doctor will be done, please show the "Schedule Management" booklet that will be given to your child. We ask your cooperation for this arrangement until the end of the trial. <u>Note</u>: The malaria vaccine has not been proven to prevent malaria, so please continue to practice your regular malaria prevention methods.

DURATION OF PARTICIPATION: 43 (excluding screening) + 40 days

NUMBER OF SUBJECTS IN THE TRIAL:

6 to 20 years old: 84 (28 volunteers for each of the following age groups: 6 to 10 y old, 11 to 15 y old, and 16-20 y old)

COST AND COMPENSATION:

All health examination, blood and urine tests at screening will be free. If after these initial tests your child would not be able to participate due to health reasons, we will refer your child to physicians at LMC. The initial referral and first aid medicines will also be free. However, if long term illness is discovered, long term treatment and care will not be covered.
Upon participation to this trial (when your child is assigned a subject code), you do not need to pay for any clinic visits or treatments until the trial ends (43 + 40 days). Treatment will include any vaccine side-effects, such as pain or swelling, with analgesics or other treatments, and will extend until your child is well. Treatment for malaria and other illnesses will also be free during the trial period. If your child has a medical problem, we welcome him or her to come consult the LMC trial staff. If needed, referral will be done after consultation with physicians and you will be informed of the best possible option(s). Referrals will be documented.

Your child will also receive, based on Ugandan standards, an equivalent of US$10 (about 21,000.00 Ugandan shillings, based on the rate specified in Bank of Uganda;

intermediate value) per visit for participating in this trial. In case of lower exchange rates the fix cost of 21,000.00 Ugandan shillings will be paid for a particular visit.

CONFIDENTIALITY:
We promise that all will be done to keep the results of this trial confidential. In any report, your child's name will not appear but only his or her subject/volunteer code. A copy of this signed consent form will be placed in our file and a copy will be given to you. All files will be kept in locked cabinets and will only be seen by trial staff (including the Sponsor Institute, Sponsor representative company) and representatives from Ugandan authorities (MBL-IRC, UNCST and NDA) for the verification of clinical trial procedures and/or data.

CIRCUMSTANCES UNDER WHICH PARTICIPATION MAY BE TERMINATED WITHOUT CONSENT:
The doctor may decide to stop participation due to:

1. Health conditions that might make your child's participation dangerous to his (or her) health
2. Any other conditions that might make continued participation dangerous to your child's health

ALTERNATIVE TO PARTICIPATION:
Your child may not participate. Medical care and health examination is available at LMC. Also during the trial, we will make our best effort to tell you at once for any new information that may influence both of your willingness to continue participation.

CONSENT:
Do you have questions taking part in this trial? If you have any questions or concerns about taking part in this trial at a later date, or if you feel that your child have been injured by taking part in this trial you may contact Prof. Thomas Egwang, *Tel. no.*: +254-712-504 010; +256-775-494 752. Any trial staff at LMC can also help you contact the Investigators. For any issues regarding your rights and welfare, please contact Prof. Edward Kirumira (Chairman MBL-IRC) on +256 752 767 439; +256 414 545 040. LMC contact numbers are: +256 392 948833 / +256 772 419397. You and your child may come to the clinic anytime for the duration of this trial.

WHAT YOUR SIGNATURE OR THUMBPRINT MEANS

Your signature or thumbprint below means that you and your child understand the information given in this consent form and that you and your child agree to participate in this trial. You and your child will be asked to sign another informed consent form for the use and storage of blood samples.

Name of Volunteer (printed)

Gender Age on Informed Consent Address

Signature of Volunteer Date/Time

Name of Parent/Guardian Relationship to Volunteer

Signature of Parent/Guardian Date/Time

Thumbprint of Volunteer if unable to sign:	Thumbprint of Parent/Guardian if unable to sign:

Name and Signature of Investigator Date/Time

Language/Dialect for Informed Consent Subject Code

18.3 Additional documents for all informed consent
18.3.1 Trial schedule and activities (for volunteers):

****Always bring "Schedule Management" booklet***

**Days –30 to 0**	**Screening & Enrollment of Subjects**	(4 to 5 hours)

Visit 1 ***(can be spread out to several days, as necessary)***

- Written Informed Consent.
- Provision of medical history.
- Health assessment (consultation, complete physical examination, laboratory tests including urinalysis, malaria smear).

<u>if ok:</u>

- Assignment of Subject Code.
- ID Photograph.
- Malaria check.

**Day 1**	**Vaccination 1**	(5 hours)

Visit 2

Before vaccination:

- Consultation: Review of inclusion/exclusion criteria and checking for contraindications/precautions.
- Complete physical examination.
- Vital signs (temperature, blood pressure, pulse rate).
- Body weight.
- Blood sampling for laboratory tests, malaria smear and anti-SE36 protein antibody titer.
- Urinalysis.

- Vaccination of investigational product, dose 1

After vaccination: (60 minutes observation)

- Vital signs (blood pressure, pulse rate, temperature).
- Consultation; Examination of injection site.

**Day 2**	**24h Post-vaccination 1**	(30 minutes)

Visit 3

- Consultation; Examination of injection site.
- Symptom-directed physical examination.
- Vital signs (temperature, blood pressure, pulse rate).
- Urinalysis.

**Day 8**	***7-day* Post-vaccination 1**	(30 minutes)

Visit 4

- Consultation; Examination of injection site.
- Symptom-directed physical examination.
- Vital signs (temperature, blood pressure, pulse rate).

**Day 15**	***14*-day Post-vaccination 1**	(1 hour)

Visit 5

- Consultation; Examination of injection site.
- Symptom-directed physical examination.
- Vital signs (temperature, blood pressure, pulse rate).
- Urinalysis.

**Day 22**	**Vaccination 2**	(4 to 5 hours)

Visit 6

- Consultation: Review of inclusion/exclusion criteria and checking for contraindications/precautions
- Complete physical examination.
- Vital signs (temperature, blood pressure, pulse rate).
- Body weight.
- Blood sampling for laboratory tests, malaria smear and anti-SE36 protein antibody titer.
- Urinalysis.

- Vaccination of investigational product, dose 2.

After vaccination: (60 minutes observation)

- Vital signs (blood pressure, pulse rate, temperature).
- Consultation; Examination of injection site.

**Day 23**	**24h Post-vaccination 2**	(30 minutes)

Visit 7

- Consultation; Examination of injection site.
- Symptom-directed physical examination.
- Vital signs (temperature, blood pressure, pulse rate).
- Urinalysis.

**Day 29**	***7- day* Post-vaccination 2**	(30 minutes)

Visit 8

- Consultation; Examination of injection site.
- Symptom-directed physical examination.
- Vital signs (temperature, blood pressure, pulse rate).

**Day 36**	***14-day* Post-vaccination 2**	(1 hour)

Visit 9

- Consultation; Examination of injection site.
- Symptom-directed physical examination.
- Vital signs (temperature, blood pressure, pulse rate).
- Urinalysis.

**Day 43**	***21-day* Post-vaccination 2**	(1 to 2 hours)

Visit 10

- Consultation. Examination of injection site.
- Complete physical examination.
- Vital signs (temperature, blood pressure, pulse rate).
- Body weight.
- Blood sampling for laboratory tests, malaria smear, anti-SE36 protein antibody titer.
- Urinalysis

*****Important: If the volunteer is sick at anytime during the trial go to LMC for checkup and medical treatment. The trial staff will be available 24 hours a day, 7 days a week.***

**Day 44-84**	**40 day post-trial follow-up after visit 10**

Volunteers are invited to come to the Centre at any time, within 40 days, whenever they are sick. The investigators will assess the relationship of the adverse event to administration and all symptoms will be noted.

18.3.2 Form for impartial witness for volunteers unable to read or write

FORM FOR IMPARTIAL WITNESS FOR VOLUNTEERS UNABLE TO READ OR WRITE:

*If a volunteer, or parent/guardian, is unable to read and/or write, an impartial witness should be present during the informed consent discussion. After the written informed consent form is read and explained, and after they have orally agreed to participate in the trial, and have either signed the consent form or provided their fingerprint, the witness should sign and personally date this consent form.

Witness to Consent Interview

On the date given next to my signature, I witnessed the "Informed Consent Interview" for the BK-SE36 research trial (Phase 1b, Stage _____). I attest that the information in these consent forms was explained to the volunteer [or volunteer's parent(s)/guardian(s)] and that the volunteer [or volunteer's parent(s)/guardian(s)] indicated that his/her questions and concerns were adequately addressed.

Signature of Witness ______________________________ Date ________________

Printed Name of Witness ____

Witness to Volunteers's Signature

On the date given next to my signature, I witnessed the volunteer [or volunteer's parent/guardian], sign his/her name or imprint his/her thumbprint(s) on the consent form(s).

Signature of Witness ______________________________ Date ________________

Printed Name of Witness ____

18.3.3 Consent form for identification photograph

CONSENT FORM FOR IDENTIFICATION PHOTOGRAPH:

I agree that the investigators may take photographs of me (or my child) for purposes of reliable identification during this research trial only. I also understand that, at trial completion, all such identification photographs will remain strictly confidential.

_______________________________________ ____________________

Signature of Person Giving the Consent Explanation Date

_______________________________________ ____________________

Printed name and Signature of Volunteer Date

____

Parents' / Guardian's Name and Signature (Relationship to Volunteer)

____

Parents' / Guardian's Name and Signature (Relationship to Volunteer)

Thumbprint of volunteer if unable to sign:	Thumbprint of parent or guardian if unable to sign:

18.3.4 Informed consent for future use of blood samples

INFORMED CONSENT FOR FUTURE USE OF BLOOD SAMPLES

Title: Single blind, randomized, controlled, Phase 1b trial of the safety and immunogenicity of lyophilized recombinant precipitated tropical malaria vaccine (BK-SE36), in Uganda

Trial Number: BK-SE36/002

Sponsor: BIKEN

Ugandan Principal Investigator: Prof. Thomas Egwang (+254-712-504 010; +256-775-494 752)

Date:

INTRODUCTION

While you (or your child) are in this trial, blood samples may be taken that may be useful for future research. These samples will be stored at Med Biotech Laboratories (MBL, Kampala) and at BIKEN Foundation (Japan). Samples may also be shared with investigators at other institutions.

WHAT SAMPLES WILL BE USED FOR

Your (or your child's) blood will be used to study malaria and the response of this disease to the test vaccine, BK-SE36. Results of these studies will not affect your (or your child's) care.

1. These samples will be used for future research (GIA, ADCI and epitope mapping) to learn more about malaria and other diseases.
2. Your (or your child's) samples will be used only for research and will not be sold or used for the production of commercial products.
3. No human genetic (DNA) studies will be undertaken with these samples. If additional studies are contemplated in the future, the investigators will first seek the approval of the MBL-IRC and UNCST.

LEVEL OF IDENTIFICATION

The samples obtained will be coded so that the subject's name cannot be readily identified. No information obtained from this research will be placed in any medical records.

In the future, researchers studying the samples may need to know more information such as age and gender and this information may be provided to the researcher. The

subject's name or any information that might be used to identify the subject personally will, however, not be provided.

RISKS

There are few risks from future use of the samples. A potential risk might be the release of information from the study records. The study records will be kept confidential as far as possible. Moreover, results of research done with the samples will not be put in any subject's health record.

BENEFITS

There will be no direct benefit to the subjects. From studying the samples we may learn more about malaria or other diseases: how to prevent them, how to treat them, how to cure them.

RESEARCH RESULTS/MEDICAL RECORDS

1. Results from future research using the samples may be presented in publications and meetings but subject names will not be identified.
2. Reports from future research done with the samples will not be given to you or any doctor. No report will be reflected in any subject's medical record.

QUESTIONS

The future use of the blood samples has been explained to you (and your child) by the person who signed below and your (or your child's) questions were answered. If you have any other concerns about the information here, you may call Prof. Thomas Egwang (+254-712-504 010; +256-775-494 752).

FREEDOM TO REFUSE

You (or your child) can withdraw this consent at any time. If you do, please contact Prof. Thomas Egwang (+254-712-504 010; +256-775-494 752) at MBL, Kampala. The samples will no longer be made available for research and will be destroyed. Whether or not you (or your child) will allow us to use the blood samples in future research will not have any effect on your (or your child's) participation in this trial.

WHAT YOUR SIGNATURE OR THUMBPRINT MEANS

Your signature (and your child's signature) or thumbprint below means that you understand the information that was given in this consent form. If you wish to allow the blood samples to be used for future research, you should sign or affix your thumbprint below.

Name of Volunteer (printed)

Signature of Volunteer | Date/Time

Name of Parent/Guardian | Relationship to Volunteer

Signature or Parent/Guardian | Date/Time

Language/Dialect for Informed Consent | Subject Code

Thumbprint of volunteer if unable to sign:

Thumbprint of parent or legal representative if unable to sign:

Name and Signature of Witness | Date

Signature of Person Giving the Consent Explanation | Date
