# Supplementary material for: Phase 1b Randomized Trial and Follow-Up Study in Uganda of the Blood-Stage Malaria Vaccine Candidate BK-SE36
Source: PLoS One. 2013 May 28;8(5):e64073. doi: 10.1371/journal.pone.0064073 (PMC3665850; doi:10.1371/journal.pone.0064073)
Supplement: Protocol S2 — Protocol for the follow-up study. (PDF) [file pone.0064073.s012.pdf]

Longitudinal study of the immunogenicity of BK-SE36 candidate malaria vaccine in children and young adults that participated in the BK-SE36 malaria vaccine trial in Uganda

This study will be carried out in accordance with the ICH HARMONISED TRIPARTITE GUIDELINE FOR GOOD CLINICAL PRACTICE E6(R1) (ICH-GCP) standards, regulatory authorities requirements and this protocol. Guidance for ethical principles were consulted in the:

Declaration of Helsinki: "Recommendations Guiding Medical Doctors in Biomedical Research Involving Human Subjects," adopted at the General Assembly of the World Medical Association held in Helsinki in 1964 (revised 2000 at Edinburgh, Scotland);

ICH Guidelines for Clinical Trials E6 (R1) CPMP/ICH/135/95 (updated 2002);

and "*National Guidelines for Research Involving Humans as Research Participants*," Uganda National Council for Science and Technology (UNCST) (Kampala, Uganda; March 2007);

The eligibility to conduct this trial, with regard to ethical and scientific validities, will be examined beforehand by the Scientific Committee/Institutional Review Committee of the participating institutions; and applied for approval to the UNCST. The IRC approval will take effect only after registration of this research project with the UNCST.

Version 1.0	1 October 2010	
1.1	22 October 2010	Updates on contact information of investigators
1.2	31 March 2011	In yellow highlights:<br>Updates on Co-investigator<br>Change in study period duration<br>Additional data collection tool: Daily body temperature recording<br>Additional assay for blood samples<br>*In italics*: minor grammatical changes

Local Principal Investigator: **Thomas Egwang, PhD**
Director General/Scientific Director
Med Biotech Laboratories, Plot 3438 Muyenga, Tank Hill By-pass,
PO Box 9364, Kampala, Uganda
Tel +254-712-504 010; +256-775-494 752

Investigators and Affliation:

Principal Investigator: **Thomas Egwang, PhD**
Director General/Scientific Director
Med Biotech Laboratories, Plot 3438 Muyenga, Tank Hill By-pass,
PO Box 9364, Kampala, Uganda
Tel +254-712-504 010; +256-785 605620
email: tgegwang@gmail.com

Co-investigators: **Adoke Yeka, MB. Ch.B., M.P.H.**
Uganda Malaria Surveillance Project,Makerere Medical School,
PO Box 7475, Kampala, Uganda
Tel +254-414-530 692; +256-772-473 533
Fax +256 414 540 524
email: yadoke@yahoo.com

Edward Hosea Ntege, MB.ChB.
MedBiotechLaboratories,
P.O. Box 9364, Kampala,Uganda
Tel + 256 783- 810228
email:edwardsdoc@yahoo.com

Betty Balikagala, MB.ChB.
MedBiotechLaboratories,
P.O. Box 9364, Uganda
Tel +256-772565506
email: balikagalabetty@yahoo.com

Japan Counterpart :

Toshihiro Horii, PhD
Professor, Department of Molecular Protozoology
Research Institute for Microbial Diseases (RIMD), Osaka University
3-1 Yamadaoka, Suita, Osaka 565-0871, Japan
Tel +81(6)-6879-8280; *Fax* +81(6)-6879-8281

Clinical Trial Site:

Lira Medical Centre
P.O. Box 1075, Plot #15, Moroto Road, Lira, Uganda
Tel +256-392-948 833; +256-772-419 397
http://www.lira-medical-centre.org/

Table of contents

A. Introduction......5

A.1 Background......5

A.2 Justification......7

A.3 Objectives 8

A.4 Endpoints 8

B. Study design and population 8

B.1 Overview or Design Summary 8

B.2 Study Population/Eligibility Criteria 9

C. Subject Recruitment Plans and Consent Process 11

D. Detailed Description of Study Visits......11

E. Statistical considerations 12

F. Use, Storage, and Tracking of Specimens (blood samples) and Data 13

G. Protection of Human Subjects 13

G.1 Institutional Review Board 13

G.2 Informed Consent 13

G.3 Precautions 13

G.4 Benefits......13

G.5 Payment......14

H. Confidentiality 14

I. Publication 14

J. References......14

K. ICF for Volunteer; and/or Parent(s)/Guardian(s) of Volunteers 16

L. ICF for Identification Photograph 21

M. ICF for Future Use of Blood Samples 22

N. Questionnaire for Volunteers 24

O. Appendix 1. Justification for Study Objectives and Methodology 26

SYNOPSIS

1. Title: ***Longitudinal study of the immunogenicity of BK-SE36 candidate malaria vaccine in children and young adults that participated in the BK-SE36 malaria vaccine trial in Uganda***

2.* Objectives*:

a) To compare long term immunogenicity of BK-SE36 malaria vaccine and antibody response to natural malaria infections in children who participated in the BK-SE36 malaria vaccine trial in Uganda.

b) To compare malaria incidence rates and severity between volunteers who received BK-SE36 vaccine (at half or full dose) and those who received placebo or no administration (control group).

3. Outcome:

1) antibody response as a result of natural malaria infections; **2**) malaria incidence; **3**) treatment incidence density (treatments per time at risk); **4**) prevalence of malaria parasitemia and parasite density for each group that received BK-SE36 vaccine (at half or full dose) and those who received placebo or no administration (control group); **5**) change in antibody titres/epitope recognition against synthetic short peptides *spanning the whole length of* SE36.

4. Study site: Lira Medical Centre (LMC), Lira, Uganda

5. Study design: Longitudinal observational study in children who participated in the BK-SE36 malaria vaccine trial in Uganda.

6. Participants: BK-SE36 Phase1b Stage2 study subjects (6-20 y old) who give consent for additional 11 months follow-up period, AND additional volunteers for recruitment to achieve an equal number of subjects in the control group (*i.e.* BK-SE36 cohort *(n=66)* = placebo cohort + *additional volunteers (n=66)*). These additional volunteers that did not participate in Phase 1b Stage2 trial will not be receiving any intervention/administration.

7. Number of participants: A total of 132 participants.
(33) subjects = administered with half dose SE36 (50 μg)
(33) subjects= administered with full dose SE36 (100μg)
(9) subjects = administered with half dose of placebo (0.5mL physiological saline)
(9) subjects = administered with full dose of placebo (1.0mL physiological saline)
(48) additional volunteers to be recruited into the control group (no administration)

8. Expected start date: End of 40 days surveillance period of Phase 1b, Stage2

9. Primary completion date: 11-months after study start

10. Overview based on approved BK-SE36 Phase 1b trial, Stage2:

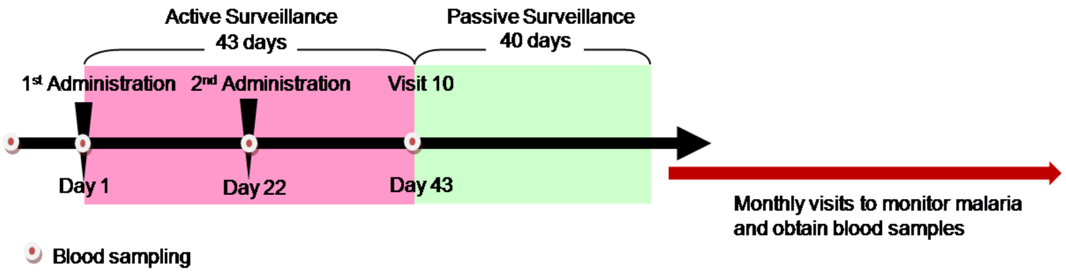

A. INTRODUCTION

A1. Background:

Burden of Malaria in Sub-Saharan Africa and Uganda. Malaria is Africa's leading cause of mortality in children under five years of age and is responsible for 10% of the overall disease burden, 40% of public health expenditure, 30-50% of inpatient admissions, and up to 50% of outpatient visits (www.rbm.who.int). There are several reasons why Africa bears a large proportion of the world's malaria burden. First, most malaria infections in sub-Saharan Africa are due to *Plasmodium falciparum*, the cause of the most difficult to treat and severe form of the disease. Second, this region is also home to the most efficient malaria mosquito vectors, members of the *Anopheles gambiae* complex and those of the *An. funestus* complex. Third, most African countries are "the poorest of the poor", lacking the basic infrastructure and resources necessary to mount sustainable malaria control efforts. Uganda, where the proposed work would be located, is emblematic of the immense problem that malaria poses for African countries. Malaria is endemic in over 95% of the country, with some of the highest malaria transmission intensities reported in the world [1]. According to the 2008 World Malaria Report, Uganda ranked 6th in terms of number of malaria cases and 3rd in terms of the number of malaria deaths [2]. Malaria is the leading cause of morbidity and mortality in Uganda and is responsible for up to 40% of all outpatient visits, 25% of all hospital admissions and 14% of all hospital deaths (Uganda Ministry of Health, unpublished). The overall malaria-specific mortality is estimated to be between 70,000 and 100,000 child deaths annually, a death toll that far exceeds that of HIV/AIDS [3]. A 1995 Burden of Disease study indicated that 15% of life years lost to premature death was due to malaria and that families spend 25% of their income on malaria prevention and treatment (Uganda Ministry of Health, unpublished). Poor school performance and absenteeism due to malaria reduce chances of escaping from poverty [4]. Poor people tend to live in environments conducive to mosquito breeding and malaria transmission. Thus malaria enhances poverty, which in turn causes poor disease management, locking people in a malaria-poverty trap [5].

Epidemiology of Malaria in Sub-Saharan Africa. Malaria in sub-Saharan Africa is largely characterized by high rates of infection and the development of partial immunity after repeated exposure. Transmission intensity ranges from under ten to several hundred infective bites per person year, but the majority of these infections do not lead to clinical illness. The risk of developing symptomatic disease is inversely proportionate to the level of acquired immunity. Partial immunity develops through repeated exposure, leading first to protection against severe forms of disease, followed by protection against symptomatic illness [6]. The result of this phenomenon is that the burden of malaria in Africa is heavily borne by young children. Newborns are protected during the first few months of life due to the transplacental acquisition of maternal antibodies and relatively high hemoglobin F content in the infants' erythrocytes [7]. After about 3-4 months of age, protection from these factors wanes, and the infant becomes highly vulnerable to malaria, including severe disease, with an estimated 75% of malaria deaths occurring in African children under the age of 5 years [8]. However, the age at which malaria risk peaks in endemic areas of Africa varies from 1-2 years of age in areas of high transmission intensity to approximately 5 years of age in areas of low to moderate transmission intensity [9]. The risk of symptomatic malaria declines as children get older, however, school aged children are at highest risk of asymptomatic parasitemia, with chronic parasitemia resulting in anemia [10-12]. Malaria parasitemia may also impact significantly on the education and learning of school-aged children [10]. However, as the intensity of malaria transmission declines due to control efforts, it is suggested that clinical immunity will be acquired more slowly and disease burdens will shift into older age groups [13]. In addition, the high

prevalence of parasitemia in children (up to 90% at any time) creates a large reservoir for transmission [14].

***Plasmodium falciparum* and Malaria Vaccine Candidates.** *P. falciparum,* responsible for the majority of malaria deaths, is a multistage parasite invoking stage-specific host immune responses. To date, the approach to malaria vaccine development has primarily concentrated on eliciting these stage-specific immune responses. Thus, malaria vaccine candidates can be divided into (1) those that stimulate a pre-erythrocytic (sporozoite or liver) immune response, (2) those that block malaria transmission by promoting an antibody response against antigens expressed in the sexual stage within the midgut of the mosquito, (3) those that target the asexual blood stages of the parasite, with vaccines reducing the toxic effect of the infection.

Natural immunity against malaria appears to be acquired over time, requires multiple infective bites, and is lost in the absence of continued exposure. The goal of blood stage malaria vaccines is to protect against disease and death, which can be achieved through acquisition of such immunity. In spite of tremendous efforts to develop malaria vaccines for decades, an effective vaccine is not yet available.

The Malaria Vaccine Candidate BK-SE36. It has been demonstrated that recombinant N-terminal domain of *P. falciparum* serine repeat antigen 5 (SERA5), SE36, is a potential erythrocyte stage malaria vaccine candidate [15]. Previous immuno-epidemiological studies in Apac show that natural antibody responses to SE36 are widespread and age-dependent with adult population roughly divided into two caterogies: sero-positive or sero-negative to SE36 antigen. An epidemiological study in malaria patients from holo-endemic Solomon Islands suggests that there is significant correlation between lower parasitemias and higher IgG titers against SE36. An experimental study using squirrel monkeys demonstrated that vaccination with SE36 protein and aluminum hydroxide conferred protection against high parasitemia and boosted serum anti-SE36 IgG after *P. falciparum* challenge. The challenge infection also changed the epitope recognition of anti-SE36 IgG to a recognition pattern similar to those observed in people with naturally acquired immunity. In chimpanzees, SE36 plus alum was highly immunogenic with antibody titers lasting more than a year. These observations provided the impetus *for the* development of BK-SE36 *as a malaria vaccine* [15].

The target population for this particular vaccine are children at risk for clinical disease (including severe disease) due to infection with *P. falciparum*. Thus in 2005, the Research Foundation for Microbial Diseases of Osaka University launched Phase 1a clinical trial of BK-SE36 in Tsukuba City, Japan (http://www.controlled-trials.com/isrctn/pf/78679862). The vaccine was tested in malaria naive adults and *confirmed* as safe and immunogenic. Moreover, a dose was identified for further evaluation. In April 2010, a Phase 1b clinical trial commenced in Lira, Uganda (http://www.controlled-trials.com/ISRCTN71619711). The trial was divided into two stages. In the first stage, 56 healthy subjects aged 21-40 years were randomized to receive either BK-SE36 or placebo (physiological saline) twice at 3 weeks interval. *Volunteers were* followed up to more than 7 weeks after the second administration and the test vaccine at 100 μg dose was found to be safe and well tolerated. The study in children population (Stage2) is ongoing at Lira Medical Centre (LMC), Lira, Uganda and targets 84 healthy subjects aged 6-20 years.

Study hypothesis.

For continued development of BK-SE36, we propose to study the long term immunogenicity of BK-SE36 malaria vaccine and antibody response to natural malaria infections and to compare malaria

incidence rates and severity between volunteers who received BK-SE36 vaccine (at half or full dose) and those who received placebo or no administration (control group). We hypothesize that:

(1) BK-SE36 cohorts will show higher antibody titres after malaria natural infection than right after administration of BK-SE36. This phenomenon is known as a natural booster effect and an important element to be considered for vaccine strategy;
(2) BK-SE36 cohorts will show higher antibody titres against the protective epitopes in SE36 molecule; and
(3) Malaria incidence, severity and treatment incidence density will be lower in participants who received the BK-SE36 vaccine compared to the controls who received physiological saline or no intervention

A2. Justification/Significance of the Study:

A2.1. Confirmation that SE36 malaria vaccine induced immunity can be boosted by natural infection shall give an insight into whether the vaccine can provide protection in a population exposed to natural infection with genetically heterogenous *P. falciparum*. Longitudinal observational study of anti-SE36 antibody titer, with monitoring of natural malaria infections has not yet been done both in children and adults. A longitudinal observational study comparing naturally acquired antibody responses to several malaria antigens (e.g. MSP-, AMA, etc) has not yet been carried out in a study population that has been administered with BK-SE36. The proposed study will address this gap. We anticipate that the results of this study will help in understanding immune responses due to vaccination by having appropriate malaria antigens for control/comparison.

A2.2. The study shall provide information to elucidate the duration of vaccine evoked immunity.

A2.3. The study shall provide information on the possible shift of vaccine induced epitope recognition of anti-SE36 IgG after natural infection. The change of immune status can be monitored by the comparison of reactivity of antibodies against synthetic peptides covering the entire region of the SE36 molecule between vaccinated serum without malaria infection and with malaria natural infection. The epitope recognition shift of antibody by natural infection is a new concept in malaria vaccine research. If the phenomenon is observed in humans, this *might* give an insight that the vaccine can provide protection in a population exposed to natural infection.

A2.4. We shall gather information on specific alleles of SERA5 gene that may escape from the vaccine immunity (by analyzing DNA of the parasites after vaccination and natural infection).

In this study, antibody titre and blood parasitemia of each subject will be longitudinally monitored. In addition, it would be a very important manifestation and valuable data if an increase of antibody titer is observed without malaria episode. Defining clinical endpoints for malaria vaccines has proved challenging. Blood stage vaccines are intended to block disease and not infection. Unfortunately, there are no defined protective antibody thresholds, and in the absence of an objective immune assay, the endpoint of a blood stage vaccine is often reduction of a carefully defined clinical illness specific to the site at which the trial is conducted. Likewise, there has also been insufficient capacity to definitely diagnose many suspected malaria cases since malaria disease has a variety of clinical symptoms, broad range of parasitemia, and maybe confused with other illnesses that occur in the setting of baseline levels of malaria infection. We need to define accurate interpretations of malaria disease to accurately monitor the next stages of clinical trial that defines efficacy. A definitive criteria will provide an accurate picture of the malaria situation, and guide further vaccine/treatment decisions.

The anticipated observations would propel further clinical trials.

A3. Objectives

A3.1. To analyze the antibody productions quantitatively and qualitatively in volunteers who experiences natural malaria infection, and those who were not able to experience malaria infection in both vaccinated and non-vaccinated groups for obtaining a baseline data of biological effects.

A3.2. To compare malaria infection rates and severity in both of vaccinated and non-vaccinated groups for obtaining a baseline data of vaccine efficacy.

A3.3. To undertake SERA5 polymorphism study of *P. falciparum* in severe malaria incidences

A4. Endpoints: **Primary**

A4.1. Change in titers of anti-SE36 antibody, parasitemia rates, malaria incidence, malaria treatment incidence density

Secondary

A4.2. Change in antibody titers/epitope recognition against synthetic short peptides *spanning the entire sequence of* SE36. Change in antibody titres relative to other malaria antigens (e.g. MSP-1, etc) may also be used for comparison to obtain further insights about immune responses/dynamics of *P. falciparum* infection in the study population.

A4.3. Possible correlation of parasite growth inhibition in a system of Antibody Dependent *P. f.* Cellular Inhibition (ADCI) by the induced antibodies and *in vivo* malaria protection.

A4.4. *P. falciparum* SERA5 gene allelic types in vaccinated group.

A4.5. Other biological analysis with the obtained sera.

B. STUDY DESIGN AND POPULATION

B1. Overview or Design Summary

Study design: Longitudinal observational follow-up study.

Trial Site. The study will be conducted at Lira Medical Centre (LMC, Lira, Uganda) in collaboration with Med Biotech Laboratories (MBL). LMC has been the site of the BK-SE36 trial and MBL has been the collaborating institution and local counterpart in Uganda. Lira Medical Centre (LMC) has been chosen as the trial site based on infrastructure, logistics and the standard health care it provides to the community and neighboring counties. The Centre itself is readily accessible by foot or ground vehicle to all perspective trial volunteers. It has an outpatient and inpatient facility, surgical theatres, has a clinical laboratory equipped to carry out basic laboratory assays (blood counts and serum

chemistries), a pharmacy, and is staffed 24 hours/day and 7 days/week. At present, the Centre is the primary provider of medical care in Lira and neighboring towns since its establishment in 2005. As a private, non-governmental organization hospital the Centre provides care free-of-charge to the community for immunization, tuberculosis drugs, blood transfusion, ante-natal care and counseling, routine malaria check for pregnant women, mosquito bednets and vitamins. For the clinical trial of Phase 1b, the whole third floor of the hospital building was renovated to include blood collection rooms, pharmacy, three private consultation rooms, waiting and observation area, vaccination room, storage and administrative space. During the clinical trial, the trial floor operated 24 h a day, 7 days a week. The team has been trained to conduct GCP-compliant studies, using source documents, written informed consent and standardized case report forms. The trial team has also been establishing a strong trust and rapport with the community, enhancing the local capacity of the community as well as being a health care provider to volunteers. The site is connected to MBL Laboratories in Kampala, Quintiles services in South Africa, Kenya, Dublin and to the sponsor via a VSAT system, which also allows faster communication links and the Internet.

B2. Study Population/Eligibility Criteria:

Background Intervention of target population:
B2.1 BK-SE36 treated cohorts: 2 dose arms (full- and half-dose); doses administered twice at 21-day interval

B2.2 Placebo treated cohorts: 2 dose arms (0.5 and 1.0mL of physiological saline); doses administered twice at 21-day interval

B2.1 and C2.2 are subjects of the Stage2 BK-SE36 Phase1b trial who will be followed-up for antibody titers and malaria infection incidences.

B2.3 *Control group*: no intervention [This is equivalent to placebo-treated cohorts. Due to the length of follow-up and the possibility of subject drop-out, additional subjects are needed for comparison to BK-SE36 cohorts]. To serve as appropriate control group (taking into consideration that an almost equal opportunity for malaria infection should occur between cohorts), the inclusion and exclusion criteria for the BKSE36 malaria vaccine Stage2 study applies:

[Inclusion criteria 2]: Stage2, 6 to 20 y old

Healthy subjects are specified, and the inclusion criteria of malnutrition index and laboratory test values were included to reduce individual variation.

<2.1> Volunteers, irrespective of gender, aged 6 to 20 years (age on informed consent)
<2.2> Those who do not suffer from severe malnutrition, whose BMI is between 5th percentile to less than the 85th percentile for 6-19 y old; and between 18.5-25.0 for 20 y old;
<2.3> Those who can give affirmative agreement to participate in the trial. For children between 8 to 17 y, the child's assent takes precedence over the parent(s)/guardian(s) consent.
<2.4> Those who are able to agree, comply with matters to be observed during participation in the trial, undergo consultation/examination, as described in this protocol, and report symptoms;
<2.5>Those who are considered to be eligible to participate in this trial based on screening:
<2.5.1> Vital signs and physical examination are within baseline range
<2.5.2> Hematology: Within 25% deviations from the upper and lower limits of the baseline range. The differential white blood count is not questioned when the white blood cell count is within the

baseline range.

<2.5.3> Blood chemistry:

- AST, ALT, and creatinine: Within the baseline range
- Total bilirubin: Within 50% deviation from the upper limit.
- Serum electrolytes: Within the baseline range
- Other blood chemistry items: Within 25% deviation from the upper and lower limits of the baseline range.

<2.5.4> Urinalysis: Within the normal range

[Exclusion criteria 2]:

Any subject meeting any of the exclusion criteria at baseline will be excluded from trial participation.

<1> Persons with fever (37.5°C or higher) on administration of the test vaccine;
<2> Persons with a clear history of food/drug-related anaphylaxis;
<3> Females (adolescents/adults) who are pregnant or have a positive urine β-hCG on the day of, or prior to, administration;
<4> Females currently lactating or breast-feeding;
<5> Persons with acute or chronic cardiovascular, pulmonary, hepatic, renal, or neurological condition, which in the opinion of the investigator may increase the risk of the subject from participating in the trial;
<6> Persons with a history of fever within 2 days after preventive administration with other types of vaccine, or those in whom symptoms have suggested systemic allergy;
<7> Persons with a history of convulsion other than febrile convulsions in malaria in the past 6 months to 1 year
<8> Persons with any confirmed or suspected immunosuppressive or immunodeficient condition, including human immunodeficiency virus (HIV) infection. (No infectious disease testing will be conducted. HIV testing will not be done. Severe, suspected infectious diseases will be ruled out by investigators during physical examination/consultation, blood hematology/chemistry tests; although not conclusive of the causative agent.);
Additional oral confirmation: Subject informed the investigator that he/she has been tested positive for HIV/AIDS. (Information on the child's HIV status could be obtained from their parents or guardians.)
<9> Persons with a history or tentative diagnosis of drug allergy; especially to common drugs like penicillin, sulphonamides, etc.
<10> Persons with (history of) chronic alcohol consumption and/or illicit drug use;
<11> Persons who took any medication within 1 week before administration of this test vaccine (except for artemether/lumefantrine and dihydroartemisinin-piperaquine);
<12> Persons to whom any live vaccine was administered within 4 weeks before administration of this test vaccine, or inactivated vaccine/toxoid was administered within 1 week;
<13> Persons who participated in another trial within 4 months before administration of this test vaccine; or simultaneous participation in any other clinical trial;
<14> Persons in whom 200 ml of blood was collected (donation) within 1 month before administration of this test vaccine, or more than 400 ml of blood was collected within 3 months;
<15> Persons who have recently undergone blood transfusion in the last 3 months.
<16>Others who are not considered to be eligible by the investigator or those, whose medical condition would, in the opinion of the investigator, make the subject unsuitable for the trial.

C. Subject Recruitment Plans and Consent Process

There are two routes for volunteer screening:

i. For subjects who participated in the Stage 2 trial.

On Visit 10, subjects are invited to participate to this follow-up research and written informed consent is solicited: Explain the study and Informed consent document (ICD) to the subject; ensure that the subject signed and received a copy of the ICD. The number of subjects who gave informed consent and their place of residences will be noted and takes an important consideration for the second route of volunteer screening-- the screening for control cohorts.

ii. For volunteers who will serve as additional control cohorts.
After obtaining the number of subjects willing to participate in the one-year follow-up at Visit10, during the 40-day passive surveillance, healthy volunteers will be recruited to make up the control arm:

Explain the study and ICD to the volunteer; ensure that the volunteer signed and received a copy of the ICD

Elicit a complete medical history; administer a complete physical examination; obtain blood for hematology, biochemistry, malaria smear; obtain urine for urine dipstick testing, as well as urine β-hCG testing for females. *To be an appropriate/adequate control group, the volunteers to be enrolled should essentially be representative of the population from subjects that gave consent to participate in the longitudinal study.*

These individuals will receive oral and written explanation of the study, after which consent will be obtained from those willing to participate. All screening tests, medical history and examinations will be performed only after consent is obtained. Individual consent process will be conducted in separate consultation rooms to ensure confidentiality. Any clinically relevant finding that is discovered upon screening will either be treated appropriately or referred for more comprehensive diagnosis and treatment. Study clinicians will generally handle acute, simple conditions such as malaria (or other infections) but more complicated or chronic renal or heart disease will be referred to appropriate sources of medical care. Volunteers will provide a medical history, with special attention to any history of recurrent infections to suggest immune suppression. They will also undergo physical examination and laboratory screening tests. A participant who meets any of the exclusion criteria will be managed initially by study clinicians and referred to the local health center for evaluation as necessary.

D. Detailed description of study visits:

Study practice: Medical interview, consultation and serum sampling once every four weeks for 11 months

At each monthly visit:

1 Consultation/examination of injection site (for administered subjects) if there are unresolved AEs
2 Consultation interview for recent illness, malaria symptoms; includes capturing malaria incidences between visits
3 Vital signs (temperature, blood pressure, pulse rate)
4 A few drops of blood sampling (by finger prick) for malaria smear, filter paper blots, and ELISA assay.
5. Blood draw (4 mls) of venous blood for serum sampling

Subjects will be assigned to fall into different consultation days. Clinic visits will be in groups. Care should be exercised whenever possible that the BK-SE36 administered cohort group can be matched to a control cohort group in the same locality for equal "bias" in malaria incidence rates.

During this follow-up period, adverse events will not be reported but malaria episodes will be documented and blood samples collected for immunological testing. Daily body temperature monitoring and recording by participants or parents /guardians will be carried out by the participants themselves at home throughout the study period. Training of participants and /or parents/guardians in the use of thermometers, reading and recording of body temperatures will be conducted during the start of the study

At the final visit:
Blood sampling (20-ml venous blood) for ADCI and ELISA assays

Passive follow-up for malaria will consist of visits to LMC trial site whenever the subject is sick. During each visit, the subject will be evaluated and a malaria smear will be done whenever malaria is suspected. During the course of evaluation for malaria, other illnesses maybe uncovered in the study subjects, such as upper respiratory infections, and other bacterial or viral infections. The pharmacy can provide subjects with common over-the-counter analgesics such as paracetamol as well as antibiotics for the treatment of minor ailments and infections. If further evaluation or treatment is necessary, the subject will be referred to the hospital arm in LMC.

Other trial activity/Blood sampling:

A few drops of blood will be taken from all subjects for the following:
1. Parasitemia determination: for confirmation of parasitemia by standard microscopy
2. Filter paper blood spots: A thick blood smear is made. *P. falciparum* DNA will be amplified for molecular genotyping of SERA gene polymorphism)
3. Serum preparations for *ELISA measurements on antibody titres against* SE36 as well as several malaria antigens

Blood drawn at the final visit shall be used for the following assays as well:
4. ELISA Assays with synthetic peptides covering SE36 molecule (epitope recognition)
5. Growth inhibition studies (ADCI)
6. Other biological studies, such as HLA haplotype typing

E. Statistical considerations

The purpose of this study is to estimate malaria episodes, patterns of immune responses as well as compare these rates and patterns in different doses/interventions of the study based on the Phase 1b, stage2 protocol. Listings will show all observed data. Should there be a need for early termination of the study, available data will be analyzed and interpreted in light of early termination.

To assess and compare the duration of specific antibody response to SE-36 over a 11 month period:

1. A test will be performed to check for any significant differences in the antibody concentrations of the two dose groups.

2. Immunogenicity responses by vaccine and dose group will be described over time. Individual responses can also be described over time and stratified by dose cohort.

3. For antibody concentrations measured, a longitudinal model will be built to describe the antibody response over time. The model will also explore if there are any differences between the two doses used for Stage2.

4. Waning will be assessed by comparing levels of antibody with time, as well as malaria infection.

5. Average parasitemia in all samples collected during the whole period will be compared between the vaccine and placebo group (including number of incidences of mild and severe malaria in BK-SE36 vs. control groups). Kaplan-Myer plots will be used to compare the immunological protection between blood parasitemia levels and administration of BK-SE36

F. Use, Storage, and Tracking of Specimens (blood samples) and Data

Samples and data collected under this study will be used to study malaria, long term protection/ immunity as induced by BK-SE36, and (whenever possible) molecular genotyping of *P. falciparum* in the study area. Access to the blood samples and data will be limited either using a locked room, a locked freezer, or a locked cabinet. Only investigators or their designees will have access. Any loss or unanticipated destruction of specimens (for example, due to freezer malfunction) or data (for example, lost of printed data or original source document with identifiers) will be reported to MBL-IRC and RIMD, Japan. Serum samples will be kept both in LMC, Lira and in RIMD, Japan; the transportation and storage of which will be tracked. Storage of all study-related documents will be such that confidentiality will be strictly maintained.

G. Protection of Human Subjects

G.1.Institutional Review Board/Ethics Committee

IRB approval will be obtained for the study. Documents will be submitted to the IRB as well as to the UNCST.

G.2.Informed consent

In obtaining and documenting informed consent, the Investigator complies with applicable regulatory requirements, Good Clinical Practices and ethical principles. The written informed consent form is to be approved by the IRB prior to use.

G.3. Precautions

None.

G.4.Benefits

There will be no direct benefit from participation in this study. It is hoped that the information gained will contribute to the development of a safe and effective malaria vaccine.

G.5. Payment

Subjects will be paid via LMC based on Ugandan standards. Compensation will cover for time spent, meal (s) and travel costs at each visit. Details will be available in the Informed Consent Documents.

H. Confidentiality

Subjects will be assigned a unique subject code. All results will be linked to this number. Trial records will only be available to staff members and all related study information will be stored securely at the trial site. Access to files may be reviewed by representatives from RIMD, MBL-IRC, UNCST for monitoring purposes.

I. Publication

It is anticipated that results of this study will be presented to the scientific community via oral presentations at meetings and written publications in scientific journals. The data to be presented and the authorship will be discussed between investigators and approved prior to any official communication.

J. References

1. Okello PE, Van Bortel W, Byaruhanga AM, et al. Variation in malaria transmission intensity in seven sites throughout Uganda. Am J Trop Med Hyg 2006;75:219-25

2. Organization WH. World malaria report 2008, 2008

3. Lynch KI, Beach R, Asamoa K, Adeya G, Nambooze J and Janowsky E. President's Malaria Initiative, Rapid Assessment Report - Uganda, 2005

4. Lalloo DG, Olukoya P and Olliaro P. Malaria in adolescence: burden of disease, consequences, and opportunities for intervention. Lancet Infect Dis 2006;6:780-93

5. Sachs J, Malaney P. The economic and social burden of malaria. Nature 2002;415:680-5

6. Rogier C. Natural history of Plasmodium falciparum malaria and determining factors of the acquisition of antimalaria immunity in two endemic areas, Dielmo and Ndiop (Senegal). Bull Mem Acad R Med Belg 2000;155:218-26

7. White NJ. Intermittent presumptive treatment for malaria. PLoS Med 2005;2:e3

8. Snow RW, Craig M, Deichmann U and Marsh K. Estimating mortality, morbidity and disability due to malaria among Africa's non-pregnant population. Bull World Health Organ 1999;77:624-40

9. Rogier C, Tall A, Diagne N, Fontenille D, Spiegel A and Trape JF. Plasmodium falciparum clinical malaria: lessons from longitudinal studies in Senegal. Parassitologia 1999;41:255-9

10. Clarke SE, Jukes MC, Njagi JK, et al. Effect of intermittent preventive treatment of malaria on health and education in schoolchildren: a cluster-randomised, double-blind, placebo-controlled trial. Lancet 2008;372:127-38

11. Koukounari A, Estambale BB, Njagi JK, et al. Relationships between anaemia and parasitic infections in Kenyan schoolchildren: a Bayesian hierarchical modelling approach. Int J Parasitol 2008;38:1663-71

12. Kurtzhals JA, Addae MM, Akanmori BD, et al. Anaemia caused by asymptomatic Plasmodium falciparum infection in semi-immune African schoolchildren. Trans R Soc Trop Med Hyg 1999;93:623-7

13. Snow RW, Marsh K. The consequences of reducing transmission of Plasmodium falciparum in Africa. Adv Parasitol 2002;52:235-64

14. Talisuna AO, Langi P, Bakyaita N, et al. Intensity of malaria transmission, antimalarial-drug use and resistance in Uganda: what is the relationship between these three factors? Trans R Soc Trop Med Hyg 2002;96:310-7

15. Horii T, Shirai H, Jie L, Ishii KJ, et. al. Evidences of protection against blood-stage infection of Plasmodium falciparum by the novel protein vaccine SE36. Parasitology International, 2010; 59:380-6.

Malaria Links for Uganda :

http://www.who.int/malaria/world_malaria_report_2009/en/index.html. World Malaria Report 2009.World Health Organization. ISBN 978 92 4 156390 1

http://whqlibdoc.who.int/publications/2009/9789241563901_eng_Profiles.pdf. Profiles: 31 high-burden countries. World Health Organization, World Malaria report 2009.

http://www.malariaconsortium.org/pages/uganda.htm. Malaria Consortium: Uganda. 2010

Clinical trial links for BK-SE36:

http://www.controlled-trials.com/isrctn/pf/78679862. Clinical trial registration for Phase1a.

http://www.controlled-trials.com/ISRCTN71619711. Clinical trial registration for Phase1b.

ICF for Volunteer; and/or Parent(s)/Guardian(s) of Volunteers:
For Volunteers 6-7 y old, needed consent (signatures or thumbmarks) are from parents/guardians
For Volunteers 8-17 y old, needed consent (signatures or thumbmarks) are from both volunteer and parent/guardian
For Volunteers 18-20 y old, needed consent (signatures or thumbmarks) are from volunteer themselves

TITLE: Longitudinal study of the immunogenicity of BK-SE36 candidate malaria vaccine in children and young adults that participated in the BK-SE36 malaria vaccine trial in Uganda

INSTITUTIONS:
1. Lira Medical Centre (LMC), Lira, Uganda
2. MedBiotechLaboratories (MBL), Kampala, Uganda
3. Research Institute for Microbial Diseases (RIMD), Osaka University, Japan

LOCAL PRINCIPAL INVESTIGATOR:
Thomas Egwang, PhD
Director General/Scientific Director
Med Biotech Laboratories, Plot 3438 Muyenga, Tank Hill By-pass,
PO Box 9364, Kampala, Uganda
Tel +254-712-504 010; +256-785-60 5620

CLINICAL TRIAL SITE:
Lira Medical Centre
P.O. Box 1075, Plot #15, Moroto Road, Lira, Uganda
Tel +256-392-948 833; +256-772-419 397
(c/o Dr. Ogwang Ochoo Ben, Director, LMC)
http://www.lira-medical-centre.org/

MBL-IRC:
Professor Edward K. Kirumira (Chairman)
Dean, Faculty of Social Science, Makerere University, Kampala
Tel +256 752 767 439
kirumira@starcom.co.ug

Flavia Zalwango K (Secretary)
MRC, UVRI Uganda,
Tel +256 782 094 273
zaly14@yahoo.com

Participant information:
We would like to invite you to participate in a follow-up clinical research for Stage 2, Phase 1b trial. As a volunteer/parent/guardian we ask your understanding for the following information that applies to all volunteers in our studies:

1. Participation is entirely voluntary
2. If you do not like (or your child does not like) to take part in this study it will not affect your child's current or future medical care in LMC. Your refusal for your child's participation will involve no penalty or loss of benefits to which your child is entitled.
3. You may withdraw your child's participation at anytime. If you decide to withdraw your child during the course of the study, please inform any staff or meet with the principal investigator.
4. For volunteers 8 to 17 years old, we need BOTH parent/guardian and child to agree for participation to the trial. Your child's agreement is important such that even if you do agree but your child will not agree we cannot allow participation to this trial.
5. The study will take 12 months, and you and your child needs to be available during that time. You (and your child) will have to come for monthly visits (once per month) to LMC.
6. After this explanation, please feel free to ask anything you do not understand.
7. If your child has HIV, it would be advisable for him/her not to participate in the trial.

Introduction/purpose:
Malaria is a disease that affects many people throughout the world, including Uganda. It is caused by parasites that are transmitted by mosquito bites. Inside the human body, the parasites multiply in the liver, and then infect red blood cells. Symptoms of malaria include fever, headache and vomiting, and if not treated can quickly become life-threatening. In many parts of the world, the parasites have developed resistance to a number of malaria medicines.

Investigators at the Research Institute for Microbial Diseases in collaboration with The Research Foundation for Microbial Diseases of Osaka University have developed an experimental vaccine against malaria called BK-SE36. BK-SE36 contains a protein derived from malaria parasites that can prevent people from getting sick with malaria but it is not approved yet for general use. Normally, before a vaccine can be sold to market, it has to be tested in several clinical trials to see the safety and the ability of the vaccine to work (or its effect on the body). These trials are done with extreme care/concerns, strictly following guidelines to be able to evaluate thoroughly the new vaccine. So far we have tested this vaccine in two study populations in LMC, 21-40 and 6-20y old. In this study, for continued development, our purpose is to compare the number of children/adults who get malaria after receiving BK-SE36 to the number of children/adults who get malaria after receiving placebo (or those not getting any vaccination).

This study has been approved by the MBL-Institutional Review Committee (MBL-IRC). MBL-IRC is an independent committee in Uganda that ensures the protection of people's right, safety and well-being in clinical research.

We hope that information from this study can be used to develop a vaccine that in the future will help protect people from getting sick with malaria.

PROCEDURES TO BE FOLLOWED:
If you/your child participated in the Stage 2 clinical trial, we would like to solicit again your informed consent for your child to participate in this follow-up study.

If you/your child have not participated in our Stage 2 clinical trial, we will set a date for your child to come back for an appointment with one of the doctors. During that appointment (screening day):
1. we will ask for your child's medical history; and

2. ask your child to undergo a medical examination. We will do a physical examination and do tests on blood and urine to look for any signs of illness in the blood, kidneys or liver. We will draw about 1.6 teaspoon of blood (8 ml) to evaluate your child's health condition. You will be told of all the test results and the possible meaning of these results.   If we find anything wrong that will not allow your child to participate in the trial, we will refer your child to the other doctors at LMC.

If your child is fine, we seek consent for a visit to the Centre every 4 weeks for a period of 12 months. At each visit, we will do consultation/interview of past illness, physical examination and vital signs. We will also need to draw blood for malaria smear, filter paper blots and other assays (ELISA and HLA haplotype typing for example) to help us see the effect of malaria infection and compare them to SE-36 vaccinated groups and non-SE36 vaccinated groups (blood volume for monthly visits: 4mL; blood volume at final visit: 20mL).   We need a separate consent for blood samples. If your child becomes ill from malaria during non-scheduled visits, we may have to prick to confirm diagnosis before we do treatment.

We also wish to inform you that your child's health condition may also be fine but due to the study design we might not be able to accommodate him/her as a participant. We hope for your understanding.

Potential risks and discomforts
Blood sampling. Drawing blood might cause discomfort and occasional bruising at the site. The amount of blood to be removed will be too small to affect your child's health.

RESTRICTIONS
While taking part in this study, you should not take part in any other research project without approval from the people in charge of the study.

Benefits:
1. For now, your child may not have any direct benefit. BK-SE36 is a candidate malaria vaccine that might help protect persons from malaria, but it is not proven yet.
2. Follow-up for malaria will consists of visits to LMC trial site whenever the subject is sick. The pharmacy will provide subjects with common over-the counter analgesics such as paracetamol as well as antibiotics for the treatment of minor ailments and infections. If further evaluation or treatment is necessary, your child will be referred to the hospital arm in LMC.
3. Community benefit. This follow-up study extends the first malaria clinical vaccine trial that was conducted in Uganda. The information gained would provide an important addition to understanding how natural malaria infection can affect the usefulness of BK-SE36 as a vaccine candidate. Likewise, no study has yet been done on assessing the malaria status of the site; this study would thus help in evaluating the current methods for malaria control and eradication in the local community.

Duration of participation: 12 months

Number of subjects in the trial:
Ideally 66 subjects that was administered with BK-SE36 and 66 subjects under the control group (or equal number of BK-SE36 to control (placebo or non-vaccinated individuals)

Cost and Compensation:
For the volunteers who did not participate in Stage 2 clinical trial, if after these initial tests your child would not be able to participate due to health reasons, we will refer you to physicians at LMC.

The initial referral and first aid medicines will also be free. However, if long term (underlying) illness during screening is discovered, long term treatment and care will not be covered.

Your child will also receive, based on Ugandan standards 21,000.00 Ugandan shillings, per visit for transport refund.

Confidentiality:
We promise that all will be done to keep the results of this study confidential. In any report, your (or your child's) name will not appear but only his/her subject/volunteer code. A copy of this signed consent form will be placed in our file and a copy will be given to you. All files will be kept in locked cabinets and will only be seen by designated staff (including the Sponsor representatives) and representatives from Ugandan authorities (MBL-IRC, UNCST and NDA) for the verification of clinical trial procedures and/or data.

Circumstances under which participation may be terminated without consent:
The doctor may decide to stop participation due to:

1. Health conditions that might make your child's participation dangerous to his/her health
2. Any other conditions that might make continued participation dangerous to your child's health

Alternative to participation:
You (or Your child) may not participate. Medical care and health examination is available at LMC.

Consent:
Do you have questions taking part in this study? If you have any questions or concerns, or if you feel that you have been injured by taking part in this study you may contact Prof. Thomas Egwang, *Tel. no.*: +254-712-504 010; +256-785-60 5620. For any issues regarding your rights and welfare, please contact Prof. Edward Kirumira (Chairman MBL-IRC) on +256 752 767 439; +256 414 545 040. LMC contact numbers are: +256 392 948833 / +256 772 419397.

WHAT YOUR SIGNATURE OR THUMBPRINT MEANS
Your signature or thumbprint below means that you understand the information given to you in this consent form and that you agree for your child to participate in this trial. You will be asked to sign another informed consent form for the use and long term storage of blood samples.

____

Name of Volunteer (print) (Signature if applicable)

____

Gender Age on Informed Consent Address

____

Name of Parent/Guardian Relationship to Volunteer

____

Signature of Parent/Guardian (when applicable) Date/Time

Thumbprint of Volunteer if unable to sign:	Thumbprint of Parent/Guardian if unable to sign:

____
Name and Signature of Investigator   Date/Time

____
Language/Dialect for Informed Consent   Subject Code

Witness to Consent Interview and Signature/Thumbmark
On the date given next to my signature, I witnessed the "Informed Consent Interview" for the study. I attest that the information in these consent forms was explained to the volunteer/volunteer's parent(s)/guardian(s)] and that the volunteer/volunteer's parent(s)/guardian(s) indicated that his/her questions and concerns were adequately addressed; and their participation voluntarily given.

Signature of Witness ______________________________   Date ________________

Printed Name of Witness ____

ICF for Identification Photograph

Consent form for identification photograph:

I agree that the investigators may take photographs of me (or my child) for purposes of reliable identification during this research study only. I also understand that, at study completion, all such identification photographs will remain strictly confidential.

____ ___________________
Signature of Person Giving the Consent Explanation Date

____ ___________________
Volunteer name Signature of Volunteer (if applicable) Date

____
Parents' / Guardian's Name and Relationship to Volunteer (for volunteers 6-17 y of age)

____
Parents' / Guardian's Signature (for volunteers 6-17 y of age)

Thumbprint of volunteer if unable to sign:	Thumbprint of parent or guardian if unable to sign:

Signature of Witness ______________________________ Date ________________

Printed Name of Witness ____

ICF for Future Use of Blood Samples

INFORMED CONSENT FOR FUTURE USE OF BLOOD SAMPLES

Title: Longitudinal study of the immunogenicity of BK-SE36 candidate malaria vaccine in children and young adults that participated in the BK-SE36 malaria vaccine trial in Uganda

Local Principal Investigator: Prof. Thomas Egwang
(+254-712-504 010; +256-785-60 5620)

INTRODUCTION
While you (or your child) are in this study, blood samples are taken that will be useful for future research. These samples will be stored at Med Biotech Laboratories (MBL, Kampala) and in Japan. Samples may also be shared with investigators at other institutions.

WHAT SAMPLES WILL BE USED FOR
Your (or your child's) blood will be used to study malaria and the response of this disease to the test vaccine, BK-SE36. Results of these studies will not affect your (or your child's) care.

1. These samples will be used for ELISA, GIA, epitope mapping studies, HLA haplotype typing as well as molecular genotyping of malaria parasite to learn more about malaria and other diseases.
2. Your (or your child's) samples will be used only for research and will not be sold or used for the production of commercial products.
3. If additional studies are contemplated in the future, the investigators will first seek the approval of the MBL-IRC and UNCST.

LEVEL OF IDENTIFICATION
The samples obtained will be coded so that the subject's name cannot be readily identified. No information obtained from this research will be placed in any medical records.

In the future, researchers studying the samples may need to know more information such as age and gender and this information may be provided to the researcher. The subject's name or any information that might be used to identify the subject personally will, however, not be provided.

RISKS
There are few risks from future use of the samples. A potential risk might be the release of information from the study records. The study records will be kept confidential as far as possible. Moreover, results of research done with the samples will not be put in any subject's health record.

BENEFITS
There will be no direct benefit to the subjects. From studying the samples we may learn more about malaria or other diseases: how to prevent them, how to treat them, how to cure them.

RESEARCH RESULTS/MEDICAL RECORDS

1. Results from future research using the samples may be presented in publications and meetings but subject names will not be identified.
2. Reports from future research done with the samples will not be given to you or any doctor. No report will be reflected in any subject's medical record.

QUESTIONS
The future use of the blood samples has been explained to you (and your child) by the person who signed below and your (or your child's) questions were answered. If you have any other concerns about the information here, you may call Prof. Thomas Egwang (+254-712-504 010; +256-785-60 5620).

FREEDOM TO REFUSE
You (or your child) can withdraw this consent at any time. If you do, please contact Prof. Thomas Egwang (+254-712-504 010; +256-785-60 5620) at MBL, Kampala. The samples will no longer be made available for research and will be destroyed. Whether or not you (or your child) will allow us to use the blood samples in future research will not have any effect on your (or your child's) participation in the study.

WHAT YOUR SIGNATURE OR THUMBPRINT MEANS
Your signature (and your child's signature) or thumbprint below means that you understand the information that was given in this consent form. If you wish to allow the blood samples to be used for future research, you should sign or affix your thumbprint below.

____
Name of Volunteer (printed)

____
Signature of Volunteer (when applicable) Date/Time

____
Name of Parent/Guardian Relationship to Volunteer

____
Signature or Parent/Guardian (when applicable) Date/Time

____
Language/Dialect for Informed Consent Subject Code

____
Name and Signature of Witness Date

______________________________________ ________________
Signature of Person Giving the Consent Explanation Date

Thumbprint of parent or legal representative if unable to sign:	Thumbprint of parent or legal representative if unable to sign:

N. Questionnaire for Stage2 follow-up period; (Doctor's consultation guide)

Subject code		Date of Visit	
Residence: ☐ Same as last time<br>☐ Moved to new house_(new address)______________________			

Medical History
1. Malaria history from last visit: ☐ None ☐ Yes (date)_________________<br>1.1 Are there other household members who are suffering from malaria?<br>☐ None ☐ Yes,(number)______________<br>1.2 Use of mosquito bednet while sleeping: ☐ No ☐ Yes<br>1.3 Was the mosquito net used soaked or treated with an insecticide to kill or repel mosquitoes?<br>☐ No ☐ Yes<br>1.4 Has anyone sprayed the interior of the house against mosquitoes? ☐ No ☐ Yes
2. No of days symptomatic before diagnosis was made: ☐ Not known ☐_______days
3. Treatment obtained: ☐ In another hospital ☐ In the trial site previous visit<br>Details (include self medications)
Presenting features:<br>☐ Fever ☐ Malaise ☐ Headache ☐ Joint pains<br>☐ Vomiting ☐ Cough ☐ Diarrhoea ☐ Respiratory Distress<br>☐ Pallor ☐ Jaundice ☐ Bleeding ☐ Abdominal Pain<br>☐ Sweating ☐ Bleeding ☐ Convulsions ☐ Drowsy/Lethargy<br>☐ Rigor ☐ Other (please specify) ____
Blood smear: ☐ Negative ☐ Positive ______________
Relevant Laboratory Results:
Present Visit/Diagnosis ☐ Fine ☐ Not Fine
1. No of days symptomatic before this visit: ☐ Not known ☐_______days
Presenting features:<br>☐ Fever ☐ Malaise ☐ Headache ☐ Joint pains<br>☐ Vomiting ☐ Cough ☐ Diarrhoea ☐ Respiratory Distress<br>☐ Pallor ☐ Jaundice ☐ Bleeding ☐ Abdominal Pain<br>☐ Sweating ☐ Bleeding ☐ Convulsions ☐ Drowsy/Lethargy<br>☐ Rigor ☐ Other (please specify) ____

Blood smear: ☐ Negative ☐ Positive ______________
Vital Signs:

Body weight (kg)	Body height (cm)	
Blood pressure / ▯ mmHg	Pulse rate bpm	Body Temp (°C)

Relevant Laboratory Results: ☐Not Applicable
Management /Treatment/Prescriptions (including dosage and frequency):
Outcome:
At the end of this consultation, we request that a few drops of blood from a finger/earlobe be drawn. The test uses disposable sterile instruments that are clean and completely safe. The blood will be used for immunogenicity tests and the results of the test will not in anyway breach your (or your child's) confidentiality.

O. Appendix 1. Justification for study objectives and methodology.

Long term immuno-epidemiological studies maybe viewed as the most appropriate means to understand the critical characteristics of the interactions between man and parasite. This is a necessary prerequisite to the development of BKSE-36 as a malaria vaccine candidate. It is believed that the development of protective immunity against malaria is slow and to be maintained, it requires exposure to multiple antigenic variants of malaria parasites and age-associated maturation of the immune system. Evidence that the protective immunity is associated with different classes and subclasses of antibodies reveals the importance of considering the quality of the response. In this study we aim to evaluate the immune response of BK-SE36 administration compared to/ and under the influence of natural infection.

To assay additional malaria antigens, in addition to SE36, would serve as controls to be enable us to understand, analyze and literally dissect malaria-related events occurring in the study population used in the BK-SE36 trial. It aims to answer questions like:

- What antibodies can correlate to reduced risk of infection?
  Only antibody titers to SE36?
- Can increase titers of BK-SE36 show some association with malaria infection?
- Is the association similar if we assay other malaria antigens that are known to be polymorphic (e.g. MSP-1, AMA-1, CSP-1)?
- Evaluations of vaccine-efficacy studies can also be complicated by parasite polymorphisms. Is the study population exposed to different *P. falciparum* genotypes?
- Are titers of SE36 antibody compromised by existing infection?
- Is the immune response more efficacious to that which is induced by other antibodies due to natural infection?

Clearly, vital information can be gained from the follow-up study of subjects administered with BK-SE36. Should BK-SE36 be deemed safe enough to proceed to further trials, a careful investigation of immune responses in the population relative to other malaria antigens might highlight the strengths and weaknesses of the present formulation of BK-SE36. This may inform and guide further development of the vaccine.

Evaluating the potential of new vaccine candidates by reliable and sensitive laboratory methods is also mandated by GCP guidelines. Priority is on robust assays of functional immune responses which could inform vaccine development. Although the potential of growth inhibition assay (GIA) and antibody-dependent cellular inhibition assay (ADCI) has been recognized by malaria immunologists, these methods are not validated in the field. Antibodies have been noted to reduce parasitemia, or limit the growth of *P. falciparum* in infected red blood cells in culture (GIA). When tested *in vitro*, immunoglobulins were not inhibitory on their own but were dependent on monocytes (ADCI). Thus, protective antibodies may be acting indirectly. We take this initial step to address technologies that can facilitate further evaluation of BK-SE36 as a promising vaccine candidate, and at the same time take the challenge for further harmonization and standardization of robust quantitative assays that can help malaria vaccine development in general.
